# The Subtelomeric *khipu* Satellite Repeat from *Phaseolus vulgaris*: Lessons Learned from the Genome Analysis of the Andean Genotype G19833

**DOI:** 10.3389/fpls.2013.00109

**Published:** 2013-10-16

**Authors:** Manon M. S. Richard, Nicolas W. G. Chen, Vincent Thareau, Stéphanie Pflieger, Sophie Blanchet, Andrea Pedrosa-Harand, Aiko Iwata, Carolina Chavarro, Scott A. Jackson, Valérie Geffroy

**Affiliations:** ^1^UMR-CNRS 8618, Saclay Plant Sciences, Institut de Biologie des Plantes, Université Paris SudOrsay Cedex, France; ^2^Université Paris Diderot, Sorbonne Paris CitéParis, France; ^3^Laboratory of Plant Cytogenetics and Evolution, Department of Botany, Universidade Federal de Pernambuco, Rua Nelson Chaves s/nRecife, Pernambuco, Brazil; ^4^Center for Applied Genetic Technologies, Institute for Plant Breeding, Genetics, and Genomics, University of GeorgiaAthens, GA, USA; ^5^Unité Mixte de Recherche de Génétique Végétale, Institut National de la Recherche AgronomiqueGif-sur-Yvette, France

**Keywords:** Common bean, satellite DNA, tandem repeat, evolution, FISH, genome sequencing, centromere, subtelomere

## Abstract

Subtelomeric regions in eukaryotic organisms are known for harboring species-specific tandemly repeated satellite sequences. However, studies on the molecular organization and evolution of subtelomeric repeats are scarce, especially in plants. *Khipu* is a satellite DNA of 528-bp repeat unit, specific of the *Phaseolus* genus, with a subtelomeric distribution in common bean, *P. vulgaris*. To investigate the genomic organization and the evolution of *khipu*, we performed genome-wide analysis on the complete genome sequence of the common bean genotype G19833. We identified 2,460 *khipu* units located at most distal ends of the sequenced regions. *Khipu* units are arranged in discrete blocks of 2–55 copies and are heterogeneously distributed among the different chromosome ends of G19833 (from 0 to 555 *khipus* units per chromosome arm). Phylogenetically related *khipu* units are spread between numerous chromosome ends, suggesting frequent exchanges between non-homologous subtelomeres. However, most subclades contain numerous *khipu* units from only one or few chromosome ends indicating that local duplication is also driving *khipu* expansion. Unexpectedly, we also identified 81 *khipu* units located at centromeres. All the centromeric *khipu* units belong to a single divergent clade also comprised of a few units from several subtelomeres, suggesting that a few sequence exchanges between centromeres and subtelomeres took place in the common bean genome. The divergence and low copy number of these centromeric units from the subtelomeric units could explain why they were not detected by FISH (Fluorescence *in situ* Hybridization) although it can not be excluded that these centromeric units may have resulted from errors in the pseudomolecule assembly. Altogether our data highlight extensive sequence exchanges in subtelomeres between non-homologous chromosomes in common bean and confirm that subtelomeres represent one of the most dynamic and rapidly evolving regions in eukaryotic genomes.

## Introduction

Common bean (*Phaseolus vulgaris*) is a major source of protein for human consumption in many parts of the world (FAO 1980), especially in developing countries such as tropical areas of Latin America and Eastern Africa where common bean is one of the major staple crops (Pastor-Corrales and Tu, 1989; Broughton et al., 2003). Together with sorghum, millet, groundnut, cowpea, chickpea, pigeonpea, cassava, yam, and sweet potato, common bean is often referred to as an “orphan crop.” Indeed, even if common bean is an important crop in developing countries, it is not extensively traded and receives less attention from researchers compared to crops such as maize, rice, and wheat (Varshney et al., 2012). Common bean has a small diploid genome (2*n* = 22) of 588 Mb (Bennett and Leitch, 1995) including a large amount of repeated sequences (Schlueter et al., 2008; Pedrosa-Harand et al., 2009) compared with other legume species with larger genome sizes, such as *Trifolium repens* (956 Mb; Bennett and Smith, 1991) and soybean (1,103 Mb; Bennett and Leitch, 1997). Recently, the revolution in sequencing technologies has allowed the establishment of full genome sequencing programs for orphan crops like common bean and the full genome is now available (since July 2012^1^; Jackson et al., in preparation). The selected common bean genotype is “G19833,” an Andean landrace for which a BAC library was used to construct a draft physical map (Schlueter et al., 2008).

Satellite DNA can be defined as highly reiterated non-coding DNA sequences, organized as long arrays of head-to-tail linked repeats located in the constitutive heterochromatin (Plohl et al., 2008). Despite their ubiquity in eukaryotic genomes, the function of such repeats is poorly understood. Early hypotheses considered them to be non-functional “selfish” DNA that proliferate for their own sake or as useless genomic elements accumulated as “junk” with no selective advantage to the organism (Ohno, 1972; Orgel and Crick, 1980). More recently, identification of satellite DNA at structurally important parts of chromosomes, such as centromeres, has suggested functional roles of satellite DNA (Ma and Jackson, 2006).

Satellite DNA is an important component of the knobs, which are cytologically visible regions of highly condensed chromatin (heterochromatin) that are distinct from pericentromeric regions in pachytene chromosomes (Fransz et al., 2000). In common bean, a 528-bp subtelomeric satellite repeat, referred to as *khipu*, has been identified (David et al., 2009). *Khipu* is present on most chromosomal terminal knobs and is specific to the *Phaseolus* genus (David et al., 2009; Geffroy et al., 2009). Subtelomeric satellite repeats have been reported in different plant and animal species. Indeed, cytologically confirmed subtelomeric satellite repeats have been identified in various plant species, including potato (Torres et al., 2011), rice (Cheng et al., 2001), tomato (Lapitan et al., 1989), maize (Li et al., 2009), barley (Brandes et al., 1995), tobacco (Kenton et al., 1993; Chen et al., 1997), rye (Vershinin et al., 1995), *Silene latifolia* (Buzek et al., 1997), and *Beta* species (Dechyeva and Schmidt, 2006). The subtelomeric locations of these repeats were confirmed mostly by FISH (fluorescence *in situ* hybridization) experiments and were not based on sequence analysis. Except in rice, where sequencing and characterization of the structure of the subtelomeric TrsA sequences were conducted (Ohmido and Fukui, 1997; Mizuno et al., 2006, 2008), sequence-based analysis of the molecular organization and evolution of subtelomeric repeats is rare.

In the present paper, we conducted genome-wide analysis to investigate the physical organization and the evolution of *khipu* sequence based on the complete genome sequence of common bean genotype G19833.

## Materials and Methods

### Data sources

We used the “*Phaseolus vulgaris* v1.0” genome sequence of the Andean common bean genotype G19833^2^ and sequenced BAC clones from G19833 reported in Innes et al. (2008) that correspond to ∼1 Mb of the *Co-2* cluster, located at the end of the long arm of chromosome 11, and from Chen et al. (2010), corresponding to 239 kb located at one end of chromosome 5, referred to as PvA05A.

### *Khipu* annotation

The *khipu* satellite DNA was recovered using hmmsearch^3^ (Eddy, 1998) with a *khipu* profile previously defined on 92 *khipu* (David et al., 2009). In order to work with a “clean” set of *khipu*, we excluded the first and last *khipu* element from blocks of tandemly organized *khipu*. In addition, we excluded *khipu* < 500 pb and having “*n*”s in their sequence. The resulting data was imported into the annotation platform Artemis for manual analysis (Rutherford et al., 2000).

Centromeric positions in individual pseudomolecules were identified by BLASTN using centromere satellite repeats; CentPv1 for chromosomes 01, 02, 03, 04, 07, 08, 09, and 10 and CentPv2 for chromosomes 05, 06, and 11 (Jackson et al., in preparation). Each *khipu* sequence extracted from the genome of G19833 was named Pvxxyk#####, with Pv referring to *Phaseolus vulgaris*, “xx” referring to pseudomolecule (01–11), “y” corresponding to the location on the chromosome (S for short arm, C for centromere, and L for long arm), “k” referring to “*khipu*,” and a 5-digit number referring to the *khipu* order on the pseudomolecules from the start (5′) to the end (3′). *khipu* elements on each pseudomolecule were sequentially numbered in increments of 10. Each *khipu* extracted from BAC sequences was named PvAxxzk##### (Axxz is the name of the contig), with Pv referring to *Phaseolus vulgaris*, A referring to Andean (these BAC come from the Andean genotype G19833), xx referring to chromosome (05 or 11), z is a letter given to the different contigs from the same chromosome, k referring to *khipu*, and 5-digit number referring to the *khipu* order on the contig in increments of 10. For example, *khipu* named Pv01Sk00010 is the first *khipu* unit on pseudomolecule 01, located on short arm and PvA05Ak00010 is the first *khipu* unit of the BAC contig A from chromosome 05.

To determine the coordinates of BACs on the pseudomolecules, we performed a BLASTN of the entire BAC sequence against the genome sequence. BLAST results were inspected manually to set the start and end positions of the BAC on the pseudomolecules. BAC sequences and pseudomolecules were aligned and visualized using Mauve, a genome alignment tool, using the minimal match seed weight value^4^ (Darling et al., 2004).

### Phylogenetic analysis

Multiple sequence alignments of *khipu* sequences were generated using Muscle (Edgar, 2004a,b) with gapopen = −1000 and MaxIter = 3. Optimized alignement is provided in Figure S1 in Supplementary Material. Graphical representation of the *khipu* alignment was visualized using the WebLogo server^5^ (Crooks et al., 2004) (Figure S2 in Supplementary Material). Recombination among loci was assessed using several methods implemented in RDP v.3.15 (Martin et al., 2005b): RDP (Martin and Rybicki, 2000), Geneconv (Padidam et al., 1999), Chimera (Posada and Crandall, 2001), and Bootscan (Martin et al., 2005a). Default parameter settings were used for each method except as follows: RDP (internal reference sequence), Bootscan (window = 150, step = 20, NJ trees, 200 replicates, 95% cutoff, J&N model with Ti:Tv = 2, coefficient of variation = 2). The maximum *p*-value for accepting recombination was set at 0.001 (after Bonferroni correction).

A Maximum-Likelihood tree was made with FastTree 2.1.3 program (Price et al., 2010) with the Jukes–Cantor model of nucleotide evolution. Bootstrap values were computed with the consensus of 100 random trees using the Phylip’s consense program (Felsenstein, 1989) and the random trees were calculated with the “−*n*” option of the FastTree program over a list of bootstrapped sequences generated from the original sequence alignment using Seqboot in the PHYLIP package. The resulting phylogenetic tree (Figure S3 in Supplementary Material) was displayed using MEGA version 5 (Tamura et al., 2011). One representative *khipu* sequence from each major clade of the phylogenetic tree, is provided in Figure S2 in Supplementary Material.

### Cytogenetic analysis

The *khipu* probe was generated using a pool of five subclones from BAC clones (Table A1 in Appendix). Three subclones come from different *khipu* blocks spread over sequenced BAC clones from the long arm of chromosome 11 (Innes et al., 2008), one additional subclone come from a subtelomeric BAC from the short arm of chromosome 5 (Chen et al., 2010), and the 1H04 subclone come from the *B4* locus (short arm of chromosome 4 from the Mesoamerican BAT93 genotype) described in David et al. (2009). Pachytene chromosomes were prepared from young flower buds of G19833 and JaloEEP558 fixed in ethanol:acetic acid (3:1, v/v). Buds were macerated in 2% cellulase/2% pectolyase/2% cytohelicase in 0.01 M citric acid-sodium citrate buffer, pH 4.8, for 3 h at 37°C, incubated in 60% acetic acid up to 2 h, and squashed after removal of petals and sepals and flaming. Slide selection and pretreatment, chromosome and probe denaturation and hybridization, posthybridization washes, detection, and image analyses were performed according to Fonseca et al. (2010).

## Results and Discussion

### Genome-wide identification of the *khipu* satellite repeat in the pseudomolecules of common bean G19833

To identify *khipu* sequences in the common bean genome, we used the *P. vulgaris* genome v1.0 from Phytozome^6^. A total of 2766 *khipu* units were identified. After size selection of *khipu* units > 500 bp and discarding *khipu* units with “*n*”s, we had 2460 *khipu* units for further analysis. The distribution of these 2460 *khipu* units in the common bean genome is presented in Table 1. The short arm of chromosome 01 (chr01S), Chr04S, Chr04L (L = long arm), Chr05S, Chr10L, and Chr11L, had the largest number of *khipu* units, with 193, 349, 261, 169, 435, and 555, respectively. Chr08S and Chr09L, however, had fewer than 10 *khipu* units and Chr06S and Chr09S were devoid of *khipu*. These data are in general agreement with the cytogenetic distribution of BAC 63H6, which contains *khipu* (K.G.B. dos Santos, personal communication) in G19833 (Altrock et al., 2011), except that Chr10S and Chr11S seem to have large amounts of *khipu* (as estimated based on the intensity of FISH signals) and no *khipu* signal could be detected in Chr09L. In agreement with previous FISH analysis on BAT93 showing the subtelomeric distribution of *khipu* (David et al., 2009), *khipu* units were mainly located in the first or last five megabase-pairs of the pseudomolecules. These *khipu* were organized in tandem arrays with varying numbers units, referred to as *khipu* blocks. For example, chr04S had 28 *khipu* blocks containing fewer than 13 units and nine blocks containing more than 13 units, within the first 4.7 Mb of the pseudomolecule. In rice, similar organization in discrete clusters of 3–103 copies in a chromosome specific manner was also observed for the TrsA subtelomeric repeats (Mizuno et al., 2008). In common bean, the largest *khipu* blocks were found on Chr04S and Chr11L, where *khipu* blocks had 45 and 55 *khipu* units, respectively. Notably, Chr04S and Chr11L contain the *B4* (David et al., 2009) and *Co-2* disease resistance gene clusters (Innes et al., 2008; David et al., 2009; Chen et al., 2010) suggesting a possible link between the evolution of resistance clusters and the *khipu* sequences. As expected for a subtelomeric repeat, few or no *khipu* (<13) were identified in centromeric regions, with the exception of chromosome 8 centromere which had 55 *khipu* sequences.

**Table 1:** **Number of complete *khipu* units in each pseudomolecule of *Phaseolus vulgaris***.

Pseudomolecules	Short arm	Centromere	Long arm	Total
Chr01	193	0	19	212
Chr02	46	0	39	85
Chr03	60	3	17	80
Chr04	349	13	261	623
Chr05	169	0	33	202
Chr06	0	0	17	17
Chr07	17	7	14	38
Chr08	7	55	51	113
Chr09	0	2	10	12
Chr10	71	1	435	507
Chr11	16	0	555	571
Total		81		2460

### Spread of *khipu* to non-homologous chromosome ends

**Figure 1:**
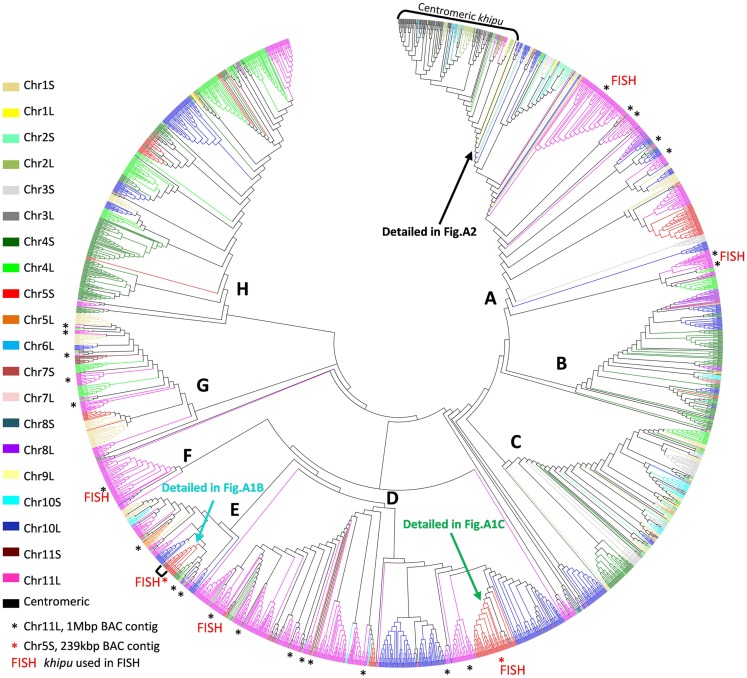
**Comparison between phylogeny and physical distribution of *khipu* repeats within the G19833 genome**. Phylogenetic tree of the 2460 *khipu* repeats from the G19833 genome sequence. The eight major clades are indicated with bold letters **(A–H)**. Each color corresponds to a chromosome arm subtelomeric region named by a number, corresponding to the chromosome number, followed by the letter L (long arm) or S (short arm). Note that centromeric *khipu* repeats (black) are found only in two small subclades highlighted by brackets. Clades comprising *khipu* repeats from previously sequenced BAC contigs from chromosome 11 long arm or chromosome 5 short arm are highlighted by black or red asterisks, respectively. Clades comprising *khipu* repeats used for FISH experiments are highlighted by FISH written in red.

**Figure A1:**
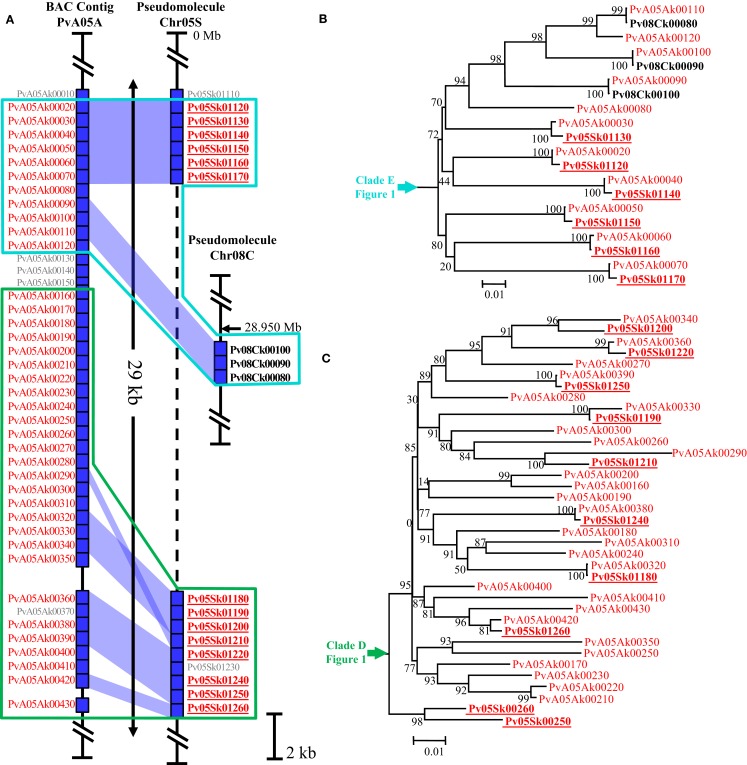
**Comparison of khipu containing region of the genome with the corresponding BAC contig and phylogenetic analysis of khipu sequence**. **(A)** Schematic representation of 29 kb of PvA05A (239 kb), containing *khipu*, with the corresponding region of pseudomolecule 5 and with part of the centromeric region of chromosome 8 (coordinates: 28.950 Mb). Sequences of these regions are represented by vertical black lines with dotted line represent a gap in the sequence. Blue rectangle represent annotated *khipu* of the region with their name written on each side, in red for *khipu* from PvA05A sequence, bold red and underlined for *khipu* from the pseudomolecule 5 and bold black for centromeric *khipu* of pseudomolecule 8. *Khipu* in small gray type correspond to *khipu* shorter than 500 bp that were discarded from analysis. The blue area represents the correspondence between *khipu* from BAC sequence and *khipu* from genome sequence according to the phylogenetic tree. **(B)** and **(C)** Phylogenetic tree clades of *khipu* present on **(A)**. **(B)** Part of the clade called “E” (*cf*. Figure 1) **(C)** Part of the clade called “D” (cf. Figure 1)

To study *khipu* satellite evolution in the common bean genome, a phylogenetic tree was constructed (Figure 1). A multiple alignment including the 2460 *khipu* units from the G19833 genome plus 201 units from G19833 BACs (Innes et al., 2008; Chen et al., 2010) were first screened for recombination events using RDP4 and recombinant sequences removed (76 and 4 *khipu* units from G19833 genome and BACs, respectively). *Khipu* units fell into eight major clades (A–H) with Bootstrap support >75% (Figure 1). Except for the small clade F that contains only *khipu* units from Chr11L, Chr04S and Chr10L, each major clade contains *khipu* units from most chromosome ends. This indicates that phylogenetically related *khipu* units were spread among the chromosome ends, suggesting frequent exchanges between non-homologous subtelomeres. Within a chromosomal cluster, *khipu* units were found across the tree, indicating that phylogenetically distant *khipu* units are physically close to each other. This is particularly striking for Chr10L (blue) where *khipu* elements are spread across the eight clades. The distribution of *khipu* units coming from BAC clones reinforces these results. For example, *khipu* units coming from the BAC from Chr11L (∼1 Mbp; Innes et al., 2008) are spread across 24 subclades belonging to five of the eight major clades (black asterisks in Figure 1). Thus, almost the entire *khipu* diversity is represented in a single genomic region as small as ∼1 Mbp. Additionally, BAC PvA05A (239kbp; Chen et al., 2010) from Chr05S contains a *khipu* block bearing phylogenetically distant *khipu* units (Figure 1; Figure A1 in Appendix). Indeed, this *khipu* block is composed of 11 complete units from major clade E (light blue, PvA05Ak00020–PvA05Ak00120) followed by an array of 27 *khipu* units from major clade D (green, PvA05Ak00160–PvA05Ak00430).

Together these results indicate that each subtelomere contains a patchwork of phylogenetically distant *khipu* units that is likely the result of shuffling between non-homologous chromosome ends. However, most subclades contain numerous *khipu* units from only one or few chromosome ends (Figure 1). Thus, in addition to *khipu* spreading between non-homologous loci, local duplication is driving *khipu* expansion. In the human genome, extensive cytogenetic and sequence analyses revealed that subtelomeres are hot spots of interchromosomal recombination and segmental duplications (Linardopoulou et al., 2005). This exceptional dynamic activity of subtelomeres has been reported in such diverse organisms as yeast and the malaria parasite *Plasmodium* (Louis, 1995; Freitas-Junior et al., 2000, 2005). As expected for a plastic region of the genome subject to reshuffling through recombination events, subtelomeres exhibit unusually high levels of within-species structural and nucleotide polymorphism (Mefford and Trask, 2002). In plants, this plasticity of subtelomeres was not found in *Arabidopsis thaliana* (Heacock et al., 2004; Kuo et al., 2006) and, to our knowledge, has not been reported for any other sequenced plant species.

### Quality of *khipu* sequences within the G19833 genome

**Figure 2:**
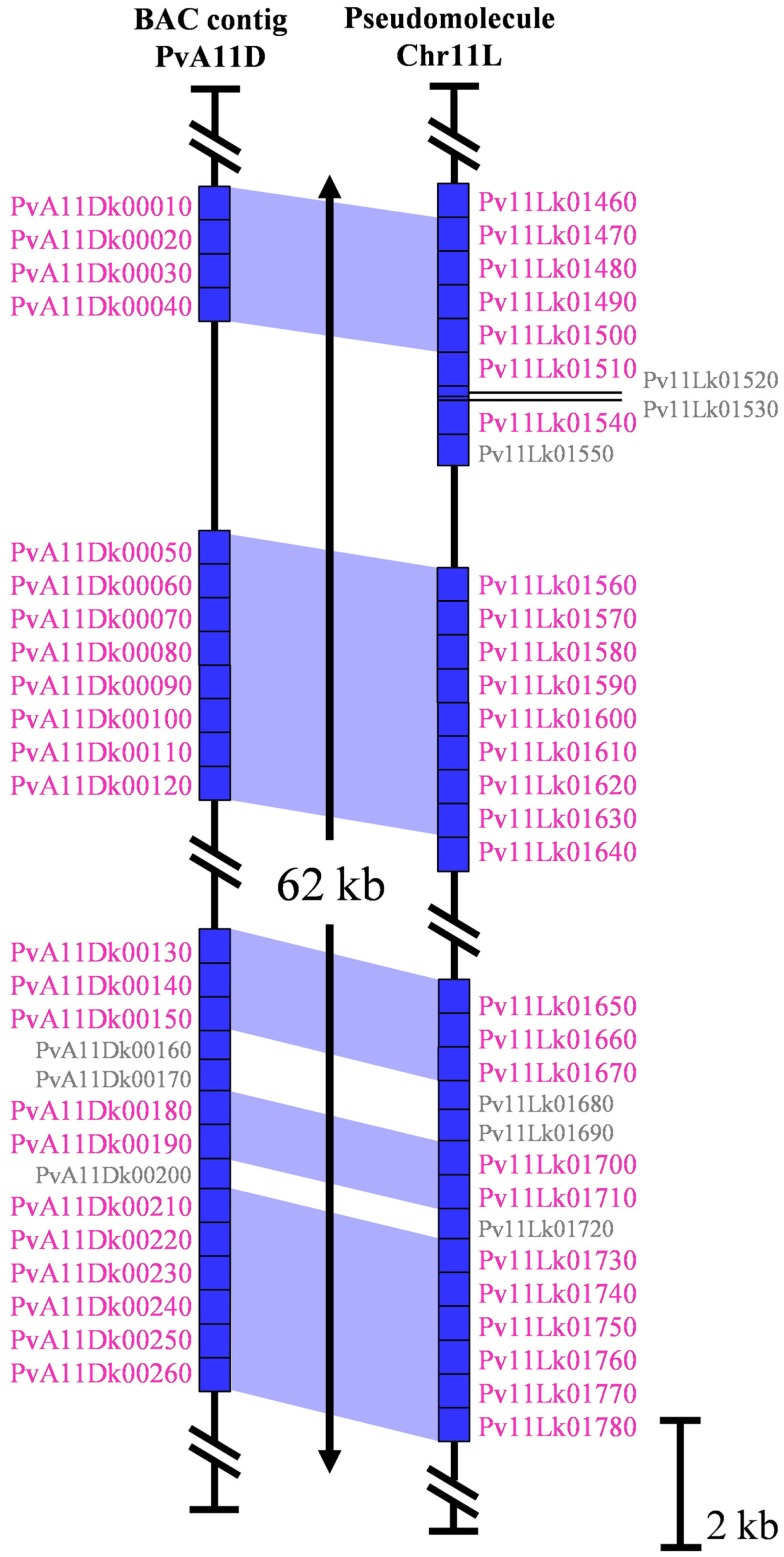
**Schematic representation of 62 kb of PvA11D (200 kb), containing *khipu*, with the corresponding region of pseudomolecule 11**. Sequences of these regions are represented by vertical black lines. Blue rectangle represent annotated *khipu* of the region with their name written on the side of each one. *Khipu* with name written in small gray correspond to *khipu* shorter than 500 bp discarded from the analyses. The blue areas represent correspondences between *khipu* from BAC sequence and *khipu* from genome sequence according to the phylogenetic tree.

Genomic regions containing highly repetitive sequences, especially tandem arrays of satellite repeats, constitute a challenge for short-read, whole-genome shotgun sequencing and are thus considered to be error-prone regions of whole-genome sequencing projects (Jackson et al., 2011). In order to check the quality and the consistency of the G19833 pseudomolecules for *khipu*, we compared pseudomolecules sequence data with six G19833 BACs (sequenced by the classical Sanger method) containing *khipu* sequences and coming from two distinct genomic regions (Innes et al., 2008; Chen et al., 2010, unpublished data). Here we present in details the results from two BAC contigs: PvA11D is a 200 kbp clone corresponding to the coordinates 47.3–47.5 Mb of the long arm of pseudomolecule 11 (Chr11L) (Figure 2) while PvA05A is a 239 kbp clone corresponding to the coordinates 1.0–1.2 Mb of the pseudomolecule 5 (Chr05S) (Figure A1A in Appendix). In these regions, apart from *khipu* blocks, sequences share over 98% nucleic identity between BAC contigs and pseudomolecule sequences (data not shown). A combination of phylogenetic and genomic analyses shows that most *khipu* units are identical in position and sequence in both the pseudomolecules and BACs (blue areas in Figure 2; Figure A1A in Appendix). For example, *khipus* from PvA11D and its pseudomolecule counterpart are nearly identical (Figure 2). Similar results were obtained with four other BAC contigs containing *khipu* units (data not shown), confirming that genome data is of high quality even for *khipu* containing regions. However, PvA11D contains *khipu* blocks of only 14 *khipu* units (∼7400 bp) and a different situation is observed for PvA05A which bears a larger *khipu* block of more than 35 *khipu* units (Figure A1 in Appendix). Of these, six (PvA05Ak00020–PvA05Ak00070) are completely identical with corresponding region of Chr05 pseudomolecule, but 21 (PvA05Ak00080, PvA05Ak00120, PvA05Ak00300, PvA05Ak00310, PvA05Ak00350, PvA05Ak00400, PvA05Ak00410, PvA05Ak00430, and PvA05Ak00160–PvA05Ak00280) are completely absent in the whole-genome sequence, resulting in a ∼10 kbp gap (Figure A1A in Appendix). Moreover, three *khipu* units (PvA05Ak00090, PvA05Ak00100, and PvA05Ak00110) from this Chr05 BAC contig share 100% nucleotide identity with three *khipu* units from pseudomolecule 8 centromeric region (Pv08Ck00100, Pv08Ck00090, and Pv08Ck00080) (Figures A1A,B in Appendix). Because we were unable to find corresponding *khipu* units from Chr05S, it is possible that these three Chr08 centromeric *khipu* are the result of errors in the assembly. Even though BACs are considered to be stable cloning vectors, it has been reported that tandem repeats can be unstable in BAC clones (Song et al., 2001). In conclusion, comparisons between BACs and the genome sequence data suggest that small arrays of satellite units can be well-resolved, while larger satellite blocks may be slightly more error prone. However, based on six independent BACs/genome comparisons, we conclude that genome data is of high quality to conduct genome-wide analysis o *khipu* sequences.

### Centromeric *khipu* units: Divergent *khipu* sequence and/or erroneous assembly?

**Figure A2:**
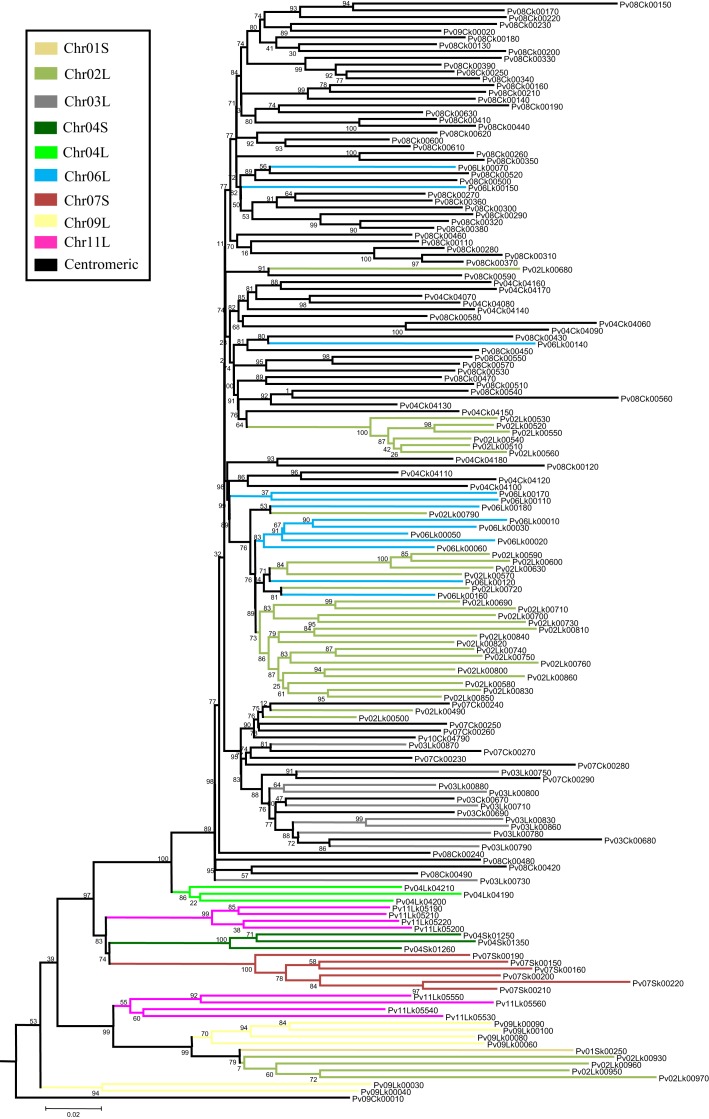
**Detailed view of a subtree from the phylogenetic tree presented in Figure 1 containing centromeric khipu units**. Branch colors correspond to the colors defined in Figure 1. Note cases of subtelomeric khipu units from Chr02L, Chr03L, and Chr06L interspersed with centromeric khipu units (black), indicating movement of *khipu* units between subtelomeres and centromeres.

Except for the three potentially false centromeric *khipu* units (identical to subtelomeric *khipu* units from Chr05S, Figures A1A,B in Appendix), all 78 centromeric *khipu* units belong to a single well-defined subclade that also includes subtelomeric units from Chr06L, Chr03L, and Chr02L (Figure 1; Figure A2 in Appendix) which indicates that they are less diverse than the subtelomeric units. It is surprising that no centromeric signals were found in previous FISH experiments on mitotic chromosomes, using either a *khipu*-specific probe in the BAT93 common bean genome (David et al., 2009; Geffroy et al., 2009) or a *khipu*-bearing subtelomeric BAC clone in G19833 (Altrock et al., 2011). These conflicting results raise the question of whether the centromeric *khipu* units are real centromeric sequences or misassembled sequences.

**Figure A3:**
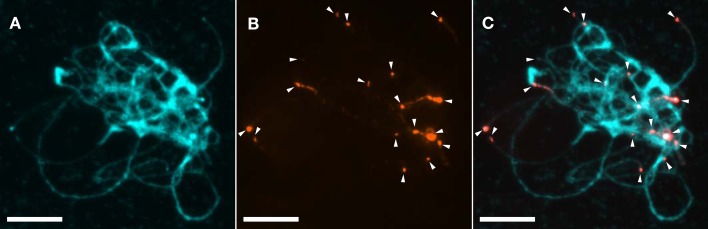
**Physical distribution of khipu on G19833 pachytene chromosomes using FISH**. FISH to G19833 pachytene chromosomes. *Khipu* was used as a probe in **(B)** and **(C)**. Signals are in red, and chromosomes in blue in **(A)** and **(C)**. The 17 chromosome termini with *khipu* signals are indicated by white arrowheads. Bars = 10 mm.

**Figure A4:**
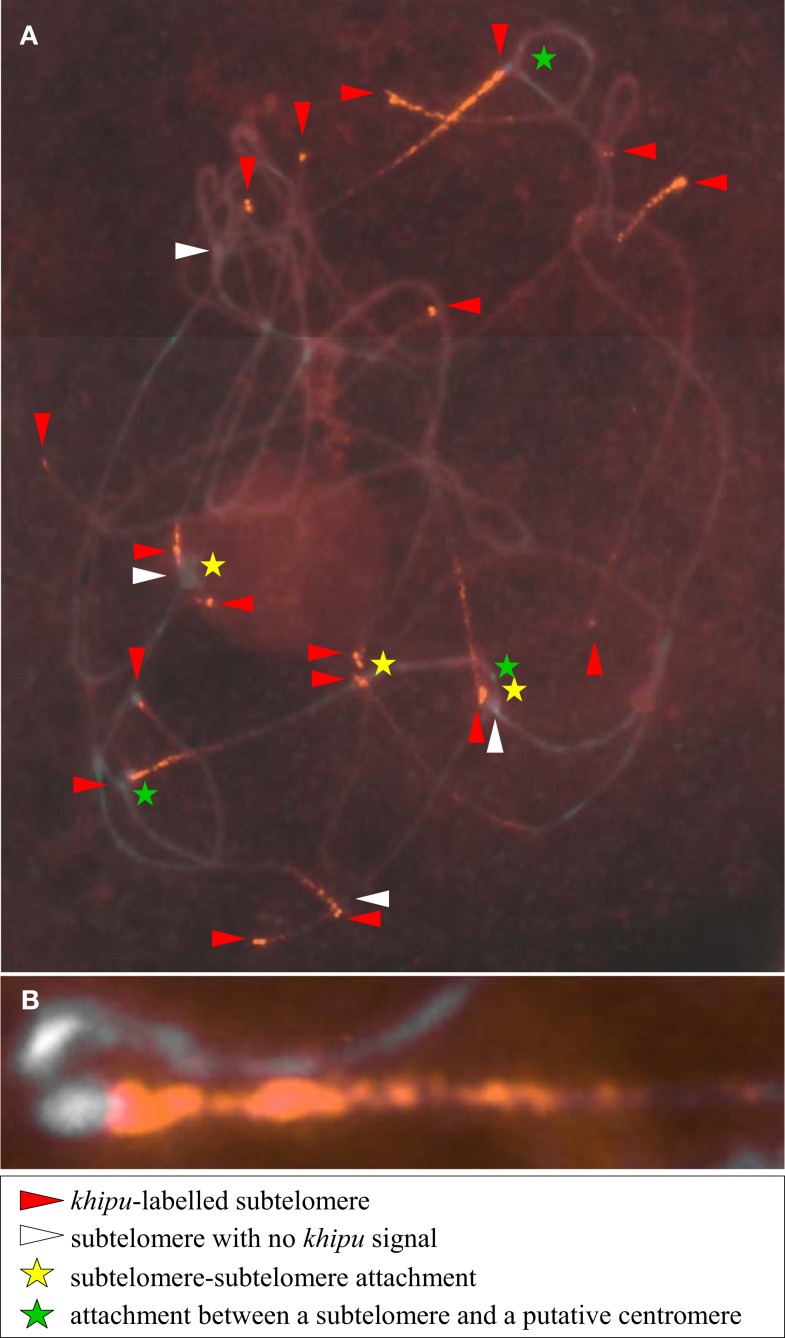
**Localization of khipu satellite DNA on JaloEEP558 pachytene chromosomes and evidences of chromosome attachments**. Chromosomes are counterstained with DAPI (gray), and *khipu* signals are in red (original picture). **(A)** Chromosome termini with or without *khipu* labeling are indicated by red or white arrowheads, respectively. Stars indicate putative chromosome attachments. **(B)** Magnified view of an attachment between two non-homologous chromosomes.

To try to solve this puzzle, we performed FISH on pachytene chromosomes from G19833, using a *khipu* FISH probe containing a wide diversity of *khipu* units (highlighted by “FISH” written in red in Figure 1; Table A1 in Appendix). We detected *khipu* signals on 17 chromosome ends, but found no evidence of centromeric signals in G19833 chromosomes (Figure A3 in Appendix). This result is similar to *khipu* distribution at 17 chromosome ends in the BAT93 genotype (David et al., 2009) suggesting that *khipu* distribution has been stable since the split between Andean and Mesoamerican gene pools. Interestingly, during these FISH experiments heterochromatin connections between non-homologous chromosomes were observed (Figure A4 in Appendix), providing indirect evidence that subtelomeres are hot spot of interchromosomal recombination. Similar attachments were observed in rye (Gonzalez-Garcia et al., 2006). In common bean, according to whole-genome data Chr08C comprises 55 *khipu* (Table 1). This begs the question: why was not it possible to detect these 55 centromeric *khipu* units by FISH?

**Figure A5:**
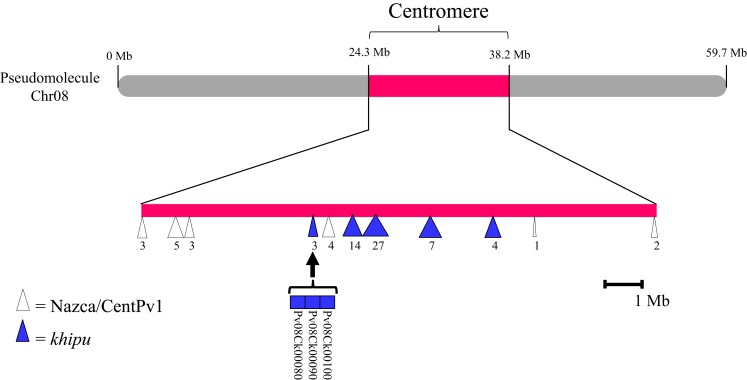
**Organization of satellite DNA Nazca/CentPv1 and khipu in the centromeric region of chromosome 08**. Chromosome 08 is represented in gray and the centromeric region in pink. The centromeric part is enlarged for positioning satellite DNA. Nazca/CentPv1 (centromeric repeat, Iwata et al., submitted) and *khipu* are represented by white and blue triangles, respectively. The number of repeated units inside each block is indicated below. The three *khipu* of the first block are detailed with their names indicated below. These are *khipu* phylogenetically clustered with three *khipu* of the BAC contig PvA05A presented in Figure A1.

**Figure A6:**
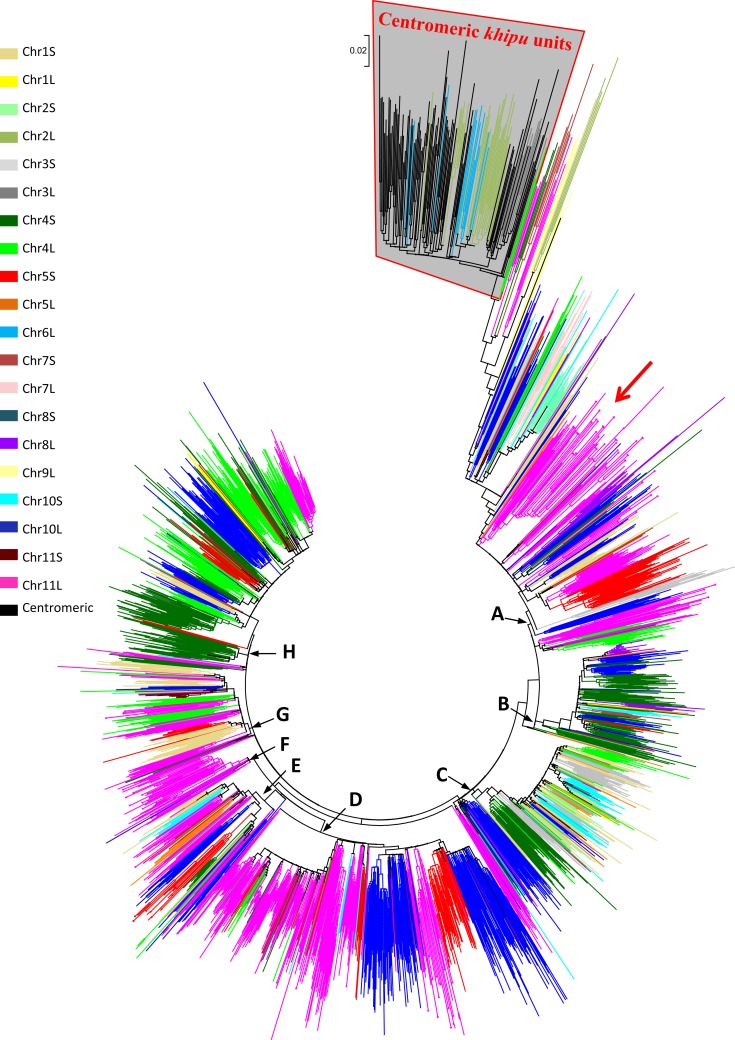
**Phylogenetic tree presented in Figure 1 with branch length**. Clade names **(A–H)** correspond to the clades defined in Figure 1 and branch colors correspond to the colors defined in Figure 1. The subclade containing the centromeric *khipu* units is boxed in gray. From this clade, the closest *khipu* unit used for FISH experiments is indicated by a red arrow.

There are four potential explanations. The first hypothesis is that *khipu* units were grouped at Chr08C due to biases during assembly of the pseudomolecule. A comparison to BAC contig PvA05A allowed us to find three *khipu* units from Chr05S subtelomeric region that were assembled at Chr08C by mistake during pseudomolecules assembling (Figures A1A,B in Appendix). What about the other centromeric *khipu*? As previously discussed, the genome assembly is of high quality and the major differences to BAC sequences are numbers of *khipu* units within a *khipu* block rather than a wrong location of *khipu* blocks (Figure 2; Figure A1 in Appendix). In addition to Chr08C, we also found *khipu* units in centromeres of Chr03, Chr04, Chr07C, Chr09, and Chr10 (Figure A2 in Appendix). It is unlikely that these *khipu* units, clustered in a single subclade, would be misassembled at the centromere of various pseudomolecules. Moreover, it is not unusual to find non-centromeric defined repeats in centromeric regions in eukaryotes. Notably, detection of DNA sequences that are partially homologous to telomeric repeats in centromeres has been reported in many species including *Drosophila melanogaster* (Abad et al., 2004; Mendez-Lago et al., 2009), maize (Alfenito and Birchler, 1993; Jin et al., 2005), and in potato (Tek and Jiang, 2004; He et al., 2013). Moreover, in rice, a satellite DNA present both in subtelomeric and centromeric regions has also been identified (Lee et al., 2005; Bao et al., 2006). A second hypothesis to explain the absence of *khipu* centromeric FISH signal is that *khipu* units from Chr08C are spread across a wide region of the centromere (13.8 Mb), thus diluting the signal in FISH. However, according to the pseudomolecule, the *khipu* units are grouped at the very center of Chr08C, with one block of 27 *khipu* units which should have been detected by FISH (Figure A5 in Appendix). The third hypothesis is that centromeric *khipu* are too divergent from the *khipu* units used for FISH experiments (Figure A6 in Appendix). Even if our FISH probe presents a wide diversity of *khipu* units (as shown in Figure 1 with “FISH” written in red), the closest *khipu* unit used in FISH (red arrow in Figure A6 in Appendix) shares only 72% nucleotide identity with the *khipu* units from the subclade comprising centromeric repeats, while it shares more than 85% identity with the *khipu* units from other major clades (data not shown). Consequently, it is possible that our probe missed the centromeric *khipu* units considering the stringency used. The fourth hypothesis is that the chromatin architecture in the centromeric region may make these sequences inaccessible to FISH.

## Conclusion

Satellite DNA is an enigmatic part of eukaryotic genomes. Subtelomeric satellite repeats have been reported in many eukaryotic chromosomes but their function remains largely unknown. A genome-wide analysis based on the complete genome sequence of the common bean genotype G19833 of the subtelomeric *khipu* satellite, revealed extensive sequence exchanges between non-homologous chromosomes in subtelomeric regions and also suggests sequence exchange between subtelomere and centromere.

## Conflict of Interest Statement

The authors declare that the research was conducted in the absence of any commercial or financial relationships that could be construed as a potential conflict of interest.

## Supplementary Material

The Supplementary Material for this article can be found online http://www.frontiersin.org/journal/10.3389/fpls.2013.00109/abstract

Figure S1 **Khipu sequence alignement**.

Figure S2 **WebLogo representation of the consensus sequence derived from the multiple alignment of the khipu units and sequence of one representative khipu unit from each major clade**.

Figure S3 **Phylogenetic tree of whole-genome khipu sequence**.

## Footnotes

^1^www.phytozome.org

^2^http://www.phytozome.net/

^3^http://hmmer.janelia.org

^4^http://asap.ahabs.wisc.edu/mauve/index.php

^5^http://weblogo.berkeley.edu

^6^http://www.phytozome.net/

## Appendix

**Table A1:** **Characteristics of the probes sed for FISH experiments**.

Probe	BAC clone	Contig	Genotype	Subclone	Length (bp)	Coordinates on BAC	Coordinates on contig	Notes and reference
*khipu*	Pva1-105k5	PvAO5A	Gl9833	TE0AAFA1Y119	6135	64475..70609	64475..70610	Chen et al. (2010)
	Pva1-68o6	PvA11B	G19833	TE0AAAA3YM09	5268	10430..15697	10430..15698	Innes et al. (2008)
	Pval-34g17	PvA11C	G19833	TE0AANA1YI09	7692	14329..22020	165163..172854	Innes et al. (2008)
	Pva1-119e5	PvA11E	G19833	TE0AAEA2YP22	5628	163980..169607	348510..354137	Innes et al. (2008)
	–	Pv410-kb	BAT93	1H04	2410	–	388326..390736	David et al. (2009)
