# Supplementary material for: The Subtelomeric khipu Satellite Repeat from Phaseolus vulgaris: Lessons Learned from the Genome Analysis of the Andean Genotype G19833
Source: Front Plant Sci. 2013 Oct 16;4:109. doi: 10.3389/fpls.2013.00109 (PMC3797529; doi:10.3389/fpls.2013.00109)
Supplement: Figure S1 — Khipu sequence alignement. [file 47451_Geffroy_DataSheet1.DOCX]

>Pv01Lk02080/1-539 Pv01Lk02080 undefined product 50594599:50595137 reverse

---CCGAAATGG---TTTTTTTTTCTG--AAATTTCGCGT-AAG-AA--T-------CT---CTACTG-AG-

TTTGGG-AC----------AAAATGCG-AC-AG-AT--TTCAGG--TC------------------------

-----------------------------------------AAAC-G--AATAAG---TATGCAC-------

---CCA-----GGAAAAAAA---ATTAAACAGTT-TC--------------------------ACA-CAC--

AAAC--G-AA-CCT------------TTC--T-TTC-------A-A--CC-C-G-GG-C---------A--G

TGATG-AAT-A--A-A-AA--A-TT-TA-TTGCG-AATCTTGT-GGGACGAG----TCT------GGA----

-------------GCTAAAATCTCAG-C----------------C-AAA-ACTCAATAGACACA---A----

------------------TGA--C----GAA-TGTCTGG----------T--A-AAAATTTC-ATA-ACA--

--AAATACAC-------AAGGA-GT-----AAGGG---GC-----AATA------AG-TCACAGA-CCGAGA

GTGAA-------------------------------------------------------------------

------------------------------------------------------------------------

--------------C--AAAATC--------------------------GG-----------------TTTT

C----------------------CGAAAACAAAA--C--GTCCTGGC-----TTTTCTTCCC----------

--------------CGTATC--TTCT-----------TTCTGTGA---------------------------

-------------------------TTT--GTTTAT-GGAC--GTG-CCT-CC-TTACCT--GGGTGC--AA

AC-----------AACAT-A----CTAAATT-ACTTGTG-T-------------GAATT-------------

----TTTTCA------------------------------GGACAAT-A-C-A-GA-CCC-GGA-----AA-

AAAATT--GCAGAAA--GTAGG------------GCAAAATTT--CACA-A-GTT-TTGC--TAGAG-----

---------GAT-AGCCT-------------------TGG-TTTGCACA----AAACTTCCGAAAAGTT-

>Pv01Lk02150/1-549 Pv01Lk02150 undefined product 51982942:51983490 reverse

--CCCGAAATAA-----TTATTTCCTG--AAATTCCACGT-AAG-AA--T-------CG---AGACTG-AT-

TTTCGGGAC----------AAAACGTG-AC-AA-AT--TTCACG--TC------------------------

-----------------------------------------AAAC-G--GATGAG---TATTCAC-------

---CTA-----GGAAAAAAA---TC-AAACAGTT-TC--------------------------ACA-CAC--

AAAC--G-AA-CTT------------TCC--T-TTC-------A-A--CC-C-T-GG-C---------A--G

TGATG-ATA-A--A-A-AA--A-AT-TA-TTGAA-AATCTTGT-GGGACGAA----TCT------GGG----

-------------GCTCAAATCTCAG-C----------------CAAAA--CTCAATACACACA---A----

------------------TGA--A----GAA-TGTCTGG----------T--A-AAGTTTTC-AGA-CCA--

--AAAGACCC-------AAGGA-AT-----AGGGC---GT-----AGTA------AG-CCCCAGA-CCGAGA

GTGAA-------------------------------------------------------------------

------------------------------------------------------------------------

--------------C--AAAATC--------------------------GG-----------------TTTT

C----------------------CGAAAACAAAA--T--GTCCTGGA-----TTTTCTTCCC----------

--------------TGTATC--TTCT-----------TTTTGTGA---------------------------

-------------------------ATTT-TTTTAT-GGAC--GTG-CGT-CC-TTAGGA--GGGTGC--AA

AC-----------AACAC-A----TGAAAGT-ACTTGTG-T-------------GATTTTCTTT-------G

TAATTTTTCA------------------------------AGGCAAA-T-A-G-GA-CCA-CGA-----AT-

AAAATT--GCAGAGA--GTATG------------GCGAAATTT--CTCA-A-ATT-TTGC--TAGAG-----

---------GAT-AGCCT-------------------TGCTTTTTCACA----AAATTTAAGAAAAAT--

>Pv01Lk02160/1-547 Pv01Lk02160 undefined product 51983494:51984040 reverse

-TCCCGAAATAA-----TTCTTTCCTG--AAATTCCACGT-AAG-AA--T-------CG---TGACTG-AA-

TTCGGG-AC----------AAAACGAG-AC-AA-AT--TTCACG--TC------------------------

-----------------------------------------AAAC-G--GATGAG---TATTCAC-------

---CTA-----GGAAAAAAA---TC-AAACAGTT-TC--------------------------ACA-CAC--

AAAC--G-AA-CCT------------TCA--T-TTC-------A-A--CC-C-T-GG-C---------A--G

TAATG-AT--A--A-A-AA--A-AT-TA-TTGAA-AATCTTGT-GGGACGAA----TCT------GGG----

-------------GCTCAAATCTCAG-C----------------CAAAA--CTCAATACACACA---A----

------------------TAA--C----GAA-TGTCTGG----------T--A-AAAATTTC-AGA-CCA--

--AAAGACCC-------AAAGA-AT-----AGGGC---GT-----AGTA------AG-ACCCAAA-CCGAGA

GTGAA-------------------------------------------------------------------

------------------------------------------------------------------------

--------------C--AAAATC--------------------------GA-----------------TTTT

T----------------------CGAAAACAAAA--T--GTCCTGGC-----TTTTCTTCCC----------

--------------TGTATC--TTCG-----------TTTTGTGA---------------------------

-------------------------ATT--TTTTAT-GGAC--GTG-CCT-CC-TTAGGA--GGGTGC--AA

AC-----------AACAG-A----TGAAAGT-ACTTGTG-T-------------GATTTTCTTT-------G

TAATTTTTCA------------------------------AGGCAAA-T-A-G-GA-CCA-CGA-----AT-

AAAATT--GCAGAGA--GTATG------------GCGAAATTT--CTCA-A-ATT-TTGC--TAGAG-----

---------GAT-AGCCT-------------------TGCTTTTTCACA----AAATTTAAGAAAAAT--

>Pv01Lk02180/1-552 Pv01Lk02180 undefined product 51986265:51986816 reverse

--CCCGAAATAA-----TTTTTTCCTG--AAATTCCACGT-AAG-AA--T-------CG---TGACTG-AT-

TTGGGG-AC----------TAAACGCG-AC-AA-AT--TTAACG--TC------------------------

-----------------------------------------AAAC-G--GATGAG---TATTCAC-------

---CTA-----GGAAATAAA---TC-AAACTGTT-TC--------------------------ACA-CAC--

AAAC--G-GA-CAT------------TCC--T-TTC-------A-A--CC-C-T-GG-C---------A--G

TGATG-ATA-A--A-T-TT--T-TT-TA-TTGAA-ATTCTTGT-GGGACGAA----TCTGAATCCGAG----

-------------GCTCCAATCTCAG-C----------------CAAAA--CTCAATACACACA---A----

------------------TAA--C----GAA-TGTCTGG----------T--A-AAAATTTC-AGA-CCG--

--AAAGACCT-------AAGGA-AT-----AGGGC---GT-----AGTA------AG-ACCCAGA-CCGAGA

ATAAA-------------------------------------------------------------------

------------------------------------------------------------------------

--------------C--AAAATC--------------------------GG-----------------TTTT

T----------------------CGAAAAC-AAA--T--GTCCTAGC-----TTTTCTTCCT----------

--------------TGTATC--TTCT-----------TTTTTTGA---------------------------

-------------------------ATT--TTTTAT-CGAT--GTG-TCT-CC-TTAGGA--GGGCGC--AA

AC-----------AACAC-A----TGAAAGT-ACTCGTG-T-------------GATTTTTTTT-------G

CAATTTTTCA------------------------------ACGCAAT-A-T-G-GA-GCC-CGA-----AT-

AAAATT--GCAGAGA--GTATG------------GCAAAATAG--CAAA-A-TTT-TTGC--TAGAG-----

---------GAT-AGCCT-------------------TGGTTTTTCACT----AAATTTAAGAAAAAT--

>Pv01Lk02190/1-548 Pv01Lk02190 undefined product 51986820:51987367 reverse

-TCCCGAAATAA-----TTCTTTCCTG--AAATTCCACGT-AAG-AA--T-------CG---TGACTG-AA-

TTCGGG-AC----------AAAACGCG-AC-AA-AT--TTCACG--TC------------------------

-----------------------------------------AAAC-G--GATGAG---TATTCAC-------

---CTA-----GGAAAAAAA---TC-AAACAGTT-TC--------------------------ACA-CAG--

TAAC--G-AA-CCT------------TCC--T-TTC-------A-A--CC-C-T-GA-T---------A--G

TGATG-ATA-A--A-A-AA--A-TT-TA-TTGAA-AATATTGT-GGGACGAA----TAT------GAG----

-------------GCTTAAATCTCAG-C----------------CAAAG--CTTAATACACACA---A----

------------------TTA--C----GAA-TGTCTGG----------T--A-AAAATTTC-AGA-CCA--

--AAAGACCC-------AAGGT-AT-----AGGGC---GT-----AGCA------AG-ACCCACA-CCGAGA

GTGAA-------------------------------------------------------------------

------------------------------------------------------------------------

--------------C--AAAACC--------------------------GG-----------------TTTT

T----------------------CGAAAACAAAA--T--GTCCTGTC-----TTTTCTTCCC----------

--------------TGTATC--TTCT-----------TTTTGTGA---------------------------

-------------------------ATT--TTTTAT-GGAC--GTG-CCT-CC-TTAGGA--CGGTGC--AA

AC-----------AACAC-A----TGGAAGT-ACTTGTG-T-------------GATTTCTTTT-------G

CAATTTTTCA------------------------------ACGCAAT-A-T-A-GA-TCC-CGA-----AT-

AAAATT--GCAGAGA--GTATG------------GCGAAATTT--CACA-A-ACT-TTAC--TAGAG-----

---------GAT-AGCCT-------------------TGGTTTTTCACA----AAATTTAACAAAAAT--

>Pv01Lk02200/1-541 Pv01Lk02200 undefined product 52051930:52052470 reverse

CTCCCGGAATAG-----TTTTTTCCTG--AAATTCCACCC-AAG-AA--T-------CT---CCACTG-AA-

TTTGCG-AC----------AAAACGCT-AC-AA-AT--TTCAGG--TC------------------------

-----------------------------------------AAAC-G--GATGAG---TATTCAC-------

---CCA-----CGGAAAAAA---TC-AAACAGTT-TC--------------------------AAG-CAC--

GAAC--G-AA-CCT------------TCC--T-TTC-------T-G--CC-C-T-GG-C---------A--G

TGACG-AAT-A--A-A-AA--A-TT-TA-TTGCC-AATCTTGT-GGGAAGAA----TCT------GGG----

-------------GCTCAGACCTCAG-C----------------CAAAA--CTCAACAGACACA---G----

------------------TGA--C----GAA-TGTCTGG----------T--A-AAAATTTC-AGA-CCA--

--AAATACCC-------AAGGA-GT-----AAGGC---GT-----AGTA------AG-TCCCAGA-CCGAGA

GTGAA-------------------------------------------------------------------

------------------------------------------------------------------------

--------------C--AAAACC--------------------------GG-----------------TTTT

C----------------------CGAAAACAAAA--C--GTCCTGGC-----TTTTCTTCCC----------

--------------CGTATC--TTCT-----------TTTTGTGA---------------------------

-------------------------TTC--GTTTGT-GGAG--GTG-CCT-CC-TTGCCT--GGGAAC--AA

AC-----------AACAT-A----CGGAAGT-GCTTGTA-T-------------GAATT-------------

---TTTTTCA------------------------------GCGCAAT-A-C-G-GA-CCC-AGA-----AT-

GAAACT--GCAGAAA--TTAGG------------CCAGAATTT--CACA-A-GTT-TCGG--TAGAG-----

---------GAT-AGCCT-------------------TGG-TTTGTACA----AAATTTCTGAAAAATTC

>Pv01Lk02210/1-528 Pv01Lk02210 undefined product 52190630:52191157 reverse

-TCCCAAAATAT-----TTTTTTCCTA--AAATTCCATGT-AAG-AA--T-------CT---GCTTTT-AA-

TTTTGG-AT----------AAAACGCG-AC-AA-AT--TTCAGG--TC------------------------

-----------------------------------------AAAC-G--GATGAG---TATTCAC-------

---CCA-----CGAAAAAAA---TA-AAAAAGTT-TC--------------------------ACA-CAC--

AAAC--A-AA-CCT------------TCC--T-TTC-------A-G--CT-C-T-GG-C---------A--C

TTATG-AAT-A--A-A-AA--A-TT-TA-TTGTC-AATCTTGT-GGGACGAA----TCT------GGG----

-------------GCTCAAATCTTTG-C----------------C-AAA-ACTCAGTAGACACA---G----

------------------TGA--C----GAA-CGTGTGG----------T--A--AAAATTTCAGA-CCA--

--AAATACCC-------AAGGA-GT-----AAGGC---GT-----AGGA------AG-TCCCATA-CTGAGA

GAGAA-------------------------------------------------------------------

------------------------------------------------------------------------

--------------C--AAAACT--------------------------GG-----------------TTTT

C----------------------TGAAAACAAAT--C--GTCCTGAC-----TTTTCTTTGC----------

--------------CGTATC--TTCT-----------TTTTCTGA---------------------------

-------------------------TTA--GTTTAT-GGAT--GTG-CCT-CC-T------------C--AA

TC-----------AATAT-A----TGAAAGT-ACTTGTG-T-------------GAA---------------

--TTTTTTCA------------------------------ATGCAAT-A-C-G-GA-CCC-AAA-----AT-

AAAATT--TCAGAAA--GTAGG------------GCTACATTT--TACA-A-GTT-TTGG--TAGAG-----

---------GAT-AGCTT-------------------TGG-TTTGCACA----AAATTTATGAAAATTT-

>Pv01Lk02220/1-525 Pv01Lk02220 undefined product 52191159:52191683 reverse

CTCCCGAAATAG-----CTTTTTCCTA--AAATTCCACAT-AAG-AA--T-------GT---ACACTG-AA-

TTTGGG-AC----------AAAACACG-AC-AA-AT--TTCAGG--TG------------------------

-----------------------------------------AATT-G--GATAAG---TATTCAC-------

---CCT------AAAAAAAA---TC-AAACAGTT-TC--------------------------ACA-CAT--

AAAC--G-AA-CCT------------TCC--T-TTC-------A-G--CC-C-T-AA-C---------A--A

TGATG-AAT-A--A-A-AA--A-TT-TA-TTACC-AATCTTGT-GGGGAGAA----ATT------GGG----

-------------GCTCAATTCTAAT-C----------------C-AAA-AATCAGTAGACATA---G----

------------------TGA--C----CAA-CGTGTGG----------T-AA-------------------

----ATACCC-------AAGGA-GT-----AAGGC---GT-----AGTA------AG-TCCCAAA-CCGAGA

GTGAA-------------------------------------------------------------------

------------------------------------------------------------------------

--------------C--AAAATT--------------------------GG-----------------TTTT

C----------------------CGAAAACAAAA--C--GTCCTGGC-----TTTTCTTCGT----------

--------------CGTATC--TTCT-----------TTTTCTGA---------------------------

-------------------------TTT--GTTTTT-GGAA--GTT-CCT-CC-TTATTC--GATTGA--AA

AC-----------AACAT-A----TGAAAGT-ACTTGTG-T-------------GAA---------------

--TTTTTTCA------------------------------GCGCAAT-A-C-G-GA-TCC-ATA-----AT-

AAAATT--TCAGAATTTGTAGG------------GCGAAATTT--CACA-A-GTT-TTGG--TAGAA-----

---------GAT-AACCT-------------------TGG-TTTGTACA----AAATTTATGAAAAATT-

>Pv01Lk02230/1-533 Pv01Lk02230 undefined product 52191684:52192216 reverse

CTCCCAATATAG-----TTATTTCCTG--AAATTCCACGT-AAG-AA--T-------AT---CCATTG-AA-

TTTGG--AC----------AAAACGCG-AC-AA-AT--TTCAGG--TC------------------------

-----------------------------------------AAAC-A--GATGTG---TATTGAC-------

---CCT-----CGATAAAAA---TC-AAACAGTT-TC--------------------------ACA-CAC--

AAAA--A-AA-CCT------------TCC--T-TTC-------A-G--CC-C-T-GG-C---------A--C

TGATG-AAT-A--A-A-AA--A-TT-TA-TTGTC-AATCTTGT-GGGACGAA----TCT------GGG----

-------------GGTCAATTCTAAG-C----------------C-AAA-ACTCAGTAGACACA---G----

------------------TGA--C----AAA-CGTCTGG----------T-AA--AAATTTC-ATT-CCA--

--AAATACCC-------AAAGA-GT-----AAGGC---AT-----AGGA------AG-TCTCAGA-CGGAGA

GTGGG-------------------------------------------------------------------

------------------------------------------------------------------------

--------------C--AAAATT--------------------------GG-----------------TTTT

C----------------------CGAAAACAAAA--C--GTCCTGGC-----TTTTCTTCGT----------

--------------CGTATC--TTCT-----------TTTTCTGA---------------------------

-------------------------TTT------AT-GGTC--GTT-CCT-CC-TTACCC--CGGTGC--AA

AC-----------AACAT-A----TGAAAAT-AATTGTG-T-------------GAA---------------

--TTTTTTCA------------------------------GCGCAAT-A-C-G-GA-CCC-AAA-----AT-

AAAATT--TTAGAAA--GTAGG------------GTGAAATTT--CACA-T-GTT-T-GG--TAGAG-----

---------GAT-AACCT-------------------TGG-TTTGCACA----AAATTTATGAAAAGTT-

>Pv01Lk02240/1-540 Pv01Lk02240 undefined product 52192217:52192756 reverse

CTCCCGAAATAG-----CTTTTTCGTG--AAATTACGCAT-AAG-AA--T-------CT---CCACTG-AA-

ATTGGG-AA----------AAAACGCG-AC-AA-AT--TTCAGG--TC------------------------

-----------------------------------------CAAC-A--GATGTG---TATTCAC-------

---CCT-----CGAAAAAAA---TA-AAACAGTT-TC--------------------------ACA-CAC--

TAAC--G-AA-CCT------------TCC--T-TTC-------A-G--CC-C-T-GT-C---------A--C

TGATG-AAT-A--A-A-AA--A-TT-TA-TTGTC-AATCTTGT-GAGACGAA----TCT------GGG----

-------------GCTCAATTCTAAG-C----------------C-AAA-ACTCAGTAGGCACA---A----

------------------TGA--C----AAA-CGTTTGG----------T-AA--AAATTTC-AGA-CCA--

--AAATACCC-------AAGGA-GT-----AAGGC---GT-----AGGA------AG-TCCAAGA-CCGAGA

GTGAA-------------------------------------------------------------------

------------------------------------------------------------------------

--------------C--AAAACT--------------------------GG-----------------TTTT

C----------------------CAAAAACAAAA--C--ATCCTGGCT----TTTCTTTTGC----------

--------------CGTATC--TTTT-----------TTTTCTAA---------------------------

-------------------------GTT--GTTTTT-GGTT--TTG-CCT-CC-TTACCT--GGGTGT--AA

AC-----------AACAT-A----TGAAAGT-ACTTGTG-T-------------GAA---------------

--TTTTTTCT------------------------------GCGCAAT-A-C-A-AA-CCC-AGA-----AT-

AAAATT--TCAAAAA--GTAGG------------GCGAAATTT--CACA-A-GTT-TTGG--TAGAG-----

---------GAT-AACCT-------------------TGG-TTTGCACA----AAATTTATGAAAAAAT-

>Pv01Lk02250/1-527 Pv01Lk02250 undefined product 52192756:52193282 reverse

CTCCTGGAATAG-----TTTTTTCCTG--AAATTCCACCC-AAG-AA--T-------CT---CCACTG-AA-

TTTGGG-AC----------AAAACGCG-AC-AA-AT--TTCAGG--TC------------------------

-----------------------------------------AAAG-G--GATGAG---TATTCAT-------

---CCA-----CGAAAAAAT---AA-AAACAGTT-TC--------------------------ACA-CAC--

AAAC--A-AA-CCT------------TCC--T-TTC-------A-G--CT-C-T-GG-C---------A--C

TGATG-AAT-A--A-A-AA--A-TT-TA-TTTCC-AATCTTGT-GGGACGAA----TCT------AGG----

-------------GCTCAAATTTAAG-C----------------C-AAA-ATTCAGTAGACACA---G----

------------------TGA--C----AAA-CGTGTGG----------T-AA--AAATTTC-ATA-CCA--

--AAATACCC-------AAGGA-AT-----AAGGC---GT-----AGTA------AG-TCCCAGA-CCGAGA

GTGAA-------------------------------------------------------------------

------------------------------------------------------------------------

--------------C--AAAATT--------------------------TC-----------------TTTT

C----------------------CAAAAACAAAA--C--GTCCTCGT-----TTTTCTTCGT----------

--------------CGTATC--TTCT-----------TTTTCTTA---------------------------

-------------------------TTT--GTTTAT-GGAC--GTG-CCT-CC-T------------C--AA

AC-----------AATAT-A----TGAAAGT-ACTTTAG-T-------------GAA---------------

---TTTTTTA------------------------------GCACAAT-A-C-G-GA-CCC-AGA-----AT-

AAATTT---AAGAAA--GTAGG------------GCGAAATGT--CACA-T-GTT-TTGG--TAGAG-----

---------GAT-AACCT-------------------AGG-TTTGCACA----AAT-TTATGAAAAATTC

>Pv01Lk02260/1-525 Pv01Lk02260 undefined product 52194352:52194876 reverse

CTCCCGAAATAG-----TTTTTTCTAG--AATTTCCACCC-AAG-AA--T-------AT---CCACTA-AA-

TTTGGG-AC----------AAAATACG-AC-AA-AT--TTCAGG--TC------------------------

-----------------------------------------AAAC-G--GAGGAG---TATTCAC-------

---CCA-----CAAAAAAAAA--TC-AAACAGTC-TC--------------------------ACA-CAC--

AAAA--T-AA-CCT------------TCC--T-TTC-------A-G--CC-T-A-GG-C---------A--G

TGACG-AAT-A--A-A-AA--A-TT-TA-TTGCT-AATCTTGT-GGGACGAA----TCT------GGG----

-------------GCTCAAACCTCAG-C----------------CAAAA--CTCAATAGACATA---G----

------------------TGA--A----GAA-TGTCTGG----------T--A-AAAATTTC-ATA-CCA--

--AAATACCC-------AAGGA-GT-----AAGGC---GT-----AGTA------AG-TCCCAGA-CCGAGA

GTGAA-------------------------------------------------------------------

------------------------------------------------------------------------

--------------C--AAAACC--------------------------GG-----------------TTTT

C----------------------CGAAAACAAAA--C--GTCCAAGC-----TTTTCTTCCC----------

--------------TGTATC--TTCT-----------TTTTGTGA---------------------------

-------------------------TTC--GTTTAT-GGAC--GTG-CCT-CC-TTGCCT--GGGTGC--AA

AC-----------AACAT-A----CGAAAGT-GCTTGTG-T-------------GAATTT------------

----TTTTCT------------------------------GCGCAAT-A-C-G-GA-CCC-AGG-----AT-

GAAATT--GCAGAAA--TTAGG------------CCGGAATTT--CACA-A-GTT-TCGG------------

-----------------------------------------TTTGCACA----AAATATCTGAAAAGTT-

>Pv01Lk02270/1-540 Pv01Lk02270 undefined product 52194876:52195415 reverse

--CCCGTAATAG----TTTTTTTCTTG--AAATTCCACCC-AAG-AA--T-------CT---GCACTG-AA-

TTTGGG-AC----------ATAACGCG-AC-AA-AT--TTCAGG--TC------------------------

-----------------------------------------AAAC-G--GATGAG---TATTCAT-------

---CCA-----CTAAAAAAA---TA-AAACACTT-TC--------------------------ACA-AAA--

AAAC--G-AA-CCT------------TCC--T-TTC-------A-G--CC-C-T-AG-C---------A--G

TGACG-AAT-A--A-A-AA--A-TT-TA-TTTCC-AATCTTGT-GGGACGAA----TCT------GGG----

-------------GCTCAAACCTCAG-C----------------CAAAA--CTCAATAGCCACA---G----

------------------TGA--C----GAA-TATCTGA----------T--A-AAAATTTC-AGA-CCA--

--AAATACCC-------AAGGA-GT-----AAGGC---GT-----AGTA------AG-TCCCAGA-CCGAGA

GTGAA-------------------------------------------------------------------

------------------------------------------------------------------------

--------------A--AAAACT--------------------------GG-----------------TTTT

C----------------------CGAAAACAAAA--C--GTCATGGC-----TTTTCTTCCC----------

--------------CGTATC--TTCT-----------TTTTGTGA---------------------------

-------------------------TTC--GTTTAT-GGAC--GTG-CCT-CC-TTGCCT--GGGTGC--AA

AC-----------AACAT-A----CAAAAGT-GTTTGTG-T-------------GGATTT------------

----TTTTCA------------------------------GCGCAAT-A-C-G-GA-CCC-ATA-----AT-

GAAATT--ACAGACA--TTAGG------------CCGAAGTTT--CACA-A-GTT-TCGG--TAGGG-----

---------GAT-AGCTT-------------------TGT-TTTGCACA----AAATTTATGAAAAATTC

>Pv01Lk02290/1-541 Pv01Lk02290 undefined product 52195861:52196401 reverse

CTCCCGGAATAG-----TTTTTTCTTG--AAATTCCACCC-AAG-AA--T-------CT---CCACTG-AA-

TTTGGG-AT----------AAAACGCG-AC-AA-AT--TTCAGG--TC------------------------

-----------------------------------------AAAC-G--GATGAG---TATTCAT-------

---CCA-----CGAAAAAAA---TC-AAACAGTC-TC--------------------------ACA-CAC--

AAAC--G-AA-CCT------------TCC--T-TTC-------A-T--CC-T-T-GA-C---------A--G

TGACG-AAT-A--A-A-AA--A-TT-TA-TTGCT-AATCTTGT-GGGACGAA----TCT------GGG----

-------------GCTCAAACATCAC-C----------------CAAAA--CTCAATAGACACC---G----

------------------TGA--C----GAA-TATCTGA----------T--A-AAAATTTC-ATA-CCA--

--AAATACCC-------AAGGA-GT-----AAGGC---GT-----AGTA------AG-TCCCACA-CCGAGA

GTGAA-------------------------------------------------------------------

------------------------------------------------------------------------

--------------A--AAAAAT--------------------------GG-----------------TTTT

C----------------------CGAAAACAAAA--C--GTCATGGC-----TTTTCTTCCC----------

--------------CGTATC--TTCT-----------TTTTGTGA---------------------------

-------------------------TTC--GTTGAT-GGAC--AGG-GCT-CC-CTGCCT--GGGTGC--AA

AC-----------AACAT-A----CGAGAGT-GCTTGTG-T-------------GAAATT------------

----TTTGCA------------------------------GCGCAAT-A-C-G-GA-CCC-AGA-----AT-

GAAATT--GCAGAAA--TCAGG------------CCGAAATTT--CACA-A-GTT-TTGG--TAGAT-----

---------GAT-TGCCT-------------------CGG-TTTGCACA----AAATTTCTGAAAAATTC

>Pv01Lk02300/1-534 Pv01Lk02300 undefined product 52201219:52201752 reverse

-TCCCGAAATAG----TTTTTTTCCTG--AAATTTCACGT-AAG-GA--T-------CT---CCATTG-AA-

TTTGGG-AC----------AAAACACG-AC-AT-AT--TCCAGG--TC------------------------

-----------------------------------------AAAG-G--GATGAG---TATTAAC-------

---CCA-----CGAAAAAAA---TC-AAACAGTT-TC--------------------------ACA-CCC--

AAAT--G-AA-CCT------------TCC--T-TTC-------A-G--CC-C-T-GA-C---------A--G

TGATG-AAT-A--A-A-AA--A-TT-TA-TTAAC-AATCTTGT-GGGACGAA----TTT------GGG----

-------------TCTCAAATCTCAG-C----------------C-AAA-AGTCGGTAGACACG---G----

------------------TGA--C----GAA-CTTCTGG----------A--A-AAAATTTC-AGA-CAG--

--AAATACCC-------AAGGA-GT-----AAGGC---GT-----AGTA------AG-TCCCAGA-CCAAGA

GTGAA-------------------------------------------------------------------

------------------------------------------------------------------------

--------------A--AAACT---------------------------GG-----------------TTTT

C----------------------TGAAAACAAAA--C--ATTATGGT-----TTTTCTTTCC----------

--------------CGTATC--TTCT-----------TTTTGTGA---------------------------

-------------------------TTT--TTTTAT-GGAT--GTG-CCT-CC-TTACCT--GGGTGC--AA

AC-----------ATAAT-A----AGAAAGT-ACTTCTA-T-------------GAA---------------

--TTTTTTCA------------------------------GCGCAAT-A-C-G-GA-TCC-AGA-----ATA

GAA-TT--TCAGAAA--GTAGG------------GCGAAATTT--CACA-A-GTT-TTGG--TAGAG-----

---------GAT-AACCT-------------------TGG-TTTGCACA----AAATTTCTGAAA-----

>Pv01Sk00010/1-529 Pv01Sk00010 undefined product 97909:98437 forward

-TTCCGGAATGG-----TTTTTTACTG--AAATTCCACCG-AAG-AA--T-------CT---CCACTG-AA-

TTTGGG-AC----------AAAACGGG-AC-AA-AT--TTCAGG--TC------------------------

-----------------------------------------AAAC-G--GATGAG---TATTCGC-------

---CCA-----CGAAAAAAA---TC-AAACAGTT-TC--------------------------ACA-CAC--

AGAC--G-AA-CCT------------TCC--T-TTC-------A-G--CC-C-T-GG-C---------A--G

TGGAC-AAT-A----A-AA--A-TT-TA-TTGCC-AATCTTGT-GAGACGAA----TCT------GGG----

-------------GCTCAAACCTCAG-C----------------CAAAA--CTCAATAGACACA---G----

------------------TGA--C----GAG-TGTCTGG----------T--A-AAACTTTC-AGA-CAA--

--AAATACCC-------AAGGA-GT-----AAGGC---GC-----AGTA------AG-TCGCAGA-CCGAGA

GTGAA-------------------------------------------------------------------

------------------------------------------------------------------------

--------------C--AAAACC--------------------------GC-----------------TTTT

C----------------------CGAAAACAACA--C--GTCCTGGG-----TTTTCTTCCC----------

--------------CGTATC--TTCT-----------TTTTGTGA---------------------------

-------------------------TTC--GTTTAT-GGAC--GTT-CCT-CC-TTGCCT--GGGTGG--AG

AC-----------AACAT-A----CCAAAGT-GCTTGTG-T-------------GAATT-------------

---TTTTTCA------------------------------GCGCAGT-A-C-G-GA-CCC-AGA-----AT-

GAAATT--GCAGAAA--TTAGG------------CCGGAATTT--CACA-A-GTT-TCGG--TAGAG-----

---------GAT-AGCCT-------------------TGG-TTTTTCCA--------------AAAATTC

>Pv01Sk00020/1-529 Pv01Sk00020 undefined product 98440:98968 forward

---CCCGAATGG-----TTTTTTCCTG--AAATTCCACCC-AGA-AT--C-------TC---CAAGGA-A--

-TTGGG-AT----------TAAACGGG-AC-AA-AT--TTCAGG--TC------------------------

-----------------------------------------AAAC-G--GATGAG---TATTCGC-------

---CCA-----CGAAAAAAA---TG-AAACAGTT-TC--------------------------ACC-CAC--

AGAA--G-AA-CTT------------CCC--T-TTC-------A-G--CC-T-T-GG-C---------A--G

TGACG-AAT-A--A-A-AT--A-TT-TA-TTCCC-AATCTTGT-GGGAGGAA----TCT------GGG----

-------------GCTCAAACGTCAG-C----------------CAAAA--CTCAATAGACACA---G----

------------------TGA--C----GAG-TGTCTGG----------T--A-AAAATTTC-AGA-CCA--

--AAATACCC-------AGGGA-GT-----ATGGC---GC-----AGTA------AG-TCCCAGA-CCGAGA

GTAAA-------------------------------------------------------------------

------------------------------------------------------------------------

--------------C--AAAACC--------------------------GG-----------------TTTT

C----------------------CGAAAACAAAG--C--GTCCTGGC-----TTTTCTTCCC----------

--------------CGTATC--TTCT-----------TTTTGTGG---------------------------

-------------------------TTC--GTTTAT-GGAC--GTT-CCT-CC-TTGCCT--GGGTGC--AA

AT-----------AACAT-A----CGAAAGT-GCTCGTG-T-------------GAATT-------------

---TTTTTTA------------------------------GCGCAGT-A-C-G-GACCCC-AGA-----AT-

GAAATT--GCAGAAA--TTAGG------------CCGCAATTT--CACA-A-GTT-TCGT--CCG-------

---------------CCT-------------------TGG-TTTTACAA----AA-TTTCTGAAAAATTC

>Pv01Sk00030/1-540 Pv01Sk00030 undefined product 98969:99508 forward

-TTCCGGAATGG-----TTTTCTCCTG--AAATTCCACCC-AAG-AA--T-------CT---CCACTG-AA-

TTTGGG-AC----------AAAACGGG-AC-AA-AT--TTCAGG--TC------------------------

-----------------------------------------AAAC-G--GATGAG---TATTCTC-------

---CCA-----CGAAAAAAA---TC-AAACACTT-TC--------------------------ACA-CAC--

AGAC--G-AA-CCC------------TCC--T-TTC-------A-G--CG-C-T-GG-C---------A--G

TGACG-AAT-A--A-A-AA--A-TT-TA-TTGCC-AATCTTGT-GGGACGAA----TCT------GGG----

-------------GCTCAAACCTCAG-C----------------CAAAA--CTCAATAGACACA---G----

------------------TGA--C----GAG-TGTCTGG----------T--A-AAAATTTC-AGA-CCA--

--AAATACCC-------AGGGA-GT-----AAGGC---GC-----AGTA------AG-TCCCAGA-CCGAGA

GTGAA-------------------------------------------------------------------

------------------------------------------------------------------------

--------------C--AAAACC--------------------------GA-----------------TTTT

C----------------------CGAAAACAAAA--C--GTCCTGGC-----TTTTCTTCCC----------

--------------CGTATC--TTCT-----------TTCTGTGG---------------------------

-------------------------TTT--GTTTAT-GGAC--GTT-CCT-CC-TTGCCT--AGGTGC--AA

AC-----------AACAC-A----CGAAAGT-GCTTGTG-T-------------GAATT-------------

---TTTTTCA------------------------------GCGCAGT-A-C-G-AA-CCC-AGA-----AT-

GAAGTT--GCAGAAA--TTAGG------------CCGGAATTT--CACA-A-GTT-TCGG--TAGAG-----

---------GAT-AGCCT-------------------TGG-TTTTTACA----AAATTTCTGAGAAATTC

>Pv01Sk00040/1-539 Pv01Sk00040 undefined product 103278:103816 forward

-TTCCGGAATGG-----TTTTTTCCTG--AAATTCCATCC-AAG-AA--T-------CT---CCACTG-AA-

TTTGGG-AC----------AAAACGGG-AC-AA-AT--TTCAGG--TC------------------------

-----------------------------------------AAAC-G--GATGAG---TATTCAC-------

---CCA-----CGAAAAAAA---TC-AAACAGTT-TC--------------------------ACA-CAC--

AGAC--G-AA-CCT------------TCC--T-TTC-------A-G--CC-C-T-GG-C---------A--G

TGGCG-ATT-A--A-A-AA--A-TT-TA-TTGCC-AATCTTGT-GGGACGAA----TCT------GGG----

-------------GCTCAAACCTCAG-C----------------CAAAA--CTCAATAGACACA---G----

------------------TGA--C----GAG-TGTCTGG----------T--A-AAAATTTC-AGA-CCA--

--AAATACCC-------AAGGA-GT-----AAGGC---GC-----AGTA------AG-TCCCAGA-CCGAAA

GTGAA-------------------------------------------------------------------

------------------------------------------------------------------------

--------------C--AAAACC--------------------------GG-----------------TTTT

C----------------------CGAAAACAAAA--C--GTCCTGCC-----TTTTCCTCCC----------

--------------CGTATC--TTCT-----------TTTTGTGA---------------------------

-------------------------TTC--GTTTAT-GGAC--GTT-CCT-CC-TTGCCT--GGGTGC--AA

AC-----------AACAC-A----CGAAAGT-GCTTGTG-T-------------GAATT-------------

---TTTTTCA------------------------------GCGCAGT-A-C-G-GA-CCC-AGA-----AT-

GAAGTT--GCAGAAA--TTACG------------CTGGAATTT--CACA-A-GTT-TCGG--TAGAG-----

---------GAT-AGCCT-------------------TGG-TTTTTAAA----AAATTTCTGAGAAACT-

>Pv01Sk00050/1-538 Pv01Sk00050 undefined product 104360:104897 forward

-TTCCAGAATGG-----TTTTTTCCTG--AAATTCCACCC-AAG-AA--T-------CT---CCACTG-AA-

TTTGGG-AC----------AAAACGGG-AC-AA-AC--TTCGGG--TC------------------------

-----------------------------------------AAAC-G--GATGAG---CATTCAC-------

---CCA-----CGAAAAAAA---TC-AAACAGTT-TC--------------------------ACA-CAC--

AGAC--G-AA-CCT------------TCC--T-TTC-------A-G--CC-C-T-GG-C---------A--G

TGGCG-ATT-A--A-A-AA--A-TT-TA-TTGCC-AATCTTGT-GGGACGAA----TCT------GGG----

-------------GCTCAAACCTCAG-C----------------CAAAA--CTCAATAGACACA---G----

------------------TGA--C----GAG-TGTCTGG----------T--A-AAATTTTC-AGA-CCA--

--AAATACCC-------AAGGA-GT-----AAGGC---GC-----AGTA------AG-TCCCAGA-CCGAGA

GTGAA-------------------------------------------------------------------

------------------------------------------------------------------------

--------------C--AAAACC--------------------------GG-----------------TTTT

C----------------------CGAAAACAAAA--C--GTCCTGGC-----TATTCTTCCC----------

--------------CGTATC--TTCT-----------TTTTGTGA---------------------------

-------------------------TTC--GTTTAT-GGTC--GTT-CCT-CC-TTGCCT--GGGTGC--AA

AC-----------AACAT-A----CGAAAGT-GCTTGTG-T-------------GAATT-------------

---TTTTTCA------------------------------GCGCAGT-A-C-G-GA-CCC-AGA-----AT-

TAAATT--GCATAAA--TTAGG------------CCGGAATTT--CACA-A-GTT-TCGG--TAGAG-----

---------GAT-AGCTT-------------------TGG-GTTTTGCA----AAATTTCTAAAAAAA--

>Pv01Sk00060/1-543 Pv01Sk00060 undefined product 108149:108691 forward

CTCCAGGAATAG-----TTTTTTCCTG--AAATTCCACCC-AAG-AA--T-------CT---ACACTG-AA-

TTTGGG-AC----------AAAACGCG-AC-AA-AT--CTCAGG--TC------------------------

-----------------------------------------AAAC-G--GATGAG---TATTCAC-------

---CCA-----CGAAAAAAA---TC-AAACAGTT-TC--------------------------AAA-CAC--

AAAC--A-AA-CCT------------TCC--T-CTC-------A-G--TC-C-T-GG-C---------A--G

TGACG-AAT-A--A-A-AA--A-TT-TA-TTGCC-AATCCTTT-GGGACGAA----TCT------GGG----

-------------GCTCAAAACTCAA-C----------------CAAAA--CTCAATAGACACC---G----

------------------TGA--C----GAA-TGCCTGG----------T--A-AAAATTTC-AGA-CCA--

--AAATACCC-------AAGGA-GT-----AAGGC---GT-----AGTA------AG-TCCCAGA-CCGGGA

GTGAA-------------------------------------------------------------------

------------------------------------------------------------------------

--------------C--AAAACC--------------------------GG-----------------TTTT

C----------------------CGAAAACAAAA--C--GTCCTGGC-----TTCTCTTCCC----------

--------------CGTATC--TTCC-----------TTTTGTGA---------------------------

-------------------------TTC--GTTTAT-GGAC--GTG-CCT-CC-TTTCTT--GGGTGC--AA

AC-----------AACAT-T----CGAAAGT-GCTTTTG-T-------------GAATTT------------

---TTTTTCA------------------------------GCGCAAT-A-C-A-AG-CCC-AGA-----AT-

GAAATT--GCATAAA--TTAGG------------CCCGAATTT--CACA-A-GTT-CCGA--TAGAG-----

---------GAT-AGCCT-------------------GTGTTTTGCACA----AAATTTCTGAAAAATTC

>Pv01Sk00070/1-541 Pv01Sk00070 undefined product 108691:109231 forward

CTCCCGGAATAG-----TTTTTTCCTG--AAATTCCACCT-AAA-AA--T-------CT---CCACTG-AA-

TTTGGG-AC----------AAAACGCG-AC-AA-AT--CTCAGG--TC------------------------

-----------------------------------------AAAG-G--GATGAG---TATTCAC-------

---CCA-----CGAAAAAAA---TC-AAACAGTT-TC--------------------------ACA-CAC--

AAA---G-AA-CCT------------TCC--T-CTC-------A-G--CC-C-T-GG-C---------A--G

TGACG-AAT-A--A-A-AT--A-TT-TA-TTGTC-AATCCTGT-GGGACGAA----TCT------GGG----

-------------GCTCAAACCTCAG-C----------------CAAAA--CTCAATAGACACA---A----

------------------TGA--C----GAA-TGTCTGG----------T--A-AAAATTTT-CGG-CCA--

--AAATACCC-------AAGGA-GT-----AAGGC---GT-----AGTA------AG-TCCCAGA-CCGGGA

GTGAA-------------------------------------------------------------------

------------------------------------------------------------------------

--------------C--AAAACC--------------------------GG-----------------TTTT

C----------------------CGAAAACAAAA--C--GTCCTGGC-----TTCTCTTCCC----------

--------------CGTATC--TTCC-----------TTTTGTGA---------------------------

-------------------------TTC--GTTTAT-GGAC--GTG-CCT-CC-TTTCTT--GGGTGC--AA

AC-----------AACAT-A----CGAAAGT-GCTTGTG-T-------------GAATTT------------

----TTTTAA------------------------------GCGCAAT-A-C-G-GA-CCC-AGA-----AT-

GAAATT--GCATAAA--TTAGG------------CCGTAATTT--CACA-A-GTT-TCGG--TAGAG-----

---------GAT-AGCCT-------------------TGGTTTTGCACA----GAATTTTTGAAAAATTC

>Pv01Sk00080/1-542 Pv01Sk00080 undefined product 110314:110855 forward

CTCCCGGAATAG-----TTTTTTCCTG--AAATTCCACCA-ATA-AA--A-------CT---CCACTG-AA-

TTTGGG-AC----------ACAACGCG-AC-AA-AT--CTCAGT--TC------------------------

-----------------------------------------AAAC-A--GATGAG---TATTCAC-------

---CCA-----CGAAAAAAAA--TC-AAACAGTT-TC--------------------------ACA-CAC--

AAA---G-AA-CCT------------TCC--T-CTA-------A-G--CC-C-T-GG-C---------A--G

TGACG-AAT-A--A-A-AT--A-TT-TA-TTGCC-AATCCTGT-GGGACGAA----TCT------GGG----

-------------GCTCAAACCTCAG-C----------------CAAAA--CTCAATAGACACA---G----

------------------TGA--C----CAA-TGTCTGG----------T--A-AAAATTTC-AGT-CCA--

--AAATACCC-------ATGGA-GT-----AAGGC---GC-----AGTA------AG-TCCCAGA-CCGAGA

GTGAC-------------------------------------------------------------------

------------------------------------------------------------------------

--------------C--AAAACC--------------------------GG-----------------TTTT

C----------------------CGAAAACAAAA--C--ATCCTGGC-----TTTTCTTCCC----------

--------------CGTATC--TTCC-----------TTTTGTGA---------------------------

-------------------------TTC--GTTTAT-GACC--GTG-CAT-CA-TTGCGT--GGGTGC--AA

AC-----------AACAT-A----CGAAAGT-GCTTGTG-A-------------GAATTT------------

----TTTTCA------------------------------GCGAATT-A-C-G-GA-CCC-AGA-----GT-

GAAATT--GCAGAAA--TTTGA------------CCGGAATTT--CACA-A-GTT-CCGG--TAGAG-----

---------GAT-AGCCT-------------------TGGTTTTGCACA----AAATTTCTGAAAAATTC

>Pv01Sk00090/1-542 Pv01Sk00090 undefined product 110855:111396 forward

CTCCGGGAATAG-----TTTTTTCCTG--AAATTCCACCC-AAA-AA--T-------CT---CCACTG-AA-

TTTGGG-AC----------AAAACACG-AC-AA-AT--CTCAGG--TC------------------------

-----------------------------------------AAAC-G--GATGAG---TATTCAC-------

---CCA-----CGAAAAAAA---TC-AAACAGTT-TC--------------------------ACA-CAC--

AAAT--G-AA-CCT------------TCC--T-CTC-------A-G--CC-C-T-GG-C---------A--G

TGACG-AAT-A--A-A-AT--A-TT-TA-TTGCC-AATCCTGT-GGGACGAA----TCT------GGG----

-------------GCTCAAACCTCAG-C----------------CAAAA--CTCAATAGACACA---G----

------------------TGA--C----GAA-TGTCTGG----------T----AAAATTTC-AGA-CCA--

--AAATACCC-------AAGGA-GT-----AAGGC---GT-----AGTA------AG-TCCCAGA-CCGGGA

GTTAA-------------------------------------------------------------------

------------------------------------------------------------------------

--------------C--AAAACC--------------------------GG-----------------TTTT

C----------------------CGAAAACAAAA--C--ATCCTGGC-----TTCTCTTCCC----------

--------------CGTATC--TTCC-----------TTTTGTGA---------------------------

-------------------------TTC--TTTTAT-GGAC--GTG-CCT-CC-TTTCGT--GGGTGC--AA

AC-----------AACAT-T----CGAAAGT-GCTTTTT-T-------------GAATTT------------

---TTTTTCA------------------------------GCGCAAT-A-C-A-AG-CCC-AGA-----AT-

GAAATT--GCATAAA--TTAGG------------CCCGAATTT--CACA-A-GTT-CCGG--TAGAG-----

---------GAT-AGCCT-------------------GTGTTTTGCACA----AAATTTCTAAAACATTC

>Pv01Sk00100/1-540 Pv01Sk00100 undefined product 111396:111935 forward

CTCCGGGAATAG-----TTTTTTCCTG--AAATTCCACCC-AAG-AA--T-------CT---CCACTA-AA-

TTT-GG-AC----------AAAACACA-AC-AA-AT--TTCAGG--TC------------------------

-----------------------------------------AAAC-G--GATGAG---TATTCAC-------

---CCA-----CGAAAAAA----TC-AAACAGTT-TC--------------------------ACA-CAC--

AAAC--G-AA-CCT------------TCC--T-CTC-------A-G--CC-C-G-GG-C---------A--G

TGACG-AAT-A--A-A-AT--A-TT-TA-TTGCA-AATCCTGT-GCGACGAG----TCT------GGG----

-------------GCTCAAACCTCAG-C----------------CAAAA--CTCAATAGACACA---G----

------------------TGA--C----GAA-TGCATGG----------T--A-AAAATTTC-AGA-CCA--

--AAATACCC-------AAGGA-GT-----AAGGC---GT-----AGTA------AG-TTCCAGA-CCGAGA

GTAAA-------------------------------------------------------------------

------------------------------------------------------------------------

--------------C--AAAACT--------------------------AG-----------------TTTT

C----------------------CGAAAACAAAA--C--GTCCTGGC-----TTCTCTTCCC----------

--------------CGTATC--TTCC-----------TTTTGTGA---------------------------

-------------------------TTC--GTTTAT-GGAC--GTG-CCT-CC-TTTCTT--GGGTGC--AA

AC-----------AACAT-T----CGAAAGT-ACTTTTG-T-------------GAATTT------------

----TTTTTA------------------------------GCGCAAT-A-C-A-AG-CCC-AGA-----AT-

GAAATT--GCATAAA--TTAGG------------CCCGAATTT--CACA-A-GTT-CCAG--TAGAG-----

---------GAT-AGCCT-------------------TAGTTTTGCACA----AAATTTCTGAAAAATTC

>Pv01Sk00110/1-539 Pv01Sk00110 undefined product 122846:123384 forward

CTCCCGGGATAG-----TTTTTTCCTG--AAATTCCACCC-AAG-AA--T-------CT---CCACTG-AA-

TTTGGG-AC----------AAAACGCG-AC-AA-AT--TTCAGG--TC------------------------

-----------------------------------------AAAC-G--GATGAG---TATTCAC-------

---CCA-----CGAAAAAAA---TC-AAACAGTT-TC--------------------------ACA-CAC--

AAAC--G-AA-CCT------------TCC--T-CTC-------A-G--CC-C-T-GG-C---------A--G

TGACG-AAT-A--A-A-TT--A-TT-TA-TTGCC-AATCCTGT-TGGACGAA----TCT------GTG----

-------------GCTCAAACCTCAG-C----------------CAAAA--CTCAATAGATACA---G----

------------------TGA--C----GAA-TGCCTGG----------T--A-AAAATTTC-AGA-CCA--

--AAATACCC-------AAGGA-GT-----AAGGC---AT-----ACTA------AG-TCCCAGA-CCGGGA

GTTAA-------------------------------------------------------------------

------------------------------------------------------------------------

--------------C--AAAATC--------------------------GG-----------------TTTT

-----------------------CGAAAACAAAA--C--GTCCTGGC-----TTCTCTTCCC----------

--------------CGTATC--TTCC-----------TTTTGTGA---------------------------

-------------------------TTC--GTTTAT-GGAC--GTG-CCT-CC-TTTCTT--GGGTGC--AA

AC-----------AACAT-A----CGAAAGT-GCTTTTG-T-------------GAATTT------------

----TTTTCA------------------------------GCGCAAT-A-C-G-GA-CCC-AGA-----AT-

GAAATT--GCATAAA--TTAGG------------CCGGAATTT--CACA-A-GTT-TCGG--TAGAA-----

---------GAT-AGCCT-------------------TGGTTTG--CCA----AAATTTCTGAAAAATTC

>Pv01Sk00120/1-541 Pv01Sk00120 undefined product 123384:123924 forward

CTCTCGGAATAG-----TTTTTTCCTG--AAATTCCACCC-AAG-AA--T-------CT---CCACTA-AA-

TTTGGG-AC----------AAAACGCG-AC-AA-TT--TTTAGG--TC------------------------

-----------------------------------------AAAC-G--GATGAG---TATTCGC-------

---CCC-----CGTAAAAAA---TC-AAACAGTT-TC--------------------------ACA-CAC--

AAAC--G-AA-CCT------------TCC--T-TTC-------A-G--CC-C-T-GG-C---------A--G

TGACG-AAT-A--A-A-AA--A-TT-TA-TGGCC-AGTCTTGT-GGGACGAA----TCT------GGT----

-------------GCTCAAACCATAG-C----------------CAAAA--CTCAATAGACACA---G----

------------------TGA--C----AAA-TGTCTGG----------T--A-AAAATTTC-AGA-CCA--

--AAATACCC-------AAGGA-GT-----AAGGT---GT-----AGTA------AG-TTACAGA-CCGATA

GTGAA-------------------------------------------------------------------

------------------------------------------------------------------------

--------------C--AAAACT--------------------------GT-----------------TTTT

C----------------------AGAAAACAAAA--C--GTCCTGAC-----TTTTCTTCCC----------

--------------CGTATC--TTCT-----------TTTTGTGA---------------------------

-------------------------TTT--GTTTAT-GGAC--GTG-CCT-CC-TTATCT--GGGTGC--AA

GC-----------AACAT-A----AGTAAAA-GTAGTTG-T-------------GTGAGG------------

---TTTTTCA------------------------------GTGCAAT-A-C-G-GA-CCG-AGA-----AT-

AAAATT--GCAGAAA--GTATA------------GCGAAATTT--TACA-A-GTT-TTTG--TAGAG-----

---------GAT-AGCAT-------------------TGG-TTTGCACA----AAATTTCTGAAAATTT-

>Pv01Sk00130/1-538 Pv01Sk00130 undefined product 223292:223829 forward

--CCCGAAATAG-----TTTTTTCCCA--AAATTCCAGGT-AAG-AA--T-------CT---CCACTG-AA-

TTTGGG-AC----------AAAACGCG-AC-AA-AT--TTCAGG--TC------------------------

-----------------------------------------AACC-T--GATGAG---TATTCAC-------

---CTA-----GGAAAGAAA---TC-AAACAGTT-TC--------------------------ACA-CAC--

AAAC--A-AA-CCT------------TCC--T-TTC-------A-G--TC-C-T-GG-C---------A--C

TGATC-AAT-A--A-A-AA--A-TT-TA-TTCCG-AATCTTGT-GGGACGAA----TTT------GGG----

-------------GCTCAAGTCTCAG-C----------------C-AAA-ACTCAGTAGACACA---A----

------------------TGA--C----GAA-TGTCCGG----------T--A-AAAATTTC-AGA-CCA--

--AAATACCC-------TAAGA-GT-----AAGGC---GC-----AGTA------AG-TCCCAGA-CCGAGG

GTGAA-------------------------------------------------------------------

------------------------------------------------------------------------

--------------C--GAAACT--------------------------GG-----------------TTTT

C----------------------CGAAAACAAAA--C--GTCCTGAC-----TTTTCCTCCC----------

--------------CGTATC--TTCT-----------TTTTGTGA---------------------------

-------------------------TTT--GTCTAT-GGAC--CTG-CCT-CC-TTACCT--GGGTGC--AA

AC-----------AACAT-A----TGAAAGT-ACTTGTG-T-------------GAA---------------

--TTTTTTCA------------------------------ACGCAAT-A-C-G-GA-CCC-AGA-----AT-

AAAATT--GCAGAAA--GTAGG------------ACGAAATTT--CACA-A-GTT-TCGG--TAAAG-----

---------GAT-AGTCT-------------------TGG-TTTTTACA----AAATTTCAGAAAAATTC

>Pv01Sk00140/1-539 Pv01Sk00140 undefined product 223831:224369 forward

--CCTGAAATAG-----TTTTTTCCCG--GAATTCCAGGT-AAG-AA--T-------CA---CCACTG-AA-

TTTGGG-AC----------AAAACGCG-AC-AA-AT--TTCAGG--TC------------------------

-----------------------------------------AAAC-G--GATGAG---TATTCAC-------

---CTA-----GGAAAGAAA---TC-AAACAGTT-TC--------------------------ACA-CAC--

AAAC--G-AA-CCT------------TCC--T-TTC-------A-G--CC-C-T-GG-C---------A--G

TGATG-AAT-A--A-A-AA--A-TT-TA-TTGCG-AATCTTGT-GGGACGAT----TTT------GGG----

-------------GCTCAAATCTCAG-C----------------T-AAA-ACTGAGTAGACACA---A----

------------------TGA--C----GAA-TGTCCGG----------T--A-AAAATTTC-AGA-CCA--

--AAATACCC-------AAGGA-GT-----AAGGC---GC-----AGTA------AG-ACCCAGA-CCGAAG

GTGAA-------------------------------------------------------------------

------------------------------------------------------------------------

--------------C--GAAACT--------------------------GG-----------------TTTT

C----------------------CGAAAACAAAA--C--GTCCTGGC-----TTTTCCTACC----------

--------------CGTATC--TTCT-----------TTTTGTGA---------------------------

-------------------------TTT--GTCTAT-GGAC--CTG-CCT-CC-TTACCT--GGGTGC--AA

AC-----------AACAT-A----TGAAAGT-ACTTGTG-T-------------GAA---------------

--TTTTTTCA------------------------------ACGCAAT-A-C-G-GA-CCG-AGA-----TT-

AAAATT--GCAGAAA--GTAGG------------GCGAAATTT--CAAA-A-GTT-TCGG--TAGAG-----

---------GAT-AAGCCC------------------TGG-TTTTTACA----AAATTTCTGAAAAATTC

>Pv01Sk00150/1-508 Pv01Sk00150 undefined product 225448:225955 forward

--CCTGAAATAG-----TTTTTTCCCA--AAATTCCAGGT-AAG-AA--T-------CA---CCATTG-AA-

TTTGGG-AC----------AAAACGAG-AC-AA-AT--TTCAGG--TC------------------------

-----------------------------------------AAAC-G--GTTGAG---TATTCAC-------

---CTA-----GGAAAGAAA---TC-AAACAGTT-TC--------------------------ACA-CAC--

AAAC--G-AA-CCT------------TCC--T-TTC-------A-G--CC-C-T-GG-C---------A--C

TGATC-AAT-A--A-A-AA--A-TT-TA-TTGCG-AATCTTGT-GGGACGAA----TTT------GGG----

-------------GCTCAAGTCTCAG-C----------------C-AAA-AATCAGTAGACACA---A----

------------------TGA--C----GAA-TGTCCGG----------T--A-AAAATTTC-AGA-CCA--

--AAATACCC-------TAAGA-GT-----AAGGC---G---------------------------------

------------------------------------------------------------------------

------------------------------------------------------------------------

---------------------CT--------------------------GG-----------------TTTT

C----------------------CGAAAATAAAA--C--GTCCTGGC-----TTTTCCTCCC----------

--------------CGTATC--TTCC-----------TTTTGTGA---------------------------

-------------------------TTT--GTCTAT-GGAC--CTG-CCT-CC-TTACCT--GGGTGC--AA

AC-----------AACAT-A----TGAAAGT-ACTTGTG-T-------------TAA---------------

--TTTTTTCA------------------------------ACGCAAT-A-C-G-GA-CCC-AGA-----TT-

AAAATT--GCACAAA--GTAGG------------ACGAAATTT--CACA-A-GTT-TCGG--TAGAG-----

---------GAT-AGCCT-------------------TGG-TTTTCACA----AAATTTCTGAAAAATTC

>Pv01Sk00160/1-538 Pv01Sk00160 undefined product 225957:226494 forward

--CCCGAAATAG-----TTTTTTCCCA--AAATTCCAGGT-AAG-AA--T-------CA---CCACTG-AA-

TTTGGG-AC----------AAAACGCG-AC-AA-AT--TTCAGG--TC------------------------

-----------------------------------------AAAA-G--GATGAG---TATTCAG-------

---CTA-----GGGAAGAAA---TC-AAACAGTT-TC--------------------------ACA-CAC--

AAAC--G-AA-CCT------------TCC--T-TTC-------A-G--CC-C-T-GG-C---------A--G

TGATG-AAT-A--A-A-AA--A-TT-TA-TTGCG-AATCTTGT-GGGACGAA----TTT------GGG----

-------------GCTCAAATCTCAG-C----------------C-AAA-ACTCAGTAGACACA---A----

------------------TGA--C----GAA-TGTCCGG----------T--A-AAAATTTC-AGA-CCA--

--AAATACCC-------TAAGA-GT-----AAGGC---GC-----AGTA------AG-TCCCAGA-CCGAGG

GTGAA-------------------------------------------------------------------

------------------------------------------------------------------------

--------------C--GAAACT--------------------------GG-----------------TTTT

C----------------------CGAAAACAAAA--C--GTCCTGGC-----TTTTCCTCCC----------

--------------CGTATC--TTCT-----------TTTTGTGA---------------------------

-------------------------TTT--GTCTAT-GGAC--CTG-CCT-CC-TTACCT--GGGTGC--AA

AC-----------AACAT-A----TGAAAGT-ACTTGTG-T-------------GAA---------------

--TTTTTTCA------------------------------ACGCAAT-A-C-G-GA-CCC-AGA-----AT-

AAAATT--GCAGAAA--GTAGG------------ACGAAATTT--CACA-A-GTT-TCGG--TAGAG-----

---------GAT-AGCCT-------------------TGG-TTTTTACA----AAATTTCTGAAAAATTC

>Pv01Sk00170/1-538 Pv01Sk00170 undefined product 227576:228113 forward

--CCCGAAATAG-----TTTTTTCTTG--AAATTCCAGGT-AAG-AA--T-------CT---CCAATG-AA-

TTTGGG-AC----------AAAACGCG-AC-AA-AT--TTCAGA--TC------------------------

-----------------------------------------AAAC-G--GATGAG---TATTCAA-------

---CTA-----GGAAAGAAA---TC-AAACAGTT-TC--------------------------ACA-CAT--

ACAC--A-AA-CCT------------TCC--T-TTC-------A-G--CC-C-T-CA-C---------A--G

TGACG-AAT-A--A-A-AA--T-TT-TA-TTGCC-AATCTCGT-GGAACGAA----TCT------GGG----

-------------GCTCAAATCTCAG-C----------------CAAAT--CTCAATAGACACA---G----

------------------TGA--C----GAA-TGTCTGG----------T--A-AAAATTTT-AGA-CCA--

--AAATACCC-------AAGGA-GT-----AAGGC---GT-----AGTA------AG-TCCTAGA-CCGAGA

GTGAA-------------------------------------------------------------------

------------------------------------------------------------------------

--------------C--AAAACA--------------------------GG-----------------TTGT

C----------------------CGGAA--AAAA--C--TTCCTGCC-----TTTTCTTCCC----------

--------------CGTATC--TTCT-----------TTTTGTGA---------------------------

-------------------------TTC--GTTTAT-GGAC--GTG-CCT-CC-TTGCCT--GCGTGC--AA

AC-----------AACAT-A----CGAAAGT-GCTTATG-T-------------GAGTTT------------

---TGTTTCA------------------------------GCGCAAT-A-C-A-GA-CCC-AGA-----AT-

GAAAAT--GCTGAAA--TTAGG------------CCGGAATTT--AACA-A-GTT-TCGG--TAGAG-----

---------GAT-AGCCT-------------------TGG-TTTGCACA----AAATTTATGAAAAATTC

>Pv01Sk00180/1-525 Pv01Sk00180 undefined product 228113:228637 forward

CTCCCGGAATAG-----TTTTTTCCTG--AAATTCCACCT-AAG-AA--T-------CT---CCACTG-AA-

TTTGGG-AC----------AAAACGCT-AC-AA-AT--TTCACG--TC------------------------

-----------------------------------------AAAT-G--AATGAG---TATTCAC-------

---CCA-----CGAAAAGAA---TC-AAACAGTT-TA--------------------------ACA-CAC--

AAAA--G-AA-CCT------------TCC--T-GTC-------A-G--CC-C-T-GG-C---------A--A

AGACG-AAT-A--A-A-TA--A-TT-TA-TTGCC-AATCTTGT-GGGACGAA----TTT------GGG----

-------------GCTCAAACCTCAA-C----------------CAAAA--CTCAATAGGCAAA---G----

------------------TGA---------------------------------AAAATGTC-AGA-CCA--

--AAATACCC-------AAGAA-GT-----AAGGC---GT-----AGAA------AG-TCTCATA-CCGAGA

GTGAA-------------------------------------------------------------------

------------------------------------------------------------------------

--------------C--AAAATC--------------------------GG-----------------TTTT

T----------------------CGAAAACAAAA--C--GTCCTGGC-----TTTTCTTCCC----------

--------------CGTATC--TTCT-----------TTTTCTGA---------------------------

-------------------------TTC--GTTTTT-GGAC--GTG-CCT-CC-TTGCCT--GGGTGC--AA

AC-----------AACAT-A----CGAAAGT-GCTTGTG-T-------------GAATTT------------

----TTTTCA------------------------------GCGCAAT-A-C-G-GA-CCC-AGA-----AT-

TAAATT--GCAGAAA--TTAGG------------CCGAAATTT--CAGA-A-GTT-TCGG--TAGAG-----

---------GAT-AGCCT-------------------TGG-TCTGCACA----AAATTTCTGAAAAT---

>Pv01Sk00190/1-537 Pv01Sk00190 undefined product 229180:229716 forward

--CCTGAAATAG-----TTTTTTCCCG--AAATTCCAGGT-AAG-AA--T-------CA---CCATTG-AA-

TTTGGG-AC----------AAAACGAG-AT-AA-AT--TTCAGG--TC------------------------

-----------------------------------------AAAC-G--GTTGAG---TATTCAG-------

---CTA-----GGAAAGAAA---TC-AAACAGTT-TC--------------------------ACA-CAC--

AAAC--G-AA-CCT------------TCC--T-TTC-------A-G--CC-C-T-GG-C---------A--G

TGATG-AAT-A--A-A-AA--A-TT-TA-TTGCG-AATCTTGT-GGGACGAT----TTT------GGG----

-------------GCTCAAATCTCAG-C----------------C-AAA-ACTGAGTAGACACA---A----

------------------TGA--C----GAA-TGTCCGG----------T--A-AAAATTTC-AGA-CCA--

--AAATACCC-------AAGGA-GT-----AAGGC---GC-----AGTA------AG-TCCCAGA-CCGAGG

GTGAA-------------------------------------------------------------------

------------------------------------------------------------------------

--------------C--GAAACT--------------------------GG-----------------TTTT

C----------------------CAAAAACAAAA--C--GTCCTGGC-----TTTTCCTCCC----------

--------------CGTATC--TTCT-----------TTTTGTGA---------------------------

-------------------------TTT--GTCTAT-GGAC--CTG-CCT-CC-TTACCT--GGGTGC--AA

AC-----------AACAT-A----TGAAAGT-ACTTGTG-T-------------GAA---------------

---TTTTTGA------------------------------ACGCAAT-A-C-G-GA-CCC-AGA-----AT-

AAACTT--GCAGAAA--GTATG------------ACGAAATTT--CACA-A-GTT-TCGG--TAGAG-----

---------GAT-AGCCT-------------------TGG-TTTTTACA----AAATGTCTGAAAAATTC

>Pv01Sk00200/1-507 Pv01Sk00200 undefined product 229718:230224 forward

--CCCGAAATAG-----TTTTTTCTTG--AAATTCCAGGT-AAG-AA--T-------CA---CCATTG-AA-

TTTGGG-AC----------AAAACGAG-AC-AA-AT--TTCAGG--TC------------------------

-----------------------------------------AAAC-G--GTTGAG---TATTCAC-------

---CTA-----GGAAAGAAA---TC-AAACAGTT-TC--------------------------ACA-CAC--

AAAC--G-AA-CCT------------TCC--T-TTC-------A-G--CC-C-T-GG-C---------A--C

TGATC-AAT-A--A-A-AA--A-TT-TA-TTGCG-AATCTTGT-GGGACGAA----TTT------GGG----

-------------GCTCAAGTCTCAG-C----------------C-AAA-AATCAGTAGACACA---A----

------------------TGA--C----GAA-TGTCCGG----------T--A-AAAATTTC-AGA-CCA--

--AAATACCC-------TAAGA-GT-----AAGGC---GC-----AGTA-----------------------

------------------------------------------------------------------------

------------------------------------------------------------------------

----------------------------------------------------------------------TT

C----------------------TGAAAACAAAA--C--GTCCTGGC-----TTTTCCTTCC----------

--------------CGTATC--TTCT-----------TTTTGTGA---------------------------

-------------------------TTT--GTCTAT-GGAC--CTG-CCT-CC-TTACCT--GGGTGC--AA

AC-----------AACAT-A----TGAAAGT-ACTTGTG-T-------------GAA---------------

--TTTTTTCA------------------------------ACGCAAT-A-C-G-GA-CCC-AGA-----AT-

AAAATT--GCAGAAA--GTAGG------------GCGAAATTT--CAAA-A-GTT-TCGG--TAAAG-----

---------GAT-AGCCT-------------------TGG-TTTTTACA----AAATTTCTGAAAAATTC

>Pv01Sk00210/1-538 Pv01Sk00210 undefined product 230226:230763 forward

--CCCGAAATAG-----TTTTTTCCCG--AAATTCCAGGT-AAG-AA--T-------CT---CCACTG-AA-

TTTGGG-AC----------AAAACGCG-AC-AA-AT--TTCAGG--TC------------------------

-----------------------------------------AAAC-G--GATGAG---TATTCAC-------

---CTA-----GGAAAGAAA---TC-AAACAGTT-TC--------------------------ACA-CAC--

AAAC--G-AA-CCT------------TCC--T-TTC-------A-G--CC-C-T-GG-C---------A--G

TGATG-AAT-A--A-A-AA--A-TT-TA-TTGCG-AATCTTGT-GGGACGAA----TTT------GGG----

-------------GCTCAAATCTCAG-C----------------C-AAA-ACGCAGTAGACACA---A----

------------------TGA--C----GAA-TGTCCGG----------T--A-AAAATTTC-AAA-CCA--

--AAATACCC-------AAGGA-GT-----AAGGC---GC-----AGTA------AG-TCCCAGA-CTGAGA

GTGAA-------------------------------------------------------------------

------------------------------------------------------------------------

--------------C--GAAACT--------------------------GG-----------------TTTT

C----------------------CGAAAACAAAA--C--GTCCTGGC-----TTTTCCTCCC----------

--------------CATATC--TTCT-----------TTTTGTGA---------------------------

-------------------------TTT--GTCTCT-GGAC--CTG-CCT-TC-TTACCT--GGGTGC--AA

AC-----------AACAT-A----TGAAAGT-ACTTGTG-T-------------GAA---------------

--TTTTTTCA------------------------------ACGCAAT-A-C-G-GA-CCC-AAA-----AT-

AAAATT--GTAGAAA--GTAGG------------GCGAAAGTT--CACA-A-GTT-TCAA--TAGAG-----

---------GAT-AGTCT-------------------TGG-TTTTTACA----AAATTTCTGAAAAATTC

>Pv01Sk00220/1-535 Pv01Sk00220 undefined product 230765:231299 forward

--CCCGAAATAG-----TTTATTCCCG--AAATTCCAGGT-AGG-AA--T-------CT---CCACTG-AA-

ATTGGG-AC----------AAAACGCG-AC-AA-AT--TTCAGG--TC------------------------

-----------------------------------------AAAC-G--GATGAG---TATTCAC-------

---CTT-----GGAAAGAAA---TC-AAACAGTT-TC--------------------------ACA-CAC--

AAAC--G-AA-CCT------------TCC--T-TTC-------A-G--CC-C-T-GA-C---------A--G

TGATG-AAT-A--A-A-AA--A-TT-TA-TTGCG-AATCTTGT-GGGACGAA----TTT------GGG----

-------------GCTCAAATCTCAG-C----------------C-AAA-ACTCAGTAGACACA---A----

------------------TGA--C----GAA-TGTCCGG----------T--A-AAAGTTTC-AGA-CCA--

--AAATACCA-------AAGGA-GT-----AAGGC---GC-----AGTA------AG-TCCCAGA-CCAAGA

GTGAA-------------------------------------------------------------------

------------------------------------------------------------------------

--------------C--GAAACT--------------------------GG-----------------TTTT

C----------------------CGAAAACAAAA--C--GTCCTGGC-----TTTTCCTACC----------

--------------CGTATC--TTCT-----------TTTTGTGA---------------------------

-------------------------TTT--GTCTAT-GGAC--CTG-CCT-CC-TTACCT--GGTTGA--AA

AC-----------AACAT-A----TGAAAGT-ACATGTG-T-------------GAA---------------

-TTTTTTTCA------------------------------ACGCAAT-A-C-G-GA-CCT-AGA-----AT-

AAAATT--GCAGAAA--GTAGG------------ACGAAATTT--CAAG-A-GTT-TCGG--TAGT------

-------------CGCCT-------------------TGG-TTTTTACA----AAATTTCTGAAAAATTC

>Pv01Sk00230/1-536 Pv01Sk00230 undefined product 231301:231836 forward

--CCCGAAATTG-----TTTTTTCCCT--AAATTACAGTT-AAG-AA--T-------CT---CCACTG-AA-

TTTGGG-AC----------AAAACGCG-AC-AA-AT--TTCAGG--TC------------------------

-----------------------------------------AAAC-G--GATGAG---TATTCAC-------

---CTA-----GGAAAGAAA---TC-AAACAGTT-TC--------------------------ACC-CAC--

AGAC--G-AA-TCT------------TCC--T-TTC-------A-G--CC-C-T-GG-C---------A--G

TGATG-AAT-A--A-A-AG--A-TT-TA-TTGCG-AATTTTGT-GGGACGAA----TAT------GGG----

-------------GCTCAAATCTCAG-C----------------C-AAA-ACTCAGTAGACAAA---A----

------------------TGA--C----GAA-TGTCCGG----------T--A-AAAATTTC-AGA-CCA--

--AAATACCC-------AAGGA-GT-----AAGGT---GC-----ACTA------AG-TCCCAGA-CCGAGA

GTGAA-------------------------------------------------------------------

------------------------------------------------------------------------

--------------C--GAAACT--------------------------GG-----------------TTTT

T----------------------CGAAAACAAAA--C--GTCCTTGC-----TTTTCCTCCC----------

--------------CGTATC--TTCT-----------TTTTGTGA---------------------------

-------------------------TTT--GTCTCT-GGAC--GTG-CCT-CC-TTACCT--AGGTGC--AA

AC-----------AACAT-A----TAAAGTA-CTTGTTG-T-------------A-----------------

--TTTTTTCA------------------------------ACCCAAT-A-C-G-GA-CCG-AGA-----AT-

AAAATT--GCAGAAA--GTAGG------------GCGAAATTT--CACA-A-TTT-TCTG--TAGAG-----

---------GAT-AGCCT-------------------TGG-TTTTTACA----AAATTTTTGAAAAATTC

>Pv01Sk00240/1-540 Pv01Sk00240 undefined product 231838:232377 forward

--CCCGAAATAG-----TTTTTTCCCT--AAATTACAGGT-AAG-AA--T-------CT---CCACTG-AA-

TTTGGG-AC----------AAAACGCG-AC-AA-AT--TTCAGG--TC------------------------

-----------------------------------------AAAC-G--GATGAG---TATTCAC-------

---CTA-----GAAAAGAAA---TC-AAACAGTT-TC--------------------------ACC-CAC--

AGAC--G-AA-TCT------------TCC--T-TTC-------A-G--CC-C-T-GG-T---------A--G

TGATG-AAT-A--A-A-AG--A-TT-TA-TTGCG-AATCTTGT-GGGACGAA----TAT------GGG----

-------------GCTCAAATCTCAG-C----------------C-AAA-ACTCAGTAGACACA---A----

------------------TGA--C----GAA-TGTCCGG----------T--A-AAAATTTC-AGA-CCA--

--AAATACCC-------AAGGA-GT-----AACGC---GC-----AGTA------AG-TCCCAGA-CCGAGA

GTGAA-------------------------------------------------------------------

------------------------------------------------------------------------

--------------C--GATACT--------------------------GG-----------------TTTT

C----------------------CGAAAACAAAA--C--GTCCTGGC-----TTTTCCTCCC----------

--------------CGTAAC--TTCT-----------TTTTGTGA---------------------------

-------------------------TTT--GTCTCT-GGAC--CTG-CCT-CC-TTACCT--GGGTGC--AA

AC-----------AACAT-A----TGAAAGT-ACTTGTG-T-------------GAA---------------

--TTTTTTCA------------------------------GCGCAAT-A-G-G-GA-TTC-GGA-----AT-

AAAATT--GCAGAAA--GCAGG------------GCAAAATTT--CACA-A-GGT-TCGT--TAGAGAAT--

---------AAT-AGTCT-------------------TGG-TTTGCACA----AAATTTCTGAAAAAAT-

>Pv01Sk00250/1-575 Pv01Sk00250 undefined product 368098:368672 reverse

--TCCCGAAAAG----ATTTTTTGCTG--AAATTCTATGT-AAT-AG--T-------CT---CAACAA-AA-

TTTGGG-AC----------AAAACGCG-AC-AA-AT--TTCAGG--TC------------------------

-----------------------------------------AAAC-A--GTTGAA---TATTCAG-------

---TCA-----GTAAAAAAA---TC-AAACAATT-TC--------------------------ACA-CAC--

AGAGGCG-AA-CCT------------TCC--T-TTC-------G-G--CT-T-T-GG-C---------A--G

TGACG-AAT-A--AAA-AT----TT-CA-TTGCA-AAGCTTAT-GGAACGAA----TTT------GAG----

-------------GCTCAAACCTTGG-C----------------C-ACA-ACTGAGTAGACACA---A----

------------------AAA--A----GAA-TGTCAGA----------T---AAAAATTTC-ATATCAA--

---AACACCC-------AGGAA-AT-----AAGGC---GT-----AACA------AG-TCCCAGA-CCGAGA

GGAAA-------------------------------------------------------------------

------------------------------------------------------------------------

--------------C--AAAATT--------------------------GAAAAA-CATG-------TTTTT

-----------------------CTAAAACAGAA--C--ATCCTGGA-----TGTTCCTCGT----------

--------------CATATC--TTTTTTT--------TTTTGTGT---------------------------

-------------------------TTT--GTTTTT-TGAT--GTG-CCT-CC-TTAGTC--GAATGC--AA

AC-----------AACAT-A----TGGAAGT-ATCTGTG-T-------------GTATTTTTTTTTC----G

GTTTTTCTCAACGTAATATGAGCTCTGAAAAAAAAAATCAACCTAAT-A-TGA-AC-TCT-GGA--------

AAAATT--GCAAAGA--GGATG------------AAAAATTTT--CAAA-A-ATA-TGAA--AAGAG-----

---------GAT-AGCCC-------------------------------------------GAAGAATTT

>Pv01Sk00270/1-540 Pv01Sk00270 undefined product 369149:369688 reverse

CTCCCGAAATAG-----GTTTTTCCTG--AAATTCCACGT-AAG-AA--T-------CT---CCACTG-AA-

TTTGGG-AC----------AAAACGCG-AC-AA-AT--TTCAGG--TC------------------------

-----------------------------------------AAAC-G--GATGAG---TATTCAC-------

---CCA-----CGAAAAAAA---TC-AAACAGTT-TC--------------------------GCA-CAC--

AAAA--G-AA-CCT------------TCC--T-TTC-------A-G--GC-C-T-GA-C---------A--G

TGATG-AAT-A--A-A-AA--A-TT-TA-TTGTC-AATCTCGT-GCGACAAA----TTT------GGG----

-------------GCTCAAATCTCAG-C----------------CAAAA--CTCAATAGACACA---G----

------------------TAA--C----GAA-TGTCTGG----------T--A-AAAATTTC-AGA-CCA--

--AAATGCCC-------AAGGA-GT-----AAGGC---GC-----AGTA------AG-TCCCAGA-CCAAGA

GTGAA-------------------------------------------------------------------

------------------------------------------------------------------------

--------------C--AAAACC--------------------------GG-----------------TTTT

T----------------------CGAAAACGAAA--C--GTCCTGGA-----TTTTCTACCC----------

--------------CGTATC--TTCT-----------TTCTGTGA---------------------------

-------------------------TTT--GTTTAT-GGAC--GTG-CCT-CC-TTACCT--GGGTGC--AA

AC-----------AACAT-A----TGAAAGT-ACTTGTG-T-------------GATT--------------

---TTTTTCA------------------------------GCGCCAT-C-C-G-GA-CCT-AGT-----AT-

GAATTT--GCAGAAA--TTAGG------------GCATAATTT--CACA-A-GTT-TCGA--TAGAT-----

---------GAT-AGCCT-------------------TTG-TTTGCACA----AAATTTCTGAAAAATTC

>Pv01Sk00280/1-542 Pv01Sk00280 undefined product 369688:370229 reverse

CTCCCGAAATAG-----TTTTTTCTTG--AAATTCCACGT-AAG-AA--T-------CT---CCACTG-AA-

TTTGGG-AG----------AAAACGCG-AC-AA-AT--TTCAAC--TC------------------------

-----------------------------------------TAAC-G--GATGAG---TATTCAC-------

---CCA-----CGAAAAAAA---TC-AAACAGTT-TC--------------------------ACA-CAC--

AAAAACG-AA-CCT------------TCC--T-TTC-------A-G--TC-C-T-GA-T---------A--G

TGATG-AAT-A--A-A-AA--A-TT-TA-TTGCC-AATCTCGT-GGGACGAA----TCT------GGG----

-------------GCTCAAATCTCAG-C----------------CAAAA--CTCAATAGACACA---G----

------------------TGA--C----GAA-TGTCTGG----------T--A-AAAATTTC-AGA-CCA--

--AAATACCC-------AAGGT-GT-----AAGGC---GC-----AGTA------AG-TCCCAGA-CCGAGA

GTGAA-------------------------------------------------------------------

------------------------------------------------------------------------

--------------T--AAAACC--------------------------GG-----------------TTTT

C----------------------CGAAAACAAAA--C--GTCCTGGA-----TTTTCTTCCC----------

--------------CGTATC--TTCT-----------TTCTGTGA---------------------------

-------------------------TTT--GTTTAT-GGAC--GTG-CCT-CC-TTACCT--GGTTGC--AA

AC-----------AACAT-A----TGAAAGT-ACTTGTG-T-------------GAAA--------------

---TTTTTCA------------------------------TTGCAAT-A-C-G-GG-CCC-AGG-----AT-

GAAATT--GCAGAAA--TTAGG------------ACAAAATTT--CACA-A-GTT-TCAG--TAGAG-----

---------GAT-AGCCT-------------------TGG-TTTGCACA----AAATTTCTGAAAAATTC

>Pv01Sk00290/1-539 Pv01Sk00290 undefined product 370229:370767 reverse

CTCCCGAATTAC-----TTTTTCCCCA--AAATTCCACGT-AAG-AA--T-------CT---CCGTTG-AA-

TTTGGG-AC----------AAAACGCT-AC-AA-AT--TTCAGG--TC------------------------

------------------------------------------AAC-G--GATGAG---TATTCAG-------

---CCA-----CGAAAAAAA---TC-AAATAGTT-TC--------------------------GCA-CAC--

AAAC--G-AA-CCT------------TCC--T-TTC-------A-A--CC-C-T-GG-C---------A--G

TGACG-AAT-A--A-A-AA--A-TT-TA-TTGCC-AATCTCGT-GGGACAAA----TCT------GTG----

-------------GCTTAAATCTCAG-C----------------CAAAA--CTCAATAGACATA---G----

------------------TGA--C----GAA-TATCTGG----------T--A-AAAATTTC-ATA-CCA--

--AAATACCC-------AAGGA-GT-----AAGGC---GT-----AGTA------AG-TCCCAGA-CTGGGA

GTGAA-------------------------------------------------------------------

------------------------------------------------------------------------

--------------C--AAAACC--------------------------GG-----------------TTTT

C----------------------CGAAAACAAAA--C--GTCCTGGA-----TTTTCTTCCC----------

--------------CGTATC--TTCC-----------TTTTGTGA---------------------------

-------------------------TTT--GTTTAT-GGAC--GTG-CTT-CC-TTACCT--TGTTGC--AA

AC-----------AACAT-A----TGAAAGT-ACTTGTG-C-------------GAAT--------------

---TTTTTCA------------------------------TCGCAAT-A-C-G-GG-CCC-AAG-----AT-

GAAATT--GCAGAAA--TTAGG------------GCAGAATTA--CACA-A-GTT-TCTG--TAGAG-----

---------GAT-AGCCT-------------------TGT-TTTGCACA----ATATTTCTGAAAAATTC

>Pv01Sk00300/1-540 Pv01Sk00300 undefined product 370767:371306 reverse

CTCCCGAATTAC-----TTTTTCCCCA--AAATTCCATGT-AAG-AA--T-------CT---CCACTG-AA-

TTTGGG-AC----------AAAACGCG-AC-AA-AT--TTCAGG--TC------------------------

-----------------------------------------AAAC-G--GATGAG---TATTGAA-------

---CCA-----CGAAAAAAA---TC-AAACAGTT-TC--------------------------ACA-CAC--

AAAC--G-AA-CCT------------TCC--T-TTC-------A-G--CC-C-T-GG-C---------A--G

TGATA-AAT-A--A-A-AA--T-TT-TA-TTATC-AATCTCGT-GGGACGAA----TCT------GGG----

-------------GCTCAAATCTGAG-C----------------CAAAA--CTCAATAGACACA---G----

------------------TGA--C----GAA-TGTCTGG----------T--A-AAAATTTC-AGA-CCA--

--AAATACCC-------AAGGA-GT-----AAGGC---GC-----AGTA------AG-TCCCAGA-CCGAGA

GTGAA-------------------------------------------------------------------

------------------------------------------------------------------------

--------------C--AAAACC--------------------------GG-----------------TTTT

C----------------------CAAAAACGAAA--C--GTCTTGGA-----TTTTCTTCCC----------

--------------CGTATC--TTCT-----------TTCTGTGA---------------------------

-------------------------TTT--GTTTAT-GGAC--GTG-CCT-CC-TTACCT--GGTTGC--AA

AC-----------AACAT-A----TGAAAGT-ACTTGTG-T-------------GAAA--------------

---TTTTTCA------------------------------TCGCAAT-A-C-G-GG-CCC-AAG-----AT-

GAAATT--GCAGAAA--TTAGG------------ACAAAATTT--CACA-A-GTT-TCAG--TAGAG-----

---------GAT-AGCCT-------------------TGG-TTTGCACA----AAATTTCTGAAAAATTC

>Pv01Sk00310/1-540 Pv01Sk00310 undefined product 371306:371845 reverse

CTCCCGAATTAC-----TTTTTCCCCC--AAATTCCATGT-AAG-AA--T-------CT---CCACTG-AA-

TTTGGG-AC----------AAAACGTG-AC-AA-AT--TTCAGG--TC------------------------

-----------------------------------------AAAC-A--GATGAG---TATTCAC-------

---CCA-----CGAAAAAAA---TC-AAACAGTT-TC--------------------------CCA-CAC--

AAAC--G-AA-CCT------------TCC--T-TTC-------A-G--CC-C-T-GA-C---------A--G

TGATG-AAT-A--A-A-CA--A-TT-TA-TTGCC-AATCTCGT-GGGACAAA----TCT------GGG----

-------------GCTCAAATCTCAG-C----------------CAAAA--CTTAATAGACACA---G----

------------------TGA--C----GAG-TGTCTGG----------T--A-AAAATTTC-AGA-CCA--

--AAATGCCC-------AAGGA-GT-----AAGGC---GC-----AGCA------AG-TCCCAGA-CCGAGA

GTGAA-------------------------------------------------------------------

------------------------------------------------------------------------

--------------C--AAAATC--------------------------GG-----------------TTTT

C----------------------CGAAAACGAAA--C--GTCTTGGA-----TTTTCTACCC----------

--------------CGTATC--TTCT-----------TTCTGTGA---------------------------

-------------------------TTT--GTTTAT-GAAC--GTG-CCT-CC-TTACCT--GGGTGC--AA

AC-----------AACAT-A----TGAAAGT-ACTTGTG-T-------------GAAT--------------

---TTTTTCA------------------------------GCGCAAT-A-C-G-GA-CCC-AGA-----AT-

GAAATT--GCATAAA--TTAGG------------GCGAAATTT--CACA-A-GTT-TCGG--TAGAG-----

---------GAT-AGCCT-------------------TGG-TTAACACA----AAATTTATTAAAAATTC

>Pv01Sk00320/1-541 Pv01Sk00320 undefined product 446742:447282 forward

CTCCCGAAATTA-----CTTTATACTG--AAATTTCACGT-AAG-AA--T-------CT---CAACTG-AA-

TTTGGC-AC----------AAAACACA-AC-GA-AT--TTTAGG--TC------------------------

-----------------------------------------AAAC-A--AATGAG---TATTCAC-------

---CTA-----GGAAAAAAA---TC-AAACACTT-TC--------------------------ACA-AAC--

AGAC--G-AA-CCT------------TCC--T-TTC-------A-G--CC-C-A-GT-C---------A--G

TGAGT-AAT-A--A-A-AA--A-AT-CA-TGGCA-AATCTTGT-GGGACGAA----TTT------GGG----

-------------GCTCAAATCTTAG-C----------------CAAAA-CTCAAC-AGACACA---G----

------------------TAT--C----GAA-CGTCTGG----------T--A--CAAATTTCAGA-CCA--

--AAATACAC-------AAGGA-GT-----AAGAC---GT-----TGTA------AG-TCCCAGA-CCGAGC

GTGAA-------------------------------------------------------------------

------------------------------------------------------------------------

--------------C--AAAACT--------------------------GG-----------------TTTT

C----------------------CGAAAACAAAA--C--GTCCTAGC-----TTTTCTTCCC----------

--------------CGTATC--TTCT-----------TTTTGTGA---------------------------

-------------------------TTT--GTTTAG-GGAT--GTG-CCT-CC-TTACCT--GGGTGA--AA

AC-----------AACAT-A----TGAAAGT-ACTTGTG-T-------------GAT---------------

--TATTTTCA------------------------------GCCCAAT-A-C-G-GA-CTC-TAA-----AT-

AAAACT--GCAGAAT--GTAGG------------GCAAAATTT--CAAA-A-GTT-TTGG--TAGCGG----

---------GAT-AGCCT-------------------TGG-TTTGCACA----AAATTTGTAAAAAATTC

>Pv01Sk00330/1-540 Pv01Sk00330 undefined product 447283:447822 forward

-TACCAAAATAG----TTTTTTTCCTG--AAATTCCACGT-TAG-AA--T-------AT---CCACTG-AA-

TTTGGG-AC----------AAAACGCG-AC-AA-AT--TTCAGG--TC------------------------

-----------------------------------------AAAC-G--AATGAG---TATTCAC-------

---CCA-----CGAAAAAAA---TC-AAACAGGT-TT--------------------------GCA-CAC--

AAAC--A-AA-CCT------------TAC--T-TTC-------A-G--CA-C-T-GG-C---------A--G

TGATG-AAT-A--A-A-AA--A-TT-TA-TTGCC-AATCTTGC-AAGATGAA----TCT------GGG----

-------------GCTCAAATATCAG-C----------------AAAAA-CTCAAC-AGACACA---C----

------------------TGA--C----GAA-CGTCTGG----------T--A--AAATTTTCAGA-CCA--

--AAATACCC-------AAGGA-GT-----AAGGC---GT-----AGTG------AG-TGCCAAA-CCGAGA

GTGAA-------------------------------------------------------------------

------------------------------------------------------------------------

--------------A--AAAACT--------------------------GG-----------------TTTT

C----------------------CGAAAACAAAA--C--GTCCTGGC-----TTTTCATCTC----------

--------------CGTATC--TTCC-----------TTTTGTGA---------------------------

-------------------------TTT--CTTTAC-GGAC--GTG-CCT-CC-TTACCT--GGGTGC--AA

AC-----------AACAT-A----TGAAAGT-ACTTGTG-C-------------GAA---------------

--TTTTTTCA------------------------------GCGCCAT-A-C-G-GA-CCC-AGA-----AT-

AAAATT--GCAGAAA--GTAGG------------GCGAAATTT--CACA-A-GTT-TTGG--TAGAG-----

---------GAT-AGCCT-------------------TGT-TTTGCACA----AAATTTCTGAAAAATTC

>Pv01Sk00340/1-541 Pv01Sk00340 undefined product 447822:448362 forward

CTCCGGAAATAG-----TTTTTTCCTG--AAAGTCCACGT-AAG-AA--T-------CT---CCACTG-AA-

TTTGGG-AC----------AAAACGCC-AC-AA-AT--TTCAGG--TC------------------------

-----------------------------------------AAAC-G--GATGAG---TATTCAC-------

---CCA-----CGAAAAAAA---TC-AAACAGTT-TC--------------------------GCA-CAC--

AAAC--G-AA-CCT------------TCC--T-TTC-------A-G--CC-T-T-GG-C---------A--G

TGATG-AAT-A--A-A-AA--A-TT-TA-TTGTA-GATCCCGT-GGGACGAA----TCT------GGG----

-------------GCTCAAATCTCAG-C----------------CAAAA-CTCAAC-AGACACA---C----

------------------TGA--C----GAA-CGTCTGG----------T--A--AAAATTTCATA-CCA--

--AAATACCC-------AAGGA-GT-----AAGGC---GT-----AGTG------AG-TCCCAGA-CCGAGA

GTGAA-------------------------------------------------------------------

------------------------------------------------------------------------

--------------C--AAAACT--------------------------GG-----------------TTTT

T----------------------CGAAAACAAAA--C--GTCCTGGC-----TTTTCTTCCC----------

--------------CGTATC--TTCC-----------TTTTGTGG---------------------------

-------------------------TTT--CTTTAC-GGAC--GTG-CCT-CC-TTACCT--GGGTGC--AA

AC-----------AACAT-A----TGAAAGT-ACTTGTG-C-------------GAA---------------

--TTTTTTCA------------------------------GCGCAAT-A-C-G-GA-CCC-AGA-----AT-

AAAATTT-TCAGAAA--GTAGG------------GCGAAATTT--CACA-A-GTT-TTGG--TAGAG-----

---------CAT-AGTCT-------------------TGC-ACTGCACA----AAATATCTGAAAAATTC

>Pv01Sk00350/1-538 Pv01Sk00350 undefined product 448366:448903 forward

----GAAAATAG-----TTCTTTCCTG--AAATTCCACGA-AAG-AA--T-------CT---CCACTG-AA-

TTTGGG-AA----------AAAACGCC-AC-AATAT--TTCAGG--TC------------------------

-----------------------------------------AAAC-G--GATGAG---TATTCAC-------

---CCA-----CGAAAAAAA---TC-AAACAGTT-TC--------------------------GCA-CAC--

AGAC--G-AA-CCT------------TCC--T-TTC-------A-G--CC-C-T-GG-C---------A--G

TGATG-AAT-A--A-A-AA--A-TT-TA-TTGCC-GATCCATT-GGGACGAA----TCT------GGG----

-------------GCTCAAATCTCAG-C----------------CAAAA-CTCAAC-AGACACA---C----

------------------TGA--C----GAA-CGTCTGG----------T--A--AAAATTTCAGA-CCA--

--AAATACCC-------AAGGA-GT-----AAGGC---GT-----AGTG------AG-TCCCAAA-CCGAGA

GTGAA-------------------------------------------------------------------

------------------------------------------------------------------------

--------------C--AAAACT--------------------------GG-----------------TTTT

C----------------------CGAAAACAAAA--C--GTCCTGGC-----TTTTCTTCCC----------

--------------CGTATA--TTCC-----------TTTTGTGA---------------------------

-------------------------TTT--CTTTAC-GGAC--GTG-CCT-CC-TTACTT--GGGTGC--CA

AA-----------AACTA-A----AGAAAGT-AATTGTG-C-------------GAT---------------

--TTTTTTCA------------------------------GCGCAAT-A-C-G-GA-CCC-AGA-----AT-

AAAATTT-TCAGAAA--GTAGG------------GCGAAATTT--CACA-A-GTT-TTGA--TAGAG-----

---------GAT-AGTCT-------------------TGG-ATTACACA----AAATTTCTGAAAAATTC

>Pv01Sk00360/1-540 Pv01Sk00360 undefined product 448903:449442 forward

CTCCGGAAATAG-----TTCTTTCCTG--AAATTCCACGT-AAG-AA--T-------CT---CCACTG-AA-

TTTGGG-AC----------AAAACGCC-AC-AA-AT--TTAAGG--TC------------------------

-----------------------------------------AAAC-G--GATGAG---TATTCAC-------

---CCA-----CGAAAAAAA---TC-AAACAGTT-TC--------------------------GCA-CAC--

AGAC--G-AA-CCT------------TCC--T-TTC-------A-G--CC-C-T-GG-C---------A--G

TGATG-AAT-A--A-A-AA--A-TT-TA-TTGCC-GATCCATT-GGGACGAA----TCT------GGG----

-------------GCTCAAATCACAG-C----------------CAAAA-CTCAAC-AGACACA---C----

------------------TGA--C----GAG-CGTCTGG----------T--A--AAAATTTCAGA-CCA--

--AAATACCC-------AAGGA-GT-----AAGGC---GT-----AGTG------AG-TCCCAGA-CCAAGA

GTGAA-------------------------------------------------------------------

------------------------------------------------------------------------

--------------C--AAAACT--------------------------GG-----------------TTTT

A----------------------CGAAAACAAAA--C--GTCCTGGC-----TTTTCTTCCC----------

--------------CGTATC--TTCC-----------TTTTGTGA---------------------------

-------------------------TTT--CTTTAC-GGAC--GTG-CCT-CC-TTACCT--GGGTGC--AA

AC-----------AACAT-A----TGAAAGT-ACTTGTG-T-------------GAA---------------

--TTTTTTCA------------------------------GCGCAAT-A-C-G-GA-CCC-AGA-----AT-

AAAATT--GCAGAAA--GTAGG------------GCGAAATTT--CACA-A-ATT-TTGG--TAAAG-----

---------GAT-AGCCT-------------------TGG-TTTGCACA----AAATTTCTGAAAAATTC

>Pv01Sk00370/1-537 Pv01Sk00370 undefined product 449443:449979 forward

-TCCGAAAATAG-----TTCTTTCTTG--AAATTCCACGT-AAG-AA--T-------CT---CCATTG-AA-

TTTGGG-AC----------AAAACGCC-AC-AA-AT--TTCAGG--TC------------------------

-----------------------------------------AAAC-G--GATGAG---TATTCAC-------

---CCA-----CGAAAAAAA---TC-AAACAGTT-TC--------------------------GCA-CAC--

AGAC--G-AG-CTT------------TCC--T-TTC-------A-G--CC-C-T-GG-C---------A--G

TGATG-AAT-A--A-A-AA--A-TT-TA-TTGCC-GATCCCGT-GGGACGAA----TCT------GGG----

-------------GCTCAAATCTTAG-C----------------CAAGA-CTCAAC---ACACA---C----

------------------TGA--C----GAA-CGTCTGG----------T--A--AAAATTTCAGA-CCA--

--AAATACCC-------AAGGA-GT-----AAGGC---GT-----AGTG------AG-TCCCAGA-CCGAGA

GTGAA-------------------------------------------------------------------

------------------------------------------------------------------------

--------------C--AAAATT--------------------------GG-----------------TTTT

C----------------------CGAAAACAAAA--C--GTCCTGGC-----TTTTCTTCCC----------

--------------CGTATC--TTCC-----------TTTTGTGA---------------------------

-------------------------TTT--CTTTAC-GGAC--GTG-CCT-CC-TTACCT--GAGTGC--AA

AC-----------AACAT-A----TGAAAGT-ACTTGTG-C-------------GAA---------------

--TTTTTTCA------------------------------GCGCAAT-A-C-G-GA-CCC-AGA-----AT-

AAAAGT--GCAGAAA--GTAGG------------GCGAAATTT--CACA-A-GTT-TTGG--TAGAG-----

---------GAT-AGTCT-------------------TGG-TTTGCACA----AAATTTCTGAAAAATTC

>Pv01Sk00380/1-541 Pv01Sk00380 undefined product 449979:450519 forward

CTCCGGAAATAG-----TTCTTTCCTG--AAATTCCACGT-AAG-AA--T-------CT---CCACTG-AA-

TTTGGG-AC----------AAAACGCC-AC-AA-AT--TTCAGG--TC------------------------

-----------------------------------------AAAC-G--GATGAG---TATTCAC-------

---CCA-----CGAAAAAAA---TC-AAACAGTT-TC--------------------------GCA-CAC--

AGAC--G-AA-CCT------------TCC--T-TTT-------ATG--CC-C-T-GG-T---------A--G

TGATG-AAT-A--A-A-AA--A-TT-TA-TTGTC-GATCCCGT-GGGAGGAA----TCT------CGG----

-------------GCTCAAATCTCAG-C----------------CAAAA-CTCAAC-AGACACA---C----

------------------TGA--C----GAA-CGTCTGG----------T--A--AAAATTTCAGA-CCA--

--AAATACCC-------AAGGA-GT-----AAGGC---GT-----AGTG------AG-TCCCAGA-CCGAGA

GTGAA-------------------------------------------------------------------

------------------------------------------------------------------------

--------------C--AAAACT--------------------------GG-----------------TTTT

T----------------------CGAAAAAGAAA--C--GTCCTGGT-----TTTTCTTCCC----------

--------------CGTATC--TTCC-----------TTTTGTGG---------------------------

-------------------------TTT--CTCTAC-GGAC--GTG-CCT-CC-TTACCT--GGGTGC--AA

AC-----------AACAT-A----TGAAAGT-ACTTGTG-C-------------GAA---------------

--TTTTTGCA------------------------------GTGCAAT-A-C-G-GA-CCC-AGA-----AT-

AAAATT--GCAGAAA--GTAGG------------GCGAAATTT--CACA-A-GTT-TTGG--TAGAG-----

---------GAT-AGTCT-------------------AGGTTTTTCACA----AAATTTGTGAAAAATT-

>Pv01Sk00390/1-536 Pv01Sk00390 undefined product 450523:451058 forward

---CGGAAATAG-----TTCTTTCCTG--AAATTCCACGT-ATG-AA--T-------CT---CCACTG-AA-

TTTGGG-AA----------AAAACGCC-AC-AA-AT--ATAAGG--TC------------------------

-----------------------------------------AAAC-G--GACGAG---TATTCAC-------

---CCA-----CGAAAAAAA---TC-AAACATTT-TC--------------------------GCA-CAC--

AGAC--G-AA-CCT------------TCC--T-TTC-------A-G--CC-G-T-GG-C---------A--C

TGATG-AAT-A--A-A-AA--A-TT-TA-TTGCC-GATCCATA-GGGACGAA----TCT------GGG----

-------------GCTCAAATATCAG-T----------------CAAAA-CTCAAC-AGACACA---C----

------------------TGA--C----GAA-CGTCTGG----------T-----AAATTTTCAGA-CCA--

--AAATACCC-------AAGGA-GT-----AAGGC---GT-----AGTG------AG-TGCCAAA-CCGAGA

GTGAA-------------------------------------------------------------------

------------------------------------------------------------------------

--------------A--AAAACT--------------------------GG-----------------TTTT

C----------------------CGAAAACAAAA--C--GTCTTGGC-----TTTTCATCTC----------

--------------CGTATC--TTCC-----------TTTTGTGA---------------------------

-------------------------TTT--CTTTAC-GGAC--GTG-CCT-CC-TTACCT--GGGTGC--AA

AC-----------AACAT-A----TGAAAGT-ACTTGTG-C-------------GAA---------------

--TTTTTTCA------------------------------GCGCCAT-A-C-G-GA-CCC-AGA-----AT-

AAAATT--GCAGAAA--GTAGG------------GCGAAATTT--CACA-A-GTT-TTGG--TAGAG-----

---------GAT-AGCCT-------------------TGT-TATGCACA----AAATTTTTGAAAAATTC

>Pv01Sk00400/1-541 Pv01Sk00400 undefined product 451058:451598 forward

CTCCGGAAATAG-----TTCTTTCCTG--AAATTCCACGT-AAG-AA--T-------CT---CCACTG-AA-

TTTGGG-AC----------AAAACGCC-AC-AA-AT--TTAAGG--TC------------------------

-----------------------------------------AAAC-G--GACGAG---TATTCAC-------

---CCA-----CGAAAAAAA---TC-AAACAGTT-TC--------------------------GCA-CAC--

AGAC--G-AA-CCT------------TCC--T-TTC-------A-G--CC-A-T-GG-C---------A--G

TGATG-AAT-A--A-A-AA--A-TA-TA-TTGCC-GATCCTGT-GGGACGAA----TTT------GGG----

-------------GCTCAAATCTCAG-C----------------CAAAA-CTCAAC-AGACACA---C----

------------------TGA--C----GAG-CGTCTGG----------T--A--AAAATTTCAGA-CCA--

--AAATACCC-------AAGGA-GT-----AAGGC---GT-----AGTG------AG-TCCCGGA-CCGAGA

GTGAA-------------------------------------------------------------------

------------------------------------------------------------------------

--------------C--AAAACT--------------------------GG-----------------TTTT

C----------------------CGAAAACAAAA--C--GTCCTGGC-----TTTTCTTCCC----------

--------------CGTATC--TTCCT----------TTTTGTGA---------------------------

-------------------------TTT--CTTTAC-GGAC--GTG-CCT-CC-TTACCT--GGGTGC--AA

AC-----------AACAT-A----TGAAAGT-ACTTGTG-C-------------GAG---------------

--TTTTTTCA------------------------------GCGCAAT-A-C-G-GA-CCA-ATA-----AT-

AAAATT--GCAGAAA--GTAGG------------GCGAAATTT--CACA-A-GTT-TTGG--TAGAT-----

---------GAT-AGCCT-------------------TGG-TTTGCACA----AAATTTCTTAAAAATTC

>Pv01Sk00410/1-539 Pv01Sk00410 undefined product 451598:452136 forward

CTCCCGAAATAG-----TTTTTTCCTA--TAATCCCAAGT-AAG-AA--T-------CT---CCACTG-AA-

TTGGGG-AC----------AAAACGCG-AC-AA-AT--TTCATG--TC------------------------

-----------------------------------------AAAC-G--GATGAG---TATTCAC-------

---TCA-----CGAAAAATA---TC-AAACAGTT-TC--------------------------AAA-CAC--

AAAC--G-AA-CCT------------TCC--T-CTC-------G-G--CC-C-C-GA-C---------A--G

TGATG-GAT-A--A-A-AA--A-TT-TA-TTGTC-AATCATGT-GGGACGAA----TCT------GGG----

-------------GCTCAAATCTCAG-C----------------AAAAA-CTCAGT-AGACACA---G----

------------------TGA--C----GAA-CGTTAGG----------T--A--AAAATTTCAGA-CCA--

--AAATACCC-------AAGGA-GT-----AACGC---GT-----AGTA------AG-TCCCACA-CCGAGA

GTGAA-------------------------------------------------------------------

------------------------------------------------------------------------

--------------C--AAAACT--------------------------GG-----------------TTTT

C----------------------CGAAAACAAAA--C--GTCCTGGC-----TTTTCTTCCC----------

--------------CGTATC--TTCC-----------TTTTGTGA---------------------------

-------------------------TTT--CTTTAC-GGAC--GTG-CCT-CC-TTACAT--GGGTGC--AA

AC-----------AACAT-A----TGAAAGT-ACTTGTG-C-------------GAA---------------

--TTTTTTCA------------------------------GCAAAAT-A-C-A-GA-TCC-AGA-----AT-

AAAATT--GCAGAAA--GTAGG------------GCGAAATTT--CACA-A-ATT--TGG--TAGAT-----

---------GAT-AGCCT-------------------TGG-TTTGCACA----AAATTTCTGAAAAATTC

>Pv01Sk00420/1-542 Pv01Sk00420 undefined product 452136:452677 forward

CTCCGGAAATAG-----TTTTTTCCTG--AAATTCCACAT-AAG-AA--T-------CT---CCACTG-AA-

TTTGGG-AC----------AAAACGCC-AC-AA-AT--TTCATA--TC------------------------

-----------------------------------------AAAC-G--GATGAG---TATTCAC-------

---CCA-----CGAAAAAAA---TC-AAACAGTT-TC--------------------------GCA-CAC--

AGAA--G-AA-CCT------------TCC--T-TTC-------A-G--CC-C-T-GG-C---------A--G

TGATG-AAT-A--A-A-AA--A-TT-TA-TTGCC-GATCCCGT-GGGACGAA----TCT------GGG----

-------------GCTCAAATCTCAG-C----------------CAAAA-CTCAAC-AGACACA---C----

------------------TGA--C----GAA-CGTCTAG----------T--A--AAAATTTCAGA-CCA--

--AAATACCA-------AAGGA-GT-----AAGGC---GT-----AGCG------AG-TCCCAGA-CCGAGA

GTGAA-------------------------------------------------------------------

------------------------------------------------------------------------

--------------C--AAAACT--------------------------GG-----------------TTTT

C----------------------CGAAAACAAAA--C--GTCCTGGC-----TTTTCTTCCC----------

--------------CGTATC--TTCC-----------TTTTGTGA---------------------------

-------------------------TTT--CTTTAC-GGAC--GTG-CCT-CC-TTACCT--GGGTGC--AA

AC-----------AACAT-A----TGAAAGT-ACTTGTG-C-------------GAG---------------

--TTTTTTCA------------------------------GCGCAAT-A-C-G-GA-CCC-ATA-----AT-

AAAATT--GCAGAAA--GTAGG------------GCGAAATTT--CACA-A-GTT-TTGG--TAGATGA---

---------TAT-AGCCT-------------------TGG-TTTGCACA----AAATTTCTGAAAAATTC

>Pv01Sk00430/1-541 Pv01Sk00430 undefined product 453269:453809 forward

CTCCGGAAATAG-----TTTTTTCCTG--AAATTCCACGT-AAG-AA--T-------CT---CCACTG-AA-

TTTGGG-AC----------AAAACGCC-AC-AA-AT--TTCAGG--TC------------------------

-----------------------------------------AAAC-G--GACGAG---TATTCAC-------

---CCA-----CGAAAAGAAA--TC-AAACAGTT-TC--------------------------GCA-CAA--

AGAC--G-AA-CCT------------TCC--T-TTC-------A-G--CC-C-T-GG-C---------A--G

TGATG-AAT-A--A-A-AA--A-TT-TA-TTGTC-GATCCCGT-GGGACGAA----TCT------GGG----

-------------GCTCAAATCTCAA-T----------------CAAAA-CTCAAC-AGACACA---C----

------------------TGA--C----GAA-CGTCTGG----------T--A--AAAATTTCAGA-CCT--

--AAATACCC-------AAGGA-GT-----AAGGC---GT-----AGTG------AG-TCTGAGA-CCGAGA

GTGAA-------------------------------------------------------------------

------------------------------------------------------------------------

--------------C--AAAACT--------------------------GG-----------------TTTT

C----------------------CGAAAACAAAA--T--GTCCTGGC-----TTTTCTTCCC----------

--------------CGTATC--TTGC-----------TTTTGTGA---------------------------

-------------------------TTT--CTTTAC-AGAC--GTG-CCT-CC-TTACCT--GGGTGC--AA

AC-----------AACAT-A----TGAAAGT-ACTTGTG-C-------------GAA---------------

--TATTTTCA------------------------------GCGCAAT-A-C-G-GA-CCC-AGA-----AT-

AAAATT--GCAGAAA--GTAGG------------GCGAAATTT--CACA-A-GTT-TTGG--TAGAG-----

---------GAT-AGCCT-------------------TGG-TTTGCACA----AAATTTCTGAAAAATTC

>Pv01Sk00440/1-538 Pv01Sk00440 undefined product 453809:454346 forward

CTCCGGAAATAG-----TTTTTTCCAG--AAATTCCACGT-AAA-GA--T-------CT---CCACTG-AA-

TTTGGG-AC----------AAAACGCC-AC-AA-AT--TTCAGG--TC------------------------

-----------------------------------------AAAC-G--GACGAG---TATTCAC-------

---CCA-----CGAAAAAAA---TC-AAACAGTT-TC--------------------------GCA-CAC--

AGAC--G-AA-CCT------------TCC--T-TTC-------A-G--CC-C-T-GG-C---------A--G

TGATG-AAT-C--A-A-AA--A-TT-TA-TTGCC-GATCTCGT-GGGACGAA----TCT------GGG----

-------------GCTCAAATCTCAG-C----------------CAAAA-CTCAAC-AGACACA---C----

------------------TGA--C----GAA-CGTCTGG----------T--A--AAAATTTCAGA-CCA--

--AAATACCA-------AAGGA-GT-----AAGGC---GT-----AGTG------AG-TCCCGGA-CCGAGA

GTAAA-------------------------------------------------------------------

------------------------------------------------------------------------

--------------C--AAAACT--------------------------GG-----------------TTTT

C----------------------CGAAAACAAAA--C--GTCCTGGC-----TTTTCTTCCC----------

--------------CGTATC--TCCC-----------TTTTGTGA---------------------------

-------------------------TTT--CTTTAC-GGAC--GTG-CCT-CC-TTACCT--GGGTGC--AA

AC-----------AACAT-A----TGAAAGT-ACTTGTG-C-------------GAA---------------

--TTTTTTCA------------------------------GCGCAAT-A-C-G-GA-CCC-AGA-----AT-

AAAATG--GCAGAAA--GTAGG------------GCGAAATTT--CAC--A-GTT-TTGG--TAGAT-----

---------GAT-AGCCT-------------------TGG-TTTGCACA----AAATTTCTCAAAAATT-

>Pv01Sk00450/1-542 Pv01Sk00450 undefined product 454349:454890 forward

--CTGGAAATAG-----TTTTTTCCTG--AAATTCCATGT-AAA-AA--T-------CT---CCACTG-AA-

TTTGGG-AC----------AAAACGCC-AC-AA-AT--TTCAGG--TC------------------------

-----------------------------------------AAAC-G--AATCAG---TATTCAC-------

---CCA-----CGAAAAAAA---TC-AAACAGTT-TC--------------------------GCA-CAC--

AGAC--G-AA-CCT------------TCC--T-TTC-------A-G--CC-C-T-GC-C---------A--G

TGATG-AAT-A--A-A-AA--A-TT-TA-TTGTC-AATCTTGT-GGGACGAA----TTT------GGG----

-------------GCTCAAATCTCAG-C----------------CAAAA-CTCAAC-AGACACA---C----

------------------TGA--C----GAA-CGTCTGG----------T--A--AAAGCTTCATA-CCA--

--AAAGACCC-------AAGGA-AT-----AAGGA---GT-----AGTA------AA-TCCTAGA-CCGAGA

GTGAA-------------------------------------------------------------------

------------------------------------------------------------------------

--------------G--AAAACT--------------------------GG-----------------TTTT

C----------------------CAAAAACAAAA--C--TTCCTGGT-----TTTTCTTCTC----------

--------------TATATC--TCCT-----------TTTTGTGA---------------------------

-------------------------TTT--GTTTAT-GGAT--GTG-CCT-CC-TTAGCA--GGGTGC--AA

AC-----------AACAG-A----TGAAAGT-ACTTATG-T-------------GTATGTTTTT-------G

GAATTTTTGA------------------------------ACGCAGT-A-T-G-GA-CCC-ATT-----ATA

AAAATT--GTTGAAA--GTAGG------------GCAAAATTT--CACA-A-ATT-TT--------------

---------GGT-AGCCT-------------------TGG-TTTGCACA----AAATTTCTGAAAAATTC

>Pv01Sk00460/1-539 Pv01Sk00460 undefined product 575994:576532 forward

CTCCTGAAATAG-----TTTTTTTCTG--AAATTCCACGT-AAG-AA--T-------CT---CGAAAG-AA-

TTTAAG-AC----------AAAACACG-AC-AA-GT--TTCAGA--TC------------------------

-----------------------------------------AAAC-G--GGTGAG---TATTCAC-------

---CTA-----AGAAAAGAA---TC-AAACAGTT-TC--------------------------AAA-CAC--

AAAA--G-AA-CCT------------TCC--T-TAC-------G-G--CC-T-T-GG-C---------A--G

TGATG-AAT-A--A-A-AA--A-AT-TA-TTGCA-AATCTTGT-GGGACGAA----TTT------GGG----

-------------GCTCAAACTTCAG-C----------------CAAAA-CTCAAT-AGACACA---A----

------------------TGA--C----GAA-TGTGTGG----------T--A--AAAATTTCAGA-CCA--

--AAATACCC-------AAGGA-GT-----AAGGC---GT-----AGTA------AG-TCCGAGA-CCGAGA

GTGAA-------------------------------------------------------------------

------------------------------------------------------------------------

--------------C--AAAACT--------------------------GG-----------------TTTT

C----------------------TGAAAACA-AA--C--GTCCTGAC-----TTTACTTCCC----------

--------------CGTATA--TTCT-----------TTTTGTGA---------------------------

-------------------------TTT--GTTTAT-GGAC--GTG-CCT-CC-TTATCT--GGGTGC--AA

AC-----------ATCAT-A----TGAAAGA-ACTTGTG-T-------------AAA---------------

--TTTTTTCA------------------------------ACGCAAT-A-C-G-GA-CCC-AGA-----AT-

AAAATC--ACAGAAA--GTAGG------------GCGAAATTT--CACA-T-GTT-TTGG--TAAAG-----

---------GAT-AGCCT-------------------TGA-CTTGCACA----AAATTTCTTAAAAATTC

>Pv01Sk00470/1-536 Pv01Sk00470 undefined product 576532:577067 forward

CTCCCGAAATAG-----TTTTTTTCCT--GAAATTCCACT-AAG-AA--T-------CT---CCACTG-AA-

TTTGGG-AC----------AAAACGAG-AC-AA-AT--TTCAGG--TC------------------------

-----------------------------------------AAAC-G--GATGAG---TATTCAC-------

---CCA-----CGAAAAAAA---TC-AAACAGTT-TC--------------------------AAA-CGA--

AAAT--G-AA-CAT------------TTC--T-TTC-------A-G--AC-T-T-GA-C---------A--A

TGATG-AAT-A--A-A-AA--A-TT-TA-TTGCC-AATCTTCT-GGGACGAA----TTT------GGG----

-------------GCTTAAATCTCAG-C----------------C-CAA-ACTCAACAGACACA---G----

------------------TGA--C----AAA-CGTCTGG----------T-AA--AAATTTC-AGA-CCA--

--AAATACCA-------AAGGA-GA-----AAGGC---GT-----AGTA------AG-TCCCAAA-CCGAGA

GTGAA-------------------------------------------------------------------

------------------------------------------------------------------------

--------------C--AAA------------------------------------------------TTTT

C----------------------CGAAAACAAAA--C--GTCTTGGA-----TTATCTTCCC----------

--------------CATATC--TTCT-----------TTTTGTGA---------------------------

-------------------------TTT--GTTTAT-GGAC--GTG-CAT-CC-TTACCT--GGGTGC--AA

AC-----------AACAT-A----TGAATGT-ACTTGTG-T-------------GAA---------------

--TTTTTGCA------------------------------GCACAAT-A-C-G-GA-CCC-AGA-----AT-

AAAATT--GCAGAAA--GTAGG------------GCCAAATTT--CACA-A-GTT-TTGG--TAGAG-----

---------GAT-AGCCTT------------------TGG-ATTGCACA----AAATTTCTGAAACATTC

>Pv01Sk00480/1-539 Pv01Sk00480 undefined product 577067:577605 forward

CTCCCGAAATGG-----TTTTTTCCCG--AAATTCCATGT-AAG-AA--T-------CT---CCACTG-AA-

TTTAGG-AC----------AAAACGCT-AC-AA-AT--TTCAGG--TC------------------------

-----------------------------------------AAAC-G--GATGAG---TATTCAC-------

---CCA-----CGAAAAAAA---TC-AAACAGTT-TC--------------------------GCA-CAC--

GAAC--G-AA-CCC------------TCC--T-TTC-------A-G--CC-C-T-GG-C---------A--G

TGATG-AAT-A--A-A-AA--A-TT-TA-TTGTC-AATCTTGT-GGGACGCA----TCT------GGG----

-------------GCTCAAATTTCAT-C----------------C-GAA-ACTCAACAGACACA---G----

------------------TGA--C----GAA-CGTCTGG----------T-AA--AAATTTC-AGA-CCA--

--AAATACCC-------AAGGA-GT-----AAGGC---GT-----AGTA------AG-TCCCAGA-CCGGGA

GCAAA-------------------------------------------------------------------

------------------------------------------------------------------------

--------------C--AAAACT--------------------------GG-----------------TTTT

C----------------------CGAAAGCAAAA--C--GTCCTGGC-----TTTTCTTCCC----------

--------------CGTATC--TTCT-----------TTTTGTGA---------------------------

-------------------------TTT--GTTTAT-GGAC--GTG-CCT-CC-TTACCT--GGGTGC--AA

AC-----------AACAT-A----GGAAAGT-ACTTGTG-T-------------GAA---------------

---TTTTTCA------------------------------GTGCAAT-A-C-G-GA-CCC-AGA-----AC-

AAAATT--GCAGAAA--GTAGG------------GCCAAATTT--CACA-A-GTT-TTGG--TAGAG-----

---------GAT-AGCCT-------------------TGG-TTTTCACA----AAATTTCTGAAAAATTC

>Pv01Sk00490/1-538 Pv01Sk00490 undefined product 577607:578144 forward

--CCCGAAATGG-----TTTTTTCCTG--AAATTCCACGT-AAG-AA--T-------CT---CCACTG-AA-

TTTGGG-AC----------AAAACGCG-AC-AA-AT--TTCAGG--TA------------------------

-----------------------------------------AAAC-G--GATGAG---TATTCAC-------

---CCA-----CGAAAAAAA---TC-AAACAGTT-TC--------------------------ACA-CAC--

GAAC--G-AC-CCC------------TCC--T-TTC-------A-G--CC-C-T-GG-C---------A--G

TGATG-AAT-A--A-A-AA--A-TT-TA-TTGCC-AATCTTGT-GGGACGCA----TCT------GGG----

-------------GCTTAAATCTCAT-C----------------C-GAA-ACTCAGCAGACACA---G----

------------------TGA--C----GAA-CGTCTGG----------T-AA--AAATTTC-AAA-CCG--

--AAATACCC-------AAGGA-GT-----AAGGC---GT-----AGTA------AG-TCCCAGA-CCGGGA

GCGAA-------------------------------------------------------------------

------------------------------------------------------------------------

--------------C--AAAACT--------------------------GG-----------------TTTT

C----------------------CGAAAACAAAA--C--GTCCTGGC-----TTTTCTTCCC----------

--------------CGTATC--TTCT-----------TTTTGTGA---------------------------

-------------------------TTT--GTTTCT-GGAC--GTG-TCT-CC-TTACCT--GGGTGA--AA

AC-----------AACAT-A----GGAAAGT-ACCTGTG-T-------------TTA---------------

--TTTTTTCA------------------------------CCGCAAT-A-C-G-GA-CCC-AGA-----AC-

AAAATT--GCAGAAA--GTAGG------------GCCAAGTTT--CACA-A-GTT-TTGG--TAGAG-----

---------GAT-AGCCT-------------------TGC-TTTGCACA----AAATTTCTGAAAAATTC

>Pv01Sk00500/1-536 Pv01Sk00500 undefined product 578144:578679 forward

CTCCCGAAATGG-----GTTTTTCCTG--AAATTCCACGT-AAG-AA--T-------CT---CCACTG-AA-

TTTGGG-AC----------AAAACGCG-AC-AA-AT--TTCAGG--TC------------------------

-----------------------------------------AAAC-G--GATGAG---TATTCAC-------

---CCA-----CGAAAAAAA---TC-AAACAATT-TC--------------------------GCA-CAC--

GAAC--G-AA-CCC------------TCC--T-TTC-------A-G--CC-C-T-GG-C---------A--G

TGATG-AAT-A--A-A-AA--A-TT-TA-TTGCC-AATCTTGT-GGGACGCA----TCT------GGG----

-------------GCTCAAATCTCAT-C----------------C-GAA-ACTCAGCAGACAAA---G----

------------------TGA--C----GAA-CGTCTGG----------T-AA--AAATTTC-AGA-CCG--

--AAATACCC-------AAGGA-GT-----AAGGC---GT-----AGTA------AG-TCCCAGA-CCGAGA

GCGAA-------------------------------------------------------------------

------------------------------------------------------------------------

--------------C--AAA--C--------------------------TG-----------------GTTT

A----------------------CCAAAACAAAC--C--TCCTTGC-------TTTCTTCCC----------

--------------CGTATC--TTCT-----------TTTTGTGA---------------------------

-------------------------TTT--TTTTAT-GGAC--GTG-CCT-CC-TTACCT--GGTTGC--AA

AC-----------AACAT-A----GGAAAGT-ACCTGTG-T-------------GAA---------------

--TTTCTTCT------------------------------GCGCAAT-A-C-G-GA-CCA-AGA-----AA-

ATTATT--GCAGAAA--GTAGG------------GCCAAATTT--CACA-A-GTT-TTGG--TAGAG-----

---------CAT-AGCCT-------------------TGG-TTTGCACA----AAATTTCTGAAAAATTC

>Pv01Sk00510/1-537 Pv01Sk00510 undefined product 578680:579216 forward

-TTCCAAAATGG-----TTTTTTCTCG--AAATTCCACGT-AAG-AA--T-------CT---CCACTG-AA-

TTTGGG-AC----------AAAACGCG-AC-AA-AT--TTCAGG--TC------------------------

-----------------------------------------AAAC-G--GATGAG---TATTCCC-------

---ACA-----CGAAAATAA---TC-AAACAGTT-TC--------------------------GCA-CAC--

GAAC--A-AA-CCC------------GCC--T-TTC-------A-G--CC-C-T-GG-C---------A--T

TCATG-AAT-A--A-A-AA--A-TT-TA-TTGCC-AATCTTGT-GGGACGAA----TCT------GGT----

-------------GCTAAAATCTCAT-C----------------C-GAA-ACTCAACAGACACA---G----

------------------TGA--C----GAA-CGTCTAG----------T-AA--AAATTTC-AGA-CCA--

--AAATACCC-------AAGGA-GT-----AAGGC---GT-----AGTA------AG-TCCCAGA-CCGAGA

GCGAA-------------------------------------------------------------------

------------------------------------------------------------------------

--------------C--AAAATT--------------------------GG-----------------TTTT

C----------------------CGAAAACAAAA--C--GTCCTGGC-----TTTTCTTCCC----------

--------------CGTATC--TTCT-----------TTTTGTGA---------------------------

-------------------------TTT--GTTTAT-GGAC--GTG-CCT-CC-TTACCT--GGGTGC--AA

AC-----------AACAT-A----GGAAAGT-ACCTGTG-T-------------GAA---------------

---TTTTTCA------------------------------GCGCAAT-A-C-G-GA-CCC-AGA-----AC-

AAAATT--GCAGAAA--GTA-G------------GCCAAATTT--CACA-A-GTT-TTGG--TAGAG-----

---------GAT-AGCCT-------------------TGG-TTTTCACA----AAATTTCTGAAAAATTC

>Pv01Sk00520/1-540 Pv01Sk00520 undefined product 579763:580302 forward

CTCCCGAAATGC-----TTTTTTCTTG--AAATTCCACGT-AAG-AA--T-------CT---TCACTG-AA-

TTTGGG-AC----------AAAACGCG-AC-AA-AT--TTCAGG--TC------------------------

-----------------------------------------AAAC-G--GATGAG---TATTCAC-------

---CCA-----CGAAAAAAA---TC-AAACAGTT-TC--------------------------GCA-CAC--

GAAC--G-AA-CCC------------TCC--T-TTC-------A-G--CC-C-T-GG-C---------A--G

TGATG-AAT-A--A-A-AA--A-TT-TA-TTGCC-AATCTTGT-GGGACGCA----TCT------GGG----

-------------GCTCAAATCTCAT-C----------------C-GAA-ACTCAACAGACACA---G----

------------------TGA--C----GAA-CGTCTGG----------T-AA--AAATTTC-AGA-CCA--

--AAATACCC-------AAGGA-GT-----AAGGC---AT-----AGTA------AG-TCCCAGA-CCGAGA

GCGAA-------------------------------------------------------------------

------------------------------------------------------------------------

--------------C--AAAACT--------------------------GG-----------------TTTT

C----------------------CGAAAACAAAA--C--GTCCTGGC-----TTTTCTTCCC----------

--------------CGTATC--TTCT-----------TTTTGTGA---------------------------

-------------------------TTT--GTTTGT-GGAC--GTC-CCT-CC-TTACCT--GGGTGC--AA

AC-----------AACAT-A----GGAAAGT-ACTTGTG-T-------------GGA---------------

--TTTTTTCA------------------------------GCGCAAT-A-C-G-GA-CCC-AGA-----AT-

AAAATT--GCAGAAA--GTAGG------------GCCAAATTT--CACA-A-GTG-TTGG--TAGAG-----

---------GAT-AGCCT-------------------TGG-TTTGCACA----AAATTTCAGAAAAATTC

>Pv01Sk00570/1-551 Pv01Sk00570 undefined product 713751:714301 reverse

CTCCCAAAATAA-----CTTTTTGTTG--AAATTACACGT-AAG-AA--T-------CT---CGACTG-AA-

TTTGGG-AC----------AAAATGCA-AC-AT-GT--TTTAGG--TC------------------------

-----------------------------------------AAAC-G--GATGAG---TATTCAC-------

---TTA-----GGAAAAAAA---TC-AAACAGTT-TC--------------------------ACA-CAC--

AAAC--C-AA-CCT------------TCA--T-TTC-------T-G--CT-C-T-GG-C---------A--G

TGATG-AGT-A--A-A-AA--A-TT-TA-TTGCA-AATCTTGT-GGAACGAA----ATT------GGG----

-------------GCTCAAATCTCAG-C----------------CAAAA--CTCAATACACACA---A----

------------------TGA--C----GAA-TCTCTGA----------T--A-AAAATTTC-AGA-CCA--

--AAAGACCC-------AAGGA-AT-----AAGGC---GT-----AGTA------AG-TTCCAGA-CCGAGT

G---A-------------------------------------------------------------------

------------------------------------------------------------------------

--------------A--GAAAAC--------------------------CG-----------------TTTT

C----------------------CGAAAAATAAA--T--GTCCTTAC-----TTTTCTTCCC----------

--------------TATATC--TGTTTTTT-------TTTTGTGA---------------------------

-------------------------TTT--GTTTAT-AGAC--GTA-CCT-CC-TTAGCA--AGGTGC--AA

GC-----------AACAT-A----TGAAGGT-ACTTGTA-T-------------TAATGTTTTC-------G

GAAATTTTCA------------------------------ACGCAAT-A-T-G-GA-CCC-ATA-----AT-

AAATTT--GTAGAGT--GTAGG------------GCAAAATTT--CATA-A-ATT-TTGG--TAGAG-----

---------GAT-AGCCT-------------------TGG-TTTGCACA----AAATTTCTGAAAAATTC

>Pv01Sk00610/1-535 Pv01Sk00610 undefined product 715926:716460 reverse

CTCCCGAAATAA-----CTCTTTTACG--AAATTCCACGT-AAG-AA--T-------CT---CGACTG-AA-

TTTGGG-AC----------AAAACGCA-AC-AA-GT--TTTAGG--TC------------------------

-----------------------------------------AAAC-G--GATGAG---TATTCAC-------

---CTA-----GGAAAAAAA---TC-AAATAGTT-TC--------------------------ACA-CAC--

AAAC--G-AA-TAA------------TCC--T-ATC-------A-G--CT-C-T-GT-C---------A--G

TGATG-AAT-A--A-A-AA--A-TT-TA-TTGCA-AATCTTGT-GCGACGAA----TTT------GGG----

-------------ACTCAAATCTCAG-C----------------CAAAA--TTCAATATACACA---A----

------------------TGA--C----GAA-TGTCTGG----------T--A-AAAATTTC-ATA-CCG--

--AGAGGCTC-------AAGGA-AT-----AAGGT---AT-----AGTA------AA-TCCTGGA-CAGAGA

GAGAA-------------------------------------------------------------------

------------------------------------------------------------------------

--------------G--AACACC--------------------------GG-----------------TTTT

C----------------------CGAAAACAAAA--A--GTCATGGA-----GTTTCTTCCC----------

--------------TATATC--TCCTT----------TTTTGTGA---------------------------

-------------------------TTT--GTTTAT-GGAC--ATG-CCT-CC-TTAGCA--GGGTGT--AA

AC-----------AACAT-A----TGAAAGT-ACTTGTG-T-------------GAATGTTTTC-------A

GAATTTTTCA------------------------------ACGCAAT-A-T-T-GA-CCC-AAA-----AT-

AAAATT--GCATAAA--GTAGG------------GAGATATTT--CACA-A-ATT-TCGG--TAGAT-----

---------GAT-A----------------------------------------AATTTTTGAAAAATTC

>Pv01Sk00620/1-547 Pv01Sk00620 undefined product 716460:717006 reverse

-TCCCGAAATAC-----TTTTTTCCTG--AAATTCCACGT-AAG-AA--T-------CT---TGACTG-AA-

TTTGTG--T----------AAAATACA-GC-AA-GT--TTCAGG--TC------------------------

-----------------------------------------AGAC-G--GATAAG---TATTCCC-------

---CAA-----GGAAAAAAA---TC-AAACTGTT-TC--------------------------ACA-CAC--

AAAC--G-AA-CCT------------TCC--T-TTC-------A-G--CC-C-T-GG-C---------A--G

TAATG-AAT-A--A-A-AC--A-TT-TA-TTGCA-AATCTTGT-GGGACGTA----TTT------GGG----

-------------GCTCAAATCTCTG-C----------------CAAAA--CTCAATAAGCACA---A----

------------------TGA--A----GAA-AGTCTTG----------T--A-AAAATTTC-AGA-TCA--

--AAAGACAC-------AAGGA-AT-----CAGGC---GT-----AGTA------AA-TACCAAA-CCGAGA

GTGAA-------------------------------------------------------------------

------------------------------------------------------------------------

--------------G--AAAACC--------------------------GA-----------------ATTT

C----------------------CGAAAACAAAA--C--GCGTTGGC-----TTTTCTTCCC----------

--------------TATATC--TTCT-----------TCATTGTA---------------------------

-------------------------ATT--TTTTAT-GCAC--GTG-CCT-CC-TTAGCA--GGGTAC--AA

AC-----------AACAT-A----TGAATGT-ACTTGTG-T-------------GAATTGTTTC--------

AAAATTTTCA------------------------------ACGCAAT-A-T-G-GA-CCC-ATA-----AT-

AAAATT--GCAGAGA--GTATG------------GCAAAATTT--CACA-A-ATT-TCGA--TACAG-----

---------CAT-AGCCT-------------------TGG-TTTGCACA----TAATTTCTGAAAAATTC

>Pv01Sk00630/1-538 Pv01Sk00630 undefined product 717008:717545 reverse

-TCCCGAAATAG-----ATTTTTCCTG--AAAATCCACGT-AAG-AA--T-------CT---GCACTG-AA-

TTTGGG-AG----------AAAACGCG-AC-AA-AT--TTCAGT--TC------------------------

-----------------------------------------AAAC-G--GATGAG---TATTCAC-------

---CAA-----CGAAAAAAA---TC-AAACAGTT-TT--------------------------GCA-CAC--

AAAC--G-AA-CCA------------TCC--T-TTC-------G-G--CC-C-T-AG-C---------A--G

TGATG-AAT-A--A-A-AA--C-TT-TA-TTGCG-AATACTGT-GGGACGAA----TCT------GGG----

-------------ACTTAAATCTCAG-C----------------C-AAA-ACTCAACAAACACA---G----

------------------TGA--C----GAA-CGTCTGG----------T-AA--AAATTTC-ATA-CCA--

--AAATACCC-------AAGGA-GT-----AAGGC---GT-----GGTA------AG-TCCCAGA-TCGAGA

GTGAA-------------------------------------------------------------------

------------------------------------------------------------------------

--------------T--AAAATA--------------------------GG-----------------ATTT

G----------------------CGAAAACAAAA--C--GTCCTGGC-----TTTTTTTCCC----------

--------------TGTATC--TTCT-----------CTTTGTGA---------------------------

-------------------------TTT--GTTTAA-GGAG--ATG-CCT-CC-TTACCT--GGGTGC--AA

AC-----------AACAT-A----GGAAAGT-GCTTGTT-T-------------GAA---------------

--TTTTTTCC------------------------------GCGCAAT-A-C-G-GA-CCC-AGA-----AT-

AAAATT--GTAGAAA--GTAGG------------GCGAAATCT--CACA-A-GTT-TTGG--TGGAG-----

---------GAT-AGCCT-------------------TGG-TTTGCTCA----AAATTTCTGAAAAATT-

>Pv01Sk00640/1-539 Pv01Sk00640 undefined product 718626:719164 reverse

-TCCCGAAATAG-----TTTTTTCCTG--AAAATCCACGT-AAG-AA--T-------CT---GCACTG-AA-

TTTCGA-AC----------AAAACGCG-AC-AA-AT--TTCAGG--TC------------------------

-----------------------------------------AAAC-G--GATGGG---TATTCAC-------

---CCA-----CGAAAAAAA---TA-AAACAGTT-TC--------------------------GCA-TAC--

AATC--G-AA-CCT------------TCC--T-TTC-------G-T--TC-C-T-GG-C---------A--G

TGATG-AAT-A--A-A-AA--A-TT-TA-TTGCC-AATCCCGT-GAAACGAA----TCT------GGG----

-------------GCTCAAATCTCAG-C----------------C-AAA-ACTCAACAGACACA---G----

------------------TGA--C----GAA-CGTCTGG----------T-AC--ACATTTC-AGA-CCA--

--AAATACCC-------AAGGA-GT-----AAGGC---GT-----GGTA------AG-TCCCAGA-CCGAGA

GTGAA-------------------------------------------------------------------

------------------------------------------------------------------------

--------------C--AAAACT--------------------------GG-----------------TTTT

C----------------------CGAAAACAAAA--C--GTCCTCGC-----TTTTCTTCCC----------

--------------CGTATC--TTCT-----------TTTTGTGA---------------------------

-------------------------TTT--GTTTAT-GGAC--GTG-CCT-CC-TCACCT--GGGTTC--AA

AC-----------AACAT-G----TGGAAGT-ACTTGTG-T-------------GAA---------------

--CTTTTTCA------------------------------GCGGAAT-A-C-G-GA-CCC-GGA-----GT-

AAAATT--GCAGAAA--GTAGG------------GCGAAATTTT-CACA-A-GTT-TTGG--TAGAG-----

---------GAT-AGCCT-------------------TGG-TTTGCACA----AAATTTCTGAAAAATT-

>Pv01Sk00650/1-538 Pv01Sk00650 undefined product 719166:719703 reverse

-TCCCGAAATAG-----TTTTTTCCTG--AAAATCCACGT-AAG-AA--T-------CT---GCACTG-AA-

TTTGGG-AC----------AAAACGCG-AC-AA-AT--TTCAGG--TC------------------------

-----------------------------------------AAAC-G--GATGGG---TATTCAC-------

---CCA-----CGATAAAAA---TC-AAACAGTT-TC--------------------------GCA-CAC--

AAAC--G-AA-CCT------------TCC--T-TTC-------G-G--CC-C-T-GG-C---------A--G

TGATG-AAT-A--A-A-AA--A-TT-TA-TTGCC-AATCCCGT-GGGACGAA----TCT------GCG----

-------------GCTCAAATCTCAG-C----------------C-AAA-ACTCAACAGACACA---G----

------------------TGA--C----GAA-CGTCTGG----------T-AA--AAATTTC-AGA-CCA--

--AAATACCC-------AAGGA-GT-----AAGGC---GT-----GGTA------AG-TCCCAGA-CCGAGA

GTGAA-------------------------------------------------------------------

------------------------------------------------------------------------

--------------A--AAAACT--------------------------GG-----------------TTTT

C----------------------CGAAAACAAAA--C--GTCCTAGC-----TTTTCTTCCC----------

--------------CGTATC--TTCT-----------TTTTGTGA---------------------------

-------------------------TTT--GTTTAT-GGAC--GTG-CCT-CC-TCACCT--GGGTGC--AA

AC-----------AACAT-G----TGGAAGT-ACTTGTG-T-------------GAA---------------

--CTTTTTCA------------------------------GCACAAT-A-C-G-GA-CCC-GGA-----GT-

AAAATT--GCAGAAA--GTAGG------------GTGAAATTT--CACA-A-GTT-TTGG--TAGAG-----

---------GAT-AGCCT-------------------TGG-TATGCACA----AAATTTCTGAAAAATT-

>Pv01Sk00660/1-538 Pv01Sk00660 undefined product 719705:720242 reverse

-TCCCGAAATAG-----TTTGTTCCTA--AAAATCCACGT-AAG-AA--T-------CT---GCACTG-AA-

TTTGGG-AC----------AAAACGCG-AC-AA-AT--TTCAGG--TC------------------------

-----------------------------------------AAAC-G--GATGGG---TATTCAC-------

---CCA-----CGAAAAAAA---TC-AAACAGTT-TC--------------------------GCA-CAC--

AAAC--G-AA-CCT------------TCC--T-TTC-------G-C--CC-C-T-GG-C---------A--G

TGATG-AAT-A--A-A-AA--A-TT-TA-TTCCC-AATCCCGT-GGGACGAA----TCT------GGG----

-------------GCTCATATCCCCG-C----------------C-AAA-ACTCAACACACACA---G----

------------------TGA--C----GAA-CGTCTGG----------T-AA--AAATTTC-ACA-CCA--

--AAATACCA-------AAGGA-GT-----AAGGC---GT-----GATA------AG-TCCCAGA-CCGAGA

GTGAA-------------------------------------------------------------------

------------------------------------------------------------------------

--------------C--AAAACT--------------------------GG-----------------TTTT

C----------------------CGAAAACAAAA--C--GTCCTGAC-----TTTTCTTCCC----------

--------------CGTATC--TTCT-----------TTTTGTGA---------------------------

-------------------------TTT--GTTTAT-GGAC--GTG-GCT-CC-TCACCT--GGGTGC--AA

AA-----------AACAT-G----TGGAAGT-ACTTGTG-T-------------GAA---------------

--CTTTTTCA------------------------------GCGCAAT-A-C-G-GA-CCC-GGA-----GT-

AAAATT--GCAGAAA--GTAGG------------GCGAAATTT--CACA-A-GTT-TTGG--TAGAG-----

---------GAT-AGCCT-------------------TGG-TTTGCATA----AAATTTCTGAAAAATT-

>Pv01Sk00670/1-538 Pv01Sk00670 undefined product 720244:720781 reverse

-TCCCGAAATAG----TTTTTTTCCTG--AAAATCCACGT-AAG-AA--T-------CT---GCACTG-AA-

TTTGGG-AC----------AAAACGCG-AC-AA-AT--TTCAGG--TC------------------------

-----------------------------------------AAAC-G--GATGGG---TATTCAC-------

---GCA-----CGAAAAAAA---TC-AAACAGTT-TC--------------------------GCA-CAC--

AAAC--G-AA-CCT------------TCT--T-TTC-------G-G--CC-C-T-GG-C---------A--G

TGATG-AAT-A--A-A-AA--A--T-TA-TTGCC-AATCCCGT-GGGACGAA----TCT------GCG----

-------------GCTCAAATCTCAG-C----------------C-AAA-ACTCAACAGACACA---G----

------------------TGA--C----GAA-CGTCTGG----------T-AA--AAATTTC-AGA-CCA--

--AAATACCC-------AAGGA-GT-----AAGGT---GT-----GGTA------AG-TCCCAGA-CCGAGA

GTGAA-------------------------------------------------------------------

------------------------------------------------------------------------

--------------C--AAAACT--------------------------GG-----------------TTTT

C----------------------CGAAAACAAAA--C--GTCCTGGC-----TTTTCTTCCC----------

--------------CGTATC--TTCT-----------TTTTGTGA---------------------------

-------------------------TTT--GTTTAT-GGAC--GTG-CCT-CC-TCACCT--GGGTGC--AA

AC-----------AACAT-G----TGGAAGT-ACTTGTG-T-------------GAA---------------

--CTTTTTCA------------------------------GCGCAAT-A-C-G-GA-CCC-GGA-----GT-

AAAATT--GCAGAAA--GTAGG------------GCGAAATTT--CACA-A-GTT-TTGG--TAGAG-----

---------GAT-AGCCT-------------------TGG-TTTGCACA----AAATTTCTGAAAAATT-

>Pv01Sk00680/1-538 Pv01Sk00680 undefined product 720783:721320 reverse

CTCCCGAAATAG-----TTTTTTCCTG--ATATTCCAAGT-AAG-AA--T-------CT---CCACTG-AA-

TTTGGG-AC----------AAAACGCG-AC-AA-AT--TTCAGG--TC------------------------

-----------------------------------------AAAC-A--GATGAG---TATTCAC-------

---CCA-----CGAAAAAAT---TC-AAAAAGTT-TC--------------------------GCA-TAC--

ACCC--G-AG-CCT------------TCC--T-TTC-------A-G--CC-C-T-GA-C---------A--G

TGATG-AAT-A--A-A-AA--A-TT-TA-TTGCC-AATCTAGT-GGGACGAA----TTT------GGG----

-------------GCTTAAATCCCAG-C----------------AAAAA-CTCAAC-AGACAAA---A----

------------------TGA--C----GAG-CGTCTGG----------T--A--AAAATTTCAGA-CCA--

--AAATACCG-------AAGGA-AT-----AAGGC---GT-----AGAA------AC-TCCCAGA-CCGAGA

GTGAA-------------------------------------------------------------------

------------------------------------------------------------------------

--------------C--AAAACT--------------------------GG-----------------TTTT

T----------------------CGAAAACAAGA--C--GTCATGAA-----TTTTCTTCCC----------

--------------CGTATC--TTCT-----------TTTTGTGA---------------------------

-------------------------TTT--TTTTAC-AGAC--ATG-CCT-CT-TT-CCT--GGGTGC--AA

AC-----------AACAT-A----TGAAAGT-ACTTGTC-T-------------GAA---------------

--TTTTTTAA------------------------------GCGCAAT-A-C-G-GA-CCC-AGA-----GT-

AAAATT--GCAGAAA--GTAGG------------GCGAAATTC--CACA-A-GTT-TTGG--TAAAG-----

---------GAT-AGCCT-------------------TGG-TTTGCACA----AAATTTCTGAAAAATT-

>Pv01Sk00690/1-509 Pv01Sk00690 undefined product 721320:721828 reverse

CTCCCGAAATAG-----TTTTTTCCAG--AAATTCCACGC-AAG-AT--T-------CT---CCACTG-CA-

TTTGGG-AA----------AAAACACG-AC-AA---------------------------------------

--------------------------------------------------------------AAC-------

---CCA-----CGAAAAAAA---TC-AAACAGTT-TC--------------------------GCA-CAC--

AAAT--G-AA-CCT------------TCC--T-TTC-------A-G--CC-C-T-TG-C---------A--G

TGAGG-AAG-A--A-A-AA--A-TT-TA-TTGCC-AATCTTGT-GGGACGAA----TCT------GGT----

-------------GCTCAAATATCCC-------------------AAAA-CTCAAC-AGACACA---G----

------------------TGA--C----GAG-CTGG----------------T--AAAAATTTTGA-CCA--

--AAATACCC-------AAGGA-GT-----AAGGC---GT-----CGTA------AG-TCCCAGA-CCGAGA

GTGAA-------------------------------------------------------------------

------------------------------------------------------------------------

--------------C--AAAATT--------------------------GG-----------------TTTC

C----------------------CGAAAACAAAA--C--GTCATCGG-----TTTTCTTCCC----------

--------------CGTATC--TTAT-----------TTTTGTGA---------------------------

-------------------------TTT--GTTTAC-GGAC--ATG-CCT-CC-TTACCC--GGATGC--AA

AC-----------AACAT-A----TAAAAGT-ACTTGTG-T-------------GAA---------------

--TTTTTTCA------------------------------GCGCATT-A-C-G-GA-TCA-TAA-----AT-

AATATT--GCAGAAA--GTGGG------------GCGAAATAT--GACA-A-GTT-TTGG--TAGAG-----

---------GAT-AGTCT-------------------CGG-TTTGCACA----AAATTTCTGAAAAATTC

>Pv01Sk00700/1-541 Pv01Sk00700 undefined product 721828:722368 reverse

CTCCCGAAATAG-----TTTTTTTCTG--AAATTCCATGT-AAG-AA--T-------CT---CCACTG-AA-

TTTTGG-AC----------AAAACGCG-AC-AA-AT--TTCAGG--TC------------------------

-----------------------------------------AAAC-G--GGTGAG---TATTCAC-------

---CTGC----GAAAAAAAA---TC-GAACAACT-TC--------------------------ACA-CAC--

AAAC--G-AA-CCT------------TCC--T-TTC-------A----CG-A-T-GG-C---------A--G

TGATG-AAT-A--A-A-AA--A-AT-TA-TTGTC-AATCTTGT-GGGACGAA----TCT------GGG----

-------------ACTCAAATCTCAG-C----------------C-AAA-ACTCAACAAACACA---G----

------------------TTA--T----GAG-CGTCTGG----------T--A-AAAATTTC-AGA-CCA--

--AAATACCG-------AAGGA-AT-----AAGGC---AT-----AGTA------AG-TCCCAGA-CTGAGA

GTGAA-------------------------------------------------------------------

------------------------------------------------------------------------

--------------C--AAAACT--------------------------GG-----------------TTTT

C----------------------TAAAAACAAAA--T--TTCCTAGC-----TTTTCTTCCT----------

--------------CGTATC--TTCT-----------TTTTGTGA---------------------------

-------------------------TTT--GTTTAT-GGAC--GTG-CTT-CT-TTACCT--GGGTGT--AA

AG-----------AACAT-A----TAAAAGT-ACTTGTG-T-------------GAAT--------------

--TTTTTTCA------------------------------ATGCAAT-A-C-G-GA-TCC-AGA-----AT-

AAAATT--GTAGAAA--GTATG------------GCGAAATCT--CACA-A-ATT-TTGG--TAGAG-----

---------GAT-AGCCT-------------------TGG-TTTGCACA----AAATTTCTGAAAAATTC

>Pv01Sk00710/1-548 Pv01Sk00710 undefined product 722369:722916 reverse

-TTCCGAAATAA-----ATTTTTGTTG--AAATTAAACGT-AGA-AA--T-------CT---CGACTG-AA-

TTTTGG-AA----------AAAATGCA-AC-AA-GT--TTCAGG--TC------------------------

-----------------------------------------AAAC-G--GATGAG---TATTCAT-------

---CTA-----GGAAAAAAA---TC-AAACTGCT-TC--------------------------ACA-CAC--

AAAC--G-AA-CCT------------TCC--C-TTC-------A-G--CA-C-T-GA-C---------A--G

TGATG-AAT-A--A-A-AA--A-AT-TA-TTGCA-AATCTTGT-GGAACGAA----TAT------GGG----

-------------GCTCAAATCTTAG-C----------------CCAAA--CTCAACACTCACA---A----

------------------TGA--C----TAA-TTTCTGG----------T--A-AAAATTTC-ATA-CCA--

--AAAGACCC-------AAGGA-AT-----AAGGC---GT-----AGTA------AG-TCCCAGA-CCGAGA

GTGAA-------------------------------------------------------------------

------------------------------------------------------------------------

--------------G--AAAATC--------------------------GG-----------------TTCT

C----------------------CGAAGACAAAA--T--GTCCTGGC-----TTTTCTTCCC----------

--------------TATATC--TCTGT----------TTTAGTGA---------------------------

-------------------------TTT--TTTAAT-GGAC--GTG-CCT-CC-TTAGAA--GGGCGC--AA

AC-----------AACAT-A----TAAAAGT-ATTTGTG-T-------------GAATGTTTTCG----G--

--ATTTTTTA------------------------------ATGCAAT-A-T-G-AA-CCA-AGA-----AT-

AAAATT--TCAGAAA--GTAGG------------GCTAAATTT--CACA-A-ATT-TTGG--TTGAG-----

---------GAT-ATTTC------------------TTGG-TTTGCACA-----AATTTTTGAAAATTT-

>Pv01Sk00730/1-528 Pv01Sk00730 undefined product 861065:861592 forward

---TCGAAATAG-----TTTTTTCCTG--AAAATCCACGT-AAG-AA--T-------CT---CCATTG-AA-

TTTGGA-AC----------AAAACACG-AA-AA-AT--TTCAGG--TC------------------------

-----------------------------------------AAAC-G--AATGAG---TATTCAC-------

---CCA-----CGAAAAAAA---TC-AAACAGTT-TC--------------------------GCA-TAC--

AAAT--A-AA-CCT------------TCC--T-TTC-------C-G--TC-C-T-GC-T---------A--G

TGATG-AAT-A--A-A-AA--A-TT-TA-TTGTC-ATTCTTGT-GGGACGAA----TCT------GGG----

-------------GCTCAAATCTCAG-C----------------C-AAA-ACTTAACAGACACA---G----

------------------TCA--C----AAA-CATCTCG----------T-AA--AAATTTC-AGA-CCA--

--AAATACCC-------AAGGA-GT-----AAGGC---TT-----GGTA------AG-TCCCAGA-CCGAGA

GTGAA-------------------------------------------------------------------

------------------------------------------------------------------------

--------------C--AACACT--------------------------GG-----------------TTTT

C----------------------CGAAAACAAAA--C--GTCATGAC-----TTTTCTTCCC----------

--------------CGTATC--TTCT-----------TTTTGTGA---------------------------

-------------------------TTT--ATTTAT-GGAC--GTG-CCT-CC-TTACCA--GGGTGC--AA

AC-----------AACAT-A----TGAAAGT-ACTTGTG-T-------------GAA---------------

--TTTTTTAA------------------------------GCGCAAT-A-C-G-GA-CCC-AGA-----AT-

AATATT--GCAGAAA--GTAGG------------GAGAAATTT--CACA-A-GTT-TTGG--TA--------

--------------GCCT-------------------TGG-TTTGCAAA----AAATTTCTAAGAAAT--

>Pv01Sk00740/1-537 Pv01Sk00740 undefined product 861594:862130 forward

--CCCGAAATAG-----TTTTTTCTTG--AAAATTTACGT-AAT-AA--T-------AT---CCACTG-AA-

TTTGGG-A-----------AAAACGCA-AA-AA-AT--TTCAGG--TC------------------------

-----------------------------------------AAAC-G--GATGAG---TATTCAC-------

---CCA-----CGAAAAAAA---TA-AAACAGTT-TC--------------------------GCA-CAC--

AAAC--A-AA-CCT------------TCC--T-TTC-------A-G--TC-C-A-GA-C---------A--A

TGATG-AAT-A--A-A-AA--A-TT-TA-TTGCC-AATCATAT-GGGACAAA----TCC------GGG----

-------------GCTCAAATCCCAG-C----------------C-AAA-ACTCAACAGACACA---G----

------------------TGA--T----GAA-CGTCTGG----------T-AA--AAATTTC-ATA-CCA--

--AAATACCC-------AAGGA-GT-----AAGGT---GT-----GGTA------AG-TCCCAGA-CAGAGA

GTCAA-------------------------------------------------------------------

------------------------------------------------------------------------

--------------C--AAAACT--------------------------AG-----------------TTTT

C----------------------CTAAAACAAAA--C--GTCCTGTT-----TTTTTTTCAC----------

--------------CGTATC--TTCT-----------TTTTGTGA---------------------------

-------------------------TTT--GTTTAT-GGAC--GTG-CCT-CC-TTACCT--GGGTGC--AA

AC-----------AACAT-A----TGACAAT-ACTTGTG-T-------------GAA---------------

--TTTTTTCA------------------------------GTGCAAT-A-C-G-AA-CCC-AGA-----AT-

AATATT--GCAGAAA--GTAGG------------GCGAAATTT--CAAA-A-GCT-TTGG--TAAAT-----

---------GAT-AGCCT-------------------TGG-TTTGCACA----AAAATTCTGAAAAATTC

>Pv01Sk00750/1-539 Pv01Sk00750 undefined product 862130:862668 forward

CTCCAGAAATAG-----TTTTTTCCTT--AAAATCTACGT-AAG-AA--T-------CT---CCTATG-AA-

ATTGAA-AA----------AAGAAGCG-AC-AA-AT--TTAAGG--TA------------------------

-----------------------------------------AAAC-G--GATGAG---TATTCAC-------

---CCA-----CAAAAAAAA---TC-AAACAGTT-TC--------------------------GCA-CAC--

AAAC--G-AA-CCT------------TCC--T-TTC-------A-G--AC-A-T-GG-C---------A--G

TGATG-AAT-A--C-A-AA--A-TT-TA-TTGCC-AATCTTGT-GGAACAAA----TCT------GGG----

-------------GCTAACATCTCAG-C----------------T-AAA-ACATAACAGACACA---G----

------------------TGA--C----GAA-CGTCTGG----------T-AA--AAATTTC-AGA-CCA--

--AAATACCC-------AAGGA-GT-----AAGGC---GT-----GGTA------AA-TCCCAGA-CCGAGA

GTAAA-------------------------------------------------------------------

------------------------------------------------------------------------

--------------C--AAAACT--------------------------GG-----------------TTTT

C----------------------CAAAAACAAAA--C--GTCATGGG-----TTTCCTTCCC----------

--------------CGTATC--TTCG-----------TTTTGTGA---------------------------

-------------------------TTT--GTTTAT-GGAC--GTG-CCT-CC-TTACCT--GGGTGC--AA

CA-----------AACAT-A----TGAAAGT-ACTTGTG-T-------------GAA---------------

--TTTTTTCA------------------------------GCGCAAT-A-C-G-GA-CCC-GGA-----AT-

AAAATT--GCAGAAA--GTAGG------------GCGAAATTT--CACA-A-GTT-TTGG--TACAT-----

---------GAT-AACCT-------------------TGG-TTTGCTCA----AAATTTCTGAAAAATT-

>Pv01Sk00760/1-538 Pv01Sk00760 undefined product 862670:863207 forward

-TCCCGGAATAG-----TTTTTTCCTG--AAAATCCACGT-AAG-AA--T-------CT---CCACCG-AA-

TTTGGG-AC----------ACAATGCG-AC-AG-AT--TTCAGG--TC------------------------

-----------------------------------------AAAT-G--GATGAG---TATTCAC-------

---CCA-----CGAAAAAAA---TC-AAACAGTT-TG--------------------------GCA-CAG--

GAAC--G-AA-CCT------------TCC--T-TTC-------A-G--GC-C-T-GG-C---------T--G

TGATG-AAT-A--A-A-AA--A-TT-TA-TTGCC-AATCTTGT-GGGACGAA----TCT------GAG----

-------------GCTCAAATCTCAG-C----------------C-GAA-ACTCAGCAGACACA---G----

------------------TGA--C----GAG-CGTCTGG----------T-AA--AACTTTC-AGA-CTG--

--AAATACCC-------AAGGA-GT-----AATGC---GT-----GGTA------AG-TCCCAGA-ACGAGA

GTGAA-------------------------------------------------------------------

------------------------------------------------------------------------

--------------C--AAAACT--------------------------GG-----------------TTTT

C----------------------CGAAAACAAAA--C--GCCATGTC-----TTTCCTTCCC----------

--------------CGTATC--TTCG-----------TTTTGTGA---------------------------

-------------------------TTT--GTTTAT-GGAA--GTG-TCT-CC-TTACCT--GGGTGC--AA

CC-----------AACAT-A----TGAAAGT-ACTTGTG-T-------------GAA---------------

--TTTTTTCA------------------------------GCGCAAT-A-C-G-GA-CCC-AGA-----AT-

AAAATT--GCAGAAA--CTAGG------------ACGAAATTT--CACA-A-GTT-TGGG--TAGAG-----

---------GAT-AGCCT-------------------TGG-TTTGCACA----AAATTTTTGAAAAATT-

>Pv01Sk00770/1-538 Pv01Sk00770 undefined product 863209:863746 forward

-TCCCAGAATAG-----TTTTTTCCTG--AAAATCCACGT-AAG-AA--T-------CT---CCACCG-AA-

TTTGGG-AC----------AAAAGGCG-AC-AG-AT--TTCAGA--TC------------------------

-----------------------------------------AAAC-G--GATGAG---TATTCAC-------

---CCA-----CGAAAAAAA---TA-AAACAGTT-TC--------------------------GCA-CAC--

AAAC--A-AA-CCT------------TCC--T-TTC-------A-G--CC-A-A-GA-C---------A--A

TGATG-AAT-A--A-A-AA--A-TT-TA-TTGCC-AATCTTCT-GCGACGAA----TCT------GGG----

-------------GCTCAAATCTTAG-C-----------------AGAA-ACTCAGCAGACACA---G----

------------------TGA--C----GAA-CGTCTGG----------T-AA--AAATTTC-AGA-CCG--

--AAATACCC-------AAGGA-GT-----AAGGC---GT-----GGTA------AG-TCCTAGA-GCGACA

GTGAA-------------------------------------------------------------------

------------------------------------------------------------------------

--------------C--AAAACT--------------------------GG-----------------TTTT

C----------------------CGAAAACAAAA--C--GTCATGGG-----TTTCCTTCCC----------

--------------CGTATC--TTCG-----------TTTTGTGA---------------------------

-------------------------TTT--GTTTAT-GGAT--GTG-CCT-CC-TTACCT--GGGTGC--AA

CC-----------AACAT-A----TGAAAGT-ACTTGTG-T-------------GAA---------------

--TTTTTTCA------------------------------GCGCAAT-A-C-G-GA-CCC-AGA-----AT-

AAAATT--GCAGAAA--CTAGG------------ACGAAATTT--CACA-A-GTT-TGGG--TAGAG-----

---------GAT-AGCCT-------------------TGG-TTTGCACA----AAATTTTTGAAAAATT-

>Pv01Sk00780/1-538 Pv01Sk00780 undefined product 863748:864285 forward

-TCCCAGAATAG-----TTTTTTCCTG--AAAATCCACGT-AAG-AA--T-------CT---CCACCG-AA-

TTTGGG-AC----------AAAAGGCG-AC-AG-AT--TTCAGG--TC------------------------

-----------------------------------------AAAC-G--GATGAG---TATTCAC-------

---CCA-----CGAAAAAAG---TC-AAACAGTT-TC--------------------------ACA-CAC--

GAAC--G-AA-CCT------------TCC--T-TTC-------A-G--CA-C-T-GG-C---------A--G

TGATG-AAT-A--A-A-AA--A-TT-TA-TTGCC-AATCTTGT-GGGACGAA----TCT------GGG----

-------------GCTCAAATCTCAG-C----------------C-CAA-ACTCACCAGACATA---G----

------------------TGA--T----GAA-CGTCTGG----------T-AA--AAATTTC-AGA-CCG--

--AAATACCC-------AAGGA-GT-----AAGGC---GT-----GGTA------AG-TCCCAGA-CCGAGA

GTGAA-------------------------------------------------------------------

------------------------------------------------------------------------

--------------C--AAAACT--------------------------GG-----------------TTTT

C----------------------CGAAAACAAAA--C--GTCATGGG-----TTTCCTTCCC----------

--------------CGTATA--TTCC-----------TTTTGTGA---------------------------

-------------------------TTT--GTTTAT-GGAC--GTG-CCT-CC-TTACCT--GGGTGC--AA

CC-----------AACAT-A----TGAAAGT-ACTTGTG-T-------------GAA---------------

--TTTTTTCA------------------------------GCGCAAT-A-C-G-GA-CCC-GGA-----AT-

AAAATT--GCAGAAA--GTATG------------GCGAAATTT--CACA-A-GTT-TTGG--TGGAG-----

---------GAT-AGCCT-------------------TGG-TTTGCACA----AAATTTCTGAAAAATT-

>Pv01Sk00790/1-538 Pv01Sk00790 undefined product 864287:864824 forward

-TCCTGGAATAG-----TTTTTTCCTG--AAAATCCACGT-AAG-AA--T-------CT---CAACTG-AA-

TTTGGA-AC----------AAAACGCG-AC-AG-AT--TTCAGA--TC------------------------

-----------------------------------------AAAC-G--GATGAG---TATTCAC-------

---CCA-----CGAAAAAAG---TC-AAACAGTT-TC--------------------------GCA-CAC--

AAAC--G-AA-CCT------------TCC--T-TTC-------A-T--CC-C-T-GG-C---------A--G

TGATG-AAT-A--A-A-AA--A-TT-TA-TTGCC-AATCTTGT-GGGACGAG----TTT------GGG----

-------------GCTCAAATCTCAT-C----------------C-GCA-ACTCAGCAGACACA---G----

------------------TGA--C----GAA-CCTCTGG----------T-AA--AAATTTC-AGA-CTG--

--AAATACCC-------AAGGA-GT-----AAGGC---GT-----GGTA------AG-TTCCAGA-CCGAGA

GTGAA-------------------------------------------------------------------

------------------------------------------------------------------------

--------------C--AAAACT--------------------------GG-----------------TTTT

C----------------------CGAAAACAAAA--C--GTCATGGC-----TTTCCTTCCC----------

--------------CGTATC--TTCG-----------TTTTGTGA---------------------------

-------------------------TTT--GTTTAT-GGAC--GTG-CCT-CC-TTAGCT--GGGTGC--AA

CC-----------AACAT-A----TGAAAGT-ACTTGTG-T-------------GAA---------------

--GTTTTTCA------------------------------GCGCAAC-A-C-G-GA-CCC-GGG-----AT-

AAAATT--GCAGAAA--GTAGG------------GCGAAATTT--CACA-A-GTT-TTGG--TGGAG-----

---------GAT-AGCCT-------------------TGG-TTTGCACA----AAATTTCTGAGAAATT-

>Pv01Sk00800/1-538 Pv01Sk00800 undefined product 864826:865363 forward

-TCCCGGAATAG-----TTTTTTCCTG--AAAATCCACGT-AAG-AA--T-------CT---CCACTG-AA-

TTTGGG-AC----------AAAACGCG-GC-AG-AT--TTCAGG--TC------------------------

-----------------------------------------AAAC-G--GATGAG---TATTCAC-------

---TCA-----CGAAAAAAA---TC-AAACAGTT-GG--------------------------GCA-CAG--

GAAC--G-AA-CCT------------TCC--T-TTC-------A-G--CC-C-T-GG-C---------T--G

TGATG-AAT-A--A-A-AA--A-TT-TA-TTGTC-AATCTTGT-GGGACGAA----TCT------GGG----

-------------GCTGAAATCTCAG-C-----------------AGAA-ACTCAGCAGACACA---G----

------------------TGA--C----GAG-CGTCTGG----------T-AA--AAATTTC-AGA-CCG--

--AAATACCC-------AAGGA-GT-----AAGGC---GT-----GGTA------AG-TCCCAGA-CCGAGA

GTGAA-------------------------------------------------------------------

------------------------------------------------------------------------

--------------C--AAAACT--------------------------TG-----------------TTTT

C----------------------CGAAAACAAAA--C--GTCCTGGC-----TTTTCTTCCC----------

--------------CGTATC--TTCC-----------TTTTGTGA---------------------------

-------------------------TTT--GTTTAT-GGAC--GTG-TCT-CC-TTACCT--GGGTGC--AA

CC-----------AACAT-A----TGAAAGT-ACTTGTG-T-------------GAA---------------

--TTTTTTCA------------------------------GCGCAAT-A-C-A-GA-CCC-GGA-----AT-

AAAATT--GCAGAAA--GTAGG------------GCAAAATTT--CACA-A-GTT-TTGG--TGGAG-----

---------GAT-AGCCT-------------------TGA-TTTGCACA----AAATTTCTGAAAAATT-

>Pv01Sk00810/1-537 Pv01Sk00810 undefined product 865365:865901 forward

-TCCCGGAATAG-----TTTTTTCCTG--AAAATCCACGT-AAG-AA--T-------CT---CCACTG-AA-

TTTGGG-AC----------AAAACGCG-GC-AG-AT--TTCAGG--TC------------------------

-----------------------------------------AAAC-G--GATGAG---TATTCAC-------

---TCA-----CGAAAAAAA---TC-AAACAGTT-GG--------------------------GCA-CAG--

GAAC--G-AA-CCT------------TCC--T-TTC-------A-G--CC-C-T-GG-C---------T--G

TGATG-AAT-A--A-A-AA--A-TT-TA-TTGTC-AATCTTGT-GGGACGAA----TCT------GGG----

-------------GCTGAAATCTCAG-C-----------------AGAA-ACTCAGCAGACACA---G----

------------------TGA--C----GAG-CGTCTAA----------T-AA--AAATTTC-AGA-CCG--

--AAATACCC-------AAGGA-GT-----AAGGC---GT-----GGTA------AG-TCCCAGA-CCGAGA

GTGAA-------------------------------------------------------------------

------------------------------------------------------------------------

--------------C--AAAACT--------------------------TG-----------------TTTT

C----------------------CGAAAACAAAA--C--GTCTTGGC-----TTTTCTTCCC----------

--------------CGTATC--TTCC-----------TTTTGTGA---------------------------

-------------------------TTT--GTTTAT-GGAC--GTG-TCT-CC-TTACCT--GGGTGC--AA

CC-----------AACAT-A----TGAAAGT-ACTTGGG-T-------------GAA---------------

--TTTTTTCA------------------------------GCGCAAT-A-C---GA-CCC-GGA-----AT-

AAAATT--GAAGAAA--GTAGG------------GCGAAATTT--CACA-A-GTT-TTGG--TAGAG-----

---------GAT-AGCCT-------------------TGG-TTTGCACA----AAATTTCTGAAAAATT-

>Pv01Sk00820/1-537 Pv01Sk00820 undefined product 865903:866439 forward

-TCCCGGAATAG-----ATTTTTCCTG--AAAATTCACGT-AAG-AA--T-------CT---CCACTA-AA-

TTTGGG-AC----------AAAACACG-AC-AG-AT--TTCAGG--TC------------------------

-----------------------------------------AAAC-G--AATGAG---TATTCAC-------

---CCA-----CGAAAAAA----TC-AAACAGTT-TC--------------------------GCA-CAC--

GAAC--G-AA-CCT------------TCC--T-TTC-------A-G--CA-C-T-GG-C---------A--G

TGGTG-AAT-A--A-A-AA--A-AT-TA-TTGCC-AATCTTGT-GCTACGAA----TCT------GGG----

-------------GCTCAAATCTCAG-C----------------C-GAA-ACTCCGCAGACACA---G----

------------------TGA--G----TAA-CCTCTGG----------T-AA--AAATTTC-AGA-CTA--

--AAATACCC-------AAGGA-GT-----AAGGC---GT-----GGTA------AG-TCCCAGA-CCGAGA

GTGAA-------------------------------------------------------------------

------------------------------------------------------------------------

--------------C--AAAACT--------------------------GG-----------------TTTT

C----------------------CGAAAACAAAA--C--GTCATGGG-----TTTCCTTCCC----------

--------------CGTATC--TTCG-----------TTTTGTGA---------------------------

-------------------------TTT--GTTTAT-GGAC--GTG-CCT-CC-TTACCT--GGGAGC--AA

CC-----------AACAT-A----TGAAAGT-ACTTGTG-T-------------GAA---------------

--TTTTTTCA------------------------------GAGCAAT-A-C-G-GA-CCC-AGA-----AT-

AAAATT--GCAGAAA--GTAGG------------GCGAAATTT--CACA-A-GTT-TTTG--TGGGG-----

---------GAA-AGCCT-------------------TGG-TTTGCACA----AAATTTCTGAAAAATT-

>Pv01Sk00830/1-537 Pv01Sk00830 undefined product 866441:866977 forward

-TCCCGGAATAG-----TTTTTTCCTG--AAAATCCACGT-AAG-AA--T-------CT---CCACTG-AA-

TTTGGG-AC----------AAAATGCG-AC-AG-AT--TTCAGG--TC------------------------

-----------------------------------------AAAC-G--GATGAG---TATTCAC-------

---CCA-----CGAAAAATA---TC-AAACAGTT-TC--------------------------GCA-CAG--

GAAC--G-AA-CCT------------TCC--T-TTC-------A-G--CC-C-T-GG-C---------A--G

TGATG-AAT-A--A-A-AA--A-TT-TA-TTGTC-AATCTTGT-GGGATGAA----TCT------GGG----

-------------GCTCAAATCTCAG-C----------------C-AAA-ACTCAGCAGACACA---G----

------------------TGA--C----GAA-CGTCTGG----------T-AA--AAATTTC-AGA-CCC--

--AAATACCC-------AAGAA-GT-----AAGGC---GT-----GGTA------AG-TCCCAGA-CCGAGA

GTGAT-------------------------------------------------------------------

------------------------------------------------------------------------

--------------C--AAAACT--------------------------GG-----------------TTTT

C----------------------CGAAAACAAAA--C--ATCCTGGC-----TTTTCTTTCC----------

--------------CGTCTC--TTCC-----------TATTGTTA---------------------------

-------------------------TTT--TTTTAT-GGAC--GTG-CCT-GA-TAACCT--GGGTGC--AA

AC-----------AACAT-A----TGAAAGT-AGTTGTG-T-------------GAA---------------

--TTTTTTCA------------------------------GCGCAAT-A-C-G-GA-CCT-GGA-----AT-

AAAATT--GCAAAAA--GTAGG------------GCGAAATTT--CTCA-A-ATT-TTGG--TAGA------

---------GAT-AGCCT-------------------TGG-TTTGCACA----AAATTTCTGAAAAATT-

>Pv01Sk00840/1-538 Pv01Sk00840 undefined product 866979:867516 forward

-TCCTGGAATAG-----TTTTTTCCTG--AAAATCCACGT-AAG-AA--T-------CT---CCACTA-AA-

TTTGGG-AC----------AAAACGCG-AT-AA-AT--TTGAGG--TC------------------------

-----------------------------------------AAAC-G--GATGAG---TATTCAC-------

---CCA-----CGAAAAAAA---TC-AAACAGTT-TC--------------------------GCA-CAG--

GAAC--G-AA-CCT------------TCC--T-TTC-------A-G--CC-C-T-GG-C---------T--G

TGATG-AAT-A--A-A-AA--A-TT-TA-TTGCC-AATCTTGT-GGGACGAA----TTT------GGG----

-------------ACTCAAATCTCAG-C----------------C-AAA-ACTCAGCAGACACA---G----

------------------TGA--C----GAA-CGTCTAG----------T-AA--AAATTTC-AGA-CAA--

--AAATACCC-------AAGGA-GT-----AAGGT---GT-----GGTA------AG-TCCCAGA-CCGAGA

GTGAA-------------------------------------------------------------------

------------------------------------------------------------------------

--------------C--AAAACT--------------------------GG-----------------TTTT

C----------------------CGAATACAAAA--C--GTCTTGGC-----TTTTCTTCCC----------

--------------CGTATC--TTCC-----------TTCTGTGA---------------------------

-------------------------TTT--GTTTAT-GGAC--GTG-CCT-CC-TTACCT--GGGTGC--AA

CC-----------AACAT-A----TGAAAGT-ACTTGTG-T-------------GAA---------------

--TTTATTCA------------------------------GCGCAAT-A-C-G-GA-CCC-GGA-----AT-

AAAATT--GCAGAAA--GTAGG------------GCGAAATTT--CACA-A-GTT-TTGG--TAGAG-----

---------GAT-AGCCT-------------------TGG-TTTGCACA----AAATTTCTGAAAAATT-

>Pv01Sk00850/1-538 Pv01Sk00850 undefined product 867518:868055 forward

-TCCCGGAATAG-----TTTTTTCCTG--AAAATACACGT-ATG-AA--T-------CT---CCACTA-AA-

TTTGGG-AC----------AAAACGCG-AC-AG-AT--GTCAGG--TC------------------------

-----------------------------------------GAAC-A--AATGAG---TATTCAC-------

---CCA-----CGAAAAAAA---TC-AAACAGTT-TC--------------------------ACA-CAC--

GAAC--G-AA-CCT------------TCC--T-TTC-------A-G--CC-C-T-GG-C---------A--G

TGATG-AAT-A--A-A-AA--A-TT-TA-TTGCC-AATCTTGT-GGGACGAA----TCT------GGG----

-------------GCTCAAATCTCAG-C----------------C-AAA-ACTTAGCCGACACA---G----

------------------TGA--C----GAA-CGTCTGG----------T-AA--AAATTTC-AGA-CCA--

--AAATACCC-------AAGGA-GT-----AAGGC---GT-----GGTA------TG-TCCCAGA-CCGAGA

GTAAA-------------------------------------------------------------------

------------------------------------------------------------------------

--------------C--AAAACT--------------------------GG-----------------TTTT

C----------------------CAAAAACAAAA--C--GTCCTGGC-----TTTTCTTCCC----------

--------------CGTATC--TTCC-----------TATTGTGA---------------------------

-------------------------TTT--TTTTAT-GGAC--GTG-CCT-GC-TTACCT--GGGTGC--AA

AA-----------AACAT-A----TGAAAGT-ACTTGTG-T-------------GAA---------------

--CTTTTTCA------------------------------GCGCAAT-A-C-G-GA-CCC-GGA-----AT-

AAAATT--GCGGAAA--GTAGG------------GCGAAATTT--CACA-A-GTT-TTGG--TAGAG-----

---------GAT-AACCT-------------------TGG-TTTGCACA----AAATTTCTGAAAAATT-

>Pv01Sk00860/1-538 Pv01Sk00860 undefined product 876570:877107 forward

-TCCCGAAATAG-----TTTTTTCCTG--AAAGTCCACAT-AAG-AA--T-------AT---GCACTG-AA-

TTTGGG-AC----------AAAACGCG-AC-AA-AT--TTCAGG--TC------------------------

-----------------------------------------AAAC-G--AATGAG---TATTCAC-------

---CAA-----CGAAAAAAA---AC-AAAAAGTT-TC--------------------------GCA-CAC--

AAAC--G-AA-CCT------------TCC--T-TTC-------A-G--CT-C-T-GG-C---------A--G

TGGTG-AAT-A--A-A-AA--A-TT-TA-TTGCC-AATCTTGT-GAAAAGAA----TCT------GGT----

-------------GCTAAAATCTCAG-C----------------C-TAA-ACTCAACAGACACA---G----

------------------TGA--C----GAA-CGTCTGG----------T-AA--AAATTTC-AGA-CCA--

--AAATACCC-------AAGGA-GT-----AGGGC---CT-----GGTA------AG-TCCCAAA-CCGAGA

CTGAA-------------------------------------------------------------------

------------------------------------------------------------------------

--------------C--AAAACT--------------------------GG-----------------TTTT

C----------------------CGAAAACAAAA--C--GTCCTGGC-----TTTTCTTCCC----------

--------------CGTATC--TTCT-----------TTTTGTGA---------------------------

-------------------------TTT--GTTTAT-GGAA--GTC-CCT-CC-TTACCT--AGGTGC--AA

AC-----------AACAT-A----TGCAAGT-ACTATTG-T-------------GAA---------------

--TTTTTTCA------------------------------ACGCAAT-A-C-G-GA-CCC-GGA-----AT-

AAAATT--GCAGAAA--GTAGG------------GCGAAATTT--CACA-A-GTT-TTGG--TAGAG-----

---------GAT-AGCCT-------------------TGG-TTTGCACA----AAATTTCTGAAAAATT-

>Pv01Sk00870/1-537 Pv01Sk00870 undefined product 877109:877645 forward

-TCCCGAAATAG-----TTTTTTCCTG--AAAGTCCACTT-AAG-AA--T-------TT---GCACTT-AA-

TTTGGG-AC----------AAAACGCG-AC-AA-AT--TTCAGG--TC------------------------

-----------------------------------------AAAC-G--GATGAG---TATTCAC-------

---CCA-----CGAAAAAGA---TC-AAACAGTT-TC--------------------------GCA-CAC--

AAAT--G-AA-CCT------------TCG--T-TTC-------A-C--CC-T-T-GG-C---------A--G

TGATG-AAT-A--A-A-AA--A-TT-TA-TTGTC-AATCTTGT-GGGACGAA----TCT------GTG----

-------------GCTCAAATCACAG-C----------------C-TAA-ACTTAAAAGACACA---G----

------------------TGA--C----GAA-CGTCTGG----------T-AA--AATTTTT-AGA-CC---

--AAATACCC-------AAGGA-GT-----AGGGC---GT-----GGTA------CG-TCCCACA-CCGAGA

GTGAA-------------------------------------------------------------------

------------------------------------------------------------------------

--------------C--AAAACT--------------------------GG-----------------TTTT

C----------------------CGAAAACAAAA--C--GTCCTGGT-----TTTTTTTCCA----------

--------------CGTATC--TTCT-----------TTTTATGA---------------------------

-------------------------TTT--GTTTAT-GGAC--GTG-CCT-CC-TCACCT--GGGTGC--AA

AC-----------AACAT-A----TGAAAGT-ACTTTTG-T-------------GAA---------------

--TTTTTTCA------------------------------GCGCAAT-A-C-G-GA-CCC-AGA-----AT-

AAAATT--GCAGAAA--GTAGG------------GCGAAATTT--CACA-A-GTT-TTGG--TAGAG-----

---------GAT-AGCCT-------------------TGG-TTTGCACA----AAATTTCTGAAAAATT-

>Pv01Sk00880/1-536 Pv01Sk00880 undefined product 877647:878182 forward

-TCCCGAAATAG-----TACTTTCCTG--AAAATCCACGT-AAA-AA--T-------CT---GCACTG-AA-

TTTGGG-AC----------AAAACGCG-AC-AA-AT--TTCAGG--TC------------------------

-----------------------------------------AAAC-G--GATGAG---TATTCAC-------

---CCA-----CACAAAAAA---TC-AAACAGTT-CT--------------------------GCA-CAC--

AAAC--G-AA-CCT------------TCC--T-TTC-------A-C--CC-T-T-GG-C---------A--A

TGATG-AAT-A--A-A-AA--A-TT-TA-TTTCC-AATCTTGT-GGGAAGAA----TCA------GGT----

-------------CCTCAAATCTCAG-C----------------C-TAA-ACTCAACAGACACA---G----

------------------TGA--A----GAA-CATCTGG----------T-AA--AAATTTC-AGA-CCA--

--AAATACGT-------AACGA-GT-----AGGGC---CT-----GGTA------AG-TCCCAGA-CCGAGA

CTGAA-------------------------------------------------------------------

------------------------------------------------------------------------

--------------C--AAAATT--------------------------GA-----------------TTTT

C----------------------CGAGAACAAAA--C--GTCCTGGC-----TGTT-TTCCC----------

--------------CGTATT--TTTT-----------TTTTGTGA---------------------------

-------------------------TTT--TGTTAG-GGAC--GTG-CCT-CC-TTACCT--GGGTGT--AA

AA-----------AACAT-A----TGAAAGC-ACTTGTG-T-------------GAA---------------

---TTTTTCA------------------------------ACTCAAT-A-C-G-GA-CCC-GGA-----AT-

AAAATT--GCAGAAA--ATAGT------------GCGAAATTT--CACA-A-GTT-TTGG--TAGAG-----

---------GAT-AGCCT-------------------TGG-TTTGCACA----AAATTTCTGAAAAATT-

>Pv01Sk00890/1-538 Pv01Sk00890 undefined product 878184:878721 forward

-TCCCGAAATAA-----TTTTTTCCTG--AAAGGCCACAT-TAG-AA--T-------CT---GCACTT-AA-

TTTGGG-AC----------AAAATGCG-AC-AA-AT--TTCAGG--TC------------------------

-----------------------------------------AAAC-G--GATGAG---TATTCAC-------

---CCA-----CGAAAAAAA---TC-AAACAGTT-TC--------------------------ACA-CAC--

AAAC--G-AA-CCT------------TCC--T-TTA-------A-G--TC-C-T-GG-C---------A--G

TGATG-AAT-A--A-A-AA--A-TT-TA-TTGCA-GATCGTGT-GGGACGAA----TCT------GTG----

-------------GCTCAAATCTCAG-C----------------C-TAA-ACTCAAAAGACACA---G----

------------------TGA--C----GAA-CGTCTTG----------T-TA--AAATTTT-AGA-CCA--

--AAATACCC-------AAGGA-GT-----AGGGC---GT-----GGTA------CG-TCCAACA-CCGAGA

GTGAA-------------------------------------------------------------------

------------------------------------------------------------------------

--------------C--AAAACT--------------------------TG-----------------TTTT

C----------------------TGAAAATAAAA--T--GTCCTGGC-----TTTTCTTCCC----------

--------------CGTATC--TTCT-----------TTATGTGA---------------------------

-------------------------TTT--GATTAT-GGAC--GTG-CCT-CC-TTAGCT--TGGTGC--AA

AC-----------AACAT-A----TGAAAGT-CCTTGTG-T-------------GAA---------------

--TTTTTTCA------------------------------ACGCAAT-A-C-G-GA-CCC-AGA-----AA-

AAAATT--GCAGAAA--ATAGG------------GCGAAATTT--CACA-A-GTT-TTGG--TAGAG-----

---------GAT-AGCCT-------------------TGG-TTTGCACA----AAATTTCTGAAAAATT-

>Pv01Sk00900/1-536 Pv01Sk00900 undefined product 878723:879258 forward

-TCCCGAAATAG-----TTTTTTCCTG--AAAATCCACAT-AAG-AA--T-------CT---ACACTG-AA-

TTTGGG-AC----------AAAACGCG-AC-AA-AT--TTTAGG--TT------------------------

-----------------------------------------AAAC-G--GATGTT---TATTCAC-------

---AC--------AAAAAAA---AC-AAACATTT-TC--------------------------GCA-CAC--

AAAA--G-AA-CCT------------TCC--T-TTC-------A-G--CT-C-T-GG-C---------A--G

TGATG-AAT-A--A-A-AT--A-TT-TA-TTGCG-AATCTTGT-GGGAAGAA----TCT------AGT----

-------------ACTCAAATCTCAG-C----------------C-TAA-ACTCAACAGACACA---G----

------------------TGA--C----GAA-CGTCTGG----------T-AA--AAATTTC-AGA-CCA--

--AAATACCC-------AAGGA-GA-----AGGGC---CT-----GGTA------AG-TCCCAGA-CCGAGA

CTGAA-------------------------------------------------------------------

------------------------------------------------------------------------

--------------C--AAAACC--------------------------GT-----------------TTTA

C----------------------CGAAAACAAAA--C--GTCCTGGC-----TTTTCTTCCC----------

--------------CGTATC--CTCT-----------TTTTGTGA---------------------------

-------------------------TTT--GTTTAT-GGAG--GTT-CCT-CC-TCACCT--GAGTGC--AA

AC-----------AACAATC----TGAAAGT-ACTTGTG-T-------------GAA---------------

--TTTTTTCA------------------------------ACGCAAT-A-C-G-GA-CTC-GAA-----AT-

AAAATT--GCAGAAA--GTAGG------------GCGAAATTT--TACA-A-TGT-TTGG--TAGAG-----

---------GAT-AGCCT-------------------TGG-TTTGCACA----AAATTTCTGAAAAATT-

>Pv01Sk00910/1-538 Pv01Sk00910 undefined product 879260:879797 forward

-TCCCGAAATAG-----TTTTTTCCTG--AACATCCCCCT-AAG-AA--T-------TT---GCACCT-AA-

TTTGGG-AC----------AAAACACG-AC-AA-AT--TTCAGG--TC------------------------

-----------------------------------------AAAC-G--GATGAG---TATTCAC-------

---CAA-----CGAAAAAAT---TC-AAATAGTT-TC--------------------------GCA-CAC--

AAAC--G-AA-CCT------------TCC--T-TTC-------A-T--TC-C-T-TG-C---------A--G

TGATG-AAT-A--A-A-AA--A-TT-TA-TTGTC-AATCTTGT-GGGAAGAA----TCT------GGT----

-------------GCTCAAATCTAAG-C----------------C-TAA-ACTCAACGGACATA---G----

------------------TGA--C----GAA-CGTCTGG----------T-AA--AAATTTC-AGA-CCA--

--AAATACCC-------AAGGA-GT-----AGGGC---CT-----GGTA------AG-TCGCAGA-CCGAGA

CTGAA-------------------------------------------------------------------

------------------------------------------------------------------------

--------------C--AAAACT--------------------------GG-----------------TTTT

C----------------------CGAAAACAAAA--T--GTCCTGGC-----TTTTCTTCCC----------

--------------CGTATC--TTCT-----------TTTTGTGA---------------------------

-------------------------TTT--ATTTAT-GGAA--GTG-CCT-CC-TCACCT--GGGTGC--AA

AC-----------AACAT-A----TGAAAAG-ACTTCTG-T-------------GAA---------------

--TTTTTTCA------------------------------GCGCAAT-A-C-G-GA-CCC-GGA-----AT-

AAAATT--GAAGAAA--GTAGG------------GCAAAATTT--CACA-A-GTT-TTGG--TAGAG-----

---------GAT-AGCCT-------------------TGG-TTTGGACA----AAATTTCTGAAAAATT-

>Pv01Sk00920/1-536 Pv01Sk00920 undefined product 879799:880334 forward

-TCCCAAAATAG-----TTTTTTCTTT--AAAATCCACCT-AAG-AA--T-------CT---GCACTG-AA-

TTTGGG-AC----------AAAACGCG-AC-AA-AT--TTCAGG--TC------------------------

-----------------------------------------AAAC-A--GATGAG---TATTCAC-------

---CCA-----CGAAAAAA----AC-AAACAGTT-TC--------------------------GCA-CAC--

AAAC--G-AA-CCT------------TCC--T-TTC-------A-G--CC-C-T-GG-C---------A--G

TGATG-AAT-A--A-A-AA--A-AT-TA-TTGAA-AATCTTGT-GAAAAGAA----TTT------GGT----

-------------GCTCAAATCTCAG-C----------------C-TAA-ACACAACAGACACA---C----

------------------TGA--C----GAA-CGTTTGG----------T-AA--AAATTTC-AGA-CCA--

--AAATACCC-------AAGGA-GT-----AGGGC---CT-----GGTA------AG-TCCCAGA-CCGAGA

CTGAA-------------------------------------------------------------------

------------------------------------------------------------------------

--------------C--AAAACT--------------------------GG-----------------TTTT

C----------------------CGAAAACAAAA--C--GTCCTGGC-----TTTTCTTCCC----------

--------------CGTATC--TTCT-----------TTTTGTGA---------------------------

-------------------------TTTT-GTTTAT-GGAA--GTG-CCA-CT-TTACCT--ATGTGC--AA

AC-----------AACAT-A----TGAAAGT-ACTTGTG-T-------------GAA---------------

--TTTTTTCA------------------------------ACGCAAT-A-C-G-GA-CCC-GGA-----AT-

AAAATT--GCAGAAA--GTAGG------------GCGAAATTT--CACA-A-GGT-TTGG--TAGAG-----

---------GAT-AGCCT-------------------TGG-TTTGCACA----AAATTTCTGAAAAT---

>Pv01Sk00930/1-538 Pv01Sk00930 undefined product 880337:880874 forward

-TCCCGAAAAAG-----TTTTTTCCTA--AACATCCACCT-AGG-AA--T-------CT---GCACTT-AA-

TTTGGG-AC----------AAAAGGCG-AC-AA-AT--TTTAGG--TT------------------------

-----------------------------------------AAAC-G--GATTAT---TATTCAC-------

---ACA-----AAAAAAAA----AC-AAACAGTT-TC--------------------------GCA-CAC--

AAAA--G-AA-CCT------------TCC--T-TTC-------A-G--CT-C-T-GG-C---------A--G

TGATG-AAT-A--A-A-AT--A-TT-TA-TTGCG-AATCTTGT-GGGAAGAA----TCT------AGT----

-------------ACTCAAATCTCAG-C----------------C-TAA-ACTCAACAGACACA---G----

------------------TGA--C----GAA-CGTCTGG----------T-AA--AATTTTC-AGA-CCA--

--AAATACCC-------AAGGA-GA-----AGGGC---CT-----GGTA------AG-TCCCAGA-CCGAGA

CTGAA-------------------------------------------------------------------

------------------------------------------------------------------------

--------------C--AAAACC--------------------------GT-----------------TTTA

C----------------------CGAAAACAAAA--C--GTCCTGGC-----TTTTCTTCCC----------

--------------CGTATC--CTCT-----------TTTTGTGA---------------------------

-------------------------TTT--GTTTAT-GGAG--GTT-CCT-CC-TCACCT--GAGTGC--AA

AC-----------AACAATC----TGAAAGT-ACTTGTG-T-------------GAA---------------

--TTTTTTCA------------------------------ACGCAAT-A-C-A-GA-CCC-GAA-----AT-

AAAATT--GCAGAAA--GTAGG------------GCGAAATTT--TACA-A-TGT-TTGG--TAGAG-----

---------GAT-AGCCT-------------------TGG-TTTGCACA----AAATTTCTGAAAAATT-

>Pv01Sk00940/1-538 Pv01Sk00940 undefined product 880876:881413 forward

-TCCCGAAATAG-----TTTTTTCCTG--AACATCCCCCT-AAG-AA--T-------TT---GCACCT-AA-

TTTGGG-AC----------AAAACACG-AT-AA-AT--TTCAGG--TC------------------------

-----------------------------------------AAAC-G--GATGAG---TATTCAC-------

---CAA-----CGAAAAAAT---TC-AAATAGTT-TC--------------------------GCA-CAC--

AAAC--G-AA-CCT------------TCC--T-TTC-------A-T--TC-C-T-TG-C---------A--G

TGATG-AAT-A--A-A-AA--A-TT-TA-TTGTC-AATCTTGT-GGGAAGAA----TCT------GGT----

-------------GCTCAAATCTAAG-C----------------C-TAA-ACTCAACGGACATA---G----

------------------TGA--C----GAA-CGTCTGG----------T-AA--AAATTTC-AGA-CCA--

--AAATACCC-------AAGGA-GT-----AGGGC---CT-----GGTA------AG-TCGCAGA-CCGAGA

CTGAA-------------------------------------------------------------------

------------------------------------------------------------------------

--------------C--AAAACT--------------------------GG-----------------TTTT

C----------------------CGAAAACAAAA--T--GTCCTGGC-----TTTTCTTCCC----------

--------------CGTATC--TTCT-----------TTTTGTGA---------------------------

-------------------------TTT--ATACAT-GGAA--GTG-CCT-CC-TCACCT--GGGTGA--AA

AC-----------AACAT-A----TGAAAAG-ACGTGTG-T-------------GAA---------------

--TTTTTTCA------------------------------GCGCAAT-A-T-G-GA-CCC-GGA-----AT-

AAAATT--GAAGAAA--GTAGG------------GCAAAATTT--CACA-A-GTT-TTGG--TAGAG-----

---------GAT-AGCCT-------------------TGG-TTTGGACA----AAATTTCTGAAAAATT-

>Pv01Sk00950/1-528 Pv01Sk00950 undefined product 881415:881942 forward

-TCCCAAAATAG-----TTTTTTCCTT--AAAATCCACCT-AAG-AA--T-------CT---GCACTG-AA-

TTTGGG-AC----------AAAACGCG-AC-AA-AT--TTCAGG--TC------------------------

-----------------------------------------AAAC-A--GATGAG---TATTCAC-------

---CCA-----CGAAAAAA----AC-AAACAGTT-TC--------------------------GCA-CAC--

AAAC--A-AA-CCT------------TCC--T-TTC-------A-G--CC-C-T-GG-C---------A--G

TGATG-AAT-A--A-A-AA--A-AT-TA-TAGAA-AATCTTGT-GAAAAGAA----TTT------GGT----

-------------GCTCAAATCTCTG-C----------------C-TAA-ACTCAACAGACTCA---C----

------------------TGA--C----GAA-CGTTTGG----------T-AA--AAATTTC-AGA-CCA--

--AAATATTC-------AAGGA-GT-----AGGGC---CT-----GGTA------AG-TCCCAGA-CCGAGA

CTAAA-------------------------------------------------------------------

------------------------------------------------------------------------

--------------C--AAAACT--------------------------GG-----------------TTTT

C----------------------CGAAAACAAAA--C--GTCCTAGC-----TTTTCTTCCC----------

--------------TGTATC--TTCT-----------TTTTGTGA---------------------------

-------------------------TTTT-GTTTAT-GGAC--GTG-CCT-TC-TCACCT--GGGTGC--AG

AC-----------AACAT-A----TGAAAGT-ACTTGTG-T-------------GAA---------------

--TTTTTTCA------------------------------ACGCAAT-A-C-G-AA-CCC-GGA-----AT-

AAAATT--GAAGAAA--GTAGG------------GCGAAATTT--CACA-T-ATT-TTGG--TAG-------

-------------------------------------TGG-TTTGCACA----AAATTTCTGATAAATT-

>Pv01Sk00970/1-538 Pv01Sk00970 undefined product 890625:891162 forward

-TCCCGAAATAG-----TTGTTTCCTG--AAAGTCCACGT-AAG-AA--T-------CT---TCACTT-AA-

CTTGGG-AC----------AAAACGCG-AC-AA-AT--TTCAGG--TC------------------------

-----------------------------------------AAAC-G--GATGAG---TTTTCAC-------

---CCA-----CGAAAAAAA---TC-AAACAGTT-TC--------------------------GCA-CAC--

ACAC--G-AA-CCT------------TCC--T-TTT-------A-G--CC-C-T-GG-C---------A--G

TGATG-AAT-A--A-A-AA--A-TT-TA-TTGTC-AATCTTGT-GGGAAGAA----TCT------GGT----

-------------GCTCAAATTTAAG-C----------------C-TAA-ACTCAACGGACATA---G----

------------------TGA--C----GAA-CGTCTGG----------T-AA--AAATTTC-AGA-CCA--

--AAATACCC-------AAGGA-GT-----AGGGC---CT-----GGTA------AG-TCGCAGA-CCGAGA

CTGAA-------------------------------------------------------------------

------------------------------------------------------------------------

--------------C--AAAACT--------------------------GG-----------------TTTT

C----------------------CGAAAACAAAA--T--GTCCTGGC-----TTTTCTTCCC----------

--------------CGTATC--TTCT-----------TTTTGTGA---------------------------

-------------------------TTT--ATTTAT-GGAA--GTG-CCT-CC-TCACCT--GGGTGC--AA

AC-----------AACAT-A----TGAAAAG-ACTTCTG-T-------------GAA---------------

--TTTTTTCA------------------------------GCGCAAT-A-C-G-GA-CCC-GGA-----AT-

AAAATT--GAAGAAA--GTAGG------------GCAAAATTT--CACA-A-GTT-TTGG--TAGAG-----

---------GAT-AGCCT-------------------TGG-TTTGGACA----AAATTTTTGAAAAATT-

>Pv01Sk00980/1-539 Pv01Sk00980 undefined product 893198:893736 forward

-TCCCTAAATTG-----TTTTTTCCTG--AAAGTCCACGT-AAG-AA--T-------AT---GCACTT-AA-

TTTGGG-AC----------AAAACGCG-AC-AA-AT--TTCAGG--TC------------------------

-----------------------------------------AAAC-G--GATGAG---TATTCAC-------

---CAA-----CGAAAAAAA---TCAAAACAGTT-TT--------------------------GCA-CAC--

AAAT--T-AA-CCT------------TTT--T-TTC-------A-G--CC-G-T-GG-C---------A--G

TGATG-AAT-A--A-A-AA--A-TT-TA-TTGTC-AATCTTGT-GGGAAGAA----TCT------GGT----

-------------CCTCAAATCACAG-C----------------C-TAA-ACTCAACAGACTAA---G----

------------------TGA--C----GAA-CGTCTGG----------T-AA--AAATTTC-AGA-CCT--

--AAATACCC-------AAGGA-GT-----AGGGC---CT-----GGTA------AG-TCCCAGC-ACGATA

CTGAA-------------------------------------------------------------------

------------------------------------------------------------------------

--------------C--AAAACT--------------------------GG-----------------TTTT

T----------------------CGAAAAGAAAA--C--GTCCTCTC-----TTTTCTTACC----------

--------------CGTATC--TTCT-----------TTTGGTGA---------------------------

-------------------------TTT--GTTTAT-GGAC--GGG-CCT-CC-TTACCT--GGGTGC--AA

AA-----------AACAT-G----TGAAAGT-ACTTGTT-T-------------GAA---------------

--TTTTTTCA------------------------------ACGCAAT-A-C-G-GA-CCC-GGA-----AT-

AAAATT--GCAGAAA--GTAGC------------ACGAAATTT--CACA-A-GTT-TTGG--TAAAG-----

---------TAT-AGCCT-------------------TTG-TTAGCACA----AAATTTCTGAAAAATT-

>Pv01Sk00990/1-538 Pv01Sk00990 undefined product 893738:894275 forward

-TCCCGAAATAG-----TTTTTTCATG--AAAGTCCACGT-AAG-AA--T-------CT---TCACTT-AA-

CTTGGG-AC----------AAAACGCG-AC-AA-AT--TTCAGG--TC------------------------

-----------------------------------------AAAC-G--GATGAG---TTTTCAC-------

---ACA-----CGAAAAAAA---TC-AAACAGTT-TC--------------------------GCA-CAC--

AAAC--G-AA-CCT------------TCC--T-TTC-------A-G--CC-C-T-GG-C---------A--G

TTATG-AAT-A--A-A-AA--A-TT-TA-TCGCC-AATCTTGT-GGTAATAA----TCT------GGT----

-------------GCTCAATTCTCAG-C----------------C-TAA-ACTCAACAGACACA---G----

------------------TGA--C----AAA-CGTATGG----------T-AA--AAATTTC-AAA-CCA--

--AAATACCC-------AAGGA-GT-----AGGGC---CT-----GGTA------AG-TCCCAGA-CCGAGA

CTGAA-------------------------------------------------------------------

------------------------------------------------------------------------

--------------C--AAAACT--------------------------GG-----------------TTTT

C----------------------CAAAAACAAAA--C--GTCCTGGC-----TTTTCTTCCC----------

--------------CGTATC--TTCT-----------TTTTGTGA---------------------------

-------------------------TTT--GTTTAT-GGAC--GTG-CAT-CC-TTACCT--GGGTGC--AA

AC-----------GACAT-A----AGAAAGA-ACTTGTG-T-------------GAA---------------

--TCTTTTTA------------------------------ACGCAAT-A-C-G-GA-TAC-GGA-----AT-

AAAATT--ACAGAAA--GTAGG------------GCAAAATTT--CACA-A-GTT-TTGG--TAGAG-----

---------GAT-AGCCT-------------------TGG-TTTGCACA----AAATTTCTGAAAAATT-

>Pv01Sk01000/1-538 Pv01Sk01000 undefined product 894277:894814 forward

-TCCCGAAATAG-----TTTTTTCCTG--AAAGTCCACGT-AAG-AA--T-------CT---TCACTT-AA-

TTTGGG-AC----------AAAACGCG-AC-AA-AT--TTCAGG--TC------------------------

-----------------------------------------AAAC-G--GATGAG---TTTTCAC-------

---CCA-----CGAAAAAAA---TC-AAACAGTT-TC--------------------------GCA-CAC--

GAAC--T-AA-CCT------------TCC--T-TTC-------A-G--CC-C-T-GG-C---------A--G

TGATG-AAT-A--A-A-AA--A-TT-TA-TTGTC-AATCTTGT-GGGACGAA----TCT------GGA----

-------------GCACAAAGCTTAG-C-----------------AAAA-ACTCAACAGACACA---G----

------------------TGA--C----GAA-CGTCTGG----------T-AA--AAATTTC-AGA-CCA--

--AAATATCC-------AAGGA-CT-----AAGGC---GT-----GGTA------AG-TCCCAGA-CCGAGA

GTGAT-------------------------------------------------------------------

------------------------------------------------------------------------

--------------C--AAAACT--------------------------GG-----------------TTTT

C----------------------CGAAAACCAAA--C--ATCTTGGC-----TTTTCTTCCC----------

--------------CGTATC--TTCT-----------TTTTGTGA---------------------------

-------------------------TTT--TTTTAT-GGAC--GTG-CTT-CC-TTACCT--GGGTGC--AA

AC-----------AACAT-A----TGAATGT-ACTTGTG-T-------------TAA---------------

--TTTTTTCA------------------------------ATGCAAT-A-C-G-GA-CCC-GGA-----AT-

AAAATT--GCAGAAA--GTAGG------------GCGAAATTT--CACA-A-GTT-TTTG--TAGAG-----

---------GAT-AGCCT-------------------TTG-TTTGCACA----AAATTTCTGAAAAATT-

>Pv01Sk01010/1-537 Pv01Sk01010 undefined product 894816:895352 forward

-TCCCGAAATAG-----TTGTTTCCTG--AAAGTCCACGT-AAG-AA--T-------CT---TCACTT-AA-

CTTGGG-AC----------AAAACGCG-AC-AA-AT--TTCAGG--TC------------------------

-----------------------------------------AAAC-G--GATGAG---TTTTCAC-------

---CCA-----CGAAAAAAA---TC-AAACAGTT-TC--------------------------GCA-CAC--

ACAC--G-AA-CCT------------TCC--T-TTT-------A-G--CC-C-T-GG-C---------A--G

TGATG-AAT-A--A-A-AA--A-TT-TA-TTGCC-AATCTTGT-GGGAAGAA----TCT------GGT----

-------------ACTCAAATCTCAG-C----------------C-TAA-ACTCAACAGACACA---G----

------------------TGA--C----GAA-CGTCTGG----------T-AA--AAATTTC-ATA-CGA--

--AAATACTT-------GAGGA-AT-----ACGGC---GT-----GGTA------AG-GCCCAGA-CCGAGA

CTGAA-------------------------------------------------------------------

------------------------------------------------------------------------

--------------A--GAAACT--------------------------GG-----------------TTTT

C----------------------CGAAAACAAAA--T--GTCCTGGC-----CTTTCTTCCC----------

--------------TGTATC--TTCT-----------TTTTGTGA---------------------------

-------------------------TTT--GTTTAT-GGAC--GTG-CTT-CC-TTACCT--TGGTGC--AA

AC-----------AACAT-A----TGAAAGT-ACTTGTG-T-------------GAA---------------

---TTTTTCA------------------------------ACACAAT-A-C-G-GA-TCC-TGA-----AT-

AAAATT--GTAGAAA--GTAGG------------GCAAAATTT--CACA-A-GTT-TTGG--CCGAG-----

---------GAT-AGCCT-------------------TTG-TTTGCACA----AAAATTCTGAAAAATT-

>Pv01Sk01020/1-537 Pv01Sk01020 undefined product 895356:895892 forward

--CCGGAAAAGG-----TTTTTTGCTG--AAAGTCCAGGT-AAG-AA--T-------CT---TCACTT-AA-

CTTGGG-AC----------AAAACGCG-AC-AA-AT--TTCAAG--TC------------------------

-----------------------------------------AAAC-A--GATGAG---TTTTCAC-------

---CTA-----CGAAAAAAA---TC-AAACAGTT-TC--------------------------GCA-CAC--

AAAC--G-AA-ACT------------TCC--T-TTC-------A-G--TC-C-T-GG-C---------A--G

TAATG-AAT-A--A-A-AA--A-TT-TA-TTGTC-AATCTTGT-GGGAAGAA----TCT------GGT----

-------------GCTCAAATCTCAT-C----------------C-TAA-ACTCAACAGACACA---G----

------------------TGA--C----AAA-CGTCTGG----------T-AA--AAATTTC-ATA-CAA--

--AAATACCC-------GAGGA-GT-----AAGGC---GT-----GGTA------AG-TCCCAGA-CCGAGA

CTGAA-------------------------------------------------------------------

------------------------------------------------------------------------

--------------C--AAAATT--------------------------TG-----------------TTTT

C----------------------CGAGAACAAAA--C--GTCCTGGC-----TTTTCTTCCC----------

--------------CGTATC--TTCT-----------TTTTGTGA---------------------------

-------------------------TTT--GTTTAT-GGAC--GTG-CCT-CC-TTACCT--GGGTGC--AA

AC-----------AACAT-A----TGAAAGT-ACTTGTG-T-------------GAA---------------

--TTTTTTCA------------------------------ACGCTAT-A-C-G-GA-CCC-AGA-----AT-

AAAATT--TCAGAAA--GTAGG------------GCGAAAGTT--CACA-A-GTT-TTGG--TAGAG-----

---------GAT-AGCCT-------------------TGG-TTTGCACA----AAATTTCTGAAAAATT-

>Pv01Sk01030/1-539 Pv01Sk01030 undefined product 895894:896432 forward

-TCCCTAAATTG-----TTTTTTCCTG--AAAGTCCACGT-AAG-AA--T-------AT---GCACTT-AA-

TTTGGG-AC----------AAAACGCG-AC-AA-AT--TTCAGG--TC------------------------

-----------------------------------------AAAC-G--GATGAG---TATTCAC-------

---CAA-----CGAAAAAAA---TCAAAACAGTT-TT--------------------------GCA-CAC--

AAAC--G-AA-CCT------------TTC--T-TTC-------T-G--CC-C-T-GG-C---------A--G

TGATG-AAT-A--A-A-AA--A-TT-TA-TTGTC-AATCTTGT-GGGACGAA----TCT------GGA----

-------------GCACAAAGCTTAG-C-----------------AAAA-ACTCAACAGACACA---G----

------------------TGA--C----GAA-CGTCTGG----------T-AA--AAATTTC-AGA-CCA--

--AAATATCC-------AAGGA-CT-----AAGGC---GT-----GGTA------AG-TCCCAGA-CCGAGA

GTGAT-------------------------------------------------------------------

------------------------------------------------------------------------

--------------C--AAAACT--------------------------GG-----------------TTTT

C----------------------CGAAAACCAAA--C--ATCTTGGC-----TTTTCTTCCC----------

--------------CGTATC--TTCT-----------TTTTGTGA---------------------------

-------------------------TTT--TTTTAT-GGAC--GTG-CTT-CC-TTACCT--GGGTGC--AA

AC-----------AACAT-A----TGAATGT-ACTTGTG-T-------------TAA---------------

--TTTTTTCA------------------------------ATGCAAT-A-C-G-GA-CCC-GGA-----AT-

AAAATT--GCAGAAA--GTAGG------------GCGAAATTT--CACA-A-GTT-TTTG--TAGAG-----

---------GAT-AGCCT-------------------TTG-TTTGCACA----AAATTTCTGAAAAATT-

>Pv01Sk01040/1-532 Pv01Sk01040 undefined product 1115061:1115592 reverse

CTCCCATAATAG-----TTTTTTCTTG--AAAATCCATGT-AAG-AA--T-------CT---CCACTC-AA-

TTTGGG-AC----------AAAACGCG-AC-AA-AT--TTTAGG--TC------------------------

-----------------------------------------AAAC-A--GATGAG---TATTCAC-------

---CCA-----CGAAAAAAA---TC-AAACAGTT-TC--------------------------ACA-CAC--

AAAC--G-AA-CTT------------TTC--T-TTC-------A-G--CC-C-T-GG-C---------A--G

TGATG-AAT-A--A-A-AA--A-TT-TA-TTGCA-AATCTTTT-GGGATGAA----AGT------GGG----

-------------ACTCAAATCTCAA-C----------------C-AAA-ACTCAACAGACAGG---G----

------------------TGA--C----GAA-CGTTTGG----------T--A--AAAATTTCAGA-CCA--

--AAATACCC-------AAGGA-GT-----AAGGC---GT-----GGTA------AG-TCCCAGA-CCGAAA

GTGAA-------------------------------------------------------------------

------------------------------------------------------------------------

--------------C--AAAACT--------------------------GG-----------------TTTT

T----------------------GAAAAACAAAA--T--GTCATGGC-----T--------A----------

--------------CGTATC--TTCT-----------TTTTGTGA---------------------------

-------------------------TTT--GTTTAT-GGAC--GTG-CCT-CC-TTACCT--GAGTGC--AA

AC-----------AACAT-A----TAGAAGT-ACTTGTG-T-------------GAAT--------------

--TTTTTTCT------------------------------GCGCAAT-A-C-G-GA-TTC-AGA-----AT-

AAAATT--TCATAAA--GTAGG------------ACGAAATTT--CACA-A-GTT-TTTG--TAGAA-----

---------GAT-TGTCT-------------------TGG-TTTGCACA----AAAATTATGAAAAAAT-

>Pv01Sk01060/1-540 Pv01Sk01060 undefined product 1115835:1116374 reverse

CTCCTGAAGTAG-----TTTTTTCCTG--AAATTCCACGC-AAG-AA--T-------CT---TCACTT-AA-

TTTGGG-AC----------AAAACGTG-AC-AA-AT--TTCAGG--TC------------------------

-----------------------------------------AAAC-G--GATGAG---TATTCAC-------

---CCA-----CGAAAAAAA---TC-AAACAGTT-TC--------------------------ACA-CAC--

AAAC--G-AA-CCT------------TCC--T-TTC-------A-G--CC-C-T-GG-C---------A--A

TGATG-AAT-A--A-A-AA--A-TT-TA-TTGTC-AATCTTGT-GGGACGAA----TCT------GGG----

-------------GCTCAAATCTCAG-C----------------C-AAA-ACACAATAGACACA---G----

------------------TGA--C----GAA-CGTCTGG----------T-AA--AAATTTT-AGA-CCA--

--AAATACTC-------AAGAA-GT-----AAGGC---GC-----AGTA------AG-TCCCATT-CCTAGA

GGGAA-------------------------------------------------------------------

------------------------------------------------------------------------

--------------C--AAATCT--------------------------GG-----------------TTTT

C----------------------CGAAAACAAGA--C--GTCCTGGC-----TTTTCTTCCC----------

--------------CGTATA--TTCT-----------TTTTGTGA---------------------------

-------------------------TTT--GTTTAT-GGGC--GTG-CCT-CC-TTACCA--GGGTGC--AA

AC-----------AATGT-A----TGAAAGT-ACTTGTG-T-------------TTA---------------

--TTTTTTCA------------------------------GCGCAAT-A-C-G-AA-CCC-AGA-----AT-

AAAATT--GCAGAAA--GTAGG------------GCGAAACTT--GACA-T-GTT-TTCG--TAGAG-----

---------GAT-AGCCT-------------------TGG-TTTGCACA----AAATTTCTAAAAAATTC

>Pv01Sk01070/1-540 Pv01Sk01070 undefined product 1116374:1116913 reverse

CTCCCGAAATAG-----TTTTTTCCTG--AAATTCCACGC-AAT-AA--T-------CT---CCACTG-AA-

TTTGGG-AC----------AAAACTCG-GC-AA-AT--TTCAGG--TC------------------------

-----------------------------------------AAAC-G--AATGAG---TATTCAC-------

---CCA-----CTAAAAAAA---TC-AAACAGTT-TC--------------------------GCA-CAC--

AAAC--G-AA-CCT------------TCC--T-TTC-------A-G--CC-C-T-GA-C---------A--A

TGATG-AAT-A--A-A-AA--A-TT-TA-TTTCC-AATCTTGT-GCGACGAA----TCT------GGG----

-------------TCTCAAATCTCAG-C----------------C-AAA-ACACAACAGACACA---G----

------------------TGA--C----GAA-CGTCTGG----------T-AA--AAATTTC-AGT-CCA--

--AAATACCC-------AATGA-GT-----AAGGC---GT-----AGTA------AG-TCACAGA-CCGAGA

GCGAA-------------------------------------------------------------------

------------------------------------------------------------------------

--------------C--AAAACT--------------------------TT-----------------TTTT

C----------------------CGAAAACAAGA--C--GTCCTGGC-----TTTTCTTCCC----------

--------------CGTATC--TTCT-----------TTTTGTGA---------------------------

-------------------------TTT--GTTTAT-GGGC--GTG-CCT-CC-TTACTT--GGGTGC--AA

AC-----------AACGT-A----TGAAAGT-ACTTGTG-T-------------GAA---------------

--TTTTTTCA------------------------------GCGCAAT-A-C-G-GA-CCC-AAA-----AT-

AAAATT--GCAGAAA--GTAGG------------GCGAAACTT--CAAG-A-GTT-TTTG--TAGAG-----

---------GAT-AGCCT-------------------TGG-TTTGCACA----AAATTTCTGAAAAATTC

>Pv01Sk01080/1-540 Pv01Sk01080 undefined product 1116913:1117452 reverse

CTCCCAAAATAG-----TTTTTTCCTG--AAAATCCACCT-AAG-AA--T-------CT---CCAGTG-AA-

TTTGGG-AC----------AGAACGCG-AC-AA-AT--ATCAGG--TC------------------------

-----------------------------------------AAAC-G--GATGAG---TATTCAC-------

---CCT-----CGAAAAACA---TC-AAACAGTT-TC--------------------------GCA-CAC--

AAAC--G-AA-TCT------------TCC--T-TTC-------A-G--CC-C-T-GG-C---------A--G

TGATG-AAT-A--A-A-AA--A-TT-TA-TTGTC-AATCTTGT-GGGACGAA----TCT------GGG----

-------------GCTCAAATCTCAG-C----------------C-AAA-ACACAACAAACACA---G----

------------------TGA--C----GAA-CGTCTGG----------T-AA--AAATTTA-AGA-CCA--

--AAATACCC-------AAGGA-GT-----AAGGC---GT-----AGTA------AG-TCCCAGA-CCGAGA

GCGAA-------------------------------------------------------------------

------------------------------------------------------------------------

--------------C--TAAACT--------------------------GG-----------------TTTT

C----------------------CGAAAACAAGA--T--GTCCTGGT-----TTTTCTTCCC----------

--------------CGTATT--TTCT-----------TTTTGTGA---------------------------

-------------------------TTT--GTTTAT-GGAC--GTG-CCT-CC-TTACCT--GGGTGC--AA

AC-----------AACAT-A----GGAAAGT-ACTTGTG-T-------------GAA---------------

--TTTTTTCA------------------------------GCGCAAT-A-C-G-GA-CCC-AGA-----AT-

TAAATT--GCAGAAA--GTATG------------GCGAAACTT--CACA-A-GTT-TTGG--TAGAG-----

---------GAT-AGCCT-------------------TGG-TTTGCACA----AAATTTCTGAAAAATTC

>Pv01Sk01090/1-539 Pv01Sk01090 undefined product 1117453:1117991 reverse

CTCCCGAAATAG-----TTTTTTCCTG--AAATTCCACGC-AAG-AT--T-------CT---CCACTG-AA-

TTTGGG-AC----------AAAACGTG-AC-AA-AT--TTCAGG--TC------------------------

-----------------------------------------AAAC-G--GATGAG---TATTCAC-------

---CCA-----CTGAAAAAA---TC-AAACAGTT-TC--------------------------GCA-CAC--

AAAC--G-AA-CCT------------TCC--T-TTC-------A-G--CC-C-T-GG-C---------A--G

TGATG-AAT-A--A-A-AA--A-TT-AA-TTGCC-AATCTTGT-GGGACAAA----TCT------GGG----

-------------GTTCAAATCTCAG-C----------------C-AAA-AGACAACAGACAGA---G----

------------------TGA--C----GAA-CGTCTGG----------T-AA--AAATTTC-AGA-CCA--

--AAATACTC-------AAGGA-GT-----AAGGC---GC-----AATA------AG-TCCCAGA-CCGAGA

GCGAA-------------------------------------------------------------------

------------------------------------------------------------------------

--------------C--AAAACT--------------------------GG-----------------TTTT

C----------------------TAAAAACAAGA--C--GTCCTGGC-----TTTTCTGCTC----------

--------------CCTATC--TTCT-----------TTTTTTGA---------------------------

-------------------------TTT--TTTTAT-GGAC--GTG-CCT-CC-TTAGCT--GGGTGC--AA

AC-----------AACGT-A----TGAAAGT-ACTTGTG-T-------------GAA---------------

--TTTTTTCA------------------------------GCGCAAT-A-C-G-GA-CCC-AGA-----AT-

AAAATT--GCAGAAA--GTAGG------------GCGAAATTT--CACA-A-GTT-TTGG--TAGAG-----

---------GAT-AGCCT-------------------TGA-TTTGCTCA----AAATTTCTGAAAATTT-

>Pv01Sk01100/1-542 Pv01Sk01100 undefined product 1117991:1118532 reverse

-TCCCGAAGTAG-----TTTTGTCCTG--AAAATCCACGT-AAG-AA--A-------CT---CCACTG-AA-

TTTGGG-AC----------AAAACGAT-AC-AA-AT--TTCAGG--TC------------------------

-----------------------------------------AAAC-G--GATGAG---TATTCAC-------

---CCA-----TGAAAAAAAA--TA-AAACAGTT-TT--------------------------CCA-CAC--

AAAC--G-AA-CCT------------TCC--T-TTC-------A-G--TC-C-T-GA-C---------A--G

TGAAG-AAT-A--A-A-AA--A-TT-TA-TTGTC-AATCTTGT-GGGACGAA----TTT------GGC----

-------------GCTCAAATCTCAC-C----------------C-AAA-ACACAACAGACACA---G----

------------------TGA--C----GAA-CGTCTGG----------T-AA--AAATTTC-AGA-CCA--

--AAATACCC-------AAGGA-CT-----AAGGC---GT-----AGTT------AG-TCCCATA-CCGAGG

GCGAA-------------------------------------------------------------------

------------------------------------------------------------------------

--------------C--AAAACT--------------------------GG-----------------TTTT

C----------------------CGAAAACAAGA--C--GTCCTGGC-----TTTTCTTCCC----------

--------------CGTATC--TTCT-----------CTTTGTGA---------------------------

-------------------------TTT--GTTTAT-GGAC--GTG-TCT-CC-TTACCT--GGGTGC--AA

AC-----------AACGT-A----TGAAAGT-ACTTGTG-T-------------GAA---------------

--TTTTTTCA------------------------------GCGCAAT-A-C-G-GA-CCC-AGA-----AT-

AAAATT--GCAGAAA--GTAGG------------GCGAAATTT--CACA-A-GTT-TTCG--TAGAGGA---

---------TAT-AGCCT-------------------TCG-TTTGCACA----AAATTTCTGAAAAATTC

>Pv01Sk01140/1-536 Pv01Sk01140 undefined product 1239301:1239836 forward

--CTTAGAATAG-----TTTTTTCCTG--AAATTCCACAT-AAG-AA--T-------CT---TGACTG-AA-

TTTGGG-AC----------AAAACGCG-AC-AA-AT--TTCAGG--TC------------------------

-----------------------------------------AAAC-G--GATGAG---TATT-AT-------

---CTA-----GGAAAAAAA---TC-AAACAGTT-TC--------------------------ACA-CAC--

AAAT--G-AA-CAT------------TCC--T-TTT-------C-A--CT-C-T-AG-C---------A--G

TGATG-AAT-A--A-A-AA--A-AT-T--TTGCA-AATCCTGT-GGGACGAA----TTT------GGG----

-------------GTTGAAATCTCAA-C----------------C-AAA-ACTCAATAGACACA---A----

------------------TGA--A----AAA-TGTCTGG----------T--A-GAAATTTC-AGA-CAA--

--AAATACCC-------AAGGA-GT-----AAGGC---GT-----AGTA------AG-TCCTAGA-CCGAGA

GTGAA-------------------------------------------------------------------

------------------------------------------------------------------------

--------------C--AAATCT--------------------------AG-----------------TTTT

T----------------------CGAAAACAAAA--C--GTTCTGGC-----TTTTCTTCCT----------

--------------CGTATC--TTCT-----------TTTTGTGA---------------------------

-------------------------TTT--GTTTAG-GGAC--GTG-CCT-CC-TTACCT--GGGTGC--AA

AC-----------AACAT-A----TGAAAGT-ACTTGTG-T-------------GAA---------------

--TTTTTTCA------------------------------ATGCAATAC-G-G-AC-CCA-GAA-----AT-

AAAATT--GCAGAAA--GTATG------------GTGAAATTT--CAAA-A-ATT-TCAG--TAGAG-----

----------AT-AGCCT-------------------TGG-TTTTCGAT---AAAATTTCTGAAAAAAT-

>Pv01Sk01150/1-541 Pv01Sk01150 undefined product 1239838:1240378 forward

-TTCTGGAATAG-----TTATTTCCTG--AAATTTAACGT-AAG-AA--T-------CT---CGACTA-AA-

TTAGGG-AC----------AAAATGCG-AC-AA-AT--TTCAGG--TC------------------------

-----------------------------------------AAAC-A--GATGAG---TATTCAT-------

---CTA-----GGAAATAAA---TA-AAACAGTT-TC--------------------------ACA-CAC--

AAAC--G-AA-CCT------------TAA--T-TTC-------A-A--CC-C-T-GG-C---------A--G

TGATG-AAT-A--A-A-AT--A-TT-TA-TTGCA-AATCTTGT-GGAACGAA----TTT------CGG----

-------------GCTCAAATCTCAT-C----------------C-AAA-ACTCCATAGACACA---A----

------------------TGA--C----GAA-TGTCTGC----------T--A-AAAATTTC-ATA-CTA--

--AAATTTCC-------AAGGA-GT-----AAGGC---GT-----CGTA------AG-TCCCAGA-CCGAGA

GTGAA-------------------------------------------------------------------

------------------------------------------------------------------------

--------------C--ATAACT--------------------------GG-----------------TTTT

T----------------------TGAAAAAAAAAG-C--GTCCTGAT-----TTTTCTTTCC----------

--------------CGTATC--CTCT-----------TTTTGTGA---------------------------

-------------------------ATT--GTTTAT-GCAC--GTG-CCT-CC-TTACTT--GGGTAC--AA

AC-----------AACAT-A----TGAAAGT-ACTTGTG-T-------------GAA---------------

--TTTTTTCA------------------------------ACGCAAT-A-C-C-GA-CGT-AGG-----AT-

AAAATT--GCAAAAA--GTATG------------ACGAAATTT--GACA-A-GTT-TCGG--TAGAT-----

---------GAT-AGACT-------------------TGG-TTTTCAAT---AAAATTTCTGAAAAATTC

>Pv01Sk01160/1-539 Pv01Sk01160 undefined product 1240380:1240918 forward

--CATGGAATAG-----TTTTCTCCTG--AAATTCAACGT-AAG-AA--T-------CT---CGACTG-AA-

TTTAGG-AC----------AAAATGCG-AC-AA-AT--TTCAGG--TC------------------------

-----------------------------------------AAAC-A--AATGAG---TATTCAT-------

---CTA-----GGAAAAAAA---TA-AAACAGTT-TC--------------------------ACA-CAC--

AAAC--G-AA-CCT------------TAA--T-TTC-------A-G--CC-C-T-AG-C---------A--G

TGATG-AAT-A--A-A-AA--A-TT-TA-TTGCA-AATCTTGT-GGGACGAA----TTT------GGG----

-------------GCTCAAGTCTCAG-C-----------------AAAA-GCTCAATAGAAAAA---A----

------------------TGA--C----GAA-TGTCAGG----------T--A-AATATTTC-AGA-CCA--

--AAATACCC-------AAGGA-GT-----AAGGC---GT-----AGTA------AG-TCACAGA-CCGAGA

GTGAA-------------------------------------------------------------------

------------------------------------------------------------------------

--------------C--AAAACT--------------------------GG-----------------TTTT

T----------------------CGAAAACAAAA--C--GTCATGGA-----TTTTCTTCCC----------

--------------CGTATC--TTCT-----------TTTTTTGA---------------------------

-------------------------TTT--GTTTAT-GGAT--GTG-CCT-CC-TTACCT--GGATGC--AA

AC-----------AACAT-A----TGAAAGT-ACTTGTG-T-------------GAT---------------

--TTTTTTCA------------------------------ACGCAAT-A-C-G-GA-CCC-AGA-----AT-

AAAATT--GCAAAAA--AAAGG------------GCGTAATTT--CACA-A-GCT-TCTG--TGGAG-----

---------GAT-AGCCT-------------------TGG-TTTTCTAT---AAAATTTCTGAAAAATTC

>Pv01Sk01170/1-540 Pv01Sk01170 undefined product 1240918:1241457 forward

CTCCCGAAATAA-----TTTTATCCAA--AAATTAAACGT-AAG-AA--T-------CT---CAATTG-AA-

TTTGGG-AA----------AAAACGCG-AC-AA-AT--TTCATA--TC------------------------

-----------------------------------------AAAC-G--GATGAG---TATTCAC-------

---CTA-----GGAAAAAAA----CAAAACTG-T-TC--------------------------ACA-CAC--

AAAC--G-AA-CCT------------TCC--T-TTC-------A-G--CC-C-T-GG-C---------A--G

TGATG-AAT-A--C-A-AA--A-TT-TA-TTCCA-AATTCTGT-GAGACGAA----TTT------GTG----

-------------TCTCAAATATCAT-C----------------C-AAA-ACTCAACTGACACA---A----

------------------TGA--T----TAA-GGTTTGG----------T--A--AAAGTTTCAGA-TGA--

--AAATACCC-------AATGA-GT-----AAGGC---GT-----AGTA------AG-TCCCACA-CCGAGA

GTGAA-------------------------------------------------------------------

------------------------------------------------------------------------

--------------C--AAAACT--------------------------GT-----------------ATTT

T----------------------CGAAAACAAAA--C--GTCCTGAC-----TTTTTTTTAC----------

--------------CGTATC--TTTT-----------TTTTCTGA---------------------------

-------------------------TTT--GTTTAT-GGAT--TTG-CCT-CC-TTACCT--GTGTCA--CA

AC-----------AACAT-A----TGAAAGT-ACTTGTG-T-------------GAT---------------

--TTTTTCCA------------------------------ACGAAAT-A-C-G-AA-CCC-ATA-----AT-

AAAATT--GCAAAAA--ATAGG------------GCAAAATTT--CACA-A-GTT-TCGG--TAGAA-----

---------GAT-AGGCT-------------------TGG-TTTTCTAT---GAAATTTCAGAAAAATTC

>Pv01Sk01180/1-545 Pv01Sk01180 undefined product 1241458:1242002 forward

-TCCCAGGATAT-----TTTTTTCTTA--AAATTCCACGT-AAG-AA--T-------CT---CAACTG-AA-

TTTCCG-AC----------AAAATACA-AA-AA-AT--TTCAGG--TC------------------------

-----------------------------------------AAAC-G--GGTGAG---TATTCAC-------

---CTA-----GAAAAAAAA---TC-AAACAGTT-TC--------------------------ACA-CGC--

AAAC--G-AA-CTT------------TCC--T-TTC-------A-G--CA-C-T-GG-C---------A--G

TGACG-AAT-A--A-A-AA--A-TT-TA-TTGCC-AATCTTGT-GGGACGAA----TCT-------GG----

-------------GCTCAAATCTCAG-T----------------C-AAA-ACACAACCGACACA---G----

------------------TGA--C----GAA-CGTCTGG----------T-AA--AAATTTC-AGA-CCA--

--AAATACCC-------AAGGA-GT-----AAGGC---GT-----AGTA------AG-TCCCAGA-CCGAGA

CCGAA-------------------------------------------------------------------

------------------------------------------------------------------------

--------------C--AAAACT--------------------------GG-----------------TTTT

C----------------------CGAAAACAAGA--C--GTCTTGACTTTTCTTTTCTTCCC----------

--------------CGTATC--TTCT-----------CTTTGTTA---------------------------

-------------------------TTT--TTTTAT-GGAC--GTG-CCT-CC-TTGACT--GGGTGC--AA

AC-----------AACGT-A----TGAAAGT-ACTTGTG-T-------------GAA---------------

--TTTTTTCA------------------------------GTGCAAT-A---G-GA-CCC-AGA-----CT-

AAAATT--GCAGAAA--GTAGG------------GCGAAATTT--CACA-A-GTT-TTCG--TAAAGGAT--

---------GAT-AGCCT-------------------TGG-TTTGCACA----AAATTTCTGAAAAATTC

>Pv01Sk01190/1-540 Pv01Sk01190 undefined product 1242002:1242541 forward

CTCCCGAAATAG-----TTTTTTCCTG--AAATTCCACGC-AAG-AA--T-------CT---CCACTG-AA-

TTTGGG-AC----------AAAACGCG-AC-AA-AT--TTCAGG--TG------------------------

-----------------------------------------AAAC-G--GATGAG---TATTCAC-------

---CCA-----CGAAAAAAA---TC-AAATAGTT-TC--------------------------GCA-CAC--

AAAC--G-AA-CCT------------TCC--T-TTC-------A-G--CC-T-T-GG-C---------A--G

TGATG-AAT-A--A-A-AA--A-GT-TA-TTGCC-AATCTTGT-GGGACGAA----TCT------GGG----

-------------GCTCAGATCTCAG-C----------------C-AAA-ACACAACAGACACA---G----

------------------TGT--C----GAA-CTTCTGG----------T-AA--AATTTTC-AGA-CCA--

--AAATACCC-------AAGAA-TT-----AAGGC---GT-----AGTA------AG-TCCCAGA-TCGAGG

GCGAA-------------------------------------------------------------------

------------------------------------------------------------------------

--------------T--AAAACT--------------------------GG-----------------TTTT

C----------------------CAAAAACAAGA--C--GTCCTGAG-----TTTTCTTCCC----------

--------------CGTATA--TTCT-----------CTTTCTGT---------------------------

-------------------------TTT--GTTTAT-GGAT--GTG-CCT-CC-TTACCT--GGGTGC--AA

AC-----------AACGT-A----TGAAAGT-ACTTGTG-T-------------GAA---------------

--TTTTTTCA------------------------------GCGCAAT-A-C-G-GA-CCC-AGA-----AT-

AAAATT--GCAGAAA--GTATG------------GCGAAATTT--CACA-A-CTT-TTCG--TAAAT-----

---------GAT-AGCCT-------------------TGG-TTTGCACA----AAATTTCTGAAAAATTC

>Pv01Sk01200/1-530 Pv01Sk01200 undefined product 1242541:1243070 forward

CTCCCGAAATAG-----TTTTTTCCTG--AAATTCCACGC-AAG-AA--T-------CT---CCACTG-AA-

TTTGTG-AC----------AAAACGCG-AC-AA-AT--TTCAGG--TC------------------------

-----------------------------------------AAAC-A--GATGAG---CATTCAC-------

---CCA-----CGAAAAAAA---TC-AAACAGTT-TC--------------------------GCA-CAC--

AAAC--G-AA-CCT------------TCC--T-TTC-------A-G--CC-C-T-GG-C---------A--G

TGATG-AAT-A--A-A-AA--A-TT-TA-TTGTC-AATCTTGT-GGGACGAA----TCT------GGG----

-------------GCTCAAATCTCAG-C----------------C-AAA-ACACAACAGGCACA---G----

------------------TGA--C----GAA-CGTCTGG----------T-AA--AAATTTT-AGA-CCA--

--AAATACCC-------AAGGA-GT-----AAGGC---GT-----AGTA------AG-TCCCAGA-CCGAGA

GCGAA-------------------------------------------------------------------

------------------------------------------------------------------------

--------------C--AAAACT--------------------------GA-----------------TTTT

C----------------------CGAAAACACAA--C--GTCCTGGCTTTTCTTTTCTTCCC----------

--------------CATATC--TTCT-----------CTTTGTAA---------------------------

-------------------------TTT--GTTTAT-GGAC--GTG-TCT-CC-TTACCT--GGGTGC--AA

AC-----------AACGT-A----TGAGAGT-ACTTGTG-T-------------GAA---------------

--TTTTTTCA------------------------------GC--------------------AA-----AT-

AAAATT--GCAGAAT--GTAGG------------GCGAAATTT--CACA-A-GTT-TTCG--TTGAT-----

---------GAT-AGCCT-------------------TGG-TTTGCACA----AAATTTCTGAAAAATT-

>Pv01Sk01210/1-525 Pv01Sk01210 undefined product 1243072:1243596 forward

-TCCCGAAATAG-----TTTTTTCCTG--AAATTCCATGC-AAG-AA--T-------CT---CCACTG-AA-

TTTGGG-AC----------AAAACGCG-AC-AA-AT--TTCAGG--TC------------------------

-----------------------------------------AAAC-G--AATGAG---TATTCAC-------

---CCA-----CGAAAAAAA---TC-AAACAGTT-TC--------------------------GCA-CAC--

AAAC--G-AA-CCT------------TCC--T-TTC-------A-G--CC-C-T-GG-C---------A--G

TGATG-AAT-A--T-A-AA--A-CT-TA-TTGCA-AATCTTGT-GGAACGAA----TCT------AGG----

-------------GACCAAATCTCAG-C-----------------AAAA-ACACAACAGACACA---G----

------------------TGT--C----GAA-CGTGTGG----------T-AA--AACTTTC-AGA-CCA--

--AAATACCC-------AAGGA-GG-----AAGGC---GT-----AGTA------AG-TCCTAGA-CCGAGA

GCGAA-------------------------------------------------------------------

------------------------------------------------------------------------

--------------C--AAAACT--------------------------TG-----------------TTTT

C----------------------CGAAAACAAGA--C--GTCCTGGT-----TTTTCTTTCC----------

--------------CGTATC--TTCT-----------CTTTGTGA---------------------------

-------------------------TTT--GTTTAT-GGAC--GTG-CCT-CC-TTACCT--GGGTGC--AA

AC-----------AACGT-A----TGAAAGT-ACTTGTG-T-------------GAA---------------

--TTTTTTCA------------------------------GC--------------------GA-----AT-

AAAATT--GCAGAAT--GTAGA------------GCGAAATTT--CAAA-G-GTT-TTCG--TTGAG-----

---------AAT-AGCCT-------------------TGG-TTTGCACA----AAATTTATGAAAAATTC

>Pv01Sk01220/1-540 Pv01Sk01220 undefined product 1243596:1244135 forward

CTCCCGAAATAG-----TTTTTTCCTG--AAATTCCACGC-AAG-AA--T-------CT---CCACTG-AA-

TTTGGG-AC----------AAAACGCG-AC-AA-AT--TTCAGG--TC------------------------

-----------------------------------------AAAC-A--GATGAA---TATTCAC-------

---CCA-----CGAAAAAAA---TC-AAACAGTT-TC--------------------------GCA-CAC--

AAAC--G-AA-CCT------------TCC--T-TTC-------A-G--CC-C-T-GG-C---------A--G

TGATG-AAT-A--A-A-AA--A-CT-TA-TTGCA-AATCTTGT-GGGACGAA----TCT------GGG----

-------------GACCAAATCTCAG-C----------------C-AAA-ACACAACAGACACA---G----

------------------TGT--C----GAA-CGTGTGG----------T-AA--AACTTTC-AGA-CCA--

--AAATAACC-------AAGGA-GG-----AAGGC---GT-----AGTA------AG-TCTCAGA-CCGAGA

GTGAA-------------------------------------------------------------------

------------------------------------------------------------------------

--------------C--AAAACT--------------------------GG-----------------TTTT

C----------------------CGAAAACAAGA--C--GTCCTGGT-----TATTCTTTCC----------

--------------CGTTTC--TTCT-----------CTTTGTTA---------------------------

-------------------------TTT--GTTTAT-GGAC--GTG-CCT-CC-TTACTT--GGGTGC--AA

CC-----------AACGT-A----TGAAAGT-ACTTGTG-T-------------GAA---------------

--TTTTTTCA------------------------------GCGCAAT-A-C-G-GA-CCC-AGA-----AT-

AAAATT--GTAGAAA--GTATG------------GCGAAATTT--CACA-A-GTT-TTCG--TACAG-----

---------GAT-AGCCT-------------------TGG-TTTGCACA----AAATTTCTAAAAAATTC

>Pv01Sk01230/1-542 Pv01Sk01230 undefined product 1244135:1244676 forward

CTCCTCAAATAC-----TTTTTTCCTG--AAATTGCACGC-AAG-AA--T-------CT---CCACTG-AA-

TTTGGG-AC----------AAAACTTG-AC-AA-TT--TTCAGG--TA------------------------

-----------------------------------------AAAC-G--GATGAG---TATTCAC-------

---CCA-----CACAAAAAA---TC-AAACAGTT-TC--------------------------GCA-CAC--

AAAC--A-AA-CCT------------TCC--T-TTC-------A-G--CC-C-T-GG-C---------A--G

TGATG-AAT-A--A-A-AA--A-TT-TA-TTGTC-AATATTGT-GAGACGAA----TCT------AGG----

-------------GCTCAAATCTCAG-C----------------C-AAA-ACACAATAGAAACA---G----

------------------TGA--C----GAA-CGTCTGT----------T-AA--AAATTTC-AGA-CCA--

--AAATACCC-------AAGGA-GT-----AAGGC---GT-----AGTA------AC-TCCCAGA-CCGAGA

GCGAA-------------------------------------------------------------------

------------------------------------------------------------------------

--------------G--AAAACT--------------------------GG-----------------TTTT

C----------------------CGAATACAAGA--C--GTCCTGAT-----TTTTCTTCCC----------

--------------CGTATC--TTCT-----------CTTTGTGA---------------------------

-------------------------TTT--GTTTAT-GGAC--GTG-CCT-CC-TTACCT--GGGTGC--AA

AC-----------AACGT-A----TGAAAAT-ACTTGTG-T-------------GAA---------------

--TGTTTTCA------------------------------ACGCAAT-A-C-A-AA-CCC-AGA-----AT-

AAAATT--GCATAAA--GTATG------------GCAAAATTT--CACA-A-GTT-TTCG--TGGAGGA---

---------CAT-AGCCT-------------------TGG-TTTACACA----AAATTTCTGAAAAATTC

>Pv01Sk01250/1-540 Pv01Sk01250 undefined product 1303930:1304469 reverse

CTCACGAAATAA-----TTTTTTCTTG--AAATTCCATGC-ATG-AA--T-------CT---CCACTG-AA-

TTTGGG-AC----------AAAACGCG-AC-AA-AT--TTCAGG--TC------------------------

-----------------------------------------AAAC-G--GATGAG---TATTCAA-------

---CCA-----TGAAAAAAA---TC-AAACAGTT-TC--------------------------GCA-CAC--

AAAC--G-AA-CCT------------TCC--T-TTC-------A-G--AC-C-T-GG-C---------A--G

TGAAG-AAT-A--A-A-AA--A-TT-TA-TTGCC-AATCTTGT-GGGACGAA----TCT------GGG----

-------------GCTCAAGTCTCAG-C----------------C-AAA-ACACAACAGACACA---G----

------------------TGA--A----GAA-CGTCTGG----------T-AA--AAATTTA-AGA-CCA--

--AAATACCC-------AAGGA-GT-----AAGGC---GT-----AGTA------AG-TCCCAGA-CCGAGA

GCGAA-------------------------------------------------------------------

------------------------------------------------------------------------

--------------C--AAAACT--------------------------GG-----------------TTTT

C----------------------TAAAAACAAGA--C--GTCATGGC-----TTTTCTTCCC----------

--------------CGTATC--TTCT-----------CTTTGTGA---------------------------

-------------------------TTT--GTTTAT-GGAC--GTG-CCT-CC-TTACCT--GGGTGC--AA

AC-----------AACGT-A----CGAAAGT-ATTTGTG-T-------------GAA---------------

--TTTTTTCA------------------------------CGGCAAT-A-C-G-GA-CCC-AGA-----AT-

AAAATT--GCAGAAA--GTAGG------------GCGAAATTT--TACA-A-GTT-TTCG--TAGAG-----

---------GAT-AGCCT-------------------TGG-TTTGCACA----AAATTTCTAAAAAATTC

>Pv01Sk01260/1-539 Pv01Sk01260 undefined product 1304469:1305007 reverse

-CCCCGAAATAG-----TTTTTTCCTG--AAATTCCACGC-AAG-AA--T-------TT---CCACTG-AA-

TTTGGG-GC----------AAAACGCA-AC-AA-AT--TTCATG--TC------------------------

-----------------------------------------AAAC-G--GATGAG---TATTCAA-------

---CTA-----TGAAAAAAA---TC-AAACAGTT-TC--------------------------GCA-CAC--

AAAC--G-AA-CCT------------TCC--T-TTC-------A-G--AC-C-T-GG-C---------A--G

TGAAG-AAT-A--A-A-AA--A-TT-TA-TTGCC-AATCTTGT-GGGACGAA----TCT------GGG----

-------------GCTCAAGTCTCAG-C----------------C-AAA-ACACAACAGACACA---G----

------------------TGA--A----GAA-CGTCTGG----------T-AA--AAATTTA-AGA-CCA--

--AAATACCC-------AAGGA-GT-----AAGGT---GT-----AGTA------AG-TCCCAGA-CCTAGA

GCGAA-------------------------------------------------------------------

------------------------------------------------------------------------

--------------C--AAAACT--------------------------GG-----------------TTTT

C----------------------CAAAAACAAGA--C--GTCCTGGC-----TTTTCTTCCC----------

--------------CGTATC--TTCT-----------CTTTGTGA---------------------------

-------------------------TTT--TTTTAT-GGAC--TTG-CCT-CC-TTACCT--GGGTGC--TA

AC-----------AACGT-A----CGAATGT-ACTTCTG-T-------------GAA---------------

--TTTTTTCA------------------------------GCGCAAT-A-C-G-AA-CCC-AGA-----AT-

AAAATT--GCAGAAA--GTAGG------------GCGAAATTT--CACA-A-GTT-TTCG--TAGAG-----

---------GAT-AGCCT-------------------TGG-TTTGCACA----AAATTTCTGAAAAATTC

>Pv01Sk01270/1-539 Pv01Sk01270 undefined product 1305008:1305546 reverse

CTCCCGAAATAG-----CATTTTCCTG--AAATTCTACGA-AAG-AA--T-------CT---CCACTG-AA-

TTTGGG-AC----------AAAACGCG-AC-AA-AT--TTCAGG--TC------------------------

-----------------------------------------AAAC-G--GATGAG---TATTCAC-------

---CCA-----CGAAAAAAA---TC-AAACAGTT-TC--------------------------GCA-CAC--

AAAC--G-AA-CCT------------TCC--T-TTC-------A-G--CC-C-T-GG-C---------A--G

TGATG-AAT-A--A-A-AA--A-TT-TA-TTCTC-AATCTTGT-GGGACGAA----TTT-------GG----

-------------GCTCAAATCTCAG-C----------------C-AAA-ACACAACAGACACA---G----

------------------TGA--A----GAA-CGTCTGG----------T-AA--AAATTTC-AGA-CCA--

--AAATACCA-------AAGGA-GT-----AAGGC---GT-----AGTA------AG-TCCCAGA-CCAAGA

GCGAA-------------------------------------------------------------------

------------------------------------------------------------------------

--------------C--AAAACT--------------------------GG-----------------TTTT

T----------------------CAAAAACAAGA--C--GTCCTGGC-----TTTTCTTCCC----------

--------------CGTATC--TTCT-----------CTTTGTGA---------------------------

-------------------------TTT--TTTTAT-GGAC--TTG-CCT-CC-TTACCT--GGGTGC--TA

AC-----------AACGT-A----CGAATGT-ACTTGTG-T-------------GAA---------------

--TTTTCTCA------------------------------GCGCAAT-A-C-G-GA-CCC-AAA-----AT-

AAAGTT--GCAGAAA--GTAGG------------ACGAAATTT--TAAA-A-GTT-TTCG--TAGAG-----

---------GAT-AGCCT-------------------TGG-TTTGCACA----AAATTTCTAAAAAATTC

>Pv01Sk01280/1-530 Pv01Sk01280 undefined product 1503649:1504178 reverse

CTCCCAAAATAG-----TTTTTTCCTG--AAATTGCATGT-AAG-AA--T-------CT---TGACTG-AA-

TTTGGA-AC----------AAAACGCA-AC-AA-AT--TTCAGG--TC------------------------

-----------------------------------------AAAC-A--AATGAG---TATTCAC-------

---CTA-----GGAAAAAAA---TC-AAACAGTT-TC--------------------------AAA-CAC--

AAAC--G-GA-CCT------------TCC--T-TTC-------A-G--CC-C-T-GG-A---------A--G

TGATG-AAT-A--A-A-AA--A-TT-TA-TTGAA-AATCTTGT-GGGACGAA----TTT------GGT----

-------------GCTCAAATCTCAG-C----------------C-AAA-ACTCAATAGACACA---A----

------------------TGA--C----GAA-TGTCTGG----------T--A-AAATTTTT-ATA-CCA--

--AAATATCC-------AAGGA-GT-----AAGAT---GT-----AGTA------AG-TCCCAAA-CCAAGA

GTGAA-------------------------------------------------------------------

------------------------------------------------------------------------

--------------C--AAAACT--------------------------GG-----------------TTTT

C----------------------CGAAAACAAAA--C--GTCCTATC-----TTTTCGTCCT----------

--------------TGTATC--TTCT-----------TTTTGTGA---------------------------

-------------------------TTT-CGTTTAT-GGAC--ATG-TCT-CC-TTACCT--AGGTGC--AA

AC-----------AACAT-A----TGAAAGT-ACTTGTG-T-------------GAA---------------

--TTTTTTC-------------------------------------T-A-C-G-GA-CCC-AGA-----AT-

AAAATT--GCAGAAA--GTATG------------ACGAAATTT--TGCA-A-GTT-TCGG--TAAAG-----

---------GAT-A-CCT-------------------TGG-TTTGCACA----AAATTTTTGAAAAA---

>Pv01Sk01290/1-539 Pv01Sk01290 undefined product 1504178:1504716 reverse

-TTCCGAAATAG-----TTTTTTCCTA--AAATTCCAGGT-AAG-AA--T-------CT---CGACTG-AA-

TTTGCG-AA----------AAAACTTG-AT-AA-AT--TTTAGG--TC------------------------

-----------------------------------------CAAC-G--GATGAA---TATTCAC-------

---TTA-----GGAAATAAA---TC-AAACAGTT-TC--------------------------ACA-AAC--

AAAC--A-GA-CCT------------TCC--T-TTC-------A-G--CC-C-T-GG-C---------A--G

TGATG-AAT-A--A-A-A---G-TT-TA-TTGAA-AATTTTGT-GGGATGAA----TTT------GGG----

-------------ACTCAAATCTCAG-T----------------C-AAA-ACTCAATAGACATA---A----

------------------TGA--C----GAA-TGTCTGG----------T--A-AAAATTTC-ATA-CCA--

--AAATACCC-------AAGGA-TT-----AAGGC---GC-----AATG------AG-TCTCAGA-CCGAGA

GTGAA-------------------------------------------------------------------

------------------------------------------------------------------------

--------------T--AAAACT--------------------------GG-----------------TTTT

C----------------------CGAAAACAAAA--C--ATCCTAGC-----TTTTCCTCCC----------

--------------TGTATC--TTCT-----------TTTTGTCA---------------------------

-------------------------TTT--GTTTAT-GAAC--TTG-TCT-CC-TTACCT--AGGTGC--AA

AC-----------AACAT-A----TGAAAGT-ACTTGAG-T-------------GAA---------------

-TTTTTTTCA------------------------------ACGCAAT-A-C-G-GA-CCC-AAA-----AT-

AAAATT--GCAGAAA--GTATG------------GGAAAATTT--CGCA-A-GTT-TCAG--TAGAG-----

---------GAT-AGCCT-------------------TTG-TTTGCACA----AAATTTGTGAAAAATTC

>Pv01Sk01300/1-536 Pv01Sk01300 undefined product 1504717:1505252 reverse

-TACCGAAATAG-----TTTTTTCCTA--AAATTCCAGGT-AAG-AA--T-------CT---CAACTG-AA-

TTTGCG-AC----------AAAACGCG-AC-AA-AT--TTCGGG--TC------------------------

-----------------------------------------AACT-G--GATTAC---TAATCAC-------

---CTA-----GGGAAAAAA---TA-AAACAGTT-TC--------------------------GCA-CAC--

AAAC--A-AA-CAT------------TCC--T-TTC-------A-G--CC-A-T-GA-C---------A--G

TGATG-AAA-A--A-A-A------C-CA-TTGCA-AATATTGT-GGAACGAA----TTT------GGG----

-------------GCTCAAATCTCAA-C----------------C-AAA-ACTCAATAGACACA---A----

------------------TGA--C----GAA-TGTCTGG----------T--A-AAAATTTC-AGA-CCA--

--AAATACGC-------AAGGA-GT-----AAGGA---GT-----AGTA------AA-TCTCTGA-CCAAGA

GTGAA-------------------------------------------------------------------

------------------------------------------------------------------------

--------------C--AAAACT--------------------------GG-----------------TTTT

C----------------------TGAAAATAAAA--C--GTCCTCGC-----ATTTCTTCCC----------

--------------CGTATC--TTCT-----------TTTTGTGA---------------------------

-------------------------TTT--GTTTAT-GGAC--CTG-CCT-CC-TTACCT--GGGTGC--AA

AC-----------AACAT-A----TGTAAGT-ACTTGTG-T-------------GAA---------------

--TTTTTTCT------------------------------ACGCAAT-A-C-G-GA-CCC-AGA-----AT-

AAAATT--GCAGAAC--GTAGG------------ACGAAATTT--CACA-A-GTT-TCGC--TAGAG-----

---------GAT-AGTCT-------------------TGG-TTTGCACA----AAATTTCTGAAAAATTC

>Pv01Sk01310/1-527 Pv01Sk01310 undefined product 1505253:1505779 reverse

-TACCGAAATAT-----TTTTTTCCTG--AAATTTCGGGT-AAG-AA--T-------CT---CTACTG-AA-

TTTGGG-AC----------AAAACACG-AC-AA-AT--TTTTTG-CTC------------------------

-----------------------------------------AAAC-A--AATGAG---TATTCAC-------

---CTA-----GAAAAAAAA---GC-AAACAGTT-TC--------------------------ACA-TAC--

ATAT--A-AA-CCT------------GCC--T-TTC-------A-G--CT-C-T-GG-C---------A--G

TGATG-AAT-A--A-A-AA--G-TT-TA-TTGCA-AATCTTGT-GGGACGAA----TTT------GGG----

-------------GCTCAAATCTTAG-C----------------C-AAA-ACTCAATAGACACA---A----

------------------T---------------------------------G-AAAATTCA-AAA-CCA--

--AAATACCC-------AAGGA-GT-----AAGGA---GG-----AGTA------AA-TTTCAGA-CCAAGA

GTGAA-------------------------------------------------------------------

------------------------------------------------------------------------

--------------C--AATACT--------------------------AG-----------------TTTT

C----------------------CAAAAACAAAA--C--GTCCTCGC-----ATTTCTTCAC----------

--------------TGTATC--CTTT-----------TTTTGGGA---------------------------

-------------------------TTT--GTTTAT-GGAC--CTG-CCT-GC-TTACCT--GGGGGC--AA

AC-----------AACAT-A----TGAAAGT-ACTTGTG-T-------------GAA---------------

-TTTTTTTCA------------------------------ACGCAAT-A-C-G-GA-CCC-AAA-----AT-

AAAATT--GCAGAAT--GTAGG------------GCGAAATTT--CACA-A-GTT-TCGG--TAAAG-----

---------GAT-AGCCT-------------------TGA-TTTGCACA----AAATTTGTAAAAAATTC

>Pv01Sk01320/1-539 Pv01Sk01320 undefined product 1505780:1506318 reverse

--TCCGAAATAG-----TGTTTTCCTG--AAATTGCAGGT-AAG-AA--T-------CT---CGATTT-AA-

TTTGGG-AC----------AAAACACG-AT-AA-AT--TTCAGG--TA------------------------

-----------------------------------------AAAC-A--AATGAG---TATTCAC-------

---CTA-----AGAAAAAAA---TC-AAACAGTT-TC--------------------------ACA-AAC--

AAAC--A-GA-CCT------------TCC--T-TTT-------A-G--CC-C-T-GG-C---------A--G

TGATG-AAT-A--A-A-AA--G-TT-TA-TTGCA-AATCTTGT-AGGACGAA----TTT------GGG----

-------------GCTCAAATCTTAG-C----------------C-AAA-ACTCAATAGACAGA---A----

------------------TGA--T----GAA-TGTCTGG----------TA-A-AAAAATTC-ATA-CCA--

--AAATACCC-------AAGGA-AT-----AAGGC---GC-----AGTA------AA-TCCAAGA-CTGAAA

GTGAA-------------------------------------------------------------------

------------------------------------------------------------------------

--------------C--GAAAGT--------------------------GG-----------------TTTT

T----------------------CGAAAACAAAA--C--ATCATCGC-----TTCTCCTCCC----------

--------------CGTATA--ATCT-----------TTTTGTGA---------------------------

-------------------------TTT--GTTTAT-GAAC--CTG-CCT-CC-TTACAT--GGGTGC--AA

AA-----------AACAT-A----TGAAAGT-AATTGTG-T-------------GAA---------------

--TTTTTTCA------------------------------ACGCAAT-A-C-G-AA-CCC-ATA-----AT-

AAAATT--GCAGAAA--GTAGG------------GCGAAATTT--CACA-A-GTT-TCGG--TCGAG-----

---------GAT-AGTCT-------------------TGG-TTTGCAAA----AAAATTCTGAAAAATTC

>Pv01Sk01330/1-526 Pv01Sk01330 undefined product 1506330:1506855 reverse

--CCCGAAATAG-----TTTTTTCATG--AAATTCCAAGT-AAG-AA--T-------CT---CGACTG-AA-

TTTGGG-AC----------AAAACACG-AC-AA-AT--TTTTTG-CTC------------------------

-----------------------------------------AAAC-A--AATGAG---TATTTAC-------

---CTA-----GGAAAAAAA---TC-AAACAGTT-TC--------------------------ACA-TAC--

AAAC--G-GA-CCT------------TCC--T-TTC-------A-G--CC-C-T-GG-C---------A--G

TGATG-AAT-A--A-A-AA--G-TT-TA-TTGCA-AATCTTGT-AGAACGAA----TTT------AGG----

-------------GCTCAAATTTCAG-C----------------C-AAA-ACTCAATAGACACA---A----

------------------TGA--C----GAA-TGTCTGG----------T--A-AAAATTTC-ATA-CCA--

--AAATACCC-------AAGTA-GT-----AAGGC---GT-----AGTA------AA-TCTCAAA-CCGAGA

GTGAA-------------------------------------------------------------------

------------------------------------------------------------------------

--------------C--AAAACT--------------------------GG-----------------TTTT

C----------------------CGAAAACAAAA--C--GTCTTGGC-----TTTTCTTCCC----------

--------------CGTATC--TT-------------TTTTTTTA---------------------------

-------------------------TTT--GTTTAT-AGAT--CTG-CCA-CC-TTACCT--GGGTGC--AA

AC-----------AACAT-A----TGAAAGT-ACTTGTG-T-------------TAA---------------

--TTTTTTCA------------------------------ACGCAAT-A-C-G-GA-CTT-AGA-----AT-

AAAATT--GCAGAAA--GTATG------------GCGAAATTT--TGCA-A-GTT-TCAG--TAGAG-----

---------GAT-AACCT-------------------TTG-TATG---------------AAAAAAATTC

>Pv01Sk01350/1-541 Pv01Sk01350 undefined product 1508182:1508722 reverse

CTCCGGAAATAG----TTTTTTTCCTG--AAATTCCACCT-AAG-AA--T-------CT---CCACTG-AA-

TTTGGC-AC----------AAAACGCC-AC-AA-AT--TTCAGG--TC------------------------

-----------------------------------------AAAC-G--GATGAG---TATTCAC-------

---CCA-----CGAAAAAAA---TC-AAACAGTT-TC--------------------------GCA-CAC--

AAAC--G-AA-CCT------------TCC--T-TTC-------A-G--CC-C-T-GG-C---------A--G

TGATG-AAT-A--A-A-AA--A-TT-TA-TTGTC-AATCTCGT-GGGACGAA----TCA------GGG----

-------------GCTCAAATCTCAT-T----------------CAAAA-CTCAAT-AGACACA---C----

------------------TAT--C----GAA-CGTCTGG----------T--A--AAAATTTCAGA-CCA--

--AAATACCC-------GATGA-GT-----AAGGC---GT-----AGTA------AG-TCCCAGG-CCGAGA

GTGAA-------------------------------------------------------------------

------------------------------------------------------------------------

--------------C--AAAACT--------------------------GG-----------------TTTT

C----------------------CAAAAATAAAA--C--GTCCTGGC-----TTTTCTTCTT----------

--------------CGTATC--TTCT-----------TTTTGTGA---------------------------

-------------------------TTT--CTTTAT-GGAT--GTG-CCT-CC-TTACCT--GAGTGC--AA

AC-----------AACAT-A----TGAAAGT-ACTCGTG-T-------------GAA---------------

--TTTTTTCA------------------------------GCGCAAT-A-C-G-AA-CCC-ATA-----AT-

AAAATT--GCAGAAA--GTAGG------------GCGAAATTT--CAGT-T-GTT-TTGG--TAGAG-----

---------GAT-AGCCT-------------------TGG-TTTGCACA----AAATTTCTGAAAAATTC

>Pv01Sk01360/1-535 Pv01Sk01360 undefined product 1508726:1509260 reverse

CTCCGGAAATAG-----TTTTTTCCTG--TAATTCCACGT-AAG-AA--T-------CT---CCACTG-AA-

TTTGGG-AC----------AAAACGTC-AC-AA-AT--TTCAGG--TC------------------------

-----------------------------------------AAAC-G--GATGAG---TATTCAC-------

---CCA-----CGAAAAAAA---TC-AAACAGTT-TC--------------------------GCA-CAC--

AAAC--G-AA-CCT------------TCC--T-TTC-------A-G--CC-C-T-GG-C---------A--G

TGATG-AAT-A--A-A-AA--A-TT-TA-TTGCC-AATCTCGT-GGAACGAA----TCT------GGG----

-------------GCTCAAATCTCAG-C----------------CAAAA-TCAAAC-AGACAAA---C----

------------------TGG--C----GAA-CATCTGG----------T--A--AAACTTTCAGA-CCA--

--AAATACCC-------AAGGT-GT-----AAGGC---GT-----AGTA------AG-TCCCAGG-CCGAGA

GTGAA-------------------------------------------------------------------

------------------------------------------------------------------------

--------------C--AAAACT--------------------------GG-----------------TTTT

-----------------------CGAAAACAAAA--C--GTCTTGGC-----TTTTCTTCCC----------

--------------CGTATC--TTCT-----------TTTTGTGT---------------------------

-------------------------TTT--TTTTAC-GGAC--GTG-CCT-CC-TTACCT--GGGTGC--AA

AC-----------AACAT-A----TGAAAGT-ACTCGTG-T-------------GAA---------------

--TTTTTTCA------------------------------GCGCAAT-A-C-G-GA-CCT-AGA-----AT-

AAAATT--GCAGAAA--GTAGG------------GCGAAATTT--CACA-A-GTT-TTGG--TAGAG-----

---------GAT-AGCCT-------------------TGG-TTTGCACA----AAATTTCTGAAAA----

>Pv01Sk01370/1-539 Pv01Sk01370 undefined product 1509260:1509798 reverse

CTCCGGAAATAG-----TTTTTTCCTG--TAATTCCACGT-AAG-AT--A-------CT---CCATTG-AA-

TTTAGG-AC----------AAAACGCC-AC-AA-AT--TTCAGG--TC------------------------

-----------------------------------------AAAC-G--GATGAG---TATTCAC-------

---CCA-----CGAAAAAAA---TC-AAACAGTT-TC--------------------------GCA-CAC--

AAAC--A-AA-CAT------------TCC--T-TTC-------A-G--CT-C-T-GG-C---------A--G

TGATG-AAT-A--A-A-AA--A-TT-TA-TTGCC-AATCTCGT-GGGACGAA----TCT------GGG----

-------------GCTCAAATCTCAG-C----------------CAAAA-CTCAAC-AGACACA---C----

------------------TGA--C----GAA-CGTCTGG----------T-----AAAATTTCAGA-CCA--

--AAATACCC-------AAGGA-GT-----AAGGC---GT-----AGTA------AG-TCCCAGA-CCCAGA

GTGAA-------------------------------------------------------------------

------------------------------------------------------------------------

--------------C--AAAACT--------------------------GG-----------------TATT

C----------------------CGAAAACAAAA--C--GTCTTGGC-----TTTTCTTCCC----------

--------------CGTATC--TTCT-----------TTTTGTGA---------------------------

-------------------------TTT--GTTTAT-GGAC--GTG-CCT-CC-ATACCT--GAATGC--AA

AC-----------AACAT-A----TGAAAGT-ACTCGTG-T-------------GAA---------------

--TTTTTTCA------------------------------GAGCAAT-A-C-G-GA-CCC-AGA-----AT-

AAAATT--GCAGAAA--ATAAG------------GCGAAATTT--CACA-A-GTT-TTGG--TAATG-----

---------GAT-AGCCT-------------------TGG-TTTGCATA----AAATTTCTGAAAAATTC

>Pv01Sk01380/1-530 Pv01Sk01380 undefined product 1710126:1710655 reverse

CTCCCGGAATAG-----TTTTTTCCTG--AAATTCCACCC-ATG-AC--T-------CT---CCACTG-AT-

TTTTGGGAC----------AAAACGCG-AC-AT-AT--TTCAGG--TC------------------------

-----------------------------------------AAAC-G--GATGAG---TATTCAC-------

---CAA-----TGAAAAAAA---TC-AAACAGTT-TC--------------------------ACA-CAC--

AAAC--G-AA-CCT------------TCC--T-TTC-------A-A--CC-A-T-GA-C---------A--G

TGACG-AAT-A--A-A-AA--A-TT-TA-TTGCC-AATCTTGT-GGGACGAA----ATT------GGG----

-------------GCTCAAACCTCAG-C----------------CAAAA--ATCAATAGATACA---G----

------------------TGA--G----GAA-TGTCTGG----------C--A-AAAATTTC-AGA-CCA--

--AAATACCC-------AAGAA-GT-----AAGGC---GT-----AGTA------AG-TCCCAGT-CCGAGA

ATGAA-------------------------------------------------------------------

------------------------------------------------------------------------

--------------C--AAAACC--------------------------GG-----------------TTTT

C----------------------CGAAAACAAAA--T--GTCCTGGC-----TTTTCTTCCC----------

--------------CGTATC--TTCT-----------TTTTGTGA---------------------------

-------------------------TTC--GTTTAA-GGAC--GTG-CCT-CC-ATGCCT--GGGTGC--AA

AC-----------AACAT-A----CGAAAGT-GCTTGTA-T-------------GAA-TT------------

---TTTTTCA------------------------------GCGCAAT-A-C-G-GA-CCC-AGA-----AT-

GAAATT--GCTGAAA--TTAGG------------CCGGAATTT--CACA-A-CTT-TTTG--T---------

-------------------------------------TGG-TTTGCACA----AAATTTTTGAAAAATTC

>Pv01Sk01390/1-521 Pv01Sk01390 undefined product 1710656:1711176 reverse

---CCAGAATAA----GTTTTTTCCTG--AAATTCCACCC-AAG-AA--T-------CT---CCACTG-AA-

TTTTGG-AC----------AAAACGCG-AC-AA-AT--TTCAGG--TC------------------------

-----------------------------------------AAAC-G--GATGAG---TATTCAC-------

---CCA-----AGAAAAAAA---TC-AAACAATT-TC--------------------------ACA-GAC--

AAAC--G-AA-CCT------------TCT--T-TTC-------A-G--CC-C-T-GG-C---------A--G

T-ACG-AAT-A--A-A-AA--A-TT-TA-TTGTC-AATCTTGT-GCGACGAA----TCC------GGG----

-------------TCTCAAACCTCAG-C----------------CAAAA--CTCAATAGACACA---G----

------------------TGA--C----GAA-TGTCTGG----------T--A-AAAATTTC-AGA-CCA--

--AAATACCC-------AAGGA-GT-----AAGGC---GT-----AGTA------TG-TCCCAGA-CCAAGA

GTGAA-------------------------------------------------------------------

------------------------------------------------------------------------

--------------C--AAAACC--------------------------GA-----------------ATTT

C----------------------TGAAAACAAAA--C--GT-------------------------------

--------------CATATC--TTCT-----------TTTTGTAA---------------------------

-------------------------TTC--GTTTAT-GGAC--GTG-CCT-CC-TTGCCT--GCGTGC--AA

AC-----------AACAT-A----CGAAAGT-GCTTGTG-T-------------GAATTT------------

----TTTTCA------------------------------GCGCAAT-A-C-G-GA-CCC-AGA-----AT-

TAAATT--GCAAAAA--TTAGT------------CCGGAATTT--CACA-A-GTT-TCGG--TAGAG-----

---------GAT-AGCCT-------------------TGG-TTTGCACA----AAATTTCTGAAAAACT-

>Pv01Sk01400/1-522 Pv01Sk01400 undefined product 1711179:1711700 reverse

CTCCCGGAATAG-----TTTTTTCCTG--AAATTCCACCC-AAG-AC--T-------CT---CCACTG-AA-

TTTGGG-AC----------AAAACGCG-AC-AA-AT--TTCAGG--TC------------------------

-----------------------------------------AAAC-G--GATGAG---TATTCAC-------

---CCA-----CGAAAAAAA---TC-AAACAGTT-TC--------------------------ACA-CAC--

AAAC--G-AA-CCT------------TCC--T-TTC-------A-A--CC-C-T-GA-C---------A--G

TGACG-AAT-A--A-A-AC--A-TT-TA-TTGTC-AATCTTGT-GGGACGAA----TAT------GGG----

-------------GCTCAAACCTCAG-C----------------CAAAA--CTCAATAGACACG---G----

------------------TGA--C----GAA-TGTCTGG----------T--A-AAAATT-C-AGA-CCA--

--GAATACCC-------AAGGA-GT-----AAGGC---GT-----AGTA------AG-TCCCAGA-CCAAGA

GTGAA-------------------------------------------------------------------

------------------------------------------------------------------------

--------------C--AAAATC--------------------------GG-----------------TTTT

C----------------------CAAAAACAAAA--C--GT-------------------------------

--------------CGTATC--TTCT-----------TTTTGTGA---------------------------

-------------------------TTC--GTTTAT-GGAC--GTG-CCT-CC-TTGCCT--GGGTGC--AA

AC-----------AACAT-A----CGAATGT-GCTTCTG-T-------------GAATTT------------

----TTTTCA------------------------------CCGCAAG-A-C-G-GA-CCC-AGA-----AT-

GAAATT--GCTGAAA--TTAGG------------CCGGAATTT--CACA-A-GTT-TCGG--TAGAC-----

---------GAT-AGCCT-------------------TGG-TGTGCACA------ATTTCTGAAAAATTC

>Pv01Sk01410/1-538 Pv01Sk01410 undefined product 1711701:1712238 reverse

CTCCCGGAATAG-----TTTTTTCCTG--AAATTCCACCC-AAG-AA--T-------CT---CCACTG-AA-

TTTGGG-AC----------AAAACGCG-AC-AA-AT--TTCACG--TC------------------------

-----------------------------------------AAAT-T--GATGAG---TATTCAC-------

---TCA-----CGAAAAGAA---TC-AAACAGTT-TC--------------------------ACA-CAC--

AAAC--G-CA-CCT------------TCC--T-GTC-------T-G--CC-C-T-GG-C---------A--G

TGACG-AAT-A--A-A-AA--A-TT-TA-TTGCC-AATCTTGT-GGGACGAA----TCT------GGG----

-------------GCTCAAACCTCAG-C----------------CAAAT--CTCAATAGACACA---G----

------------------TGA--C----GAA-TATCTGG----------T--A-AAAATTTC-AGA-CCA--

--AAATACCC-------AAGGA-GT-----AAGGC---GT-----AGTA------AG-TCCTAGA-CCGAGA

GTGAA-------------------------------------------------------------------

------------------------------------------------------------------------

--------------C--AAAACA--------------------------GG-----------------TTGT

C----------------------CGAAA--AAAA--C--GTCATGGC-----TTTTCTTCCC----------

--------------CGTATC--TTCT-----------TTTTGTGA---------------------------

-------------------------TTC--GTTTAT-GGAC--GTG-CCT-CC-TTGCCT--GCATGC--AA

AC-----------AACAT-A----CGAAAGT-GCTTGTG-T-------------GAATTT------------

----TTTTCA------------------------------GCACAAT-A-C-G-GA-CCC-AGA-----AT-

GAAATT--GCATAAA--TTATG------------CCGGCATTT--CACA-A-GTT-TCGG--TAGAT-----

---------GAT-AGCCT-------------------TGG-TTTGCACA----AAATTTATGAAAAACT-

>Pv01Sk01420/1-529 Pv01Sk01420 undefined product 1712238:1712766 reverse

CTCCCGGAATAG-----TTTTTTCCTA--AAATTCCACCC-AAA-AC--T-------CT---CCATTA-AA-

TTTGGG-AC----------AAAACGCG-AC-AA-AT--TTTAGA--TC------------------------

-----------------------------------------AAAC-G--GATGAG---TATTCAC-------

---TCA-----CGAAAAAAA---TA-ATACAGTT-TC--------------------------ACA-CAA--

AAAC--G-AA--CT------------TCT--T-TTC-------A-G--CC-C-T-GA-C---------A--G

TGACG-AAT-A--A-A-AA--T-TT-TA-TTCCC-AATCTCGT-GGGATGAA----TCT------GGG----

-------------GCTCAAATCTCAG-T----------------CAAAT--CTCAATAGACACA---G----

------------------TAA--C----GAA-TGTCTGG----------T--A-AAAATTTC-AAA-CCA--

--AAATACCC-------AAGGA-GT-----AAGGT---GT-----TGTA------AG-TCCTAGA-CCGAGA

GTGAA-------------------------------------------------------------------

------------------------------------------------------------------------

--------------C--AAAACA--------------------------GG-----------------TTGT

C----------------------CGAAA--AAAA--C--GTCCTGCC------TTTCTTCCC----------

--------------CGTATC--TTCT-----------TTTTGTGA---------------------------

-------------------------TTT--GTTTAT-GAAC--GTG-CCT-CC-TTGCCT--GCGTGC--AA

AC-----------AACAT-A----CGAAAGT-GCTTGTG-T-------------GAGTTT------------

---CTTTTCA------------------------------GCGCAAT-A-C-G-GA-CCC-AGA-----AT-

G-----------AAA--TTAGG------------CCGGAATTT--AACA-T-GTT-TCGG--TAGAG-----

---------GAT-AGCCT-------------------TGG-TTTGCACA----GAATTTCTGAAAAATTC

>Pv01Sk01430/1-522 Pv01Sk01430 undefined product 1712770:1713291 reverse

CTCCCGAAATAG-----GTTTTTCCTG--AAATGCCACCC-AAG-AA--T-------CT---CTACTG-AA-

TTTGGG-AT----------AAAACATG-AC-AA-AT--TTCAAG--TC------------------------

-----------------------------------------AAAG-A--GATGAG---TATTCAC-------

---TCA-----GGAATAAAA---TC-AAACAGTT-CC--------------------------ACA-AAC--

AAAC--G-AA-CCT------------TCC--T-TTT-------A-G--CC-C-T-GG-C---------A--G

TGACG-AAT-A--A-A-AA--A-TT-TA-TTGCC-AATCTTGT-GGGACGAA----TCT------GGG----

-------------GCTCAAACTTCAG-C----------------CAAAA--CTCAATAGACACA---G----

------------------TGA--C----GAA-TGTCTGG----------T--A-AAAATTTC-ATA-CCA--

--AAATACCC-------AAGGA-GT-----AAGGC---GT-----AGTA------AG-TCCCGAA-CCGAGA

GTGAA-------------------------------------------------------------------

------------------------------------------------------------------------

--------------C--AAAACC--------------------------GG-----------------TTTT

C----------------------CAAAAACAAAA--C--GT-------------------------------

--------------CGTATC--TTCT-----------TTTTGTGA---------------------------

-------------------------TTC--GTTTAT-GGAC--GTG-CCT-CC-TTGCCT--GGGTGC--AA

AC-----------AACAT-A----CGAAAGT-GCTTGTG-C-------------GAATTT------------

----TTTTCA------------------------------GCGCAAT-A-C-G-GA-CCC-AGA-----AT-

GAAATG--GCAGAAA--TTAGG------------CCGGAATTT--CACA-A-GTT-TCGG--TAGAG-----

---------GGT-AGCCT-------------------TGG-TTTGCACA----AAATTTCTGAAAAA---

>Pv01Sk01440/1-540 Pv01Sk01440 undefined product 1713292:1713831 reverse

CTCCCGGAATAA-----TTTTTTCCTG--AAATTCCACCC-AAG-AA--T-------CT---CCAGTG-AA-

TTTGCG-AC----------AAAACCCG-AC-AA-TT--TTCAGG--TC------------------------

-----------------------------------------AAAC-G--GATGAG---TATTTAC-------

---CCA-----CGAAAAAAA---TC-AAACAGTT-TC--------------------------ACA-CAC--

AAAC--G-AA-CAT------------TCC--T-TTC-------A-G--CA-C-T-GA-C---------A--G

TGACG-AAT-A--A-A-AA--A-TT-TA-TTGTC-AATCTTGT-GGGACGAA----TAT------GGG----

-------------GCTCAAACCTCAG-C----------------CAAAA--CTCAATAGACACA---G----

------------------TGA--G----GAA-TGTCTGG----------T--A-AAAATTTC-AGA-CCA--

--AAATACCC-------AAGGA-GT-----AAGGT---GT-----AGTA------AG-TCCCTGA-CCGAGA

GTGAA-------------------------------------------------------------------

------------------------------------------------------------------------

--------------A--AAAATC--------------------------GG-----------------TTTT

C----------------------CGAAAACAAAA--C--GTCCTGGC-----TTTTCTTCCC----------

--------------CGTATC--TTCG-----------TTTTGTGA---------------------------

-------------------------TTC--GTTTAT-GGAC--GTG-CCT-CC-TTGCCT--GGGTGC--AA

AC-----------AATAT-A----CGAAAGT-GCTTGTG-T-------------GAATTT------------

----TTTTCA------------------------------GCGCTAT-A-C-G-GA-TCT-AGA-----AT-

GATATG--GCAGAAA--TTAGG------------CCGGAATTT--CACA-A-GTT-TCGA--TTGAG-----

---------GAT-AGCCT-------------------TGG-TTTGCACA----AAATTTCTGAAAAACT-

>Pv01Sk01450/1-513 Pv01Sk01450 undefined product 1713831:1714343 reverse

CTCCCATAATAT-----TTTTTCCCTG--AAATTCCACCC-AAG-AA--T-------CT---CCACTG-AA-

TTTGGG-AC----------AAAACGCG-AC-AA-GT--TTCAGG--TT------------------------

-----------------------------------------AAAC-G--GATGAG---TATTCAC-------

---GCA-----CGAAAAAAA---TC-AAACAGTT-TC--------------------------ACA-TAC--

AAAC--G-AA-CCT------------TCC--T-TTC-------A-G--CT-C-T-GA-C---------A--G

TGACG-AAT-A--A-A-AA--A-TT-TA-TTGCC-AATCTTAT-GTGACGAA----TCT------AGA----

-------------G-TCAAACTTCAG-C----------------AAAAA--CTCAATAGACACA---G----

------------------TGA--C----GAA-TGTCTGG----------T--A-AAAATTTC-ATA-CCA--

--AAATACCC-------AAGGA-GT-----AAGGC---GT-----AGTA------AG-TCCCAGA-CCGAGA

GTGAA-------------------------------------------------------------------

------------------------------------------------------------------------

--------------C--AAAACC--------------------------GG-----------------TTTT

C----------------------CGAAAACAAAA--C--GTCCTGGC-----TTTTCTTCCC----------

--------------CGTATC--TTCT-----------TTTTGTGA---------------------------

-------------------------TTC--GTTTAT-GGAC--GTG-CCT-CC-TTGCCT--AGGTGC--AA

--------------------------------GCTTGTA-T-------------GAA-TT------------

---TTTTTCA------------------------------GCGCAAT-A-C-G-GA-CCC-AGA-----AT-

GAAATT--GCTGAAA--TTAGG------------CCGGAATTT--CACA-A-CTT-TTTG--T---------

-------------------------------------TGG-TTTGCACA----AAATTTTTGAAAAATTC

>Pv01Sk01460/1-523 Pv01Sk01460 undefined product 1714343:1714865 reverse

CTCCCAGAATAG-----TTTTTTATTG--AAATTCCACCC-AAG-AA--T-------CT---CCATTA-AA-

TTTGGG-AC----------AAAACGTG-AC-AA-AT--TTTAGG--TC------------------------

-----------------------------------------AAAC-G--GATGAG---TATTCAC-------

---CCA-----CGAAAAAA----TC-AAACAGTT-TC--------------------------CCA-CAC--

AAAT--A-AA-CCT------------TCC--T-TTC-------A-G--CC-C-T-GG-C---------A--G

TAACG-AAT-A--A-A-AA--A-TT-TA-TTGCC-AATCTTGT-GGGACGAA----TCT------GGG----

-------------GCTCAAACCTCAG-C----------------CAAAA--CTCAATAGACAGA---G----

------------------TGA--A----GAA-TGTCTGG----------T--A-AATATTTT-AGA-CCA--

--AAATACCC-------AAGGA-GT-----AAGGC---GT-----AGTA------AG-TCCCAGA-CCGAGA

GTGAA-------------------------------------------------------------------

------------------------------------------------------------------------

--------------C--AAAACC--------------------------GG-----------------TATT

C----------------------CAAAAACAAAA--C--GT-------------------------------

--------------CGTATC--TTCT-----------TTTTTAGA---------------------------

-------------------------TTC--GTTTAT-GGAC--GTG-CCT-CC-TTGCCT--GGGTGC--AA

AC-----------AACAT-A----CGAAAGT-GCTTGT--------------------GT------------

---TTTTTCA------------------------------GTACAAT-A-C-G-GA-CCC-AGA-----AT-

AAAATT--GCAGAAT--TTAGG------------CCGGAATTT--TACA-A-GTT-TCGG--TAGAGGATA-

---------GAT-AGCCT-------------------TGG-TTTGCACA----AAATTTCTAAAAAATTC

>Pv01Sk01470/1-526 Pv01Sk01470 undefined product 1714865:1715390 reverse

CTCCCGGAATAC-----TTGTTTCCTG--AAATTCCACCC-AAG-AA--T-------CT---CCACTG-AA-

TTTGGG-AC----------AAAACGCG-AC-AA-AT--TTCAGC--TC------------------------

-----------------------------------------AAAC-G--GATGAG---TATTCAC-------

---CCA-----CGAAAAAAA---TC-AAATTGTT-TC--------------------------ACA-CAC--

AAAT--A-AA-CCT------------TCC--T-TTC-------A-G--CC-T-T-GA-C---------A--G

TGACG-AAT-A--A-A-AA--A-TC-TA-CTGCC-AATCTTGT-GGGACGAA----TCC------GAG----

-------------GCTCAAACCTCAG-C----------------CAAAA--CTTAATAGACACA---G----

------------------TGA--C----GAA-TGTCTCA----------T--A-AAATTTTC-AGA-CCA--

--AAATTCCC-------AAGAA-GT-----AAGGC---GT-----AGTA------AG-TCCCAGA-CCGAGA

TTGAA-------------------------------------------------------------------

------------------------------------------------------------------------

--------------C--AAAACC--------------------------GG-----------------TTTT

C----------------------CGAAAAAAAAAA-C--G---------------------C----------

--------------TGTATC--TTCT-----------TTTTGTGA---------------------------

-------------------------TTC--GTTTAT-GGAC--GTG-CCT-CC-TTGCCT--GGGTGC--AA

AC-----------AACAT-A----CGAAAGT-ACTTGTG-T-------------GAATTT------------

----TTTTCA------------------------------GCGCAAT-A-C-G-GA-CCC-ACA-----AT-

GAAATT--CCAGAAA--TTAGG------------CCGGAATTT--CACA-A-GTT-TCGG--TAGAG-----

---------GAT-ATCCT-------------------TGG-TTTGCACA----AAATTTCTGAAAAATTC

>Pv01Sk01480/1-540 Pv01Sk01480 undefined product 1715390:1715929 reverse

CTCCTGGAATAG-----TTTTTTCCTG--AAATGCCACCC-AAG-AA--T-------CT---CCACTG-AA-

TTTGGG-AC----------AAAACATG-AC-AA-AT--ATTAGG--TC------------------------

-----------------------------------------AAAC-A--GATGAG---TATTCAC-------

---CAA-----GTACAAAAA---TC-AAACAGTT-TC--------------------------ACA-CAC--

AAAC--G-AA-TCT------------TCC--T-TTT-------A-A--CT-C-T-AG-C---------A--G

TGACA-AAT-A--A-A-AA--C-TT-TA-TTGTC-AATCTTGT-GGGACGAA----TCT------AGG----

-------------GATCAAACCTCAG-C----------------CAAAA--CTAAATAGACAAA---A----

------------------CGA--C----GAA-TGTCTGG----------T--A-AAAATTTC-AGA-CCA--

--AAATACCC-------AAGGA-AT-----AAGGC---GT-----AGTA------AG-TCCCAGA-CCTAGA

GTGAA-------------------------------------------------------------------

------------------------------------------------------------------------

--------------C--AAAACC--------------------------GG-----------------TTTT

C----------------------CGAAAACAAAA--C--GTCCTTGC-----TTTTCTTTCT----------

--------------CGTATC--TTCT-----------TTTTTGTG---------------------------

-------------------------ATT--CGTTAT-GCAC--GTG-CCT-CC-TTGCCT--GGGTGC--AA

AC-----------AACAT-A----CGAAAGT-GTTTGTG-T-------------GAATTT------------

-----TTTCA------------------------------GCGCAAT-A-C-G-GA-CCC-AGA-----AT-

GAAATT--GTAGAAA--TTAGG------------ACGGAATTT--CACA-A-GTA-TCAG--TAGAG-----

---------GAT-AGCCT-------------------TGG-TTTGCACA----AAATTTCTGAAAAATTC

>Pv01Sk01490/1-540 Pv01Sk01490 undefined product 1715929:1716468 reverse

-TTCCGAAATAT-----TTTTTTCCTG--AAATTCCACCC-AAG-AA--T-------CT---CCACTG-AA-

TTTCGA-AC----------AAAATGCG-AC-AA-AT--TTTAGG--TT------------------------

-----------------------------------------TAAG-G--GATGAG---TATTCAC-------

---CTA-----CCAAAAAAA---TC-AAACAGTT-TC--------------------------ACA-CAC--

AGAC--G-AA-GCT------------TCC--T-TTC-------A-G--CC-C-T-GG-C---------A--G

TGACG-AAT-A--A-A-AA--A-TG-TA-TTGCC-AATCTTGT-AGGACGAA----TCT------GGG----

-------------ATTCAAACCTCAA-C----------------CAAAA--CTCAATAGGCACA---G----

------------------TGA--C----GAA-TGTCTGG----------T--A-AAAATTTC-AGA-CTA--

--AAATACCC-------AAGGA-GT-----AAGGC---GC-----AGTA------TG-TCCTAGA-TCGAAA

GTGAA-------------------------------------------------------------------

------------------------------------------------------------------------

--------------C--AAAACC--------------------------AA-----------------TTTT

C----------------------CGAAAACAAAA--C--GTCTAGGA-----TTTTCTTCCC----------

--------------CGTATC--TTCT-----------TTTTGTCA---------------------------

-------------------------TTC--GTTTAT-GGAC--GTG-CTT-CC-TAGCCT--GGGTGC--AA

AC-----------AGCAT-A----CGAAAGT-GCTTGTG-T-------------GAATTT------------

----TTTTCA------------------------------GCACAAT-A-C-G-GA-CCC-TCA-----AT-

GAAATT--CCAGAAA--TTAGG------------CCGGAATTT--CACA-A-GTT-TCGG--TTGAG-----

---------GAT-ATCCT-------------------TGG-TTTGCATA----AAATTTCTGAAAAATTC

>Pv01Sk01500/1-540 Pv01Sk01500 undefined product 1716469:1717008 reverse

CTCCCGGAATAG-----TTTTTTCCTG--AAATTCCACCA-AAG-AA--T-------TT---CCACTG-AA-

TTTTGG-AC----------AAAACGTG-AC-AA-AT--TTCAGG--TC------------------------

-----------------------------------------AAAG-G--GATGAG---TATTCAC-------

---CCA-----CGAAAAAAA---TC-AAACAGTT-TC--------------------------ACA-CAC--

AAAC--G-AA-CCT------------TCC--T-TTC-------A-G--CT-C-T-GG-C---------A--G

TGACG-AAT-A--A-A-AA--A-TT-TA-TTGCC-AATCTTAT-GGGACGAA----TCT------GG-----

-------------GGTTAAACCTCAA-C-----------------AAAA-ACTCAATAGACACA---G----

------------------TGA--C----GAA-AGTCTGG----------T--A-AAAATTTC-AGA-CCA--

--AAATACCT-------AAGGA-GT-----AAGGC---GA-----AGTA------AG-TCCCAGA-CCGAGA

GTGAA-------------------------------------------------------------------

------------------------------------------------------------------------

--------------C--AACACC--------------------------GG-----------------TTTT

C----------------------CGAAAACAAAA--C--GTCCTAAC-----TTTTCTTCCC----------

--------------TGTATC--TTCT-----------TTTTTTGA---------------------------

-------------------------TTC--GTTTAT-GGAC--GTG-CCT-CC-TTGCCT--AGGTGC--AA

AC-----------AACAT-A----CGAAAAT-GCTTGTG-T-------------GAA-TT------------

---TTTTTCA------------------------------GCACAAT-A-C-G-GA-CCA-AGA-----AT-

GAAATT--GCAGAAA--TTAGG------------ACGGAATTT--CACA-A-GTT-TCGG--TAGAG-----

---------GAT-AGCGT-------------------TGG-TTTTCACA----AAATTTCTCAAAAATTC

>Pv01Sk01510/1-541 Pv01Sk01510 undefined product 1717008:1717548 reverse

CTCCCAGAATAG-----TTTATTCCTG--AAATTCCACCC-AAG-AA--T-------CT---CCATTA-AA-

TTTGGG-AC----------AAAACGCG-AC-AA-AT--TTTAGG--TC------------------------

-----------------------------------------AAAC-G--GATGAG---TATTCAC-------

---CCA-----CGAAAAAAA---TC-AATCAGTT-TC--------------------------ACA-CAC--

AAAC--G-GA-CCT------------TCC--T-TTC-------A-G--CT-C-T-AG-C---------A--G

TGACG-AAG-A--A-A-AA--A-TT-TA-TTGCC-AATCCTGT-GGGATGAA----TCT------GAC----

-------------GCTCAAACCTCAG-C----------------CAAAA--CTCAATAGACACA---G----

------------------TGA--C----GAA-TGTCTGG----------T--A-AAAATTTC-AAA-CCA--

--AAATACCA-------AAGGA-GT-----AAGGC---GT-----AGTA------AG-TCCCAGT-CCGAGA

GTGAA-------------------------------------------------------------------

------------------------------------------------------------------------

--------------C--AAAATC--------------------------GG-----------------TTTT

C----------------------CGAAAACAAAA--C--GTTCTGGC-----TTTTCTTCCC----------

--------------CGTATC--TTCT-----------TTTTGTGA---------------------------

-------------------------TTC--GTTTAT-GGAC--GTG-CTT-CC-TTGCCT--AGGTGC--AA

AC-----------AACAT-A----CGAAAGT-GCTTGTG-T-------------GAATTT------------

----TTTTCA------------------------------GCGCAAT-A-C-G-GA-CCC-AGA-----AT-

TAAATT--GCAGAAA--TTAGG------------CCGGAATTT--CACA-A-GTT-TCAG--TAGAG-----

---------GAT-AACTT-------------------TGG-TTTGTACA----AAATTTCTGAAAAATTC

>Pv01Sk01520/1-540 Pv01Sk01520 undefined product 1717548:1718087 reverse

-TCCTTGAATAG-----TTTTTTCCTG--AAATTCCACCC-AAG-AA--T-------CT---CTACTG-AA-

TTTGGT-AC----------AAAACGCG-AC-AA-AT--TTTAGG--TC------------------------

-----------------------------------------AAAC-G--GATGAG---TATTCAC-------

---CTG-----CGAAAAAAA---TC-AAAAAGTT-TC--------------------------ACA-CAC--

AGAC--G-AA-CCT------------TCC--T-TTC-------A-G--CC-C-T-GA-C---------A--G

TGACG-AAT-A--A-A-AA--A-TT-TA-TTGCC-AATCTTGT-GGGACGAA----TCT------GAG----

-------------GCTCAAACCTAAG-C----------------CAAAA--CTCAATAGACACA---G----

------------------TGT--C----CAA-TGTCTGG----------T--A-AAAATCTC-AGA-CCA--

--AAATACCC-------AAGGA-GT-----AAGGC---GT-----AGTA------AG-TCTCAGA-CCAAGA

GTGCA-------------------------------------------------------------------

------------------------------------------------------------------------

--------------C--AAAATC--------------------------GG-----------------TTTT

C----------------------CGAAAACAAAA--C--GTCCTGAC-----TTTTCTTCCC----------

--------------CGTATC--TTCT-----------TTTTAAGA---------------------------

-------------------------TTC--GTTTAT-GGAC--GTG-CCT-CC-TTGCCT--AGGTGC--AA

AA-----------AACAT-A----CGAAAGT-GCTTGTG-T-------------GAATTT------------

----TTTTCA------------------------------GCGCAAT-A-C-G-GA-CCA-AGA-----AT-

GAAATT--GCAGAAA--TTAGG------------ATGGAATTT--CACA-A-GTT-TCGG--TAGAT-----

---------GAT-AGCGT-------------------TGG-TTTGCACA----AAATTTCTGAAAAATTC

>Pv01Sk01530/1-539 Pv01Sk01530 undefined product 1718088:1718626 reverse

-TTCCGAAATAG-----TTTTTTCTTG--AAATTCCACCC-AAG-AA--T-------CT---CCACTA-AA-

TTTGGG-AC----------AAAACGCG-AC-AA-AT--TTCAGG--TT------------------------

-----------------------------------------AAAC-A--GATGAG---TATTCAC-------

---CCA-----CGAAAAAAA---TC-AAACAGTT-TC--------------------------ACA-CAC--

AAAC--G-AA-CAT------------TTC--T-TTC-------A-G--CC-A-T-GA-C---------A--G

TGACG-AAT-A--A-A-AA--A-TT-TA-TTACC-ATTCTTGT-GAGATGAA----TCT------GGA----

-------------GCTCAAACCTCAG-C----------------CAAAA--CTCAATAGACAAA---G----

------------------TGA--C----GAA-TGTCTGG----------T--A-AAAATTTA-AGA-CCA--

--AAATACTC-------AAGGA-GT-----AAGGT---GT-----AGTA------AG-TCCTAGA-CCGAGA

ATGAA-------------------------------------------------------------------

------------------------------------------------------------------------

--------------C--AAAACC--------------------------AG-----------------TTTT

T----------------------CGAAAACAAAA--C--GTCCTAGC-----TTTTCTTTCC----------

--------------CGTATA--TTTT-----------TTTTTT-A---------------------------

-------------------------TTC--GTTTAT-GGAC--GTG-CCT-CC-TTTCCT--GGGTAC--AA

AC-----------AACAT-A----CAAACGT-GCTTGTA-T-------------GAATTT------------

----TTTTCA------------------------------GCGCAAT-A-C-G-GA-CAC-ATA-----AT-

GAAATT--GCAGAAA--TTAGG------------CCGAAATTT--TACA-A-GTT-TCTT--AAAAG-----

---------GAT-AGACT-------------------TGG-TTTGCACA----AAATTTTTAAAAAATTC

>Pv01Sk01540/1-544 Pv01Sk01540 undefined product 1863785:1864328 reverse

CTCCCGAAATAA-----TTTTTTCTTG--AAATTCCACGT-AAG-AA--T-------CT---CGATTG-AA-

TTTGGA-AC----------AAAACGCG-AT-AA-AT--TTCAGG--TC------------------------

-----------------------------------------AAAC-G--GATGAG---TATTCAC-------

---CTA-----AGAAAAAAA---TC-AAACAGTT-TC--------------------------ACA-CAC--

AAAC--A-AA-CCT------------TCC--T-TTC-------A-G--CC-T-T-GA-C---------A--A

TTTTG-AAT-A--A-A-AA--A-TT-TA-TTGTG-AATCTTGT-GAGACGAA----TTT------TGG----

-------------GCTCAAACCCAAG-C----------------C-AAA-ACTCAACACACACA---A----

------------------TGA--C----GAA-TGTCTGG----------T--A-AAAATTTC-AGA-CCA--

--AAAAACTC-------AAGGA-AT-----AAGGC---GT-----AGTA------AA-TCACAGA-CCAAGA

GTGAA-------------------------------------------------------------------

------------------------------------------------------------------------

--------------C--AAAACT--------------------------AG-----------------TGTT

C----------------------GGAAAACAAAA--C--ATTCTGTC-----TTTTCTTCCC----------

--------------TGTATC--TCAT-----------TTTTGTGA---------------------------

-------------------------TTT--GTTTAT-GGAC--GTG-TCT-CC-TTACCT--GGGTGC--AA

AC-----------AACAT-A----TGAAAGT-ACTTGTG-T-------------GAA---TTTTT----TCG

GAATTTTTCA------------------------------ACGCAAT-A-C-G-GA-CCC-AGA-----AT-

AAAATT--GTAGAAA--GTAGG------------GCGAATATT--CACA-A-GTT-TTGG--TAGAG-----

----------------TT-------------------TCG-TTTGCACA----AAATTTCTGAAAAATTC

>Pv01Sk01550/1-541 Pv01Sk01550 undefined product 1864328:1864868 reverse

CTCCTGAAATAA-----TTTTTT-TGG--AAATTCGACGT-AAG-AA--T-------CT---TGATTG-AA-

TTTTGA-A-----------------CG-AC-AA-AT--TTCAGG--TC------------------------

-----------------------------------------AAAC-A--GATGAG---TACTCAC-------

---CTA-----GAAAAATA----TC-AAACAGTT-TC--------------------------ACA-CAC--

ACAC--G-AA-CCT------------TCC--T-TTG-------A-G--CC-C-T-AG-C---------A--A

TGATG-AAT-A--A-A-AA--T-TT-TA-TTGCA-AATCTTGT-GGGACGAA----TTT------TGG----

-------------GCTCAAACCCAAG-C----------------C-AAA-ACTCAACACACACA---A----

------------------TGA--C----GAA-TGTCTGG----------T--A-AAAATTTC-AGA-CCA--

--AAAGACCC-------AA-GA-AT-----AAGGC---GT-----AGTA------AG-TGCCAGA-CCGAGA

TTGAA-------------------------------------------------------------------

------------------------------------------------------------------------

--------------C--AAAACT--------------------------GT-----------------TTTT

C----------------------GGAAAACAAAA--T--GTTCTTTC-----TTTTCTTCCC----------

--------------AATATC--TCAT-----------TTTTGTGA---------------------------

-------------------------TTT--TTTTAT-GGAC--ATG-CCT-CG-TTACCT--AGGTGC--AA

AA-----------AACAT-A----TGAAAGT-ACTTCTG-T-------------GAA---TTTTT----TTG

GAATTTTTCA------------------------------ACACGAT-A-T-G-GACCCC-AGA-----AT-

AAAATT--GTAGAAA--GTATG------------GTAAAATTT--CACA-A-GGT-TTGG--TAGAA-----

---------GAT-AGACT-------------------TGG-TTTGCACA----AAATTTCATAAAAATTC

>Pv01Sk01580/1-539 Pv01Sk01580 undefined product 1870106:1870644 forward

CTCCCAAAATAG-----ATTTTGCCTG--AAATTCCACGT-AAA-AA--T-------CT---CCACTG-AA-

TTTAGG-AC----------AAAACGCA-AC-AA-AT--TTCAGG--TC------------------------

-----------------------------------------AAAC-G--GATGAG---TATTTAC-------

---CCA-----CAAAAAAAA---TC-AAACAGTT-TC--------------------------CCA-CAC--

AAAC--G-AA-CCC------------TCC--T-TTC-------A-G--CC-C-T-GG-C---------A--G

TGATG-AAT-A--A-A-AA--A-TT-TA-TTGCC-AATCTTGT-GAGACGAA----TAT------TGG----

-------------TCTCAAATCTCAA-C----------------C-AAA-ACTTATCAGACACC---G----

------------------TGA--C----GAA-CGTCTGG----------T-AA--AAATTTC-AGA-CCA--

--AAATATCC-------AAGGA-GT-----AAGGC---GT-----AGTA------AG-TCCCAGA-CCGAGA

GCGAA-------------------------------------------------------------------

------------------------------------------------------------------------

--------------C--AAAACT--------------------------GG-----------------TTTT

C----------------------CGAAAACAAAC--C--CTCATGGC-----TTTTCTTCCC----------

--------------CGTATC--TTCT-----------TTTTGTGA---------------------------

-------------------------TTT--TTTTAT-GGAC--GTG-CCT-CC-TTACCT--GGGTGC--AA

AG-----------AACAT-A----GGAAAGT-ACTCGTA-T-------------GAA---------------

--TTTTTTCA------------------------------GCGGAAT-A-C-G-G--CCC-AAA-----TT-

AAAATT--GCAGAAA--GTAGG------------GCCAAATTT--CACA-A-GTT-TTGG--TAGAT-----

---------GAT-GGCCT-------------------TGG-TTCGCACA----AAATTTCTGAAAAATTC

>Pv01Sk01590/1-538 Pv01Sk01590 undefined product 1870645:1871182 forward

-TACCGAAATAG-----TTTTTTCCTG--AAATTCCACGT-AAG-AA--T-------CT---CCACTG-AA-

TTTGGG-AC----------AAAACGCG-AC-AA-AT--TTCAGG--TC------------------------

-----------------------------------------AAAC-G--GATGAG---TATTCAA-------

---CAA-----CGAAAAAAA---TC-AAACAGTT-TC--------------------------GCT-CAC--

AAAC--G-AA-CCC------------TCC--T-TTC-------A-G--CC-C-T-GA-C---------A--G

TGACG-AAT-A--A-A-AA--A-TT-TA-TTGCC-AATCTTGT-GAGACGAA----TCT------GGT----

-------------TCTCAAATCTCAG-T----------------C-AAA-ACTTATCAGACACA---A----

------------------TGA--C----GAA-CGTCTGG----------A-AA--AAATTTC-AGA-CCA--

--AATTATCC-------AAGGA-GT-----AAGGC---GT-----AGTA------AG-TCCCAGA-CCGAGA

GCGAG-------------------------------------------------------------------

------------------------------------------------------------------------

--------------A--AAAACT--------------------------GG-----------------TTTT

C----------------------CGAAAACAAAA--C--GTCATGGC-----TTTTCTTCCC----------

--------------CGTATC--TTCT-----------TTTTGTGA---------------------------

-------------------------TTT--TTTTAT-GGAC--GTG-ACT-CC-TTACCT--GGGTGC--AA

AC-----------ACCAT-A----GGAAAGT-AGTCGTG-T-------------GAA---------------

--TTTTTTCA------------------------------GCACAAT-A-C-G-G--CCC-AGA-----AT-

AAAATT--GCAGAAA--GTAGG------------GCCAAATTT--CACA-A-GTT-TTGG--TAGAG-----

---------GTT-AGCCT-------------------TGG-TTTGCACA----AAATTTCTGAAAAATTC

>Pv01Sk01600/1-530 Pv01Sk01600 undefined product 1872259:1872788 forward

CTCCCAAAATAG-----ATTTTGCCTG--AAATTCCACGT-AAA-AA--T-------CT---CCACTG-AA-

TTTAGG-AC----------AAAACGCA-AC-AA-AT--TTCAGG--TC------------------------

-----------------------------------------AAAC-G--GATGAG---TATTTAC-------

---CCA-----CAAAAAAAA---TC-AAACAGTT-TC--------------------------CCA-CAC--

AAAC--G-AA-CCC------------TCC--T-TTC-------A-G--CC-C-T-GG-C---------A--G

TGATG-AAT-A--A-A-AA--A-TT-TA-TTGCC-AATATTGT-GGGACGAA----TCT------GGG----

-------------GCTCAAATGTCAT-C----------------C-AAA-ACTCTACAGACAGA---G----

------------------TGA--C----GAA-CGTCTGG----------T-AA--AAATTTC-AGA-CCA--

--AATTACCC-------AAGGA-GT-----AAGGC---GT-----AGTA------AG-TCCCAGA-CCGAGA

GCGAA-------------------------------------------------------------------

------------------------------------------------------------------------

--------------C--AAAACT--------------------------GG-----------------TTTT

C----------------------CGAAAACAAAA--C--GTCCTAGC-----TTTTCTTCCC----------

--------------CGTCTC--TTCT-----------TTCCGTGA---------------------------

-------------------------TTT--GTTTAT-GGAC--GTG-CGA-CC-TTACCT--GGGTGC--AA

AC-----------ACCAT-A----GGAAAGT-AGTCGTG-T-------------GAA---------------

--TTTTTTCA------------------------------GCGCAAT-A-C-G-GA-CCC-AGA-----AT-

AAAATT--GCAGAAA--GTGTG------------GCAAAATTT--CACA-A-GTT-CTGG--TA--------

----------------TT-------------------TGG-TTTGCACA----AAATTTAAGAAAAATT-

>Pv01Sk01610/1-541 Pv01Sk01610 undefined product 1872790:1873330 forward

-TCCCGAAATAG-----TTTTGTCCTG--AAATTCCACGT-AAG-AA--T-------CT---CCACTG-AA-

TTTGGG-AC----------AAAACGCG-AC-AA-AT--TTCAGG--TC------------------------

-----------------------------------------AAAC-G--GATGAG---TATTCAC-------

---CCA-----CGAAAAAAAA--TC-AAACAGTT-TC--------------------------GCT-CAC--

AAAC--G-AA-TCC------------TCC--T-TTT-------A-G--CC-C-T-GG-A---------A--G

TGATG-AAT-A--A-A-AA--A-TT-TA-TTGCC-AATATTGT-GGTACGAA----TCT------GGG----

-------------GTTCAAATCTCAT-C----------------C-AAA-ACTCAACAGACACA---G----

------------------TGA--C----GAA-CGTCTGG----------T-AA--AAATTTC-ATA-CCA--

--AAATACCC-------AAGGA-GT-----AAGGC---GT-----AGTA------AG-TCCCAGA-CCGAGA

GCGAA-------------------------------------------------------------------

------------------------------------------------------------------------

--------------C--AAAACT--------------------------GG-----------------TTTT

C----------------------GGAAAACAAAA--C--ATCCTGGC-----TTTTCTTCCC----------

--------------CGTATC--TTCT-----------TTCTGTGA---------------------------

-------------------------TTT--GTTTAT-GGAC--GTG-CCT-CC-ATACTT--GGGTGC--AA

AC-----------AACAT-A----GGAAAGT-ACTCGTG-T-------------GAA---------------

-TTTTTTTCA------------------------------GCGCAAT-A-C-G-GC-CTC-AGA-----AT-

AAAATT--GCAGAAA--GTAGG------------GCCAAATTT--CACA-A-GTT-TTGG--TAGAG-----

---------GAT-AGCCT-------------------TGG-TTTGCACA----AAATTTCTGAAAAATTC

>Pv01Sk01620/1-538 Pv01Sk01620 undefined product 1873330:1873867 forward

CTCCCGAAATAG-----TTTTTTCCTA--AAATTCCACGT-AAG-AA--T-------CT---TCA--G-AG-

TTTTGG-AC----------AAAACGCG-AC-AA-AT--TTCAAG--TC------------------------

-----------------------------------------AAAC-G--GATGAG---TATTCAC-------

---CCA-----CGAATAAAA---TC-AACCAATT-TC--------------------------ACA-CAC--

AAAC--G-AA-CCC------------TTT--T-TTC-------A-G--CC-C-T-AG-C---------A--G

TGATG-AGT-A--A-G-AA--A-TT-TA-TTGCC-AATCTTGT-GAGACGAA----TCT------GGG----

-------------GCTCAAATCTCAG-C----------------C-AAA-ACTCAATAGACACA---G----

------------------TGA--C----GAA-CGTCTGG----------T-AA--AAATTTC-AGA-CTG--

--AAATACCT-------AAGGA-GT-----AAGGC---GT-----AGTA------AG-TCCCACA-CCGAGA

GCGAA-------------------------------------------------------------------

------------------------------------------------------------------------

--------------C--AAAACT--------------------------GG-----------------TTTT

C----------------------CGAAAACAAAA--C--ATCCTGGC-----TTTTCTTCAA----------

--------------CATTTC--TTCT-----------TTCCGTGA---------------------------

-------------------------TTT--GTTTAT-GGAC--GTG-CCT-CC-TTACCT--AGGTGC--AA

AA-----------AACAT-A----GGAAAGT-ACTCGTG-T-------------GAA---------------

--TTTTTTCA------------------------------GCGCAAT-A-C-A-GG-CCC-ATA-----AT-

AAAAAT--GTAGAAA--GTAGA------------GCCAAATTT--CACA-A-GTT-TTGG--TAGAG-----

---------GAT-AGCCT-------------------TGG-TTTGCACA----AAATTTCTGAAAAATTC

>Pv01Sk01630/1-539 Pv01Sk01630 undefined product 1873867:1874405 forward

CTCCCGAAATAG-----TTTTTTCCTG--AAATTCCACAT-AAG-AA--T-------CT---TCACTG-AA-

TTTTGG-AC----------AAAATACG-AC-AA-AT--TTCAGG--TC------------------------

-----------------------------------------AAAT-G--GATGAC---TATTCAC-------

---CCA-----CGAAAAAAA---TC-AAACAGTT-TC--------------------------GCA-TAC--

AAAC--G-AA-CCC------------TTT--T-TTC-------A-G--CC-C-T-AG-C---------A--G

TGATG-AAT-A--A-A-AA--A-TT-TA-TTGCC-CATCTTGT-GCGATGAA----TCT------GGG----

-------------GCTCAAATCTTAG-C----------------C-AAA-ACTCAACAGACAAA---G----

------------------TGA--C----GAA-CGTTTGG----------T-AA--AAATTTC-AGA-CCA--

--AAATATCC-------AAGGA-GT-----AAGGT---GT-----AGTA------AG-TCCCACA-CCGAGA

GCGAA-------------------------------------------------------------------

------------------------------------------------------------------------

--------------C--AAAACT--------------------------GG-----------------TTTT

C----------------------TGAAAACAAAA--C--GTCCTGGC-----TTTTCTTCCC----------

--------------CGTATC--TTCT-----------TTCCGTGA---------------------------

-------------------------TTT--GTTTAT-GGAC--TTG-CCT-CC-TTACCT--GGGTGC--AA

AC-----------AACAT-A----GGAAAGT-ACTCGTG-T-------------GAA---------------

--TTTTTTCA------------------------------GCGCAAT-A-C-G-GG-CCC-AGA-----AT-

AAAATT--GCAAAAA--GTAGG------------GCCAAATTT--CACA-A-GTT-TTTG--TAAAG-----

---------GAT-AGCCT-------------------TGG-TTTGCACA----AAATTTCTGAAAAATT-

>Pv01Sk01640/1-539 Pv01Sk01640 undefined product 1874407:1874945 forward

-TCCCTAAATAG-----TTTTTTCCTG--AAATTCCACGT-AAG-AA--T-------CT---TCACTG-AA-

TTTTGG-AC----------AAAACGCG-AC-AA-AT--TTCAGG--TC------------------------

-----------------------------------------AAAT-G--GATGAA---TATTCAC-------

---CCA-----CGAAAAAAA---TC-AAACAGTT-TC--------------------------GCA-TAC--

AAAC--G-AA-CCC------------TTT--T-TTC-------A-G--CT-C-T-GG-C---------A--G

TGATG-AAT-A--A-A-AA--A-TT-TA-TTGCC-CATCTTGT-GCGATGAA----TCT------GGG----

-------------GCTCAAATCTTAG-C----------------C-AAA-ACTCAATAGACAAA---G----

------------------TGA--C----GAA-CGTTTGG----------T-AA--AAATTTC-AGA-CCA--

--AAATATCA-------AAGGA-GT-----AAGGT---GT-----AGGA------TG-TCCCACA-CCGAGA

GCGAA-------------------------------------------------------------------

------------------------------------------------------------------------

--------------C--AAAACT--------------------------GG-----------------TTTT

C----------------------CGAAAACAAAA--C--GTCCTGGC-----TTTTCTTCCC----------

--------------CGTATC--TTCT-----------TTCCGTGA---------------------------

-------------------------TTT--GTTTAT-GGAC--GTG-CCT-CC-TTACCT--GGGTGC--AA

AC-----------AACAT-A----GGAAAGT-ACTCGTG-T-------------GAA---------------

--TTTTTTCA------------------------------GCGCAAT-A-C-A-GG-CCC-ATA-----AT-

AAAAAT--ATAGAAA--GTATA------------GCCAAATTT--CACA-A-GTT-TTGG--TAGAG-----

---------GAT-AGCCT-------------------TGG-TTTGCACA----AAATTTCTGAAAAATTC

>Pv01Sk01650/1-540 Pv01Sk01650 undefined product 1874945:1875484 forward

CTCCCGAAATAG-----TTTTTTCCTG--AAATTCCACAT-AAG-AA--T-------CT---TCATTG-AA-

TTTTGG-AC----------AAAACGCG-AC-AA-AT--TTCAGG--TC------------------------

-----------------------------------------AAAC-G--GATAAG---TATTCAC-------

---CCA-----CGAAAAAAA---TC-AAACAGTT-TC--------------------------GCA-TAC--

AAAC--G-AA-CCC------------TTT--A-TTC-------A-G--CC-C-T-GG-C---------A--G

TGATG-AAT-A--A-A-AA--A-TT-TA-TTACC-CATCTTGT-GGGATGAA----TCT------GGG----

-------------TCTCAAATCTCAG-C----------------C-AAA-ACTCAACAGACACA---G----

------------------TGA--C----GAA-CGTTTGG----------T-AA--AAATTTC-AGA-CCA--

--AAATATCC-------AAGGA-GT-----AAGGT---GT-----AGTA------AG-TCCCACA-CCGAGA

GCGAA-------------------------------------------------------------------

------------------------------------------------------------------------

--------------C--AAAACT--------------------------AG-----------------TTTT

C----------------------CGAAAACAACA--C--GTCCTGGC-----TTTTCTTCCC----------

--------------CGTATC--TTCT-----------TTCCATGA---------------------------

-------------------------TTT--GTTTAT-GGAC--GTG-CCT-CC-TTACCT--GGGTGC--AA

AC-----------AACAT-A----GGAAGGT-ACTCGTG-T-------------GAA---------------

--TTTTTTCA------------------------------GCGCAAT-A-C-G-GG-CCC-AGA-----AT-

AAAATT--GTAGAAA--GTAGG------------GCCAAATTT--CACA-A-GTT-TTTG--TAGAG-----

---------GAT-AGCCT-------------------TGG-TTTGCACA----AAATTTCTGAAAAATTC

>Pv01Sk01660/1-551 Pv01Sk01660 undefined product 1879295:1879845 reverse

-TCCTGAAATAA--TATTTTTTTTTGG--AAATTCGATGC-AAG-AA--T-------CA---TGATTG-AA-

TTTTGA-AC----------AAAATGCGAAA-AA-AT--TTCAGG--TC------------------------

-----------------------------------------AAAC-G--GATGAC---TATTCAC-------

---TTA-----GAAAAAAA----TC-AAACAGTT-TC--------------------------ACA-AAC--

AGAC--G-AA-CCT------------TGC--T-TTG-------A-G--CC-T-T-GG-G---------A--G

TGATG-AAT-A--A-A-AA--A-TT-TA-TTGCG-AATCTTGT-GGGACAAA----TTT------TGG----

-------------GCTCAAACCCAA--C----------------C-AAA-ACTCAATTCACACA---A----

------------------TGA--T----GAA-TGTATGG----------C--A-AATATTTC-AAA-CCA--

--AAATAACC-------TAGTA-AT-----AAGGT---GT-----AGTA------AG-TCATAGA-CCGAGA

GTTAA-------------------------------------------------------------------

------------------------------------------------------------------------

--------------G--AAAACA--------------------------GA-----------------ATTT

C----------------------GGAAAAACAAA--C--GTCCTGAC-----TTTTCCACCC----------

--------------TATATC--TACT-----------TTTTGTGA---------------------------

-------------------------TTT--GTTTAT-GGAC--GTG-CCT-CA-TTACCT--GGGTAC--AA

AC-----------AATAT-A----TGAAAGT-ACTTGTC-T-------------GAG---TTTTT----TTG

GAATTTTTCA------------------------------ACGTAAT-A-C-G-AA-CTC-TGA-----AT-

AAAATT--GCAGAAA--GTATG------------GCGAAATTT--CACA-A-GTT-TTGG--TAGAG-----

---------GAT-AGTCT-------------------TGG-TTTGTACA----AAATTTCTAAAAAATTC

>Pv01Sk01670/1-547 Pv01Sk01670 undefined product 1879847:1880393 reverse

--TCAAAAATAA-----TTTTTTCTTG--AAATTCCATGT-AAG-AA--T-------CT---CCATTG-AA-

TTTGGA-AC----------AAAACGCT-AC-AA-AT--TTCAGG--TC------------------------

-----------------------------------------AAAC-G--GACGAG---TATTAAC-------

---CTA-----GGAAAAAAA---TC-AAACAATT-TC--------------------------ACG-CAC--

AAAC--G-AA-CCT------------TCC--T-TTC-------A-G--CC-C-T-GA-C---------A--G

TGATG-AAT-A--A-A-AA--A-TT-TA-TTGAG-AATCTTGT-GGGACGAA----TTT------TTG----

-------------GCTCAAACCCAAG-C----------------C-AAA-ACTCAATTCACACA---A----

------------------TGA--T----GAA-TGTCTGG----------T--A-AAAATTTC-AAA-CCA--

--ATATACTA-------AAGTA-AT-----ATGGT---GT-----AGTA------AG-TCACAAA-CCGAGA

GTTAA-------------------------------------------------------------------

------------------------------------------------------------------------

--------------C--AAAACT--------------------------GG-----------------TTTT

C----------------------GGAAAACAAAG--C--GTCCTGGC-----TTTTCTTCCC----------

--------------TATATC--TCCT-----------TTTTGTGA---------------------------

-------------------------TTT--GTTTAT-GGAC--GTG-CTT-CG-TTACAT--GGGTGC--AA

AA-----------AACAT-A----TGAAAGT-ACTTTTA-T-------------TAA---TTTTT----TCA

GAATTATTCA------------------------------ACGCAAT-A-C-G-GC-CCC-AAA-----AT-

AAAATT--GCGGAAA--GTATG------------GCAAAATTT--CACA-A-GTT-TTGG--TAGAG-----

---------GAT-AGTCT-------------------TGG-TTTGCACA----AAATTTATGAAAAAGT-

>Pv01Sk01680/1-549 Pv01Sk01680 undefined product 1880395:1880943 reverse

--TCAAAAATAA-----TTTTTTCTTG--AAATTCCACAT-AAG-AA--A-------AT---CTATTG-AA-

TTTGGA-AC----------AAAACGCG-AC-AA-AT--TTCAGG--TC------------------------

-----------------------------------------AAAC-G--AATGAG---TATTCAC-------

---CTA-----GGAAAAAAA---TC-AAATAATT-TC--------------------------ACA-CAC--

AATAG-A-AA-CCT------------TCC--T-TTG-------A-G--CC-C-T-GG-G---------A--G

TGATG-AAT-A--A-A-AA--A-TT-TA-TTGAG-AATCTTGT-GGGACGAA----TTT------CGG----

-------------GCTCAAACCCAAG-C----------------C-AAA-ACTTAACACACACA---A----

------------------TGA--C----GAA-TGTCTAG----------T--A-AAAATTTA-TGA-CCA--

--AATGACCC-------AAGGA-AT-----AAGGC---GT-----AGTA------AG-TCATAGA-CCGAGA

GTCAA-------------------------------------------------------------------

------------------------------------------------------------------------

--------------C--AAATCT--------------------------GG-----------------TTTT

C----------------------GGGAAACAAAA--C--GTCCTGAC-----TTTTCTTCCC----------

--------------TATATT--TCCT-----------TTTTGTGA---------------------------

-------------------------TTT--GTTTAT-GGAC--GTG-TCT-CG-TTACCT--CGGTGC--AA

AC-----------AACAT-A----TGAAAGT-AATTGTG-T-------------AAA---TTTTT----TCC

GAATTTTACA------------------------------ACGAAAT-A-C-G-GA-CCC-AGA-----AT-

AAAATT--GTAGAAA--GTAGG------------GCGAAATTT--CACA-A-GGT-TGGG--TAGAG-----

---------GAT-AGCCT-------------------TGG-TTTGTACA----AAATTTCTGAAAAATTC

>Pv01Sk01690/1-550 Pv01Sk01690 undefined product 1880945:1881494 reverse

CTCCTGAAATAA---TTTTTTTTTTGG--AAATTCGACGC-AAG-AA--T-------CA---TGATTG-AA-

TTTTGA-AC----------AAAATCCG-AC-AA-AT--TTCAGG--TC------------------------

-----------------------------------------AAAC-G--GATGAC---TATTCAC-------

---TTA-----GAAAAAAA----TC-AAACAGTT-TC--------------------------ACA-CAC--

AAAC--G-AA-CCT------------TCC--T-TTG-------A-G--CC-C-T-GA-C---------A--G

TGATG-AAT-A--A-A-AA--A-AT-TA-TTGCG-AATTTTGT-GGGATGAA----TTT------TGG----

-------------GCTCAAACCCAA--C----------------C-AAA-ACTCAATTCACACA---A----

------------------TGA--T----GAA-TGTCTGG----------T--A-AAAATTTC-AGA-CCA--

--AAATAACC-------TAGTA-AT-----AAGGT---GT-----AGTA------AG-TCACAGA-CCGAGA

GTTAA-------------------------------------------------------------------

------------------------------------------------------------------------

--------------G--AAAACA--------------------------AG-----------------ATTT

C----------------------GGAAAAACAAA--C--TTCATGGC-----TTTTCTTCCC----------

--------------TATATC--TACT-----------TTTTGTGA---------------------------

-------------------------TTT--GTTTAT-GGAC--GTG-CCT-CA-TTACCT--GGGTAC--AA

AC-----------AATAT-A----TGAAAGT-ACTTGTG-T-------------GAT---TTTTT----TTT

CAATTTTTCA------------------------------ACGCAAT-A-C-G-GA-TCC-TGA-----AT-

AAAATT--GTACAAA--GTAAG------------GCGAAATTT--CACA-A-GTT-TTGG--TAGAG-----

---------GAT-AGTCT-------------------TGG-TTTGCACA----AAATTTCTGAAAAATTC

>Pv01Sk01700/1-539 Pv01Sk01700 undefined product 2016384:2016922 reverse

CTCCCAAAATAG-----TTTTTTCCTA--AAATTTCACGT-AAG-AA--T-------CT---CCACTG-AA-

TTTGGG-AC----------AGAACGCG-AC-AA-AT--TTCAGG--TC------------------------

-----------------------------------------AAAC-G--GATGAG---TATTCAC-------

---CCA-----CGAAAAAAA---TC-AATCAGTT-TC--------------------------GCA-CAC--

AAAC--G-AA-CCC------------TCC--T-TTC-------A-G--CC-C-T-GA-C---------A--G

TGATG-AAT-A--A-A-AA--A-TT-TA-TTGTC-AATCTTGT-GGGACGAA----TCT------GGG----

-------------GCTCAAATCTCAG-T----------------C-AAA-ACTCAGCAGACACG---G----

------------------TGA--C----GAA-CGTCTGG----------T-AA--AAATTTC-AGA-CCA--

--AAATACCC-------AAGGA-GT-----AAGGC---GT-----AGTA------AG-TCCCAGA-CCGAGA

GCGAA-------------------------------------------------------------------

------------------------------------------------------------------------

--------------C--AAAAGT--------------------------GG-----------------TTTT

C----------------------CGAAAACTAAA--C--GTCATGGC-----TTTTCTTCTC----------

--------------CGTATA--TTGT-----------TTTTGTAA---------------------------

-------------------------TTT--GTTTAT-GGAC--GTG-CCT-CC-TTACCT--GGGTGC--AA

AA-----------AACGT-A----GGAAAGT-ACTTGTG-T-------------GAA---------------

--TTTTTT-G------------------------------GCGCAAT-A-C-G-GA-CCC-AGA-----AT-

AAAATT--GCAGAAA--GTATG------------GCCAAATTT--CACG-A-GTT-TTGG--TAGAG-----

---------GAT-AGCCT-------------------TGG-TTTGCACA----AAATATCTGAAAAATTC

>Pv01Sk01710/1-540 Pv01Sk01710 undefined product 2016922:2017461 reverse

CTCCCGAAATAG----TTTTTTTCGTG--AAATTCCAAGT-AAG-AA--T-------CT---CCACTG-AA-

TTTGAG-AC----------AGAACGCG-AG-AA-AT--TTCAGG--TC------------------------

-----------------------------------------AAAC-T--GATGAT---T-TTCAC-------

---CCA-----CGAAAAAAA---TC-AAACAGTT-TC--------------------------GCT-CAT--

AAAC--G-AA-CCC------------TCT--T-TTC-------A-G--CC-C-T-GG-C---------A--G

TGATG-AAT-A--A-A-AA--A-TT-TA-TTGTC-ATTCTTGT-GGGACTAA----TCG------GGG----

-------------GCTCAAATCTCAA-C----------------C-AAA-ACTCAACAGACACG---G----

------------------TGA--C----GAA-CGTCTGG----------T-AA--AAATTTC-AGA-CCA--

--AAATACCC-------AAGTA-GT-----AAGGC---GT-----AGTA------AG-TCCCAGA-CCGAGA

GCGAA-------------------------------------------------------------------

------------------------------------------------------------------------

--------------C--AAAAGT--------------------------AG-----------------TTTT

C----------------------CGAAAACAAAA--C--GTCCTGGC-----TGTTCTTTCC----------

--------------CCTATC--TTCT-----------TTTTGTAA---------------------------

-------------------------TTT--GTTTAT-GGAC--GTG-CCT-CC-TTACCT--GGGTGC--AA

AA-----------AACAT-A----GGAAAGT-ACTTGTG-T-------------GA----------------

-ATTTTTTCG------------------------------GCACAAT-A-C-G-GA-CCC-AGA-----AT-

AAAATT--GCAGAAA--GTAAA------------GCCAAATTT--CACG-A-GTT-TTAG--TAGAG-----

---------GAT-AGCCT-------------------TGG-TTTGCACA----AAATTTTTGAAAAATTC

>Pv01Sk01720/1-542 Pv01Sk01720 undefined product 2017461:2018002 reverse

CTCCCGAAATAG----TTTTTTTCGTG--AAATTCCACGT-AAG-AA--T-------CT---CCACTG-AA-

TTTGAG-AC----------AGAACGCG-AG-AA-AT--TTCAGG--TC------------------------

-----------------------------------------AAAC-A--GATGAT---T-TTCAC-------

---CCA-----CGAAAAAAA---TC-AAACAGTT-TC--------------------------GCA-CAC--

AAAC--G-AA-CCC------------TCT--T-TTC-------A-G--CC-C-T-GG-C---------A--G

TGACG-AAT-A--A-A-AA--A-TT-TA-TTGCC-AATCTTGT-GGGACGAA----TAT------GGG----

-------------GCTCAAATCTCAG-T----------------C-AAA-ACTCAACAGACATG---G----

------------------TGA--C----GAA-CGTCTGG----------T-AA--AAATTTC-AGA-CCA--

--AAATACCC-------AAGGA-GT-----AAGGC---GT-----AGTA------AG-TCCCAGA-CCGAGA

GCGAA-------------------------------------------------------------------

------------------------------------------------------------------------

--------------A--AAAACT--------------------------GG-----------------TTTT

C----------------------CGAAAATAAAA--C--GTCCTGGTT----TTTTCTTCCC----------

--------------CATATC--TTAT-----------TTTTGTGA---------------------------

-------------------------TTT--GTTTAT-GGAC--GTG-CCT-CC-TTACCT--TGGTGC--AA

GC-----------AACAT-A----GGAAAGT-ACTTGTG-T-------------GAT---------------

-ATATTTTCG------------------------------GCGCAAC-A-C-G-GA-CCC-AGA-----AT-

AAAATT--GCAGAAA--GTATG------------GCCAAATTT--CACG-A-GTT-TTGG--TAGAG-----

---------GAT-AGCCT-------------------TGG-TGCGCACA----AAATTTATGAAAAATTC

>Pv01Sk01730/1-541 Pv01Sk01730 undefined product 2018002:2018542 reverse

CTCCCGAAATAC-----TTTTTTCCTG--AAATTCCACGA-AAG-AA--T-------CT---CCACTG-AA-

TTTGGG-AC----------ACAACGCG-AC-AA-AT--TTCAGG--TC------------------------

-----------------------------------------AAAC-G--GATGAG---TATTCAC-------

---CCA-----CAAAAAAAA---TC-AAACAGTT-AC--------------------------GTA-CAC--

AAAC--G-AA-TCC------------TCC--T-TTC-------A-A--CC-A-T-GA-C---------A--G

TGATG-AAT-A--A-A-AA--A-TT-TA-TTTAC-AATCTTGT-GAGACGAA----TCT------GGG----

-------------GCTCAAATCTTAG-C----------------C-AAA-ACTAAACAGGCACG---G----

------------------TAA--C----GAA-CGTCTGG----------T-AA--AAATTTC-AGA-CCA--

--AAATACCC-------AAGGA-GT-----AAGGC---GT-----AGTA------AG-TCCCAGA-CCGAGA

GCGAA-------------------------------------------------------------------

------------------------------------------------------------------------

--------------A--AAAACT--------------------------GG-----------------TTTT

T----------------------CGAAAACAAAA--C--GTCCTGGT-----GTTTTTTCCC----------

--------------CGTATA--TTAT-----------TTTTGTGA---------------------------

-------------------------TTT--GTTTAT-GGAC--GTG-CCT-CC-TTACCT--TGGTGC--AA

GC-----------AACAT-A----GGAAAGT-ACTTGTG-T-------------GAT---------------

-ATATTTTCG------------------------------GCGCAAC-A-C-G-GA-CCC-AGA-----AT-

AAAATT--GCAGAAA--GTAGG------------GCCAAATTT--CACG-A-GTT-TTGG--TAGAG-----

---------GAT-AGCCT-------------------TGG-TGCGCACA----AAATTTATGAAAAATTC

>Pv01Sk01740/1-531 Pv01Sk01740 undefined product 2018542:2019072 reverse

-TCCCGAAATAG-----TTTGTTCTTG--AAATTCCACGT-A------------------------TG-AA-

TTTAGG-AC----------AGAACGCG-AC-AA-AT--TTCAGG--TC------------------------

-----------------------------------------AAAC-G--GATGAG---TATTCAC-------

---CCA-----CGAAAAAAA---TC-AAACAGTT-TC--------------------------TCA-GAC--

AAAC--G-AA-CCC------------TCA--T-TTC-------A-T--CC-C-T-GG-C---------A--G

TGATG-AAT-A--A-A-AA--A-TT-TA-TTACC-AATCTTGT-GGGACAAA----TCT------GGG----

-------------GCTCAAATCTCAG-C----------------C-AAA-ACTCAACAGACACA---G----

------------------TGA--C----GAA-CGTTTGG----------T-AA--AAATTTC-AGA-CCA--

--AAATACCC-------AAGGA-GT-----AAGGC---GT-----AGTA------AG-TCCCAGA-CCAAGA

GAAAA-------------------------------------------------------------------

------------------------------------------------------------------------

--------------C--AAAACT--------------------------AA-----------------TTTT

C----------------------CGGAAAAAAAAAAAC-GTCCTGGT-----TGTTCTTCCC----------

--------------CGTATC--TTCT-----------TTTTGTGA---------------------------

-------------------------TTT--GTTTAT-GGGC--GTG-CCT-CC-TTTACT--GGGTGC--AA

AC-----------AACAT-A----GGAAACT-ACTTGTG-T-------------GAA---------------

--TATTTTCT------------------------------GTGCAAT-A-C-G-GA-CCC-AGA-----AT-

AAAATT--GCAGAAA--GTATG------------GCCAAATTT--CACA-A-GTT-TTGG--TAGAG-----

---------TAT-AGCCT-------------------TGG-TTCGCAAA----AAATTTCTGAAAAATTC

>Pv01Sk01750/1-533 Pv01Sk01750 undefined product 2019077:2019609 reverse

CTCCTAAAATAG-----TTTTTTCCTG--AAATTCCACGT-AAA-AA--T-------CT---CCACTG-AA-

TTTGGG-AC----------AGAACTCG-AC-AA-AT--TTCAGG--TC------------------------

-----------------------------------------AAAC-G--GATGAG---TATTCAC-------

---CCA-----CGAAAAAAA---TC-AAACAGTT-TC--------------------------GCA-CAC--

AACC--A-AA-TCC------------TCC--T-TTC-------A-G--CC-C-T-GG-C---------A--G

TGATG-AAT-A--A-A-AA--A-TT-TA-TAGCC-AATCTTGT-GGGACGAA----TAT------GGG----

-------------GCTCAAATCTCAG-C----------------C-AAA-ACTCAACACACACG---G----

------------------TGA--C----GAA-CGTCTGG----------T-AA--AAATGTC-AGG-CCA--

--AAATACC---------AGGA-GT-----AAGGT---GT-----AGTA------AG-TACCAGA-CCGAGA

GCGAA-------------------------------------------------------------------

------------------------------------------------------------------------

--------------C--AAAACT--------------------------CG-----------------TTTT

C----------------------CGAAAAGAAAA--C--GTCCTGGC-----TTTTCTTCCC----------

--------------CGTATC--TTCC-----------TTTTGTGA---------------------------

-------------------------TTT--GTTTAT-GGAC--GTG-CCT-CC-TTACTT--GGGAGT--AA

AC-----------AACAT-A----GCAAAGT-ACTTGCG-T-------------GAA---------------

--TTTTTTCG------------------------------GCGTAAT-A-C-G-GA-TCC-AGA-----AT-

AAAATT--CCAGAAA--GTATG------------GCCAAATTT--CACG-A-GTT-TTGG--TAGAG-----

---------GAT-AGCCT-------------------TGG-TTTGCACA----AAATTTTTAAAA-----

>Pv01Sk01760/1-535 Pv01Sk01760 undefined product 2019610:2020144 reverse

CTCCCGAAATAG-----TTTTCTTTTG--AAATTCCACAA-AAG-AA--T-------CT---CCACTG-AA-

TTTGGG-AC----------AGAACGCG-AC-AA-AT--TTCAGG--TC------------------------

-----------------------------------------AAAC-G--GATGAG---TATTCAC-------

---CCA-----TGAAAAAAA---TC-AAACAGTT-TC--------------------------GCA-CAC--

AAAC--G-AA-CCC------------TCC--T-TTC-------A-G--GC-C-T-GA-C---------A--G

TGATG-AAT-A--A-A-AA--A-TT-TA-TTGTC-AATCTTGT-GGGACGAA----TCT------GGG----

-------------GCTCAAACCTCAG-C----------------C-AAA-ACTCAACAGACACG---G----

------------------TGA--C----GAA-CGTCTGG----------T-AA--AAATTTC-AGA-CCA--

--AAATACTC-------AAGGA-GT-----AAGGC---GT-----AGTA------AG-TCCCAGA-CCGAGA

GCGAA-------------------------------------------------------------------

------------------------------------------------------------------------

--------------C--AAAACA--------------------------GG-----------------TTTT

C----------------------CAAAAACAAAA--C--CTCTTGGC-----TTTTCTTCCC----------

--------------CGTATC--TTCC-----------TTTTGTGA---------------------------

-------------------------TTT--GTTTAT-GAAC--GTG-CCT-CC-TTACCT--GGGAGC--AA

AC-----------AACAT-A----GGAAAGT-ACTTGTG-T-------------GAA---------------

--TTTTTTCG------------------------------GTGCAAT-A-C-G-GA-CCC-AGA-----AT-

AAAATT--GCAGAAA--GTAGG------------GCCAAATTT--CACG-A-GTT-TTGG--TAGAG-----

--------------TCCT-------------------TGA-TTTGCACA----AAAGTTCTGAAAAGTT-

>Pv01Sk01770/1-540 Pv01Sk01770 undefined product 2020144:2020683 reverse

CTCCTAAAATAG-----TTTTTTCCTG--AAATTCCACGT-AAA-AA--T-------TT---CCACTG-AA-

TTTGGG-AC----------AGAACTCG-AC-AA-AT--TTCAGG--TC------------------------

-----------------------------------------AAAC-G--GATGAG---TATTCAA-------

---CCA-----CGAAAAAAA---TC-AAAAAGTT-TC--------------------------GCA-CAC--

AAAC--G-AA-CCC------------TCC--T-TTC-------A-T--CC-C-T-GG-C---------A--G

TGATG-AAT-A--A-A-AA--A-TT-TA-TAGTC-AATCTTGT-GGGATGAA----TAT------GGG----

-------------GCTCAAATCTCAG-C----------------C-AAA-ACTCAATAGACACG---G----

------------------TGA--C----GAA-CGTCTGG----------T-AA--AAATGTC-AGG-CCA--

--AAATACCC-------AAGGA-GT-----AAGGC---GT-----AGTA------AG-TTCCAGA-CCGTGA

GCGAA-------------------------------------------------------------------

------------------------------------------------------------------------

--------------C--AAAACT--------------------------GG-----------------TTTT

C----------------------CGAAAACAAAA--C--GTCCTGGC-----TTTTCTTCCC----------

--------------CGTATC--TTCT-----------TTTTGTGA---------------------------

-------------------------TTT--TTTTAT-GGAC--GTG-CCT-CC-TTACCT--GGGTGC--AA

AC-----------AACAT-G----AGAAAGT-ACTTGTG-T-------------GAA---------------

--TTTTTTCG------------------------------GCGCAAT-A-C-A-GA-GCC-AGA-----AT-

AAAATT--GAAGAAA--GTAGG------------GCCAAATTT--CACG-A-CTT-TTGG--TAGAG-----

---------GAT-AGCCT-------------------TGG-TTTGCACA----AAATTTCTGAAAAATTC

>Pv01Sk01790/1-540 Pv01Sk01790 undefined product 2020980:2021519 reverse

CTCCCGAAATAG-----TTTTTTCCTG--AAATTCCACGT-AAG-AA--T-------CT---CCACTG-AA-

TTTGGG-AT----------GGAACGCG-AC-AA-AT--TTCAGG--TC------------------------

-----------------------------------------AAAA-G--GATGAG---TATTCAC-------

---CCA-----CGAAAAAAA---TC-AAACAGTT-TC--------------------------ACA-CAC--

AAAC--G-AA-CCC------------TCC--T-TTC-------A-A--CC-C-T-GA-C---------A--G

TGATG-ACT-A--A-A-AA--A-TT-TA-TTGTC-AATCTTGT-GCGACTAA----TCG------GGG----

-------------GCTCAAATCTCAG-C----------------C-AAA-ATTCAACAGAAACG---G----

------------------AGA--C----GAA-CGTCTGG----------T-AA--AAATTTC-ATA-CCA--

--AAATACCG-------AAGGA-GT-----AAGGC---GT-----AGTA------AG-TCCCAGA-CCAAGA

GCGAA-------------------------------------------------------------------

------------------------------------------------------------------------

--------------C--AAAATT--------------------------GG-----------------TTTC

C----------------------CGAAAACAAAA--C--GTCCTGAC-----CTTTCTTTTC----------

--------------CGTACC--TTCT-----------TTTTGTGA---------------------------

-------------------------TTT--GTTTAT-GGAC--GTG-CCT-CC-TTACCT--GAGTGC--AA

AG-----------AACAT-A----GGATAGT-ACTTGTG-T-------------TAA---------------

--TATTTTTG------------------------------GTGCAAT-A-C-G-GA-CCC-ACA-----AT-

AAAATT--GCAGAAA--GTAGG------------ACCAAATTT--CACA-A-GTT-TTGG--TAGAG-----

---------GAT-AGCCT-------------------TGA-TTTGCACA----AAATTTCTGAAAAATTC

>Pv01Sk01800/1-540 Pv01Sk01800 undefined product 2021519:2022058 reverse

CTCCCGAAATAG-----TTTTTTCTTG--AAATTCCACGT-AAG-AA--T-------CT---CCACTG-AA-

TTTGGG-AC----------AGATCGCG-AC-AA-AT--TTCAAG--TC------------------------

-----------------------------------------AAAC-G--GGTGAG---TATTCAC-------

---GCA-----CGAAAAAAA---TC-AAATAGCT-TC--------------------------GCA-CAC--

AAAC--G-AA-CCC------------TCT--T-TTC-------A-G--CC-C-T-GA-C---------A--G

TGATG-AAT-A--A-A-AA--A-TT-TA-TTGCC-AATCTTGT-GGGACGAA----TTT------GGG----

-------------GCTCAAATCTCAG-C----------------C-AAA-ACTCATTAGACACA---G----

------------------TGA--C----GAA-CGTCTGG----------T-AA--AAATTTC-AGA-CCA--

--AAATACCC-------AAGGA-GT-----AAGGC---GT-----AGTT------AG-TCCCAGA-CCGAGA

GCGAA-------------------------------------------------------------------

------------------------------------------------------------------------

--------------C--AACACT--------------------------GG-----------------TTTT

C----------------------CGAAAACAAAA--C--GTCATGGC-----TTTTCTTCCC----------

--------------CATATC--TTCT-----------TTTTGTGA---------------------------

-------------------------TTT--GTTTAT-GGAC--GTG-CCT-CC-TTACCT--GGGTGC--AA

AC-----------AACAT-A----GGAAAGT-ACTTGTG-G-------------GAA---------------

--TTTTTTCG------------------------------GCACAAT-A-C-G-GA-CCC-AGA-----AT-

AAAATT--GCAGAAA--GTAGG------------GCCAAATTT--CACA-A-GTT-TTGG--TAGAG-----

---------GAT-AGCCT-------------------TGG-TTTGCACA----AAATTTCTGAAAAATTC

>Pv01Sk01810/1-540 Pv01Sk01810 undefined product 2022058:2022597 reverse

CTCCCAAAATAG-----TTTTTTCCTG--AAATTCCACGT-AAG-AA--T-------CT---CCACTG-AA-

TTTGGG-AC----------AGAACGCA-AC-AA-AT--TTCAGG--TC------------------------

-----------------------------------------AAAC-G--GATGAG---TATTCAC-------

---CCA-----CGAAAAAAA---TC-AAACAGTT-TT--------------------------GCA-CAC--

AAAC--G-AA-CCC------------TCC--T-TTC-------A-G--CC-C-T-AG-C---------A--G

TGATG-AAT-A--A-A-AA--A-TT-TA-TTGTC-AATCTTGT-GAGACGAA----TCT------GGG----

-------------GCTCAAATCTCAG-C----------------C-AAA-ACTCAACAGACACA---G----

------------------TGA--C----AAA-CGTCTAG----------T-AA--AAATTTC-AGA-CCA--

--AAATACCC-------AAGGA-GT-----AAGGC---CT-----AGAA------AG-TCCCAGA-CCGAGA

GCGAA-------------------------------------------------------------------

------------------------------------------------------------------------

--------------C--AAAACT--------------------------GG-----------------TTTT

T----------------------CGAAAACAAAA--C--GTCCTGAC-----TTTTCTTCCC----------

--------------CGTATC--CTCT-----------TTTTGTGA---------------------------

-------------------------TTT--TTTTAC-GGAC--ATG-CCT-CC-TTACCT--GGGTGC--AA

AC-----------AACGT-A----GGAAAGT-TCTTGTG-T-------------GAA---------------

--TTTTTTCG------------------------------GCGCAAT-A-C-G-GA-CCC-AGA-----AT-

AAAATT--GCAGAAA--GTATG------------ACCAAATTT--CACA-A-GTT-TTGG--TAAAG-----

---------GAT-AGCCT-------------------TGG-TTTGCACA----AAATTTCTGAAAAATTC

>Pv01Sk01830/1-541 Pv01Sk01830 undefined product 2300332:2300872 reverse

CTCTCGAAATAG------TTTTTCCTG--AAATTCCACTT-AAG-AA--T-------CT---TCACTG-AA-

TTTGGG-AC----------AAAACGTG-AA-AA-AT--TTCAGG--TC------------------------

-----------------------------------------AAAC-G--GACGAA---TATTCGT-------

---CCA-----CGAAAAAAA---TC-AAACAGTT-TC--------------------------ACA-CAC--

AAAC--G-AA-CCT------------TCC--T-TTC-------A-G--CC-C-T-GG-T---------A--G

TGATG-AAT-A--C-G-AA--A-TT-TA-TTGCC-AATCTTGT-GGGACGAA----TCT------GGA----

-------------GTTCAAATCTCAA-C----------------C-AAA-A-TCAGTAGACACA---G----

------------------TGA--C----GAA-CGTCTAG----------T-AA--AAATTTC-AAA-CCA--

--GAATGCCT-------AAGGA-GT-----AAGGC---GT-----AGTA------AG-TCCCAGA-CCGAGA

GTGAA-------------------------------------------------------------------

------------------------------------------------------------------------

--------------C--AAATTT--------------------------GG-----------------TTTT

C----------------------CGAAAACATAA--C--GTCATCGC-----TTTTCTCCCC----------

--------------TGTATC--TTCT-----------TTTTGTGA---------------------------

-------------------------TTT--GTTTAT-GGAC--GTG-CCT-CC-TTACCT--GGGTGC--AA

AC-----------AACAT-A----TGAAAGT-ACTCGTG-T-------------GAA---------------

--TTTTTTTA------------------------------GCGCAAT-A-C-G-GA-CCT-AGA-----AT-

ACAATT--TCGGAAA--GTAGG------------GCGAAATTT--CACA-A-GTT-TTGG--TAGAGGAT--

---------GAT-AACCT-------------------TGG-TTTGCACA----AAATTTCTGAAAAATTC

>Pv01Sk01840/1-539 Pv01Sk01840 undefined product 2300872:2301410 reverse

-TACCGAAATAG-----TTTTTTCCTG--AAATTCGACGT-AAG-AA--T-------CT---TCACTG-AA-

TTTGGG-AC----------AAAACGCG-AT-AA-AT--TTCAGG--TC------------------------

-----------------------------------------AAAC-G--GACGAG---TATTCGT-------

---CCA-----C-AAAAAAA---TC-AAATAGTT-TC--------------------------ACA-CAC--

AAAC--G-AA-CCT------------TCC--T-TTC-------A-G--CC-C-T-GG-C---------A--G

TGAAG-AAT-A--A-A-AA--A-TT-TA-TTCCA-AATCTTTT-GGGACAAA----TCT------GGG----

-------------GCTCAAATCTCAG-C----------------C-AAA-ACTCAGTACACACA---G----

------------------TGA--C----GAA-CGTCTGG----------T-AA--AAATTTC-AGA-CCA--

--GAATGCTC-------AAGGA-GT-----AAGGC---GT-----AGCA------AG-TCCCAGA-CCGAGA

GTTAA-------------------------------------------------------------------

------------------------------------------------------------------------

--------------C--AATACT--------------------------GG-----------------TTTT

C----------------------CGAAAAAAAAA--C--GTCTTGGC-----TTTTCTCCCC----------

--------------CGTATC--TTCT-----------TTTTGTGA---------------------------

-------------------------TTT--TTTTAT-GGAC--GTG-GCT-CC-TTACCT--GGGTGC--AA

AC-----------AACAT-A----TGAAAGT-AGTCGTG-T-------------GAA---------------

-TTTTTTTAA------------------------------GCGCAAT-A-C-G-GA-CCC-AGA-----AT-

ACAATT--TCGAAAA--GTAGG------------GCGAAATTT--CACA-A-GTT-TTAG--TAGAG-----

---------GAT-AGTCT-------------------TAC-TTTGCACA----AAATTTCTGAAAAATTC

>Pv01Sk01850/1-541 Pv01Sk01850 undefined product 2301411:2301951 reverse

-TTCCAAAATAG----TTTTTTTCCTG--AAATTCCACGT-AAG-AA--T-------CT---TCACTG-AA-

CTTGGG-AC----------AAAACGCG-AT-AA-GT--TTCAAG--TC------------------------

-----------------------------------------AAAC-G--GACGAG---TATTCGC-------

---CCA-----CGATAAAAA---TC-AAACAGCT-TC--------------------------ACA-CAC--

AAAC--G-AA-CTT------------TCC--T-TTC-------A-G--CC-A-T-GG-C---------A--G

TGATG-AAT-A--A-A-AA--A-TT-TA-TTGCC-AATATTTT-GGGACGAA----TCT------GGA----

-------------GCTCAAATCTCAG-C----------------C-AAA-ACTCAGTACAGACA---G----

------------------TGA--C----TAA-CGTCTGG----------T-AA--AAATTTC-ATA-CCA--

--GAATGCCC-------AAGGA-GT-----AAGGC---AT-----AGCA------AG-TCCCAGA-CCGAGA

GTGGA-------------------------------------------------------------------

------------------------------------------------------------------------

--------------C--AAAACT--------------------------GG-----------------TTTT

C----------------------CGAAAACAAAA--C--GTCCTGGC-----TTTTCTCCCC----------

--------------CGTATC--TTCT-----------TTTTGTGA---------------------------

-------------------------TTT--GTTTAT-GGAC--GTG-CCT-CC-TTACCT--GGGTGC--AA

AA-----------AACAT-A----TGAAAGC-ACTCATG-T-------------GAA---------------

-TTTTTTTAA------------------------------GCACAAT-A-C-G-AA-CCC-AGA-----AT-

ACAATT--TCGGTAC--GTAGG------------GCGGAATTT--CACA-A-GTT-TTGG--TAAAA-----

---------GAT-AGCCT-------------------TGG-TTTGCACA----AAATATCTGAAAAATTC

>Pv01Sk01860/1-539 Pv01Sk01860 undefined product 2301953:2302491 reverse

CTCCCGAAATAG-----TTTTTTCCTG--AAATTCCACGT-ATA-AA--T-------CT---CCACTG-AA-

TTTGAG-AC----------AAAATGCG-AC-AA-AT--TTTAAG--TC------------------------

-----------------------------------------AAAC-G--CACGAG---TATTCGC-------

---CTA-----CGAAAAAAA---TC-AAACAGTT-TT--------------------------ACA-CAC--

AAAC--G-AA-CCT------------TCT--T-ATC-------A-G--TT-C-T-GG-C---------C--G

TGATG-AAT-A--A-A-AC--A-TT-TA-TTGCG-AATCTTGT-GGGACGAA----TCT------GAG----

-------------GCACAAATCTCAA-C----------------C-AAA-ACTCAGTAGAAACA---G----

------------------TGA--C----GAA-CGTCTGA----------T-AA--AAATTTC-AGA-CCA--

--GAATGCCC-------AAGGA-GT-----AAGGC---GT-----AGTA------AG-TCCCAGA-CCGAGA

GTGAA-------------------------------------------------------------------

------------------------------------------------------------------------

--------------C--AAAACT--------------------------GG-----------------TTTT

T----------------------CGAAAACAAAA--C--GTCCTGGC-----TTTTCTCCCC----------

--------------CGTATC--TTCT-----------TTTTGTAA---------------------------

-------------------------TTT--ATTTAT-GGAC--GTG-CCT-CA-TTACCT--TGGTGC--AA

AG-----------AACAT-A----TGAAAGT-ACTCGTG-T-------------GAA---------------

--TTTTTTAA------------------------------GCGCAAT-A-C-G-GA-CCC-AGA-----AT-

ACAACT--TCGGAAC--GTAGG------------GCGAAATTT--CACA-A-GTT-TTGG--TAGAA-----

---------GAT-AGCCT-------------------TGG-TTTGCACA----AATTTTTTGAAAAGTT-

>Pv01Sk01870/1-541 Pv01Sk01870 undefined product 2302491:2303031 reverse

CTCCCGAAATAG-----TTTTTTCCTG--AAATTCCACAT-AAG-AA--T-------CT---CCACTG-AC-

TTTGGG-AT----------AAAACGCG-AC-AA-AT--TTCAGG--TC------------------------

-----------------------------------------AAAC-G--GACAAG---TATTCGC-------

---CCA-----CGAGAAAAA---TC-AAACAGTT-TC--------------------------ACT-CAC--

ACAC--G-AA-CCT------------TCC--T-TTC-------A-G--TT-C-T-GG-C---------A--A

TGATG-AAT-A--A-A-AT--T-TT-TA-TTGCC-AATCTTTG-TTGACGAA----TCT------GGG----

-------------GCTCAAAACTCAG-C----------------C-AAA-ACTCAGTAGACACA---G----

------------------TTA--C----GAA-CGTCTGG----------T-AA--AAATTTC-AGA-CCA--

--GAATGTCT-------AAGGA-GT-----AAGGC---GT-----AGTT------AT-TCCCAGA-CCGAGA

TTGAA-------------------------------------------------------------------

------------------------------------------------------------------------

--------------C--AAAACT--------------------------GG-----------------TTTT

C----------------------CGAAAACAAAA--C--GTCCTGGG-----TTTTCTCCCC----------

--------------CCTATC--TTCT-----------TTTTGTGA---------------------------

-------------------------TTT--GTTTAT-GGAC--GTG-CTT-CC-TCACCT--GGGTGC--AA

AC-----------AACAT-A----TGAAAGT-ACTCGTG-T-------------GAA---------------

-TTATTTTCA------------------------------GTGCAAT-A-T-G-GA-TCC-AGA-----AT-

ACAATT--TCGGAAC--ATAGG------------GCGAAATTT--CACA-A-GTT-TTAG--TAGAG-----

---------GAT-AGCCT-------------------TGG-TGTGTACA----AAATTTCTAAAAAATTC

>Pv01Sk01880/1-538 Pv01Sk01880 undefined product 2303031:2303568 reverse

----CGAAATAG-----TTTTTTCCTG--AAATTCCACAT-AAG-AA--T-------CT---TCACTG-AA-

TTTGGG-AC----------AAAACGCG-AC-AA-AT--TTGAGG--TC------------------------

-----------------------------------------AAAC-A--GACGAG---TATTCGC-------

---CCA-----CGAAAAAA----TC-AAACAGTT-TC--------------------------ACA-CAC--

AAAC--G-AA-CCT------------TCC--T-TTC-------A-G--CC-C-T-GG-C---------A--G

TGATG-AAT-A--A-T-AA--A-TT-TG-TTGCC-AATCTTGT-GGGACGAA----TCT-------GG----

-------------GCTCAAATCTCAA-C----------------C-AAA-ACTCAGTAGACACA---G----

------------------TGA--C----GAA-TGTCTGG----------T-AA--AAAATTC-AAA-CCA--

--GAATGCCC-------AAGGA-GT-----AAGGC---GT-----AGCA------AG-TCCCAGA-CCGAGA

GTAAC-------------------------------------------------------------------

------------------------------------------------------------------------

--------------C--AAAACT--------------------------GA-----------------TTTT

C----------------------AAAAAACAAAA--C--GTCCTGGT-----TTTTCTCCCC----------

--------------CGTATC--TTCT-----------TTTTCTGA---------------------------

-------------------------TTT--TTTAAA-GGAC--GTG-CCT-CC-TCACTT--GGGTGC--AT

CC-----------AACAT-A----TGAAATT-ACTCGTG-T-------------GAA---------------

--TTTTTTCA------------------------------GCACAAT-A-C-G-GA-CCA-AGA-----AT-

ACAATT--TCGAAAA--GTAGG------------GCGAAATTT--CACA-A-GTT-TTGG--TAGAGAATA-

---------AAT-AGCCT-------------------TGG-TTTGCACA----AAACTTCTGAAATATTC

>Pv01Sk01900/1-538 Pv01Sk01900 undefined product 2303700:2304237 reverse

CTCCCGAAATAG----TTTTTTTCCTG--AAGTTCAACGT-AAG-AA--T-------CT---CCACTG-AA-

TTTGGG-AC----------AAAAGGCG-AC-AA-AT--TTTAGG--TC------------------------

-----------------------------------------AAAC-A--GACGAA---TATTTGC-------

---CCA-----CGAAAAAAA---TC-AAACAGTT-TC--------------------------ACA-CAA--

AAAC--G-AA-TCT------------TCC--T-TTC-------A-G--CT-C-T-GA-C---------A--G

TGATG-AAT-A--A-A-AA--T-TT-TA-TTGCC-AATTTTCT-GAGACGAT----TCA------GAG----

-------------GCTCAAATCTCAA-C----------------C-AAA-ACTCAGTAGACACA---G----

------------------TGA--C----GAA-CGTCTGG----------T-AA--AAATTTC-AGA-CCA--

--GAATGCCC-------AAGGA-GT-----AAGGC---GT-----AGCA------AG-TCCCAGA-CCGAGA

GTGAA-------------------------------------------------------------------

------------------------------------------------------------------------

--------------C--AAAACT--------------------------GG-----------------TTTT

C----------------------CGAAAACAAAA--C--GTCCTGGC-----TTTTCTCCCC----------

--------------CGTATC--TTCT-----------TTTTGTGA---------------------------

-------------------------TTT--GTTTAT-GGAC--GTG-TCT-CC-TCACCT--GGAAGC--AA

AC-----------AACAT-A----TGAAAGT-ATTCGTG-T-------------GAA---------------

--TTTTTTCA------------------------------GCGCAAT-A-C-G-GA-CCT-AAA-----AT-

ACAATT--TCAGAAA--ATAGG------------GCGAAATTT--CACA-A-GTT-TTGG--TAGAG-----

---------GAT-AACCT-------------------TGG-TTTGCACA----AAACTTCTGAATAA---

>Pv01Sk01910/1-528 Pv01Sk01910 undefined product 2500850:2501377 reverse

CTCCCGAAATAG-----TTTTTTCCTG--AAATTCCACGT-AAG-AA--T-------CT---CCACTG-AG-

TTTGGG-AC----------AAAACGCT-AC-AA-AT--TTCAGG--TC------------------------

-----------------------------------------AAAC-G--GATGAG---TATTCGC-------

---CTC-----CGAAAAAAA---TC-AAACAGTT-TC--------------------------ACA-CAC--

TAAC--G-AA-CCT------------TCC--T-TTG-------A-G--TT-C-T-GA-C---------A--G

TGATG-AAT-T--A-A-AA--A-TT-TA-TTGTC-AATCTTGT-GGGACGAA----TCT------GGG----

-------------GCT----------------------------C-AAA-ACTCAGTAGACACA---G----

------------------TGA--C----GAA-CGTCTGG----------T-AA--AAATTTC-ATA-CCA--

--GAATGCC--------AAGGA-GT-----AAGGC---GT-----AGTA------AG-TCCCAGA-CCGAGA

GTGAA-------------------------------------------------------------------

------------------------------------------------------------------------

--------------A--AAAGCT--------------------------GG-----------------TTTT

T----------------------CGAAAACAAAA--C--GTCCTGGC-----TTTTCTCCCC----------

--------------CGTATC--TTCT-----------TTTTGTGA---------------------------

-------------------------TTT--GTTTAT-GGAC--ATG-CCT-CC-TTACCT--TAGTGC--AA

AC-----------AACAT-A----TGGAAGT-ACTCGTG-T-------------CAT---------------

--TTTTTTCA------------------------------GCACAAT-A-C-G-GA-CCC-ATA-----AT-

ACAATT--TTGGAAA--GTATG------------GCGAAATTT--CACC-A-GTT-TTGG--TAGAG-----

---------GAT-AGCCT-------------------TGG-TTTGCACA----AATTTTCTGAAAAATTC

>Pv01Sk01920/1-540 Pv01Sk01920 undefined product 2501377:2501916 reverse

CTCCCAAAATAG-----TTTTTTCCTG--AAATTCCACGT-AAG-AA--T-------CT---CCACTG-AG-

TTTGGG-AA----------ATAACGTG-AC-AA-AT--TTCAGG--TC------------------------

-----------------------------------------AAAC-G--TACGAG---TATTCGC-------

---CCA-----CGAAAAAAT---AC-AAACAGTT-TC--------------------------ACA-CAC--

AAGC--G-AA-CCT------------TCC--T-TTG-------A-G--CC-C-T-GA-C---------A--T

TGATG-AAT-A--A-A-AA--A-TT-TA-TTGTC-ACTCTTGT-GGCATAAA----TCT------GGG----

-------------GCTCAAATCTCAA-C----------------C-AAA-ACTCAGTAGACACT---G----

------------------TGA--C----GAA-CGTGTGA----------T-AA--AAATTTC-AGA-CCA--

--GAATGCCC-------AAGAA-GT-----AAGGC---GT-----AGTA------AG-TCCCAGG-CCGATT

GTGAA-------------------------------------------------------------------

------------------------------------------------------------------------

--------------C--AAAACT--------------------------GG-----------------CTTT

C----------------------CGAAAACAAAA--C--GTCCGGGC-----TTTTCTCTCC----------

--------------CGTATC--TTCT-----------TTTTGTGA---------------------------

-------------------------TTT--GGTTAT-GGAC--GTG-CCT-CT-TTACCT--GGGTGC--AA

AC-----------AACAT-A----TGGAAGT-ACTCGTG-T-------------GAA---------------

--GTTTTTCA------------------------------GCGCAAT-A-C-G-GG-CCC-AGA-----AT-

ACAATT--TCGGAAA--GTATG------------GCGAAATTT--CACA-A-GTT-TTGG--TAGAG-----

---------GAT-AGCCT-------------------TTG-TTTGCACA----AAATTACTGAAAAATTC

>Pv01Sk01930/1-540 Pv01Sk01930 undefined product 2501916:2502455 reverse

CTCCCGAAATAG-----TTTTTTCCTG--AAATTCCACGT-AAG-AA--T-------CT---CCATTG-AG-

TTTGGG-AC----------AAAACGCG-AC-AA-AT--TTCAGG--TC------------------------

-----------------------------------------AAAC-G--TACGAG---TATTCAC-------

---CCA-----CGAAAAAAA---TC-AAACAGTT-TC--------------------------ACA-CAC--

AAAC--G-AA-CCT------------TCC--T-TTG-------A-G--AC-C-A-GA-C---------A--G

TGATG-ATT-A--A-A-AA--A-GT-TA-TTGCC-AATATTGT-GGGACGAA----TCT------GGG----

-------------GCTCAAGTCTCAG-C----------------C-AAA-ACTTAGTAGACACA---G----

------------------TGA--C----GAA-TGTCTGG----------T-AA--AAATTTC-ACA-CCA--

--GAATGCCC-------AAGGA-GT-----AAGGC---GT-----AGTA------AG-TCCCAGA-CCGAGA

GTGAA-------------------------------------------------------------------

------------------------------------------------------------------------

--------------C--AAAACT--------------------------GG-----------------TTTT

C----------------------CGAAAACAAAA--C--GTCCTGAC-----TTTTCTCCTC----------

--------------CTTATC--TTCT-----------TTTTGTGA---------------------------

-------------------------TTT--GTTCAT-GGAC--TTG-CCT-TC-TTACTT--GGGTGC--AA

AC-----------AACTT-A----TGGAAGT-ACTCGTG-T-------------GAA---------------

--TTTTTTCA------------------------------CCGCAAT-A-C-G-GA-CCC-AGA-----AT-

ACAATT--TCAGAAA--GTAGG------------GCGAAATTT--CACA-A-GTT-TTTG--TAGAG-----

---------GAT-AGCCT-------------------TGG-TTTGCACA----AATTTTCTGAAAAATTC

>Pv01Sk01940/1-536 Pv01Sk01940 undefined product 2502455:2502990 reverse

-TCCCGAAATAG-----TTTTTTCCTG--AAATTCCACGT-AAG-AA--T-------CT---CCACTG-AG-

TTTGGG-AC----------AAAACTCG-AC-AA-AT--TTCAGG--TC------------------------

-----------------------------------------AAAC-G--GACGAG---TATTCGC-------

---CCA-----CGAAAAAAA---TC-AAACAGTT-TC--------------------------ACA-CAC--

AAAC--G-AA-CCT------------TCC--T-TTG-------A-G--CC-C-T-GG-C---------A--G

TGATG-ATT-A--A-A-AA--A-TT-TA-TTGCC-AATCTAGT-GGGACGAA----TCT------GGG----

-------------GCTCAAGTCTCAG-C----------------C-AAA-ACTCAGTAAACACG---G----

------------------TAA--C----GAA-CGTCTGG----------T-AA--AAATTTC-AGA-CCA--

--GAATGCCC-------AAGGA-GT-----AAGGC---GT-----AGTA------AG-TCCCAGA-CCGAGA

GTGAA-------------------------------------------------------------------

------------------------------------------------------------------------

--------------C--AAAACT--------------------------AG-----------------TTTT

C----------------------CGAAAACAAAA--C--GTCCTGGC-----TTTTCTCCCC----------

--------------CGTATC--TTCT-----------TTTTGTGA---------------------------

-------------------------TTT--GTTTAT-GGAA--GTG-TCT-CC-TTACCT--GGGTGC--AA

AC-----------AACAT-A----TGAAAGT-ACTCGTG-T-------------GAA---------------

--TTTTTTCA------------------------------CCGCAAT-A-C-G-GA-CCC-AGA-----AT-

ACAATT--TCGGAAA--GTAGG------------GCGAAATTT--CACA-A-GTT-TTGG--TA--------

---------GAC-CGCCT-------------------TGG-TTTACACA----AAATTTCTGAAAAATTC

>Pv01Sk01950/1-521 Pv01Sk01950 undefined product 4833246:4833766 forward

CTCCGGGAATAG-----TTTTTTCCTC--AAATTCTACCC-AAG-AA--T-------CT---CCACTG-AA-

TTTGGG-AT----------AAAACAAG-AC-AA-AT--TTCAGG--TC------------------------

-----------------------------------------AAAC-G--GATGA----TATTCAC-------

---CCA-----CGAAAAAAA---TC-AAACAGTT-TC--------------------------ACA-CAC--

AAAC--G-AA-CCG------------TCC--T-TTC-------A-G--CC-C-T-GG-A---------A--G

TGACG-AAT-A--A-A-AG--A-TA-TA-TAGCC-AATCTTGT-GGGACGAA----TCT------GGG----

-------------GCTCAAACCTGAG-C----------------CAAAA--CTCAATAGACACA---G----

------------------TGG--C----GAA-TGTCTGG----------T--A-AAAATTTC-AGA-CCA--

--AAATACCC-------AAGGA-GT-----AAGGC---GT-----AATA------AG-TCTCAGG-TCGAGA

GTGAA-------------------------------------------------------------------

------------------------------------------------------------------------

--------------C--AAAACC--------------------------GG-----------------TTTT

C----------------------CGAAAACAAAA--C--GTCCTGGC-----TTTTCGTCCC----------

--------------CGTATC--TTCT-----------TTTTGTGA---------------------------

-------------------------TTC--GTTTAT-GGAC--GTG-CCT-CC-TTGCCT--GGGTGC--AA

AC-----------AACAT-A----CGAAAGT-GCTTGTG-T-------------GAATTT------------

----TTTTCA------------------------------GCGCAAT-A-C-G-GA-CCC-AGA-----AT-

GAACTT--CGAGAAA--CTAGG------------CCAGAAATT-----------------------------

-----------A-GGCCT-------------------TGG-TTTGCACA----AAATTTCTGAAAAATTC

>Pv01Sk01960/1-522 Pv01Sk01960 undefined product 4833766:4834287 forward

CTCCCGGAATAG----TTTTATTCCTA--AAATTCCACCC-AAG-AA--T-------CT---CCACTG-AA-

TTTGGG-AC----------CAAATGCG-AC-AA-AT--TTCAGG--TC------------------------

-----------------------------------------AAAC-G--GATGAG---TATTCAC-------

---CCA-----TGAAAAAAAT--AA-AAACAGTT-TC--------------------------ACA-CAC--

AAAC--G-AA-ACT------------TCC--T-TTC-------G-G--CC-C-T-GG-C---------A--G

TGACC-AAT-A--A-A-AG--A-TT-TC-TTGCT-AATCTTGT-GGAACGAA----TCT------AGG----

-------------GCTCAAACCTCAG-C----------------CAAAA--CTCAATAGACACA---G----

------------------TGA--C----GAA-TGTCTGG----------T--A-AAAATTTC-AGA-CCA--

--AAATACCC-------AAGGA-GT-----AAGGC---GT-----AGCA------AG-TCCCAGA-CCGATA

GTGAA-------------------------------------------------------------------

------------------------------------------------------------------------

--------------C--AACACC--------------------------GG-----------------TTTT

C----------------------CGAAAACAAAA--C--GTCCTGGC-----TTTTCATCTC----------

--------------CGTATC--TTCT-----------TTTTATGA---------------------------

-------------------------TTC--GTTTAT-GGAC--GTG-GCT-CC-TTGCCT--AGTTGC--AA

AC-----------AACAT-A----CGAAAGT-GTTTGTG-T-------------GAATTT------------

----TTTTCA------------------------------GCTTAAT-A-C-A-GA-CTC-AGA-----AT-

GAAATC--GTAGAAA--CTAGG------------CCAGAAT-------------------------------

-----------T-AGGCC-------------------TTG-GTTTGACA----AAATATCTGAAAAATTC

>Pv01Sk01970/1-541 Pv01Sk01970 undefined product 4834287:4834827 forward

CTCCCAGAATAG-----TTTTTTCCTA--AAATTCCACCC-AAG-AA--T-------CT---CCACTG-AA-

TTTGGG-AC----------CAAACGCG-AC-AA-AT--TTCAGG--TC------------------------

-----------------------------------------AAAC-G--GATGAG---TATTCAC-------

---CCA-----CGAAAAAAA---TC-AAACAGTT-TC--------------------------ACA-CAG--

AAAC--A-AA-CCT------------TCC--T-TTC-------A-G--CC-T-T-GG-C---------A--C

TGACG-AAT-A--A-A-AG--A-TT-TA-TTGTC-AATCTTGT-GGGACGAA----TCT------GAG----

-------------GCTCAAACCTTAG-C----------------CAAAA--CTTAATAGACACA---G----

------------------TGA--C----GAA-TGTCTGG----------T--A-AAAACTTT-AGA-CCA--

--AAATACCC-------AAGGA-GT-----AAGGC---AT-----AGTA------AG-TCGCAGA-CCGAAA

GTGAA-------------------------------------------------------------------

------------------------------------------------------------------------

--------------C--AAAATT--------------------------TG-----------------TTTT

C----------------------CAAAAACAAAA--C--GTCTTGGC-----TTTTCGTCCC----------

--------------CGTATC--TTCT-----------TTTTGTGA---------------------------

-------------------------TTC--GTTTAT-GGAC--GTG-CCT-CC-TTGCCT--GGGTGC--AA

AC-----------AACAT-A----CGAAAGT-GTTTGTG-T-------------GAATTT------------

----TTTTCA------------------------------CTGCAAT-A-C-G-TT-CCC-AGA-----AT-

GAAATT--GCAGAAA--TTAGG------------TCGGAAACT--CACA-A-GTT-TCGG--TAGAT-----

---------GAT-AGCCT-------------------TGG-TTTGCACA----AAATTTCTGAAAAATTC

>Pv01Sk01980/1-533 Pv01Sk01980 undefined product 4834827:4835359 forward

CTCCCAGAATAG-----TTTTTTCCTG--AAATTCCGCCC-AAG-AA--T-------CT---CCACTG-AA-

TTTGGG--------------AACGCA-AC--AA-AT--TTCAGA--TC------------------------

-----------------------------------------AAAT-G--GATGAG---TATTCAC-------

---CCA-----CAAAAAAAA---TC-AAACAGTT-TC--------------------------ACA-CAC--

AAAC--G-AA-CTT------------CCA--T--CC-------A-G--CC-T-T-GG-T---------A--G

TGACG-AAT-A--A-A-AG--A-TT-TA-TTGCC-AATCTTGT-GGGATTGA----TTT------GGG----

-------------GCTCAAACCTCAG-C----------------CAAAG--TTCAATAGACACA---G----

------------------TGA--C----GAA-TGTCTGG----------T--A-AAAATTTC-AGA-CCA--

--AAATACCC-------AAGGA-GT-----AAGGC---GT-----AGTA------AG-TCCCAGA-CCGAGA

GT-AA-------------------------------------------------------------------

------------------------------------------------------------------------

--------------C--AAAACC--------------------------GG-----------------TTTT

C----------------------CGAAAACAAAA--C--GTCCTGTC-----TTTTCTTCCA----------

--------------CGTATC--TTCT-----------TTTTGTGA---------------------------

-------------------------TTC--GTTTAT-GGAC--GTG-CCT-CC-TTGCCT--GGGTGC--AA

AC-----------ATCAT-A----CGAAAGT-GCGTGTG-T-------------GAAATT------------

----TTC--A------------------------------GCGCAAT-A-C-G-GA-CCC-AAA-----AT-

GAAATT--GCAGTAA--TTAGA------------CCGGAATTT--CACA-A-GTT-TCGG--CAGAG-----

---------GAT-AGCCT-------------------TGG-TTTGCACA----AAATTTCTGAAAAATTC

>Pv01Sk01990/1-538 Pv01Sk01990 undefined product 4835362:4835899 forward

--CCAGAAGAGT----TTTTTTTCCTG--AAATTCCACCC-AAG-AA--T-------CT---CCACTA-AA-

TTTGGG-AC----------AAAATGAG-AC-CA-AT--TTTCGG--TC------------------------

-----------------------------------------AAAC-G--AACGAG---TATTCAC-------

---CCA-----AGAAAAAAA---TC-AAACAGTT-TC--------------------------ACA-CAA--

AAAC--G-AA-CCT------------TCC--T-TTC-------A-G--CC-C-T-GC-C---------A--G

TGACG-AAT-A--A-A-AG--A-TT-TA-TTGTC-AATCTTGT-GGGACGAA----TCT------GGG----

-------------GCTCAAACCTCAA-C----------------CAAAA--CTCAATAGACACA---G----

------------------TGA--C----GAA-TGTCTGC----------T--A-AAAATTTC-AGA-CCA--

--AAATACCC-------AAGGA-GT-----AAGGC---CT-----AGTA------AG-TCCCAGA-CCGAGA

GTGAA-------------------------------------------------------------------

------------------------------------------------------------------------

--------------C--AAAACC--------------------------GG-----------------AATT

C----------------------CAAAAACAAAA--C--TTCCTGCC-----TTTTTTTCCC----------

--------------CGTATG--TTCT-----------TTTTCTGA---------------------------

-------------------------TTC--GTTTAT-GGAC--GTG-TCT-CC-TTGCCA--GGGTGC--AA

AT-----------AACAT-A----CGAAAGT-GCTTGTG-T-------------GAA--T------------

---TTTTTCA------------------------------GCGGAAT-A-C-A-GA-CCC-AAA-----AT-

GAAATT--GCATAAA--TTAGG------------TCGGAATTT--CACA-A-GTT-TCGG--TAGAG-----

---------GAT-AGCCT-------------------TGG-TTTGCAAA----AAATTTCTGAAAAGTT-

>Pv01Sk02000/1-540 Pv01Sk02000 undefined product 4835900:4836439 forward

CTCCCGGAATAG-----TTTTTTCCTG--AAATTCCACCT-AAG-AA--T-------CT---CCACTG-AA-

TTTGGG-AC----------AAAACGCG-AG-AA-AT--TTCAGG--TC------------------------

-----------------------------------------AAAC-A--GATGAG---TATTCAC-------

---CCA-----CGAAAAAAA---TT-AAACAGTT-TC--------------------------ACA-CAC--

AAAC--G-AA-CCT------------TCC--T-TTC-------A-G--CC-C-T-AG-C---------A--G

TGACA-AAT-A--A-A-AG--A-TT-TA-TTGCA-AATCTTGT-GGGACGAA----TCT------GGT----

--------------CTGAAACCTCAG-C----------------CAAAA--GTCAATAGACACA---G----

------------------TGA--C----GAA-TGTTTGG----------T--A-AAAATTTC-CAA-CCA--

--AAATACCC-------AAGGA-GT-----AAGGC---GT-----AGCA------AG-TCCCTTA-CCGAGA

GTGAA-------------------------------------------------------------------

------------------------------------------------------------------------

--------------C--AAAACC--------------------------GG-----------------TCTT

C----------------------TGAAAACAAAA--C--GTCCTGGC-----TTTTCTTCCC----------

--------------CGTATC--TTCT-----------TTTTGTGA---------------------------

-------------------------TTC--ATTTAT-AGAT--GTG-CCT-CC-TTTCTT--GGGTGC-AAA

AC-----------AACGT-A----CGAAAGT-GCTTGTG-T-------------GAATTT------------

---TTTTTCA------------------------------ACGCAAT-A-C-G-GA-CCC-AGA-----AT-

GAAATT--GCAGAAA--TTAAA------------TTGGAATTT--CACA-A-GTT-TCAG--TACAC-----

---------GAT-AGTCA-------------------TGG-TTTGCATA----AAATTTTTGAAAAAA--

>Pv01Sk02010/1-536 Pv01Sk02010 undefined product 4836442:4836977 forward

-TCCCAGAACAG-----TTTTTTCCTG--AAATTCAACCC-AAG-AA--T-------CT---CCACTT-AA-

TTTGGG-AC----------AAAACGCG-AC-AA-AT--TTCAGG--TA------------------------

-----------------------------------------AAAC-G--GATGAG---TATTCAC-------

---CCA-----CGAAAAAAA---GC-AGACAACC-TC----------------------------A-CAC--

AAAC--G-AA-CCT------------TCC--T-TTA-------A-G--CC-C-T-CG-C---------A--G

TGACG-AAT-A--A-A-AG--A-TT-TA-TTGCC-AATCTTGT-GGGACGAA----TTT------GGG----

-------------GCTCAAACCTCAG-T----------------CAAAA--CTCAATAGACGCA---G----

------------------TGA--C----GAA-TGTCTGG----------T--A-AAAATTTC-AGA-CCA--

--AAATACCC-------AAGGA-GT-----AAGGC---GT-----AGTA------AC-TCC-AGA-CCAAGA

GTGAA-------------------------------------------------------------------

------------------------------------------------------------------------

--------------C--AAAATC--------------------------GA-----------------TTTT

C----------------------CGAAAACAAAA--C--GTCCTGGT-----TTTTCGTCCC----------

--------------CATATC--TTCT-----------TTTTGTGA---------------------------

-------------------------TTC--GTTTAT-GGAT--GTG-CCT-CC-CTTCCT--AGGTGC--AA

AA-----------AATAT-A----CGAAAGT-GTTTGTG-T-------------GAA-TT------------

---TTTTTCA------------------------------GCGCAAT-A-C-G-GA-CCC-AGA-----AT-

GAAATT--GCAGAAA--TTAAG------------TCGGATTTT--CACA-A-GTT-TTGG--TAGAG-----

---------CAT-AACCT-------------------TGG-TTTGCACA----AAATTTTTGAAAATTT-

>Pv01Sk02020/1-540 Pv01Sk02020 undefined product 4836978:4837517 forward

CTCCCGAAATAT----TTTTTTTCTTG--AAATTTAACCC-AAG-AA--T-------CC---CCACTG-AA-

TTTGGG-AA----------AAAACGCA-AC-AA-AT--TTCAGG--TC------------------------

-----------------------------------------AAAC-G--GATGAG---TATTCAC-------

---TCA-----CGAAAAAAA---TC-AAACAGTT-TC--------------------------ACA-CAC--

AAAT--G-AA-CCT------------TCC--T-TTT-------A-G--CC-C-T-GG-C---------A--G

AGACG-AAT-A--A-A-AG--A-TT-TA-TTGCT-AATCTTGT-GGGACGAA----TCT------GGG----

-------------ACTCAAACCTCAG-C----------------CAAAA--CTCAATAGACGCA---G----

------------------TGA--C----GAA-TGTTTGG----------T--A-AAAATTTG-AGA-CAA--

--AAATACCC-------AAGGA-AT-----AAGGC---GT-----AGTA------AG-TCCCAAA-CCAAGA

GTAAA-------------------------------------------------------------------

------------------------------------------------------------------------

--------------C--AAAACT--------------------------GG-----------------TTTT

C----------------------CGAAAACAAAA--C--ATCCTGCC-----TTTTCTTCCC----------

--------------CGTATC--TTCC-----------TTTTGTGA---------------------------

-------------------------TGT--TTTTAG-GGAC--GTG-CCT-CC-TTGCCT--GGGTGC--AA

AC-----------AACAT-A----CAAAAGT-GCTTGTG-T-------------GATTTT------------

----TTTT-A------------------------------TAGCAAT-A-C-G-TA-CCC-AGA-----AT-

GAAATT--TCAGAAA--TTAGG------------CCGGAATTT--CACA-A-GTT-TCGG--TAGAG-----

---------AAT-AGCCT-------------------TGG-TTTGCACA----AAATTTCTGAAAATTT-

>Pv01Sk02030/1-542 Pv01Sk02030 undefined product 4837518:4838059 forward

CTCCCAGAATAG----TTTTTTTCCTG--AAATTCCACCC-AAA-AA--T-------CT---ACACTG-AA-

TTTGGG-AC----------AAAACCCG-AC-AA-AT--TTCCGG--TC------------------------

-----------------------------------------AAAC-G--GATGAG---TATTCAC-------

---CCA-----CGAAAAATA---TC-AAACATTT-TC--------------------------ACA-CAC--

AAAC--G-AA-CTT------------TCC--T-TTC-------A-G--CC-C-T-GG-C---------A--A

TTACG-AAT-A--A-A-AG--A-TT-TA-TTGCC-AATCTTGT-AGGACGAA----TCT------GGG----

-------------GCTCAAACCTCAG-C----------------CAAAA--CTCAATAGACACA---A----

------------------TGA--T----GAA-TGTCTGG----------T--A-AAAATTAC-AGA-CCA--

--AAATACCC-------AAGGA-GT-----AAGGC---GT-----AGTA------AG-TCCGAGA-CCGAGA

GTGAA-------------------------------------------------------------------

------------------------------------------------------------------------

--------------C--AAAACC--------------------------GG-----------------TTTT

C----------------------CAAAAACAAAA--C--GTCCTGTC-----TTTTCTTTCC----------

--------------CGTATC--TTCT-----------TTTTGTGA---------------------------

-------------------------TTC--GTTTAT-GGAC--GGG-CCT-CC-TTGCCT--GGGTGC--AA

AC-----------AACAT-A----CGAAAGT-GCTTGTG-T-------------GAA-TT------------

---TTTTTCA------------------------------GCGCAAT-A-T-G-GA-CCT-AGA-----AT-

GAAATT--GCAGAAA--TTAGG------------CCAAAAATT--CACA-A-GTT-TTGG--TAGAG-----

---------GAT-AACCA-------------------TGG-TTGGCACA----AAATTTCTGAAAAATTC

>Pv01Sk02040/1-545 Pv01Sk02040 undefined product 4838059:4838603 forward

CTCCCGGAATAG----TTTTTTTCCTG--AAATTCCACTC-AAG-AC--T-------AT---CCACTG-AA-

TTTTGG-AT----------AAAACACG-AA-GA-AT--TTCAGG--TC------------------------

-----------------------------------------AAAC-G--GATGAG---TATTCAC-------

---CCA-----TGAAAAAAAA--TC-AAACAGTT-TC--------------------------ACA-CAC--

AAAT--G-AA-CCT------------TCC--T-TTC-------A-G--CT-C-T-GG-C---------A--A

TGACG-AAT-A--A-A-AG--A-TT-TA-TTGTC-AATCTTGT-GGGACGAA----TCT------AGG----

-------------GCTCAAACCTCAG-T----------------CAAAC--CTCAATAGACGCA---G----

------------------TGA--C----GAA-TGTCCGG----------G--A-AAAATTTC-AGA-CTA--

--AAATACCC-------AAGGA-GT-----AAGGC---GT-----AGTA------AG-TCCCGGA-CCGAGA

GTGAA-------------------------------------------------------------------

------------------------------------------------------------------------

--------------C--AAAACC--------------------------AG-----------------TTTT

C----------------------CGGAAACAAAA--T--GTCCTGGT-----TTTTCTTCCC----------

--------------CGTATC--TTCT-----------TTTTGTGA---------------------------

-------------------------TTC--GTTTAT-GGAC--GTG-CCT-CC-TTGCCT--GGGTGC--AA

AA-----------ACCAT-A----AGAAAAT-GCTTGTG-T-------------GAATTT------------

----TTTTCA------------------------------GCGCAAT-A-C-A-GA-CCC-ATA-----AT-

GAAATT--GCAGAAA--TTAGG------------CCGGAATTT--CACA-A-GTT-TCGG--TAGAATA---

---------TAT-AGCCT-------------------TGG-TTTGCACA----AAATTTCTGAAAAATTC

>Pv01Sk02050/1-538 Pv01Sk02050 undefined product 4838603:4839140 forward

CTCCCGGAATAG-----TTTTTTCCTG--AAATTCCACCC-AAG-AA--T-------CT---CCATTG-AA-

TTTGGT-AC----------AAACCGCG-AC-AA-AT--TTCAGG--TC------------------------

-----------------------------------------AAAT-G--GAGGAG---TATTCAT-------

---CCA-----CGAAAAAAA---TC-AAGTAGTT-TC--------------------------ACA-C----

AAAC--G-AA-CTT------------TCC--T-TTC-------A-G--CC-C-T-GG-C---------T--G

TGACG-AAT-A--A-A-AG--A-TT-TA-TTGCC-AATCTTGG-TGGACGAA----TCT------AGG----

-------------GCTCAAACCTCAG-T----------------CAAAA--CTCAATAGACACA---G----

------------------TGA--C----GAA-TGTCTAG----------T--A-AAAAGTTC-AGA-CCA--

--AAATACCC-------AAGGA-GT-----AAGGC---GT-----AGTA------AG-TCCCAAA-CCGAGA

GTGAA-------------------------------------------------------------------

------------------------------------------------------------------------

--------------C--AAAACC--------------------------GA-----------------TTTT

C----------------------CGGAAACAAAA--C--GTCTTGG------CTTTTCTTCC----------

--------------TGTAGC--TTCT-----------TTTTGTGA---------------------------

-------------------------TTC--GTTTAT-GGAC--GTG-CCT-CC-TTGCCT--GGTTGC--AA

AA-----------ACCAT-A----TGAAAGT-GCTTGTG-T-------------GAATTT------------

----TTTTCA------------------------------GCGCAAT-A-C-G-GA-CCC-AAA-----AT-

GAAATT--GCAGAAA--ATAGG------------CCTATATTT--CACA-A-GTT-TCGG--TAGAG-----

---------GAT-AGCCT-------------------TGG-TTTACACA----AAATTTCTGAAAAATTC

>Pv01Sk02060/1-534 Pv01Sk02060 undefined product 4839140:4839673 forward

CTCCCGGAATAG-TTTTTTTTTTCCTG--AAATTCCTCCC-AAG-AA--T-------CT---CCACTG-AA-

TTTGGG-AC----------AAAACGCG-AC-AA-AT--TCCAGG--TC------------------------

-----------------------------------------AAAC-G--GATGAG---TATTCAC-------

---CCA-----CGAAAAAAA---TC-AAATCGTT-TC--------------------------ACA-TAC--

AATC--G-AA-CCT------------TGT--T-TTC-------A-G--CC-C-T-GG-C---------A--G

TGACG-AAT-A--C-A-AG--A-TT-TA-TTGCC-AATTTTGT-GGGACTTA----TCT------GAG----

-------------GCTCAAACCTCAG-C----------------CAAAA--CTCAATAGACGCA---G----

------------------TGA--T----GAA-TGTCTGG----------T--A-AAAATTTC-ACA-CAA--

--AAATACCC-------AAGGA-AT-----AAGGC---GT-----AGTA------AG-TCCCAGA-CCGAGA

GTAAA-------------------------------------------------------------------

------------------------------------------------------------------------

--------------C--AAAACC--------------------------GG-----------------TTTT

C----------------------CGAAAACAAAA--C--GTCCTGCG-----TTTTCTTCCT----------

--------------CGTATC--TTCC-----------TTTTGTGA---------------------------

-------------------------TTC--GTTTAT-GGAC--ATG-CCT-CC-TTGCCT--GGGTGC--AA

AC-----------AACAT-A----CAAAAGT-GCTTGTG-T-------------GATTTT------------

----TTTT-A------------------------------GTGCAAT-A-C-G-TA-CCC-AGA-----AT-

GAAATT--TCAGAAA--TTAGG------------CTGGAATTT--CACA-A-GTT-TCGG--TAGAG-----

---------AAT-ACCCT-------------------TGG-TTTGCACA----AAATTTA----------

>Pv01Sk02070/1-541 Pv01Sk02070 undefined product 4839674:4840214 forward

-TCTCGAAATAG----TTTTTTTCCTG--AAATTCCACCC-AAG-AA--T-------CT---CCACTG-AA-

TTTTGG-AC----------AAAACGAG-AC-AA-AT--TTCAGG--TC------------------------

-----------------------------------------AAAC-G--GATGAA---TATTCAC-------

---CCA-----CGAAAAAA----AC-AAACAGTT-TC--------------------------ACT-CAC--

AAAC--G-AA-CCT------------TCC--T-TTC-------A-G--CT-T-T-GG-C---------A--G

TGACG-AAT-A--A-A-AT--A-TT-TA-TTGCC-TATCTTTT-GGGACGAA----TCT------GGG----

-------------GCTCAAACCTCAG-C----------------CAAAA--TTCAATAGACGCA---G----

------------------TGA--C----GAA-AGTCTGG----------T--A-AAAATTTC-AAA-CCA--

--AAATACCC-------AAGGA-TT-----AAGGC---GT-----AGTA------AG-TCCGAGA-CCGAGA

GTGAA-------------------------------------------------------------------

------------------------------------------------------------------------

--------------C--AAAACC--------------------------GG-----------------TTTT

C----------------------CGAAAACAAAA--C--GTTCTGGC-----TTTTCATCCC----------

--------------CGTATC--TTCT-----------TTTTGTGA---------------------------

-------------------------TTC--GTTTAT-GGAC--GTG-CCT-CC-TTGCCT--GGGTGC--AA

AC-----------ACCTT-A----CGAAAGT-GCTTGTG-T-------------GAA-TT------------

---TTTTTCA------------------------------GCGATAT-A-C-A-GA-CCC-ATA-----AT-

GAAATT--ACATAAA--TTAGG------------CCGGAATTT--CACA-A-GTT-TCAG--TAGAGG----

---------AAT-AGCCT-------------------TGG-TTTGCACA----AAATTTCTGAAAAATTC

>Pv02Lk00490/1-549 Pv02Lk00490 undefined product 21573742:21574290 forward

-----AAAATAA-----TTTCTTATTG--AAATCTGATGG-AAG-AA--T-------CC---CAATAG-AA-

TTTGGG-AC----------GAAACGCG-AC-AA-GT--TTCAGC--TA------------------------

-----------------------------------------AAAC-G--GACGAG---TATTCAG-------

---TT------AGAAAATTA---TC-AAACAGTT-TC--------------------------ATA-CAG--

AAAC--G-AA-CCT------------TCT--T-TTC-------A-G--TC-G-T-GG-C---------A--A

TGATG-AAT-A--AAA-AA----TT-CA-TTACA-AATCTTGT-GGAACGAA----TTT------GGG----

-------------GCTCAAATCTC-G-C----------------C-ACA-TCTCAATACACACA---A----

------------------TAA--G----GAA-TGTCTGG----------T---AAAAATTTC-AAATCTA--

---AACACCC-------AAGGA-AT-----AAGGC---GT-----AATA------AG-TCCCAGA-CCAAGA

CCAAA-------------------------------------------------------------------

------------------------------------------------------------------------

--------------C--AAAACT--------------------------GCAAAA-CCTG-------TTTTT

C----------------------CGAAAAAAAAA--C--GTCCTGGT-----TTTTCTCCCT----------

--------------TATAAC---TTT-----------TTCCA-GT---------------------------

-------------------------TTT--CTTTTT-GGAA--ATG-CCT-CC-TTGACT--GGATAC--AA

AC-----------GACAT-A----TGAAACC-ACTTATA-TAAGTGTTTTCG--GAAC--------------

----TTTTCA------------------------------GCGTAGT-A-T-G-AA-CTC-ATA-----AT-

TAAATT--ATAGAAA--CTAGG------------GCAAAATTT--CTCA-A-ATC--TGG-AATGAT-----

---------GAG-CGCCT-------------------TAG-AGTGCAAG----AAGTTTCTGGAACATT-

>Pv02Lk00500/1-547 Pv02Lk00500 undefined product 21574292:21574838 forward

-TCCAAAAATAA-----TTTCTTGCTG--AAATCTTATGG-A---------------------AACAG-AA-

TTTAGA-AC----------GAAATGCG-AC-AA-GT--TTCAGC--TC------------------------

-----------------------------------------AAAT-G--GACGAG---TATTCAG-------

---TT------AGAAAATGA---TC-AAACAGTT-TT--------------------------ACA-CAG--

AAAT--G-AA-CCT------------TCC--T-TTC-------A-G--TC-G-T-GG-C---------A--A

TGATG-AAT-A--AAA-AA----TT-CA-TTACA-AATCTTGT-GGAACGAA----TTT------GAG----

-------------GCTCAAATCTTAG-C----------------C-ACA-TCTTAATACACACA---A----

------------------TAA--G----GAA-TGTCCGA----------T---AAAAATTTC-AAATCTA--

---AACACCC-------AAGGA-AT-----AAGGC---GT-----AATA------AG-TCCCAGA-CGGAGA

GTAAA-------------------------------------------------------------------

------------------------------------------------------------------------

--------------C--AAAACT--------------------------GGAAAG-CTTG-------TTTTT

C----------------------CGAAAACAGAA--T--GTCCTGGC-----TTTTCTTCCT----------

--------------TATATC---TTT-----------TTCCGTGT---------------------------

-------------------------TTC--CTTTTT-GGAC--GTG-CCT-CC-TTAACT--GGATAC--AA

AC-----------GACAT-A----TGAAACT-ACTTGTG-AAAGTGTTTTCG--GAAC--------------

----TTTTTA------------------------------GTGTAAT-A-T-G-AA-CTC-AGA-----AT-

AAAATT--GCAGAAA--CTAGG------------GCAAAATTT--CACA-A-ATC--TGG-AAAGCG-----

---------GAG-CGCCT-------------------TGG-AGTGCAAG----AAGTTTCTGGAACATT-

>Pv02Lk00510/1-552 Pv02Lk00510 undefined product 22666113:22666664 forward

--CCAAAAACAA-----ATTATTGTTG--TAATCTTATGG-AAG-AA--T-------CC---CAACAA-AA-

TTTGGG-AC----------GAAACGCG-AC-AA-AT--TTCAGC--TC------------------------

-----------------------------------------AAAC-G--GACAAG---TATTTAG-------

---TT------AGACAATGA---TC-AAACAGTT-TC--------------------------AAA-TAG--

AAAC--G-AA-CAT------------TTC--T-TTC-------A-G--TC-G-T-GG-C---------A--A

TGATA-AAT-A--AAA-AA----TT--A-TTGCA-AATCTTGT-GTAATGAA----TTT------GAG----

-------------GCTCAAATCTCAG-C----------------G-ACA-ACTCAATACACAAA---A----

------------------TAA--G----AAA-AGTCTGG----------T---AAAAATTTC-AAATCAA--

---AACACCC-------AAGGA-AT-----AAGGC---GT-----AATA------AG-TCCACGA-CCGAGA

GCAAA-------------------------------------------------------------------

------------------------------------------------------------------------

--------------T--ACAACT--------------------------AGAAAA-CTTGT------TTTTC

-----------------------CAA-AATAGAA--C--GACTTGAC-----TTTTCTTCCT----------

--------------TATATC---TTT-----------TTCTACGT---------------------------

-------------------------TTG--TGTTTT-GGAC--GTG-CCT-CC-TTGACT--GGATAC--AA

AC-----------AACAT-A----TGAAAGT-GCTTGTG-TAAGTGTTTTCA--AAAC--------------

----TTTTCA------------------------------GCGTAAT-A-T-G-AA-CTT-AAA-----AT-

AAAATT--CCAAAAC--TTAGG------------ACAAAATTT--CGAA-A-ATC--TGG-AAAGTG-----

---------GAG-CGCCT-------------------TGG-ACTGCAAG----AAGTTTCTGGAACATT-

>Pv02Lk00520/1-548 Pv02Lk00520 undefined product 22666670:22667217 forward

-----AAAAGAA-----AATCTTGTTG--TAATCTTATGG-AAG-AA--T-------CC---CGACAG-AA-

TTTGTG-AC----------TAAACGCG-AC-AA-AT--TGCAGC--TC------------------------

-----------------------------------------AAAC-G--AACGAG---TATTTAG-------

---TT------AGACAATGA---AC-AAACAGTT-TC--------------------------AAA-CAG--

AAAC--G-AA-CAT------------TCC--T-TTC-------A-G--TC-G-T-GG-C---------A--A

TAATA-AAT-A--AAA-AA----TT--A-TTGCA-AATCTTGT-GTAACGAA----TTT------GAG----

-------------GCTCCAATCTGAG-C----------------C-ATA-ACTCAATACACGGA---A----

------------------TAA--G----AAA-AGTCTGG----------T---AAAAATTTC-AAATCAA--

---AACACCC-------AAGGA-AT-----AAGGC---GT-----AATA------GG-TCCGCGA-CCGAGA

CCAAA-------------------------------------------------------------------

------------------------------------------------------------------------

--------------T--AAAACT--------------------------AGAAAA-CTTGT------TTTTC

-----------------------CAA-AATAGAA--C--GTCTTGAC-----TTTCCTTCCT----------

--------------TATATC---ATT-----------TTCTACGT---------------------------

-------------------------TTG--TGTTTT-GGAA--GTG-CCT-CC-TTGACT--GGATAC--AA

AC-----------AACAT-A----TGAAAGT-GCTTGTG-TAAGTGTTTTCA--TAAC--------------

----TTTTCA------------------------------GTGTAAT-A-G-A-AA-CTT-AGA-----AT-

AAAATT--CCAAAAC--TTAAG------------GCAAAATTT--CGAA-A-ATC--TAG-AAAGTG-----

---------GAG-CGCCT-------------------TGG-ACTGCAAG----AAGTTTTTGGAACAT--

>Pv02Lk00530/1-553 Pv02Lk00530 undefined product 22667220:22667772 forward

-TCCTAAAAGAA-----ATTCTTCTTG--AAATCTTATGG-AAG-AA--T-------CC---CAACAG-AA-

TTTGGG-AC----------GAAACGCA-AC-AA-AT--TTCAAC--TC------------------------

-----------------------------------------AAAC-A--AACGAG---TATTTAG-------

---TT------AGACAATGA---TC-AAGCAGTT-TC--------------------------AAA-CAG--

AAAC--G-AA-CAT------------TCC--T-TTC-------A-G--TC-G-T-GG-C---------A--A

TGATA-AAT-A--AAA-AA----TT--A-TTGCA-AATCTTGT-GTAACTAA----TTT------GAG----

-------------GCTCAAATCTTAG-C----------------C-ACG-ACTCAATACGCGGA---A----

------------------TAA--G----ATA-AGTTTGA----------T---AAAAATTTT-AAATCAA--

---AACACCC-------AAGGA-AT-----AAGGC---AT-----AATA------AG-TCCGCGA-CCGAGG

GCAAA-------------------------------------------------------------------

------------------------------------------------------------------------

--------------T--AAAACT--------------------------AGAAAA-CTTGT------TTTTC

-----------------------CAA-AATAGAA--C--GTCTTGAC-----TTTTCTTCCT----------

--------------TATATC---TTT-----------TTCTGTTT---------------------------

-------------------------TTG--TGTTTT-GGAC--GTG-CCT-CC-TTGACT--GGATAC--AA

AC-----------AATAT-A----TGAAGAT-GCTTGTG-TAAGTGTTTTCA--GAAC--------------

----TTTTCA------------------------------GCGTAAT-A-T-G-AA-CTT-AGA-----AT-

AAAATT--CACAAAC--TTAGG------------GCAAAATTT--CTCA-T-ATT--TCG-AAAGAG-----

---------GAG-CGCCT-------------------TGA-AGTGCAAG----AAGTTTCCGGAACATT-

>Pv02Lk00540/1-535 Pv02Lk00540 undefined product 22667782:22668316 forward

---------GAA-----ATTGTTGTTG--TAATCTTATGG-AAG-AA--T-------CC---CAACAG-AA-

TTTGGG-AC----------GGAACGCG-AC-AA-AT--TTCAGC--TC------------------------

-----------------------------------------AAAC-G--GATGAG---TATTTAG-------

---TT------AGACAATGA---TC-AAAC--------------------------------------AG--

AAAC--G-AA-CAT------------TCC--T-TTC-------A-G--TC-G-T-GA-C---------A--A

TGATA-AAT-A--AAA-AA----TT--A-TTACA-AATCTTGT-GTAACGAA----TTT------GAG----

-------------GCTAAAATCTCAG-C----------------C-ACA-ACTCAATACACGGA---A----

------------------TAA--G----AAA-AGTCTAG----------T---AAAAATTTC-AAATCAA--

---AACACCC-------AAGGA-AT-----AAGGA---GT-----AATA------AG-TCCGCGA-CCGAGG

GCAAA-------------------------------------------------------------------

------------------------------------------------------------------------

--------------T--AAAACT--------------------------AGAAAA-CTTGT------TTTTC

-----------------------CAA-AATAGAA--T--GTCTTGAC-----TTTTCTTCCT----------

--------------TATATA---TTT-----------TTCTGTGT---------------------------

-------------------------TTG--TGTTTT-GGAC--GTG-CCA-CC-TTGACT--GGATAC--AA

AC-----------AACAT-A----TGAAAGT-GCTTGTG-TAAGTGTTTTCA--AAAC--------------

----TTTTCA------------------------------GCGTAAT-A-T-G-AA-CTT-AGA-----AT-

AAAATT--CCAAAAC--TTAGG------------CCAAAATTT--CGAA-A-ATC--TGG-AAAGTA-----

---------GAG-TGCCT-------------------TGG-ACTGCAAG----AATTTTCTGGAACATT-

>Pv02Lk00550/1-548 Pv02Lk00550 undefined product 22668319:22668866 forward

--CCAAAAAGAA-----ATTCTTCTTG--AAATCTTATGG-AAG-AA--T-------CC---CAACAG-AA-

TTTGGG-AC----------GAAACGCG-AC-AG-AT--TTCCGC--TC------------------------

-----------------------------------------AAAC-G--AACGAG---TATTTAG-------

---TT------AGACAATGA---TC-AAGCAGTT-TC--------------------------AAA-CAG--

AAAC--G-AA-CAT------------TCC--T-TTC-------A-G--TC-G-T-CG-C---------A--A

TG--T-AAA-T--AAA-AA----AT--A-TTTCA-AATCTTGT-GTAACGAA----TTT------GAG----

-------------GCTCAAATCTCAC-C----------------C-ACA-ACTCAATACACGAA---A----

------------------TAT--G----AAA-AGTCTGG--------------AAAAATTTC-AAATCAA--

---AACACCC-------AAGGA-AT-----AAGGC---GT-----AATA------AG-TCTGCGA-CCGAGA

CCAAA-------------------------------------------------------------------

------------------------------------------------------------------------

--------------T--AAAACT--------------------------AGAAAA-CTTGT------TTTTC

-----------------------CAA-AATAGAA--C--ATCTTGAC-----TTTTCTTCCT----------

--------------TATATC---ATT-----------TTCTACGT---------------------------

-------------------------TTG--TGTTTT-GGAA--GTG-CCT-CC-TTGACT--GGATAC--AA

AC-----------AACAT-A----TGAAAGT-GCTTGTG-GAAGTGTTTTCA--TAAC--------------

----TTTTCA------------------------------GTGTAAT-A-G-A-AA-CTT-AGA-----AT-

AAAATT--CCAAAAC--TTAAG------------GCAAAATTT--CGAA-A-ATC--TGG-AAAGTG-----

---------GAG-CGCCT-------------------TGG-ACTGCAAG----AAGTTTTTGGAACAT--

>Pv02Lk00560/1-553 Pv02Lk00560 undefined product 22676740:22677292 forward

-TCCAAAAATAA-----ATTCTTGTTG--TAATCTTATGG-AAG-AA--T-------CC---CAACAA-AA-

TTTGGG-AC----------GAAACGCG-AC-AA-AT--TGCAGC--TC------------------------

-----------------------------------------AAAC-G--GACGAG---TATTTAG-------

---TT------AGACAATGA---TC-AAACAATT-TC--------------------------AAA-CAG--

AAAT--G-AA-CAT------------TCC--T-TTT-------A-G--TC-G-T-GG-C---------A--A

TGATA-AAT-A--AAA-AA----TA--A-TTGCA-AAACTTGC-GTAACGAA----TTT------TAG----

-------------GCTGAAATCTCAG-G----------------C-ACA-ACTCAGTACACGGA---A----

------------------TAA--G----AAA-AGTCTGG----------T---AAAAGTTTC-AAATCAA--

---AACACCT-------ATGGA-AT-----AAGGC---GT-----AATA------AG-TCTACGA-CCGAGA

GTAAA-------------------------------------------------------------------

------------------------------------------------------------------------

--------------T--AAAACT--------------------------GGAAAA-CTTAT------TTTTC

-----------------------CAA-AATAGAA--C--GTCTTGAC-----TTTTCTTCCT----------

--------------TATATT---TTT-----------TTCTTTGT---------------------------

-------------------------TTG--TGTTTT-GGAC--GTG-CCT-CC-TTGACT--GGATAC--AA

AC-----------AACAT-A----TGAAAGT-GCTTGTG-TAAGTGTTTTCA--GAAC--------------

----TTTTCA------------------------------GCGTAAT-A-T-G-AA-CTT-AGA-----AT-

AAAATT--CCAAAAC--TTAGG------------GCAAAATTT--TGAA-A-ATC--TGG-AAACTG-----

---------GAG-CGCCT-------------------TGG-ACTGCAAG----AAGTTTCTGGAACATT-

>Pv02Lk00570/1-522 Pv02Lk00570 undefined product 23736928:23737449 forward

-TCCAAAAACAA-----TTTTTTGTTG--AAATATTATGG-AAG-AA--T-------TC---CAAAAG-AA-

TATGGG-AC----------GAAACGCG-AC-AA-AT--TTCAGC--TC------------------------

-----------------------------------------AAAC-G--GACAAG---TATTCAA-------

---TT------AGAAAATGA---TC-AAACAGTT-TC--------------------------ACA-CAG--

AAAC--G-GA-CAT------------TCC--T-TTC-------A-G--TC-G-T-GG-C---------A--A

TGATG-AAT-A--AAA-AA----TT-CA-TTGCA-AATCTTGT-GGAACAAA----TTT------GAG----

-------------GCTCAAATCTTAA-C----------------C-AAA-ACTCAATACACACA---A----

------------------TAA--G----GAA-TATCTGG----------T---AAAAATTTC-AAATCAA--

---AACACCC-------AAGGA-AT-----AAGGC---GT-----AATA------AT-TCCTAAA-ACAAGA

GCAAA-------------------------------------------------------------------

------------------------------------------------------------------------

--------------C--AAAACT--------------------------GGAAAA-CTTG-------TTTTT

C----------------------CGAGAACACAA--C--GTCCTGGG-----TTTTCTTCCT----------

--------------TATATT---TTCT----------TTTTGTGT---------------------------

-------------------------TTT--CTTTTT-GGGT--GTG-CCA-CC-TTGACT--AGATAC--AA

AT-----------GACAT-A----TGAAAGT-ACTTTTG-TAAGTGTTTTCG--GAAC--------------

----TTATTA------------------------------GTGCAAT-A-C-G-AA-CTC-ATA-----AT-

AAAATT--GCAAACA--CTAGA------------AAAAAATTC--CGCA-A-ATC--TGG-AAAAGG-----

---------G------------------------------------------------------------

>Pv02Lk00580/1-525 Pv02Lk00580 undefined product 23737486:23738010 forward

--CCAAAATCAA-----TTTCTTGTTG--CAATCTTATAG-AAG-AA--T-------CC---CAACAG-AA-

TTTGGA-AC----------AAAACGCG-AC-AA-AT--TTCAGC--TC------------------------

-----------------------------------------AAAA-G--GACGAG---TATTCAG-------

---TT------AGAAAATGA---TC-AAACAATT-TC--------------------------ACA-CAA--

AAAC--A-GA-CCT------------TCC--T-TTC-------A-G--TC-A-T-GG-C---------A--A

TAATG-ATT-A--AAA-AA----TT-CA-TTTCA-AATC---------------------------------

----------------------TCAG-C----------------C--CA-ACTCAATAAACACA---A----

------------------TAA--G----GAA-TTTTTGG----------T---AAAAATTTC-AAATCAA--

---AACACCG-------AAGGA-AA-----AAGGC---GT-----AATA------AT-TCCCAGA-ACGAGA

GAAAA-------------------------------------------------------------------

------------------------------------------------------------------------

--------------C--AAAACT--------------------------GGAAAA-CTTG-------TTTTT

T----------------------CGAAAACAAAC--C--GTCCTGAC-----TTTACTTCCT----------

--------------TATATC---TTT-----------TTCTGTGT---------------------------

-------------------------TTT--CTTTTT-GGGG--TTG-CCT-AC-TTGACT--GGATAC--AA

AT-----------GACAT-A----TGAAAGT-ACTTGTG-CAACTGTTTTTG--GAAC--------------

----TTATCA------------------------------GCGTAAT-A-C-A-AA-CTC-AGA-----AT-

AAAATT--GCAAAAA--CTAGG------------GCAAAATTT--TGCA-A-GTT--TGG-AAAGTG-----

---------GAG-CTCCT-------------------TGC-AGTCCAAG----AAGTTTCAAGAACAT--

>Pv02Lk00590/1-515 Pv02Lk00590 undefined product 23739847:23740360 forward

-------------------------------------------------T-------TC---CAATAG-AA-

TTTGGG-AT----------GAAATGCA-AC-AA-AT--TTCAAC--TC------------------------

-----------------------------------------AAAC-A--GACGAG---TATTTAG-------

---TT------AGAAAATGA---TC-AAACAGTT-TC--------------------------ACA-CAG--

AAAC--A-GA-CCT------------TCC--T-TTC-------A-G--TC-G-T-AG-C---------A--A

TGATG-AAT-A--AAA-AA----TT-CA-TTGCA-AATCTTGT-GGAACAAA----TTT------GAG----

-------------GCTCAGATCTCAG-C----------------C--CA-ACCCAATACACAGA---A----

------------------TAA--G----GAA-TGTCTGG----------T---AAAAATTTC-AAATCAA--

---AACAC-C-------AAGGA-AT-----AAGGT---GT-----AATA------TT-TCCCAGA-CCAAGA

GCAAA-------------------------------------------------------------------

------------------------------------------------------------------------

--------------C--AAAACT--------------------------GGAAAA-CTTGT------TTTTT

C----------------------CGAACACAAAA--T--GTCCTGGC-----TTTTCTTCCT----------

--------------CATATA---TTT-----------TTCTATTT---------------------------

-------------------------TTT--CATTTT-TTGC--GTG-CCT-CC-TTGACT--AGATAA--AA

AC-----------GACAT-A----TGAAAGT-ACTTGTG-TACGTGTTTTCA--GAAG--------------

----TTATCG------------------------------GGGTAAT-A-T-G-----TC-AGA-----AT-

AAAATT--GCAAAAA--TTAGA------------GCAAAATTC--TGCA-A-GTG--TGG-AAAGTG-----

---------GAG-CTCCT-------------------TGA-AGTCTAAG----AAGTTTCCGGAACATT-

>Pv02Lk00600/1-553 Pv02Lk00600 undefined product 23742269:23742821 forward

--CCAAAAGCAA-----TTTCTTGTTG--AAATCTTACGA-AAG-AT--T-------TC---CAATAG-AA-

TTTGGG-AC----------GAAACGCG-AC-AA-AT--TTCAGC--TC------------------------

-----------------------------------------AAAC-A--GACGAG---TATTCAG-------

---CT------AGAAAATGA---TC-AAATAGTT-TC--------------------------ACA-GAG--

AAAC--A-AA-CCT------------TCC--T-TTC-------C-G--CC-G-T-AG-C---------A--A

TGATT-TAT-A--AAA-AA----TT-CA-TTGCA-AATCTTGT-GGAACGAA----TTT------GAG----

-------------GCTCAGATCTCAG-C----------------C--CA-ACCTAATACACACA---A----

------------------TAA--G----GAA-TTTCTGG----------T---AAAAATTTC-AAATCAA--

---AACAC-C-------AAGCA-AT-----AAGGC---GT-----AATA------TT-TCCCAGA-TCGAGA

GCAAA-------------------------------------------------------------------

------------------------------------------------------------------------

--------------T--AAAACT--------------------------GGAAAA-CTTGT------TTTTT

C----------------------CGAACACAAAA--T--GTCCTGGC-----TTTTCTTCCT----------

--------------CATATC---TTT-----------TTCTGTTT---------------------------

-------------------------TTT--CATTTTGTTGT--GTG-CCT-CC-TTGACT--AGATAA--AA

AT-----------GACAT-A----TGAAAAT-ACTTGTG-TAAGTGTTTTCG--GAAG--------------

----TTATCG------------------------------GGGTAAT-A-T-G-AG--TG-AGA-----AT-

AAAATT--GCAAAAA--TTAGA------------GCAAAATTC--CGCA-A-GTC--TGG-AAAGTG-----

---------GGG-CTCCT-------------------TGA-TGTCCAAG----AAGTTTCCGGAACATT-

>Pv02Lk00630/1-552 Pv02Lk00630 undefined product 23743638:23744189 forward

-TCCAAAAACAA-----TTTCTTGTTG--AAATCTTACGG-AAG-AT--T-------CC---CAACAA-AA-

GTTGGA-AC----------GAAACGTG-AC-AA-AT--TTCAGC--TC------------------------

-----------------------------------------AAAC-A--GACGAG---TATTCGG-------

---TT------AGAAAATGA---TC-AAACAGTT-TC--------------------------ACA-CAG--

AAAC--A-AA-CCT------------TCC--T-TTC-------A-G--TT-G-T-AG-C---------A--A

TGATG-AAT-A--AAA-AA----TT-CA-TTGCA-AATCTTGT-GGAACGAA----ATT------GAG----

-------------GCTCAGATCTCAG-C----------------C--CA-ACCCAATACACACA---A----

------------------TAA--G----GAA-TGTCCGG----------T---AAAAATTTC-AAATCAG--

---AACAC-C-------AAGGA-AA-----AAGGC---GT-----AA-A------AT-TCCCAGA-CTAAGA

GCAAA-------------------------------------------------------------------

------------------------------------------------------------------------

--------------A--AAAACT--------------------------GGAAAA-CTTGT------TTTTT

C----------------------CGAGCACATAA--T--GTCCTGGC-----TTTTCTTCCT----------

--------------CATATA---TTT-----------TTCTGTGT---------------------------

-------------------------TTT--CATTTT-TTGC--ATG-CCT-TC-TTGACT--GGATAA--AA

AT-----------GACAT-A----TGAAAGT-ACTTGTG-TAAGTGTTTTCA--GAAA--------------

----TTATCG------------------------------GGGTAAT-A-A-G-AA-CTA-AGA-----AT-

AAAATT--GCAAAAT--TTAGA------------GCAAAATTC--CGCA-A-GTC--TGG-AAAGTG-----

---------GAG-CTCCT-------------------TGA-AGTCCAAG----AAGTTTCCAGAACAT--

>Pv02Lk00680/1-549 Pv02Lk00680 undefined product 23745114:23745662 forward

------AAAGAA-----TTTCTTTTTG--AAATCTTATTG-AAG-AA--T-------CC---CAAAAT-AT-

TTTGGA-AC----------AAAGTGCG-AT-AA-GT--TTCAGT--TT------------------------

-----------------------------------------AAAC-G--TACGAG---TATTGAG-------

---TT------CGAAAAAGA---TC-AAACAGTT-TC--------------------------ACA-CAA--

AAAC--G-AA-CCT------------TCC--T-TTT-------G-A--TC-G-T-GG-C---------A--A

TGATC-AAT-A--AAA-AA----TT-TA-TTGAA-ATTCTTGT-GGAACGAA----TTT------CAG----

-------------GCTCGAATCTCAG-C----------------C-ACA-ACTCAATACACACA---A----

------------------TAA--G----GAA-TGTCTGG----------T---AAAAAATTT-AAGTCGA--

---AACACCC-------AAGGA-AT-----AAGAC---GT-----AATA------AT-TCCCAGA-CCGAGA

GAAAC-------------------------------------------------------------------

------------------------------------------------------------------------

--------------C---AAACT--------------------------AGAAAA-CTTGT------TTTTT

C------------------------GAAATAGAA--T--GTCCTGGC-----TTTTCTTCCT----------

--------------GATAAC--TTTT-----------TACTGTGT---------------------------

-------------------------TTT--CCTTTT-GGGC--GCC-CCG-CC-TTGACT--AGATAC--AA

AC-----------GACAT-A----AGAAAGT-ACACATG-TAAGTGTTTTCG--GAAC--------------

----TTATCA------------------------------GTGTAAT-A-T-G-AA-CTC-AAA-----AT-

AAAATT--GCAAAAA--CTAGG------------GCAAAATTT--CACA-A-ATC--TGG-AAAGGG-----

---------GAG-CTCCT-------------------TGG-AATCCAAG----AAGTTTCTGGAACATT-

>Pv02Lk00690/1-544 Pv02Lk00690 undefined product 23745667:23746210 forward

---CAAAAACAA-----TTTCTTGGTG--AAATCTTATGG-AAG-AA--T-------CC---CAAAAT-AA-

TTTGGG-AT----------GAAACGCG-AC-AA-AT--TTCAA---TC------------------------

-----------------------------------------AAAC-G--GATGAG---TATTCAA-------

---TT------ATAAAATGA---TC-AAACA-TT-TT--------------------------ACA-CAG--

AAAC--A-GA--CT------------TCT--T-TTC-------A-G--TC-G-T-GG-C---------A--A

TGATG-AAT-A--AAA-AA----TT-CA-TTGCA-AATCTTGT-GGAACAAA----TTT------GAG----

-------------GATCAAATCTCAG-C----------------C-ACA-ACTCAAAACACACA---A----

------------------TAA--G----GAA-TGTCTGG----------T---AAAACATTC-AAATCAA--

---AACACCC-------AAGGA-AT-----AAGGC---GT-----AATA------AT-TCCCAGA-TCGAGG

GGAAA-------------------------------------------------------------------

------------------------------------------------------------------------

--------------C--AAAAAT--------------------------GGAAAA-CCTT-------TTTTT

C----------------------CGAAAACAGAG--A--GTCCTAGC-----TTTTCCTCCT----------

--------------TATATC---TTA-----------TTCTTTAT---------------------------

-------------------------TTT--CTTTTT-GGGT--GTG-CCT-CC-TTG-CT--GGATAC--AA

AT-----------GACAT-A----TGAAAGT-ACTAGTG-TAAGTGTTTTCA--GAAC--------------

----ATATCA------------------------------GTGTAAT-A-T-G-AA-CTC-AAA-----AT-

AAAATT--GCAAAAA--TTAGG------------ACAAAATTT--CGCA-A-ATC--TGG-AAAGAG-----

---------GAG-CTCCT-------------------CGG-AGTCCAAG----ACGGTTTTGCA------

>Pv02Lk00700/1-553 Pv02Lk00700 undefined product 23746219:23746771 forward

---CAAAACCAA-----TTTCTTATTG--AAATCTTATGG-AAG-AA--T-------CC---CAACAG-AA-

TTTTGG-AC----------GAAAACCG-AC-AA-AT--TTTAAC--TC------------------------

-----------------------------------------TAAC-A--GACGAG---TATTCAG-------

---TT------AGAGAATGA---TC-ATACAGTT-TC--------------------------ACA-CAG--

AATC--G-GG-CCT------------TCT--T-TTC-------A-G--TC-G-T-GG-C---------A--A

TGATG-AAT-A--AAA-AT----TT-CA-TTGCA-AATCTTGT-GGATTGAA----TTT------GAT----

-------------GCTCAAATATGAG-C----------------C-GCA-ACTAAACACAAACA---A----

------------------TAA--G----GAA-TGTCTAG----------T---AAAAATTTC-AAATCAA--

---AACACCC-------AAGGC-AT-----AAGGC---GT-----AATA------AT-TCCCATG-CCGAGA

GTAAA-------------------------------------------------------------------

------------------------------------------------------------------------

--------------C--CAAACT--------------------------GGAAAA-CTTG-------TTTTT

T----------------------CTGAAACAGAA--C--GTCCTGAC-----TTTTCTTCCT----------

--------------TATATA---TTT-----------TTATGTAT---------------------------

-------------------------TTT--CTTTTT-GGGC--ATG-CCT-CC-TTGAAT--GGATAC--AA

AC-----------GACAT-A----TGAAAGT-ACTTGTC-TAAGTGTTTTCG--GAAC--------------

----TTATCA------------------------------ACGTAAT-A-T-G-AA-CAC-GAA-----AT-

AAATTT--GGAAAAT--CTAGA------------GCAAAATTT--CGCA-A-ATC--TGG-AAGCAG-----

---------AAG-CTCCT-------------------TGG-AGTCCAAG----AAGTTTCTGGAACATT-

>Pv02Lk00710/1-550 Pv02Lk00710 undefined product 23746776:23747325 forward

----AAATACAA-----TTTCTTGTTG--AAATCTTATCG-AGG-AA--T-------CC---CAACTA-AA-

TTTAGG-AC----------GAAACGCG-AC-AA-AT--TTCAA---TC------------------------

-----------------------------------------AAAC-G--GATGAG---TATTCAG-------

---TT------ATAAAATGA---TC-AAAAAGTT-TC--------------------------ACA-CAG--

AAAC--G-GA-CCT------------TCT--T-TTC-------A-G--TT-G-T-GC-C---------A--A

TGATG-AAT-A--AAA-AA----TT-AA-TTCCA-AATATTGT-GGAACAAA----TTT------AAG----

-------------GCTCAAATCTCAA-A----------------C-ACA-AATCAAAACACACA---A----

------------------TAA--G----GAG-TGTCTGG----------T---AAAAATCTC-AAATCAA--

---AACACCC-------AAGGA-AT-----AAGGT---GT-----AATC------AT-TCTCATA-TCGAGA

GGAAC-------------------------------------------------------------------

------------------------------------------------------------------------

--------------C--AAAACT--------------------------GAAAAA-CTTA-------TTTTT

C----------------------CAAAAACAGAA--A--GTCGTGCC-----TTTTTTTCCT----------

--------------TATATC---ATA-----------TTCTTTAT---------------------------

-------------------------TTT--CTTTTT-GGGC--GTG-CCT-CC-TTGAAT--GGATAC--AA

AC-----------AACAT-A----TAAAAGT-ACTAGTG-TAAGTGTTTTCG--AAAC--------------

----TTATCA------------------------------GCGTAAT-A-T-G-AA-CTC-ATA-----AT-

AAAATT--GCAAAAA--GTAGG------------GCAAAATTT--CGCA-A-ATC--TGG-AAAGAA-----

---------GAG-CTTCT-------------------TGG-AGTCAAAG----AAGGTTCTGCAACAT--

>Pv02Lk00720/1-554 Pv02Lk00720 undefined product 23747328:23747881 forward

-TCCAAAAACAA-----TTTCTTGTTG--AAATATTATCC-AGG-AA--T-------CC---CAACAT-AA-

TTTGGG-AC----------GAAACGCA-AC-AA-AT--TTCAAA--TC------------------------

-----------------------------------------AAAC-A--GAAAAG---TATTCCG-------

---TT------AGTATTTGA---TC-AAACAGTT-TC--------------------------ACA-CAG--

AAAC--A-GA-CCT------------CCC--T-TTC-------A-G--TC-G-C-GG-G---------A--A

TCACG-AAT-A--AAA-AA----TT-CA-TTGCA-AATCTTGT-TGAACGAA----TTT------GAG----

-------------GCTCAAATGTCAG-C----------------C-ACT-ACTCAATAAACACA---A----

------------------TAA--G----GAA-TGTCTGG----------T---AAAACTTTC-AAATCAA--

---AACACCC-------AAGGA-AT-----AAGGC---GT-----AATA------AT-TCCCAGA-CCGAGA

GCAAA-------------------------------------------------------------------

------------------------------------------------------------------------

--------------C--CAAACT--------------------------AGAAAA-CTTGT------TTTTC

-----------------------CGA-AATAGAA--C--GTCCTAGC-----TATACTTCCT----------

--------------GATAAC--TTTT-----------TTCTGTGT---------------------------

-------------------------TTT--CTTTTT-GGGC--ATC-CCT-CC-TTGACT--GGATAC--AA

AG-----------GACAT-A----TGAAAGT-ACTTGTG-TAAGTGTTTTCG--GAAG--------------

----TTCTCA------------------------------ATGTAAT-A-T-G-AA-CTT-AGA-----AT-

AAAATT--GCAAAAA--CTAGG------------GCAAAATTT--CGCA-T-ATT--TGG-AAAGAG-----

---------GAG-CTCCT-------------------TGG-CGTC-AAG----AAGTTTCTGGAACATT-

>Pv02Lk00730/1-539 Pv02Lk00730 undefined product 23747888:23748426 forward

------AAAAAA-----TTTCATGTTG--AAATATTATGG-AAG-AA--T-------TC---CAAAAG-AT-

TTTGGG-AT----------GAAACGCG-AC-AA-AT--TTCAAC--TC------------------------

-----------------------------------------AAAC-A--GACGAG---TATTCAG-------

---TT------AAAAAATGA---TA-AAACAGTT-TC--------------------------ACA-CAA--

AAAC--A-GA-CCT------------TAA--T-TTT-------A-G--AC-G-T-GG-C---------A--A

TGATG-AAT-A--AAA-AT----TT-CA-TTGGA-AATCTTGT-GAATCG-T----TTT------GAT----

-------------GCTCAAATATGAG-C----------------C-ACA-ATTAAATACACCCA---A----

------------------TAA--G----AAA-TGTCTGG----------T---AAAAATTTC-AAATTAA--

---AATACCC-------AAGGA-AT-----AAAGC---GT-----AATA------AT-TTTCAGA-CCGGGA

GTAAA-------------------------------------------------------------------

------------------------------------------------------------------------

--------------C--CAAACT--------------------------GGAAAA-CTTG-------TTTTT

C----------------------CGA-AACAGAA--C--TTCCTGAC-----TTTTCTTCCT----------

--------------TATATC---TTC-----------TTATGCCT---------------------------

-------------------------TTT--CTTTTT-TAGG--GTT-CCT-CC-TTGA-------------A

AC-----------GACAT-A----TGAAAGT-ACTAGTG-TAAGTGTTTTCG--GAAC--------------

----TTATCA------------------------------GTGTAAT-A-T-G-AA-CTC-AGA-----AT-

AAAATT--GCATAAA--CTATG------------GCAAAATTT--CGCA-A-ATA--TTG-AAAGAG-----

---------GAG-CTCCT-------------------TGG-AGTCTAAG----AAGTTTCTGGAACATT-

>Pv02Lk00740/1-552 Pv02Lk00740 undefined product 23748428:23748979 forward

-TCCAAAATCCA-----TTTCTCGTTG--AAA-CTTATGG-AAG-AA--T-------CC---TAACAG-AA-

TTTGGG-AC----------GAAATGTA-AC-TA-AT--TTAAGC--TC------------------------

-----------------------------------------AAAC-G--GATGAG---TATTCAA-------

---TT------AGAAAATGA---TC-AAACAGTT-TC--------------------------ACA-CAA--

AAAC--G-GA-CCT------------TCC--T-TTC-------A-G--TC-T-T-GG-C---------A--A

TGATG-AAT-A--AAA-AA----TT-CA-TTGCA-AATCTTGT-GGAATGAA----CTT------GAG----

-------------GCTGAAATCTCAG-C----------------C-ACA-A-TCAATAAACAGA---A----

------------------TAA--G----GAA-TGTCTGG----------T---AAAAATTTC-AAATCAA--

---AACACCA-------AGCGA-AT-----AAGGC---GT-----AATA------AT-TCCCAGA-CCGAGA

GCAAA-------------------------------------------------------------------

------------------------------------------------------------------------

--------------A--ACAACT--------------------------AGAAAA-CTTG-------TTTTT

C----------------------CAAAAACAGAA--C--GTCGTGAC-----TTTTCTTCCT----------

--------------TCTATC---TTT-----------TTATATGT---------------------------

-------------------------TTT--CTTTTT-GGGC--ATG-CCT-CC-TTGACT--GGATAC--AA

AC-----------GACAT-A----CCAAAGT-ACTTGTG-TAAGTGTTTTCG--GAAC--------------

----TTATCA------------------------------GCGTAAT-A-T-G-AA-CTC-ATA-----AT-

AAAATT--GCTTAAA--CTATG------------GCAAAATTT--CGCA-A-ATA--TTA-AAATAG-----

---------GAG-CTCCT-------------------TGG-AGTTTAAG----AAGTTTCTAGAACAT--

>Pv02Lk00750/1-552 Pv02Lk00750 undefined product 23748983:23749534 forward

--CCAAAATCAA-----TTTCTTGTTG--AAA-CTTATGG-AAG-AA--T-------CC---TAACAG-AA-

TGTGGG-AC----------GAAAAGTG-AC-AA-AT--TCAAGC--TC------------------------

-----------------------------------------AAAC-G--GACGAG---TATTCAA-------

---TT------AGAAAATGA---TC-AAATAGTT-TC--------------------------ACA-CAA--

AAAC--G-GA-CCT------------ACC--T-TTC-------A-G--TC-G-T-GG-C---------A--G

TGATA-AAT-A--AAA-AA----TT-CA-TTGGA-AATCTTGT-AGAACGAA----CTT------GAG----

-------------TCTAAAATCTCAG-C----------------C-ACA-ACTCAATACACACA---A----

------------------TAA--G----GAA-TGTCTGG----------T---AAAAACTTC-AAATCAA--

---AATACCC-------AAGGA-AT-----AAGGG---GT-----AATA------AT-TCCCAGA-CCGAGA

GCAAA-------------------------------------------------------------------

------------------------------------------------------------------------

--------------C--AACACT--------------------------GGAAAA-CTTG-------TTTTT

C----------------------CGAAAACAGAA--T--GTCCTGTC-----TTTTCTTCCT----------

--------------TATATC---TTT-----------TTTTGTGT---------------------------

-------------------------TTT--CGTTTT-GGGC--ATG-CCT-TC-TTGATT--GGATAC--AA

AC-----------AACAT-A----TGAAAGT-ACTTGTG-TAAGTGTTTTCG--GAAC--------------

----TTAGAA------------------------------GCGTAAT-A-T-G-AA-CTC-AAA-----AT-

AAAATT--GCATAAA--CTATC------------ACAAAATTT--CGCA-A-ATA--TTG-AAAAAG-----

---------AAT-CTCCT-------------------TGG-AGTCTAAG----AAATTTAAGGAACAT--

>Pv02Lk00760/1-553 Pv02Lk00760 undefined product 23749539:23750091 forward

---CAAAATCAA-----GTTCTTGTTG--AAATCTCAAGG-AAG-AA--T-------AC---TTACAC-AA-

TTTGGG-AC----------GAAACGCG-AA-CA-AT--TTTAGC--TC------------------------

-----------------------------------------AAAC-G--GACGAG---TATTCAG-------

---TT------AGAAAATGA---TC-AAACAGTT-TC--------------------------ACA-CAA--

AAAC--G-GA-CAT------------TAC--T-TTG-------A-G--TC-G-T-GG-A---------A--A

TGATG-AAT-A--AAA-AA----TT-CA-TTGGA-AATCTTGT-AGGACGAA----CTC------GAG----

-------------GCTCAAATTTCAG-C----------------C-ACA-ACTCAATACACGCA---A----

------------------TAA--G----GAA-TGTCTGG----------T---AAAATTTTT-AAATCCA--

---AACACCA-------AAGAA-AT-----AAGAC---GT-----AATA------AT-TCCTAGA-TCGAGA

GAAAA-------------------------------------------------------------------

------------------------------------------------------------------------

--------------C--AAAACT--------------------------GGAAAA-CTTG-------TTTTT

C----------------------CAAAAACAAAA--C--GTCCTGGC-----TTTTCTTCCT----------

--------------TATATC---CTT-----------TTCTGTGT---------------------------

-------------------------TTT--ATTTTT-GGGC--GTG-CCT-CC-TTGATT--GGAAAC--AT

AC-----------AACAT-A----TGAAAGT-ACTCCTG--AAGTGTTTTTG--GAAC--------------

----TTATCA------------------------------ACGTAAT-A-T-G-AA-CTC-AGA-----AT-

AAAATT--GCAAAAA--CAAGG------------GCAAAATTT--TTCA-A-ATA--TGG-AAAAAA-----

---------GGA-GCACC-------------------TTGGACTCCAAG----AAATTTCTGGAACATT-

>Pv02Lk00790/1-553 Pv02Lk00790 undefined product 23750474:23751026 forward

--CCAAAAAAAA-----TTTCTTCTTG--AAATCTTATGA-AAG-AA--T-------CC---CAACAT-AAT

TTTGGG-AC----------AAAACGCG-AC-AA-AT--TTCAGC--TC------------------------

-----------------------------------------AAAC-G--GACGAG---TATTCAA-------

---TT------AAAAAATGA---TA-AAATAGTT-TC--------------------------ACA-CAA--

AAAC--A-GA-TCT------------TCC--T-TTC-------A-G--TC-G-T-GG-C---------A--A

TGATG-AAT-A--AAA-AA----TT-TA-TTGCA-AACATTGT-GGAATGAA----TTT------GAG----

-------------GCTCAAATCTCAT-C----------------C-ACA-ACTTAATACACACA---A----

------------------TAA--G----CAA-TGTCTGG----------T---AAAAATTCC-AAATCAA--

---AACACCA-------ATGGA-AT-----AAAGG---GT-----CATA------AT-TCCCAGA-CCGAAA

G-AAA-------------------------------------------------------------------

------------------------------------------------------------------------

--------------C--AAAACT--------------------------GGAAAA-CTTG-------TTTTT

C----------------------TAAAAAAAGAA--C--GTCCTGGC-----TTTTGTTCCT----------

--------------TATATA---TTT-----------TTCTGTGT---------------------------

-------------------------TTT--CTTTTT-GGAC--GT--CCT-CC-TTGACT--GGATAC--AA

AC-----------GACAT-A----TGAAAAT-ACTTGTG-TAAGTGTTTTCG--GAAC--------------

----TTCTAA------------------------------GCGTAAT-A-T-G-AA-CTC-AGA-----AT-

AAAATT--GCAAAAA--CTAGG------------GCAAAATTT--TGCA-A-ATA--AGG-AAAGAG-----

---------GAG-CTCCT-------------------TGG-AGACCAAG----AAGTTTCTGGAACATT-

>Pv02Lk00800/1-541 Pv02Lk00800 undefined product 23751025:23751565 forward

-------------------TTTTTTTG--CAATCTTATGG-AAG-AA--T-------TC---CAATAG-AA-

TTTAGG-AC----------GAAATGCG-AC-AA-AT--TTCAGC--TT------------------------

-----------------------------------------AAAC-G--GGCGAG---TATTCAT-------

---TT------AGAAAATGA---TC-AAACAATT-TC--------------------------ACA-TAA--

AAAC--G-GA-CCT------------TCC--T-TTC-------A-G--TC-G-T-GG-C---------A--A

TGATA-AAC-A--AAA-AG----TT-CA-TTGCA-AATCTTGT-GGAACGAA----TTT------GAG----

-------------GTTCAAATCTCAG-C----------------C-ACA-TCTCATTACACACA---A----

------------------TAA--G----GTA-TGTCTGG----------T---AAAAATTTC-AAATCAA--

---AACACTC-------GAAGA-AT-----AAGGT---GT-----AAGA------AT-TCCCAGA-CCGAGA

GCAAA-------------------------------------------------------------------

------------------------------------------------------------------------

--------------C--AAAACT--------------------------GGAAAA-GTTG-------TTTTT

A----------------------CGGAAACAGAA--C--GTCCTGAC-----TTTTCTTTCT----------

--------------TATATC---TTT-----------TTCTGTGT---------------------------

-------------------------TTT--CTTTTT-GGGC--GTG-CCT-CC-TAGACT--GGATAC--AA

A------------GACAT-A----TGAAAGT-ACTTGTG-TAAGTTTTTTCA--GAAC--------------

----TTATCA------------------------------GCGTAAT-A-T-G-AA-CTC-ATA-----AT-

AAAATT--GCAAAAA--CAAGG------------GCAAAATTT--TGCA-A-ATA--TGG-AAAAAG-----

---------GAG-CACCT-------------------TGG-ACTTCAAG----AAGTTTCTGGAACATT-

>Pv02Lk00810/1-551 Pv02Lk00810 undefined product 23751568:23752118 forward

----CCCAAAAA-----ATTCTTGTTG--AAATCTTAGGA-AAG-AA--T-------CC---CAACAG-AA-

TTTGGG-AC----------GAAAGAAA-AC-AA-AT--TTCAGC--TC------------------------

-----------------------------------------AAAC-A--GACAAG---AATTCAG-------

---TT------TGAAAATGA---TC-AAAGAGTT-TA--------------------------ACA-TAG--

AAAC--G-AA-CAT------------TCC--A-TTC-------A-G--TC-G-T-GG-C---------A--A

TGATG-AAT-A--AAA-AA----TA-CA-TTGCA-AATCTTGT-GGAACAAT----TTT------GAG----

-------------GCTCAAATCTCAG-T----------------C-ATA-ACTCAATACACACA---A----

------------------TAA--T----GAA-TGTCTGG----------T---AAAAACTTC-AAATCAA--

---AACACCC-------AAGGC-AT-----AAGGG---GT-----AATA------AT-TCTCAGT-CTGAGA

GCAAA-------------------------------------------------------------------

------------------------------------------------------------------------

--------------C--AAAACT--------------------------GGAAAT-CTTG-------TTTTT

T----------------------CGAAAATAGAA--C--GTCAATGC-----TTTTCTTCCT----------

--------------TATATC---TTT-----------TTCTGTGT---------------------------

-------------------------TTT--CTTTTC-GGAT--GTG-CCT-CC-TTGACT--GGATAC--A-

AT-----------GACAT-A----TGAAAGT-ACTTGTC-TAAGTGATTTCG--GAAC--------------

----TTATCC------------------------------GCGTAAT-A-T-G-AA-CTT-AGA-----AT-

AAAATT--GGAAAAA--CTAGG------------GCAAAATTT--TGCA-T-ATA--TGG-AAAGAG-----

---------GAG-GTCCT-------------------TGG-ACTCCAAA----ATATTTCAGTAATATT-

>Pv02Lk00820/1-555 Pv02Lk00820 undefined product 23752120:23752674 forward

-TCCAAAAACAA-----TTTCTTGTTG--AAATCTTATGG-AAG-AA--A-------CG---CAACAA-AG-

TTTGGG-AT----------GAAATGCG-AC-AA-AT--TTCAGC--TC------------------------

-----------------------------------------AAAA-A--GACAAG---TATTCAG-------

---TT------GGAAAATGA---TC-CAACAGTT-TC--------------------------ACA-CAG--

AAAT--G-GA-CCT------------TGC--T-TTC-------A-G--TC-G-T-GG-C---------A--A

TGTTG-AAT-A--AAT-AT----TT-CA-TTGCA-AATCTTGT-GGAACGAA----TTT------GAG----

-------------GCTCAAATATCAG-T----------------C-ACA-TCTCAATACACACA---A----

------------------TAA--G----GAA-TGTCTAG----------T---AAAAACTTC-AAATCAA--

---AAC-CTA-------AAGGA-AT-----AAGGG---GT-----CATA------AT-TCCCGGA-CAGAGA

GCAAA-------------------------------------------------------------------

------------------------------------------------------------------------

--------------C--AAAATT--------------------------GAAAAA-CATT-------TTTTT

C----------------------CGAAAACAGAA--C--GTCTCTGC-----TTTTCTTCCT----------

--------------TATATC---TTT-----------TTCTATGC---------------------------

-------------------------ATT--CTTTTT-GGGC--GTG-CCT-CC-TTGACT--GGATAC--AA

AC-----------GACAT-A----TGAAAGT-ACTTGTC-TAAGTGTTTTTG--GAAC--------------

----TTATCA------------------------------GCGTAAT-A-T-G-AA-CTC-AGA-----AT-

AAAATT--GCAAAAA-ACTAGG------------GCAAAATTT--TGAA-A-ATA--TGA-AAAGAG-----

---------GAG-CTCCT-------------------TGG-AGTCCAAG----AAGTTTCTGGAACATT-

>Pv02Lk00830/1-550 Pv02Lk00830 undefined product 23752677:23753226 forward

--CCAAAATCAA-----TTTCTTGTTG--CAATATTATGG-AAG-AA--T-------CC---CAACAT-AA-

TTTCAT-AC----------GAAACGCG-AC-AA-AT--TTCAGC--TC------------------------

-----------------------------------------AAAC-A--AACGAG---TATTCAG-------

---TT------AGAAAATTA---TC-AAACAGTT-TC--------------------------ACA-CAA--

AAAC--G-GA-CCT------------TCC--T-TTA-------A-G--TC-G-T-GG-C---------A--A

TGATG-ATT-A--AAA-AA----TT-CA-TTGCA-AATCTTGT-GGAACGAA----TTT------GAG----

-------------GCTCAAATTTCAT-C----------------C-AAG-ACTCAATACACACA---A----

------------------TAA--G----GAA-TGTTTGG----------T---AAAGGTTTC-AAATTAA--

---AATACCC-------AAGGA-AT-----AAGAC---AT-----AATA------AC-TCTGAGA-CAGAGG

GCAAC-------------------------------------------------------------------

------------------------------------------------------------------------

--------------C--AATATT--------------------------GGAAAA-CTTG-------TTTTT

C----------------------CGAAAATAGAA--C--CTCCAGGC-----TTTTCTTCCT----------

--------------TATATC---TTT-----------TTTTG------------------------------

--------------------------TT--TTTTTT-GGGT--GTG-CCT-CA-TTGACT--GGATAC--AA

AC-----------GACAT-A----TGAAAGT-ACTTGTG-TAAGTGTTTTCG--GAAC--------------

----TTATCA------------------------------GCGTAAT-A-T-G-AA-CTC-ATA-----AT-

AAAATT--GCAAAAA--CTAGG------------GAAAATTTT--TGCA-A-ATA--TGG-AAAGAG-----

---------GTT-CTCCT-------------------TGG-AGTCCAAG----AAATTTCTGCAACATT-

>Pv02Lk00840/1-553 Pv02Lk00840 undefined product 23753229:23753781 forward

--CCAAAATAAA-----CTTCTTGTTG--AAATTTTTTGG-AAG-AA--T-------CT---TAATAG-AA-

TTTGGG-AC----------GAAACGCA-AC-AA-AT--TTCAGC--TC------------------------

-----------------------------------------AAAC-T--GATGAG---TATTCAG-------

---TT------AGAAAATGA---TC-AAATAGTT-TC--------------------------ACA-CAG--

AAAC--G-AA-CAT------------TCC--T-TTC-------A-G--TC-G-T-GA-C---------A--A

TGATG-AAT-A--AAA-AA----TT-CA-TTGCA-AATTGTTT-GGAACAAA----ATT------GAG----

-------------GCTCAAATCCTAG-C----------------C-ACA-ATTCAATACACACA---A----

------------------TAA--G----GAA-TGCCTGG----------T---AAAAACTTC-AAATCAA--

---AACACTA-------AAGGA-AT-----AAGGG---GT-----AATA------AT-TCCCAGT-CCAAGA

GCAAA-------------------------------------------------------------------

------------------------------------------------------------------------

--------------C--AAAACT--------------------------GGAAAA-CTTG-------TTTTT

T----------------------CGAAAACAAAA--C--ATCCTGCC-----CTTTCTTCCT----------

--------------TATATC---TTT-----------TTTTGTGT---------------------------

-------------------------TTT--CTTTTT-GGGC--GTG-CCT-CC-TTGACT--GGATAG--AA

AT-----------GACAT-A----TGAAAGT-AGTTGTG-TAAGTGTTTTCG--AAAC--------------

----TTATCA------------------------------GCGTAAT-A-T-G-AA-CGC-AGA-----AT-

AAAATT--GCAAAAA--CTAGG------------G-AAAAAAT--TGCA-A-ATA--TGG-AAAGAG-----

---------GAG-TTCCT-------------------TGC-AGTCCAAG----AAGTTTCTGGAACATT-

>Pv02Lk00850/1-551 Pv02Lk00850 undefined product 23753785:23754335 forward

---CAAACTAGA-----TTTCTTGTTG--TAATTTTATGG-AAG-AA--T-------CC---CAACAG-AA-

TTTGGG-AC----------GAAACGCG-AC-AA-AC--TTCAGC--TC------------------------

-----------------------------------------AAAC-G--AACGAG---TATTCAA-------

---TT------AGAAAATAA---CC-AAACAGTT-TC--------------------------ACA-CAT--

AAA---G-GA-CCT------------TCC--T-TTC-------G-A--TC-G-T-GG-C---------A--A

TGATG-ATT-A--AAA-AA----TT-CA-TTGCA-AATCTTGT-GGAACGAA----TTT------GAG----

-------------ACTCAAATTTCAG-C----------------C-AAG-ATTCAATACGCACA---A----

------------------TAA--G----GAA-TGTCTGG----------T---AAAAATTTC-AAATAAA--

---AAAACCT-------AAGGA-AT-----AAGGC---GT-----AATA------AG-TCCCAGA-CCGATG

GCAAA-------------------------------------------------------------------

------------------------------------------------------------------------

--------------C--AAAACT---------------------------GAAAA-CTTG-------TTTTT

T----------------------CGAAAATAGAA--T--GGCCTGGC-----TTTTCTTCCT----------

--------------TATATC---TTT-----------TTTTGTGT---------------------------

-------------------------TTT--CTTTTT-GGGC--GTG-CCT-CC-TTGACT--GGATAC--AA

AC-----------GACAT-A----TGAAAGT-ACTTGTG-TAAGTGTTTTCG--GAAC--------------

----TTATCA------------------------------GCGTAAT-A-T-A-AA-CTC-ATA-----AT-

AAAATT--GCAAAAA--CTTGG------------GCAAAATTT--TGCA-A-ATA--TGG-AAAGAG-----

---------GAG-CTCCT-------------------TGG-AGTCCAAG----AAGTTTATGGAACATT-

>Pv02Lk00860/1-550 Pv02Lk00860 undefined product 23754342:23754891 forward

-------AAAAA-----AATTCTTGTT--GAAATTTTAGG-AAG-AA--T-------TC---CAATAG-AA-

TTTGGG-AC----------GAAACGCT-AC-AA-AT--TTCAAC--TC------------------------

-----------------------------------------AAAC-G--GATGAG---TATTCAG-------

---TT------AGAAAATGA---TC-AAACAATT-TC--------------------------ACA-CAA--

AAAC--G-GA-CAT------------TCC--T-TTC-------A-G--TC-G-T-GG-C---------A--A

TGATG-AAT-A--CAA-AA----TT-GA-TTGCA-AATCTTGT-GGAACAAA----TTT------GAG----

-------------GCTGAAATCTTAG-C-----------------AAAA--CTCAATACACACA---A----

------------------TAT--G----GAA-TGTCTGG----------T---AAAAATTTC-AAATGAA--

---AACACTG-------AAGGA-AT-----AAGGT---GT-----ATTA------AT-TCCCAGA-CCTAGA

GCAAA-------------------------------------------------------------------

------------------------------------------------------------------------

--------------A--AAAACT--------------------------GGAAAA-CTTG-------TTTTT

C----------------------CGAAAACAGAA--C--GTCATGGTTT---TTTTTTTCCT----------

--------------TATATC---TTT-----------TTCTGTGT---------------------------

-------------------------TTT--CTTTTT-AGGC--GTG-CCT-CC-TTGATT--GGAAAC--AA

AC-----------GACAT-A----TGAAAGT-ACTTGTA-TAATTGTTTTCA--AAAT--------------

----TTATCA------------------------------GCGTAAT-A-T-G-AA-TTC-ATA-----AT-

AAAATT--ACAAAAA--CTTGG------------GCAAAATTT--CGCA-A-ATA--TGG-AAAGAG-----

---------GAG-CTCCT-------------------TGG-AGTTCAAG----AAGTTTCTGGAACATT-

>Pv02Lk00870/1-539 Pv02Lk00870 undefined product 33322635:33323173 forward

CTCCCGGAATAG-----TTTTTCC-TG--AAATTCCACCC-AAG-AA--T-------CT---CCACTG-CA-

TTTGGG-AC----------AAAACGCG-AC-AA-AT--TTCAAG--TC------------------------

-----------------------------------------AAAC-G--GATGAG---TATTCAC-------

---CCA-----TGATAAAAA---TC-AAACAGTT-TC--------------------------ACA-CAA--

AAAC--G-AA-CCT------------ATC--T-TTC-------A-G--CC-C-T-GG-C---------A--G

TGACG-AAT-A--A-A-AA--A-TT-TA-TTGCC-AATATTGT-GGGACGAA----TCT------GTG----

-------------GCTCAAACCTCAG-C----------------CAAAA--CTCAATAGACACA---G----

------------------TGA--C----GAA-TGTCTGG----------T--A-AAATTTTT-AGA-CCA--

--AAATACCC-------AAGGA-GT-----AAGGA---GT-----AATA------AG-TCCCAGA-CCGAGA

GTGAA-------------------------------------------------------------------

------------------------------------------------------------------------

--------------C--AAAACT--------------------------GA-----------------TTTT

C----------------------CGAAAAGAAAA--C--GTCCTGGC-----TTTTGTTCTC----------

--------------CGTATC--ATCT-----------TTTTGTGA---------------------------

-------------------------TTT--GTTTAT-GGAC--GTG-CCT-CC-TTGCCT--GGGTGC--AA

AC-----------AACAT-A----CGAAAAT-GCTTATG-T-------------GCA-TT------------

----TTTTCA------------------------------GCGCAAT-A-C-G-GA-CCC-AGA-----AT-

GAAATT--GCAGAAA--TTAGG------------CCGAAATTT--CATA-A-GTT-TCGG--TAGAG-----

---------GAT-AGCCT-------------------TGG-TTTGCACA----AAATTTCTGAAAAATTC

>Pv02Lk00880/1-541 Pv02Lk00880 undefined product 33323173:33323713 forward

CTCCCGGAATAG-----TTTTTTCAAG--AAATTCCACCC-AAG-AA--T-------CT---CCACTG-AA-

TTTGGG-AC----------AAAACGCG-AC-AA-AA--TTAAGG--TC------------------------

-----------------------------------------AAAC-G--GATGAG---TATTCAC-------

---ACA-----CGAAAAAAA---TC-AAACAGTT-TC--------------------------ACA-CAC--

AAAC--G-AA-CCT------------ATC--T-TTC-------A-G--CT-C-T-GG-C---------A--G

TGACG-AAT-A--A-A-AA--A-TT-TA-TTGCC-AATCTTGT-GGGACGAA----TCT------GGG----

-------------GCTCAAACCTCAG-C----------------CAAAA--CTCAATAGACACA---G----

------------------TGA--C----GAA-TGTCTGG----------T--A-AAAATTTT-AGA-CCA--

--AAATACCC-------AAGGA-GT-----AAGGT---GT-----AATA------AG-TCCCAGA-CCGAGA

GTGAA-------------------------------------------------------------------

------------------------------------------------------------------------

--------------A--AAAATC--------------------------GA-----------------TTTT

C----------------------CGAAAAGAAAA--C--GTCCTGGC-----TTTTGTTCCC----------

--------------CGTATC--ATCT-----------TTTTGTGA---------------------------

-------------------------TTT--GTTTAT-GGAC--GTG-GCT-AC-TTGCCT--GGGTGC--AA

AC-----------AACGT-A----GGAAAAT-GCTTGTG-T-------------GAATTT------------

----TTTTCA------------------------------GCGCAAT-A-C-G-GA-CCC-AGT-----AT-

GAAATT--GCAGAAA--TTAGG------------CCGAAATTT--CACA-A-GTT-TCGG--TAGAG-----

---------GAT-AGCCT-------------------TGG-TTTGCACA----AAATTTCTGAAAAATTC

>Pv02Lk00890/1-538 Pv02Lk00890 undefined product 33323713:33324250 forward

CTCCCGGAATAG-----TTTTTTCCTG--AAATTCCACCC-AAG-AA--T-------CT---CCACTG-AA-

TTTCGG-AC----------AAAACGTG-AC-AA-AT--TTCAGG--TC------------------------

-----------------------------------------AAAC-G--AATGAG---TATTCAC-------

---ACA-----CGAAAAAAA---TC-AAACTGTT-TC--------------------------ACA-CAC--

AAAC--G-AA-CCT------------ATC--T-TTC-------A-G--CC-C-T-GC-C---------A--G

TGACG-AAT-A--A-A-AA--A-TT-TA-TTGCC-AATCTTGT-GGGACGAA----TCA------GGG----

-------------GCTCAAACCTCAG-C----------------CAAAA--CTCAATAGACACA---G----

------------------TGA--T----GAA-TGTCTGA----------T--A-AAAATTGC-AGT-CCA--

--AAATACCC-------AAGGA-GT-----AAGGC---GT-----AGTA------AG-TCCCAGA-TCGAGA

GTGAA-------------------------------------------------------------------

------------------------------------------------------------------------

--------------C--AAAACC--------------------------GA-----------------TTTT

C----------------------CGAAAATAAAA--C--GTCCAGGC-----TTTTGTTCCT----------

--------------CGTATC--TTCT-----------TTTAGTGA---------------------------

-------------------------TTC--GTTTAT-GGAC--GTG-CCT-CC-TTGCCT--GGGTGC--AA

AC-----------AACAT-A----CGAAAGT-GCTTGTG-T-------------GAAT-T------------

----TTTTCA------------------------------GCGCAAT-A-C-G-GA-CCC-ATA-----AT-

GAAATT--GCAGAAA--TTAGG------------CCGGAATTT--CACA-A-GTT-TCGG--TAGAG-----

---------GAT-AGCCT-------------------TGG-TTTGCACA----AAATTTCTGAAAAAA--

>Pv02Lk00900/1-529 Pv02Lk00900 undefined product 33324253:33324781 forward

CTCCCAGAATAG------TTTTTCCTG--AAATTCCACCC-AAG-AA--T-------CT---CCACTG-AA-

TTTGGG-AC----------AAAACGCG-AC-AA-AT--TGCATG--TC------------------------

-----------------------------------------AAAC-G--GATGAG---TATTCAC-------

---CCA-----CGAAAAAAA---TC-AAACAGTT-TC--------------------------ACA-CAC--

AAAC--G-AA-CCT------------TCC--T-TTC-------A-G--CC-C-T-GG-C---------A--G

TGACG-AAT-A--A-A-AA--A-TT-TA-TTGCC-AATCTTGT-GGGACCAA----TCT------GGG----

-------------GCTCAAACCTCAG-C----------------CAAAA--CTCAATAGACACA---G----

------------------CGA--C----AAA-TGTCTGA----------T--A-AAAATTTC-AGT-GCA--

--AAATACCC-------AAGGA-CT-----AAGGC---GT-----AGTA------AG-TCCCAGA-CCGAGA

GTGAA-------------------------------------------------------------------

------------------------------------------------------------------------

--------------C--AAAACC--------------------------GA-----------------TTTT

C----------------------CGAAAAGAAAA--C--GTCCAGGC-----TTTTCTTCCC----------

--------------CGTATC--TTCT-----------TTTTGTGA---------------------------

-------------------------TTC--GTTTAT-GGAC--GTG-CCT-CC-TTGCCT--GGGTGC--GA

AC-----------AACAT-A----CGAAAGT-GCTCGCG-T-------------GAATTT------------

----TTTTCA------------------------------GCGCAAT-A-C-G-GA-CTC-AGA-----AT-

GAAATT--GCAGAAA-------------------------TTT--CACA-A-GTT-TCGG--TAGAG-----

---------GAT-AGCCT-------------------TGG-TTTGCACA----AAATTTCTAAAAAATTC

>Pv02Lk00910/1-539 Pv02Lk00910 undefined product 33324781:33325319 forward

CTCCCAGAATAG-----TTTTTTACTG--AAATTCCACCC-AAG-AA--T-------CT---CCACTG-AA-

TTTGGG-AC----------AAAACGCG-AA-AA-AT--TTCAGG--TC------------------------

-----------------------------------------AAAC-G--GATGAG---TATTCAC-------

---ACA-----CGAAAAAAA---TC-AAACAGTT-TC--------------------------ACA-CAC--

AAAC--G-AA-CCT------------TTC--T-ATC-------A-G--CC-T-T-GG-C---------C--G

TGACG-AAT-A--A-A-AA--A-TT-TA-TTGCC-AATCTTGT-GGGACGAA----TCT------GGG----

-------------GCTCAAACCTCAG-C----------------CAAAA--CTCAATAGACACT---G----

------------------TGA--C----GAA-TGTCTGG----------T--A-AAAATTTC-AGT-CCA--

--AAATACCC-------ATAGA-GT-----AAGGC---GT-----AGTA------AG-TCCCAGA-CCGAGA

GTGAA-------------------------------------------------------------------

------------------------------------------------------------------------

--------------C--AAAACC--------------------------GA-----------------TTTT

C----------------------CGAAAAGAAAA--C--GTCCAGGC-----TTTTCTTTCC----------

--------------CGTATC--TTCT-----------TTTAGTGA---------------------------

-------------------------TTC--GTTTAT-GGAC--GTG-CCT-CC-TTGCCT--GGGTGC--AA

AC-----------AACAT-A----CGAAAGT-GCTTGTG-T-------------GAATTT------------

----TTTGCA------------------------------GCGCAAT-A-C-G-GA-ACC-AGA-----AT-

TAAATT--GCAGAAA--TTAGG------------TCGGAATTT--CACA-A-GTT-TCGG--TAGAG-----

---------GAT-AGCAT-------------------TGG-TTTGCACA----AAATTTCTGAAAACT--

>Pv02Lk00930/1-544 Pv02Lk00930 undefined product 49031862:49032405 forward

----------------TTTTTTTTCTG--AAATTCTATGT-AAG-AT--T-------CT---CAATAG-AT-

TTAGGA-CC----------ATACGCGA-AG-AA---------TT--TC------------------------

-----------------------------------------ACAT-G--CATGAT---TATTCAG-------

---TCG-----GGAAAAAAA---TC-AAACAGTT-TT--------------------------ACA-CAC--

AAAGGTG-AA-CCT------------TCC--T-TTC-------A-G--CC-C-T-GG-C---------A--A

TGATG-AAT-A--CAA-AA----AT-TA-TTGCA-AATCCGGT-GAAACGAA----TTT------GAG----

-------------GCTAAAACCTTGG-C----------------T-ACA-ACTCAGTAGACACA---A----

------------------TAA--A----GAA-CGTCTGA----------T---ACAAAATTC-ATATCAA--

---AACAACC-------AAGGA-AT-----AAGGC---GT-----AATA------AG-TCTTAGA-TTGAGA

GGAAA-------------------------------------------------------------------

------------------------------------------------------------------------

--------------T--TTTTTT--------------------------GAAAAA-CTTG-------TTTTT

G----------------------CAAAAATGAAA--T--CTCCTAGC-----TTTTCCTCGC----------

--------------CATATT---TCT-----------TTTTGTGT---------------------------

-------------------------TTT--GTTTTT-GGAT--GTG-CCT-CC-TTAGAT--GGATAT--AA

AA-----------AACAT-A----TGAAATT-ACTTTTG-TGAATTTTTTCA--GAAT--------------

----TTTTCA------------------------------ACGTAGT-A-A-G-AG-CTC-TGG-----AA-

AAAATT--GCAGAAA--CCAGG------------GCCAAACTT--TAAA-A-ATA--TGG-AAAAAGGA---

---------TAT-AGCTT-------------------TGG-AGGGCCCA----AAATTTTTGCAAAATT-

>Pv02Lk00950/1-544 Pv02Lk00950 undefined product 49032880:49033423 forward

--TCCCAAAAGG----ATTTTCTGTTG--AAATTCAATTT-AAG-AG--T-------CT---CAAGAG-AA-

TTTGAG-AC----------AAAACGCG-AC-AA-AT--TTCAGG--TC------------------------

-----------------------------------------AAAC-G--GATGAG---TATTCAG-------

---TCA-----GGAAAAAAA---TT-AAACAGTT-TT--------------------------ACA-CAG--

AGAGACA-AA-CTT------------TTC--T-TTC-------G-G--CC---T-GG-C---------A--A

TGATG-AAT-A--AAA-AA----TT-TA-TTGCA-AATTTTAT-GGAACAAA----TTT------GAG----

-------------GTCGAAACCTTGC-C----------------C-ACA-ACTCAGTAGACACA---A----

------------------AAA--G----GAA-CGTCTGA----------T---AAAATTTGT-AGATAAA--

---AAATACA--------AGG------------------------ACTA------AG-TCCCAGA-CCAAGA

GGAAA-------------------------------------------------------------------

------------------------------------------------------------------------

--------------C--AAAATT--------------------------GGAGAA-CTTG-------TTTTT

C----------------------CAAAAACGAAA--C--GTCTTGGC-----TTCTCCTCGT----------

--------------CATATC--TTTT-----------TTATGTGT---------------------------

-------------------------TTT--GTCTTT-TGAC--GTG-CCT-CC-TTAGCT--GATGCA--AA

AA-----------AACAT-A----TGAAAGT-ACTTGTA-TGAATTTTTTGG--GAAA--------------

----TTTTCA------------------------------ACGTAAT-A-T-G-AG-CTT-CGA-----AA-

-AAATT--GTAGAAA--CCGAG------------GTTGAATTT--AAAA-A-AAT--AGG-AAATAG-----

---------GAT-AGCCC-------------------CGA-AGGGCCAA-----AATTTTTTAAAAAA--

>Pv02Lk00960/1-543 Pv02Lk00960 undefined product 49033427:49033969 forward

--TCCCAAAATG----ATTCTCTGCTG--AAATTCTATGT-AAG-AG--T-------CT---CAAAAG-AA-

TTTTTG-AA----------AAAACACG-AC-AA-AT--TTCAGG--TC------------------------

-----------------------------------------AAAC-A--GATGAG---TATTTAG-------

---TGA-----AGAAAAAA----TC-AAATAGTT-TC--------------------------ACA-CAA--

AGAGGCG-AA-CTT------------TCC--T-TTC-------A-G--TC-T-T-GA-C---------A--A

TGATG-AAT-A--AAA-TA----TT-TA-TTACA-AATTCTGT-GGAGCTAA----TTT------GAG----

-------------GCTGAAACTTTGG-C----------------C-ACA-ATCCATTAGACACA---A----

------------------TAA--G----GAA-CGTCTGA----------T---AAATATTTC-CAATCAA--

---AAGTTAA--------AGG------------------------AATA------AG-TCCCAGA-TTGGGA

GGAAA-------------------------------------------------------------------

------------------------------------------------------------------------

--------------C--AAAATT--------------------------AAGAAA-GTTG-------TTTTT

C----------------------AAAAAACGAAA--T--GTTTTGGC-----TCGTCCTCGT----------

--------------CATATA---TAT-----------TTT--TGT---------------------------

-------------------------TTT--CTTTTT-GGAC--GTG-TCT-CC-TTAGCT--GAATGC--AA

AT-----------AACAT-A----GAAAAAT-ACT-GTG-TGATTTTTTTTG--GAAT--------------

----TTTTCA------------------------------ATGTAAT-A-T-G-AG-CTC-TGG-----AA-

AAAATT--GTAGAAA--CTAGG------------GCTAAATTT--AAAA-A-ATA--TGG-AAAACG-----

---------GAT-TGCCT-------------------TGG-AGGGCCCA----ATATTTTTGAAAAATT-

>Pv02Lk00970/1-529 Pv02Lk00970 undefined product 49033971:49034499 forward

--TCCCGAAAGG----ATTTTCTACTA--AAATTCTATGT-AAT-AG--T-------CT---CAAAAA-AA-

TTTGAG-AC----------AAAACGCG-AC-AA-AT--TTCAAG--TC------------------------

-----------------------------------------AAAC-G--GATGAG---TATTTAG-------

---TC------GGAAAAAAA---TC-AAACAGTT-TC--------------------------ACA-CAC--

AGATGTG-AA-CCT------------TCC--T-TTC-------A-T--GC-A-T-GA-C---------A--A

TGATG-GAT-A--CAA-AA----TT-TA-CTACC-TATCTTGT-GGAACGAA----TTT------AAG----

-------------GTTGAAACCCATG-A----------------C-AAA-AT--------------------

----------------------------GAA-CGTCTCC----------T---AAAATTTTC-AGATCGA--

---AAATCCA--------AGG-------------------------ATT------GA-GTACCAA-CCGAGA

GGAAA-------------------------------------------------------------------

------------------------------------------------------------------------

--------------A--AGAATT--------------------------GGAAAA-TTTG----------TT

C----------------------CAAAAACGAAA--C--GTCCTTGC-----TTCTCCTTGT----------

--------------CATATA---TTT-----------TTATGTGT---------------------------

-------------------------TTT--ATTATT-GGAT--GTG-CCT-AC-TTAGTT--GGATGC--AA

AC-----------AATAT-A----TGAAAGT-ACTTGTG-TGAATTTTTTTT-TG-GAAT------------

----TTTTCA------------------------------ATGAAAT-A-T-G-AT-CAC-CGA-----AA-

AAAATT--GCAGAAA--CCAAG------------GTGAAATTT--CAAA-T-ATC--TTC-AAAGAGGATA-

---------GAT-AGCCT-------------------TGA-AGTGCCCA----AAATTTATGAAAAA---

>Pv02Sk00010/1-530 Pv02Sk00010 undefined product 439593:440122 forward

CTCCCGCAATAG-----TTTTTTCCTG--AAATTCCACCC-AAG-AA--T-------CT---CCACTG-AA-

TTTGGG-AC----------AAAACGCG-GC-AG-AT--TTCAGA--TC------------------------

-----------------------------------------AAAC-G--GATGAG---TATTCAC-------

---CCA-----CGAAAAAAA---TT-AAACAGTT-TG--------------------------ACA-CAC--

AAAC--G-AA-CCT------------TCC--T-TTC-------A-G--CC-C-T-GG-C---------A--G

TGAGG-AAT-A--A-A-AA--A-TT-TA-TTTCC-AATCTTGT-GGGACGAA----TCT------GGG----

-------------GCTCAAAC------------------------------CTCAGTAGACACA---G----

------------------TGA--C----GAA-TGTCCGG----------T--A-AAAATTTC-AGA-CCA--

--AAATACCC-------AAGGA-GA-----AAGGC---GT-----GGTG------AG-TCCCAGA-CCGAGA

GTGAA-------------------------------------------------------------------

------------------------------------------------------------------------

--------------C--AAAACC--------------------------GG-----------------TTTG

C----------------------CGAAAACAAAA--C--GTCCTGGC-----TTTTCTTCCC----------

--------------CGTATC--TTCT-----------TTTTGTGA---------------------------

-------------------------TTC--GTTTAT-GGAC--GTG-CCT-CC-TTGCCT--GGGTGC--AA

AC-----------AATAT-A----CGAAAGT-GCTTGTG-T-------------GAATTC------------

----TTTTCA------------------------------GCGCCAT-A-C-G-GA-CCC-AGA-----AT-

GAAATT--GCAGAAA--TTAGG------------CCGGAATTT--CACA-A-GTT-TCGG--TAGAG-----

---------GAT-AGCCT-------------------TGG-TTTGCACA----AAATTTCTGAAAAATTC

>Pv02Sk00020/1-540 Pv02Sk00020 undefined product 440123:440662 forward

-TTCCAGAATAG-----TTTTTTCCTG--AAATTCCACCC-AAG-AA--T-------CT---CCACTG-TA-

TTTGGG-AA----------AAAACCCG-AC-AA-AT--TTCAGG--TC------------------------

-----------------------------------------AAAC-G--GATGAG---TATTCAC-------

---CCA-----CGAAAAAAA---TC-AAACAGTT-TC--------------------------ACA-CAC--

AAAC--G-AA-CCT------------TCC--T-TTC-------A-G--CC-C-T-GG-C---------A--G

TGACG-AAT-A--A-A-AA--A-TT-TA-TTTCC-AATCTTGT-GGGACGAA----TCT------GGG----

-------------GCTCAAACCTCTG-C----------------CAAAA--CTCAGTAGACACA---G----

------------------TGA--C----GAA-TTTCCGG----------T--A-AAAATTTA-AGA-CCA--

--AAATACCC-------AAGGA-GT-----AAGGC---GT-----AGTG------AG-TCCCAGA-CCGAGA

GTGAA-------------------------------------------------------------------

------------------------------------------------------------------------

--------------A--AAAACC--------------------------GG-----------------TTTT

C----------------------CGAAAAAAAAA--C--GTCCTGGC-----TTTTCTTCCC----------

--------------CGTATC--TTCT-----------TTTTGTGA---------------------------

-------------------------TTC--GTTTAT-GGAC--GTG-CCT-CC-TTGCCT--GGGTGC--AA

AC-----------AACAT-A----CGAAAGT-GCTTGTG-T-------------GAATTC------------

----TTTTCA------------------------------GCGCAAT-A-C-G-TA-CCC-ATA-----AT-

GAAATT--GCAGAAA--TTAGA------------CCGGAATTT--CACA-A-GTT-TCGG--TAGAG-----

---------GAT-AGCCT-------------------TGG-TTTGCACA----AAATTTCTGAAAAATTC

>Pv02Sk00030/1-537 Pv02Sk00030 undefined product 440662:441198 forward

CTCCCGGAATAG-----TTTTTTCCTG--AAACTCCACCC-AAG-AA--T-------CT---CCTCTG-AA-

TTTGGC-AC----------AAAACGGG-AC-AG-AT--TTCAGG--TC------------------------

-----------------------------------------AAAC-G--GATGAG---TATTCAC-------

---CCA-----CGAAAAAAA---TC-AAACAGTT-TC--------------------------ACA-CAC--

AAGC--G-AA-CCT------------TCC--T-TTC-------A-G--CC-C-T-GG-C---------A--G

TGACG-AAT-A--A-A-AA--A-TT-TA-TTGCC-AATCTTGT-GCGACGAA----TCT------GGG----

-------------GCTCAAACCTCAT-C----------------CAAAA--CTCAGTAGACACA---G----

------------------TGA--C----GAA-TGTCCGG----------T--A-AAAATTTC-AGA-CCA--

--AAATACCC-------AAGGA-GT-----AAGGC---GA-----AGTG------AG-TCCCAGA-CAGACA

GTGAA-------------------------------------------------------------------

------------------------------------------------------------------------

--------------C--AAAACC--------------------------GG-----------------TTTT

C----------------------CGAAAACAAAA--C--GTCCTGGC-----TTTTCTTCCC----------

--------------CGTATC--TTCT-----------TTTTGTGA---------------------------

-------------------------TTC--GTTTAT-GGAC--GTG-CCT-CC-TTGCCT--GGGTGC--AA

AC-----------AACAT-A----CGAAAGT-GCTTGTG-T-------------GAATTC------------

----TTTTCA------------------------------GCGCAAT-A-C-G-GA-CCC-ATA-----AT-

GAAATT--GCAGAAA--TTAGG------------CCGGAATTT--CACA-A-GTT-TCT-----CGG-----

---------TAT-AGCCT-------------------TGG-TTTGCACA-----AATTTCTGAAAAATTC

>Pv02Sk00040/1-541 Pv02Sk00040 undefined product 441198:441738 forward

CTCCCGGAATAG--TTTTTTTTTTCTG--AAACTCCACCC-AAG-AA--T-------CT---CCACTG-AA-

TTTGGG-AA----------AAAACGCG-AC-AG-AT--TTCAGG--TC------------------------

-----------------------------------------AAAC-A--GATGAG---TATTCAC-------

---CCA-----CG-AAAAAA---TC-AAACAGTT-TC--------------------------ACA-CAC--

TAAC--G-AA-CCT------------TCC--T-TTC-------A-G--CC-C-T-GG-C---------A--G

TGACG-AGT-A--A-A-AA--A-TT-TA-TTGCC-AATCTTGT-GGGACGAA----TCT------GGG----

-------------GCTCAAACCTCAG-C----------------CAAAA--CTCAGTAGACACA---G----

------------------TGA--C----GAA-TGTCCGG----------T--A-TAAATTTC-AGA-CCA--

--AAATACCC-------AAGGA-GT-----AAGGC---GT-----AGTG------AG-TCCCAGA-CCGAGA

GTGAA-------------------------------------------------------------------

------------------------------------------------------------------------

--------------C--AAAACC--------------------------GG-----------------TTTT

T----------------------CG-AAAAAAAA--C--GTCCTGGC-----TTTTCTTCCC----------

--------------CGTATC--TTCT-----------TTTTGTGA---------------------------

-------------------------TTC--GTTTAT-GGAC--GTG-CCT-CT-TTGCCT--GGGTGC--AA

AC-----------AACAT-A----CGAAAGT-GCTTGTG-T-------------GAATTC------------

----TTTTCA------------------------------GCGCTAT-A-C-G-GA-CCC-ATA-----AT-

GAAATT--GCAGAAA--TTAGG------------CCGGAATTT--CACA-A-GTT-TCGG--TATAG-----

---------GAT-AGCCT-------------------TGG-TTTGCACA-----AATTTCTGAAAAATTC

>Pv02Sk00050/1-541 Pv02Sk00050 undefined product 446441:446981 forward

CTCCCGGAATAG-----TTTTTTTCTG--AAACTCCACCC-AAG-AA--T-------AT---CCACTG-AA-

TATGGG-AA----------AAATTGCG-AC-AG-AT--TTCACG--TC------------------------

-----------------------------------------AAAC-A--GATAAG---TATTCAC-------

---CCA-----CGAAAAAAA---TC-AAACAGTT-TC--------------------------ACA-CAC--

TAAC--G-AA-CCT------------TCC--T-TTC-------A-T--CC-C-T-GG-C---------A--G

TGACG-AGT-A--A-A-AA--A-TT-TA-TTGCC-AATCTTGT-GGGACGAA----TCT------GGG----

-------------GCTCAAACCTCAG-C----------------TAAAA--CTCAGTAGACACA---G----

------------------TGA--C----GAA-TGTCCGG----------T--A-AAAATTTA-AGA-CCA--

--AAATACCC-------AAGGA-GT-----AAGGC---GT-----AGTG------AG-TCCCAGA-TCGAGA

GTGAA-------------------------------------------------------------------

------------------------------------------------------------------------

--------------C--AAAACC--------------------------GG-----------------TTTT

G----------------------CGAAAACAAAA--C--GTCCTGGA-----TTTTCTTCCC----------

--------------CGTATC--TTCT-----------TTTTGAGA---------------------------

-------------------------TTT--TTTTAT-GGAC--GTG-GCT-TC-TTGCCT--GGGTGC--AA

AC-----------AACAT-A----CGAAAGT-GCTTGTG-T-------------GAATTA------------

----TTTTCA------------------------------GCGCAAT-A-C-G-GA-CCC-ATA-----AT-

GAAATT--GCAGAAA--TTAGG------------CCAGAATTT--CACA-A-GTT-TCGG--TAGAG-----

---------GAT-AGCCT-------------------TGG-TTTGCACA----AAATTTCTGAAAAATTC

>Pv02Sk00060/1-540 Pv02Sk00060 undefined product 459682:460221 forward

CTCCCGGAATAG-----TTTTTTCCTG--AAATTCCACCC-AAG-AA--T-------CT---CCACTG-AA-

TTTGGG-AC----------AAAACGCG-AC-AA-AA--ATCAGG--TC------------------------

-----------------------------------------AAAC-G--GATGAG---TATTCAC-------

---CCA-----CGAAAAAAA---TC-AAACAGTT-TC--------------------------ACA-CAC--

AAAC--G-AA-CAT------------TCC--T-TTC-------T-G--CA-C-T-AG-C---------A--G

TTACG-AAT-A--A-A-AA--A-TT-TA-TTGCC-AATCTTGT-GGGACGAA----TCT------GGG----

-------------GTTCAAACCTCAG-C----------------CAAAA--CTCAATATACACA---G----

------------------TGA--C----GAA-TGTCTGG----------T--A-AAAATTTG-AGA-CCA--

--AAATACCC-------AAGGA-GT-----AAGGC---GT-----AGTA------AG-TCCCAGA-CCTAGA

GTGAA-------------------------------------------------------------------

------------------------------------------------------------------------

--------------C--AAAACC--------------------------GG-----------------TTTT

C----------------------CGAAAACAAAA--C--GTCCTGGA-----TTTTCTTTCC----------

--------------CGTATC--TTCT-----------TTTTGTGA---------------------------

-------------------------TTC--GTTTAT-GGAC--GTG-CCT-CC-TTGCCT--GGGTGC--AA

AC-----------AACAT-A----CGAAAGT-GCTCGTG-T-------------GAATTT------------

----TTTTCA------------------------------GCGCGAT-A-C-G-GA-CCC--GA-----CG-

GAAATT--GCAGAAA--TTAGG------------CCGGAATTT--CACA-A-GTT-TCGG--TAAAG-----

---------GAT-AGCCT-------------------TGG-TTTGCACA----AAATTTCTGAAAAATTC

>Pv02Sk00070/1-515 Pv02Sk00070 undefined product 513818:514332 forward

--CCAGAATAAT-----TTTTTTCCTA--AAATTCCACGT-AAG-AA--T-------AT---GGACTG-AA-

TTTGGG-AA----------AAAATGCC-AC-AA-AT--TTCACG--TC------------------------

-----------------------------------------AAAC-G--GATGAG---TATTCAC-------

---CTA-----GAAAAAAAG---TC-AAACAGTT-TC--------------------------ACA-CAC--

AAAC--G-AA-CCT------------TCC--T-TTC-------A-A--CC-C-T-AG-C---------A--G

TGATG-AAT-A--A-A-AA--A-TT-TG-TTGAA-AATCTTGT-GGGACGAA----TCT------GGG----

-------------GCTCAAATCTCAG-C----------------CAAAA--CTCAATACACACA---A----

------------------TGA--C----GAA-TGTCTGG----------T--A-CAATTTTC-AGA-CCA--

--AAAGACCC-------AAGGA-AT-----AGGGC---GT-----AGTA------AG-ACCCAGA-CCGAGA

GCAAA-------------------------------------------------------------------

------------------------------------------------------------------------

--------------C--AAAACC--------------------------GG-----------------TTTT

C----------------------CGAAAACAAAA--T--GTCGTGGT-----TTTTCTTCCC----------

--------------TATATC--TCCA-----------TTTTGTGA---------------------------

-------------------------TTA--ATTTAT-GGAC--GTG-CCC-CC--TCGCA--GGGTGC--AA

AC-----------AGCAC-A----TGAAAGT-ACTTGTG-T-------------G-----------------

--------------------------------------------AAT-TTTT-CAA-CAC-AAA-----AT-

AAAATT--GCAAAAA--GTAGG------------GCGAAATTT--CATA-A-ATT-TTGC--AAGTA-----

---------GTT-GGCCT-------------------TGGTTTTTCACA----AAATTT-----------

>Pv02Sk00080/1-528 Pv02Sk00080 undefined product 514335:514862 forward

--TCCGAAATAA-----TTTTTTCCTG--AAATTCCACGT-AAG-AA--T-------CT---GGACTG-AA-

TTTGGG-AT----------AAAACGCC-AC-AA-AT--TTCACG--TC------------------------

-----------------------------------------AAAC-G--GATGAG---TATTCAC-------

---CTA-----GGAAAAAAG---TC-AAACAGTT-TC--------------------------ATA-CAC--

AAAC--G-AA-CCT------------TCC--T-TTC-------A-A--CC-C-T-GG-C---------A--G

TGAAG-AAT-A--A-A-AA--A-TT-TG--TGAA-AATCTTGT-GGGATGGA----TTT------GGG----

-------------GCTCAAATCTCAC-C----------------CAAAA--CTCAATGCACACA---A----

------------------TGA--C----GAA-TGTCTGG----------T--A-AAATTTTC-AGA-CCA--

--AAAGACCC-------AAGGA-AT-----AGGGC---GT-----AGTA------AG-ACCCAGA-CCGAGA

GCGAA-------------------------------------------------------------------

------------------------------------------------------------------------

--------------C--AAAACC--------------------------GA-----------------TTTT

C----------------------CGAAAACAAAA--T--GTCCTGCA-----TTTTCTTCCC----------

--------------TACATG--TCCT-----------TCTTGTGA---------------------------

-------------------------TTT--GTTTAT-GGAC--GTG-CCT-CC-TTCGCA--GGGTGC--AA

AC-----------AGCAC-A----TGAAAGT-ACTTGTG-T-------------GAAT--------------

----TTTTCA------------------------------ACGCAAT-TTTT-CAA-CAC-ATA-----AT-

AAAATT--GCAGAAA--GTAGG------------ACGAAATTT--CACA-A-ATT-TTGC--TAGAG-----

---------GAT-AGCCT-------------------TGGTTTTTCACA----AAATTT-----------

>Pv02Sk00090/1-529 Pv02Sk00090 undefined product 778477:779005 forward

-TCCCGAAATAA-----TTTTTTCCTG--AAATTCCACGT-AAG-AA--T-------CT---GGACTG-AA-

TTTGGG-AC----------AAAACGCC-AC-AA-AT--TTCACG--TC------------------------

-----------------------------------------AAAA-G--GATGAG---TATTCAC-------

---CTA-----GGAAAAAAG---TC-AAACAGTT-TC--------------------------ACA-CAC--

AAAC--G-AA-CCT------------TCC--T-TTC-------A-A--CC-C-T-GA-C---------A--G

TGATG-AAT-A--A-A-AA--A--T-TG-TTGAA-AATCTTGT-GGGACGAA----TCT------GGG----

-------------GCTCAAATCTCAG-C----------------CAAAA--GTCAATAAACACA---A----

------------------TGA--C----GAA-TGTCTGG----------T--A-AAATTTTC-AAA-CCA--

--AAAGACCC-------AATGA-AT-----AGAGC---GT-----AGTA------AG-ACCTAGA-CCGAGA

GTGAA-------------------------------------------------------------------

------------------------------------------------------------------------

--------------T--AAAACC--------------------------GG-----------------TTTT

C----------------------CGAAAATAAAA--T--GTCCTGGC-----TTTTCTTCCC----------

--------------TATATC--TCCT-----------TTTTGTGA---------------------------

-------------------------TTT--GTTTAT-GGAC--GTG-CGC-CC-TTCGCA--GGGTGC--AA

AC-----------AGCAC-A----TGAAAGT-ACTTGTG-T-------------GAAT--------------

----ATTTCA------------------------------ACGCAAT-TTTT-CAA-CAC-AGA-----AT-

AAAATT--GTAGAAA--GTAGT------------GCGAAATTT--AACA-A-ATT-TTAC--TAGAT-----

---------GAT-AGCCT-------------------TGGTTTTTCACA----AAATTT-----------

>Pv02Sk00100/1-530 Pv02Sk00100 undefined product 781063:781592 forward

-TCCCGGAATAA-----TTTTTTCCTG--AAATTCCACGT-AAG-AA--T-------TT---GGACTG-AA-

TTTGGG-AC----------AAAACACC-AC-AA-AT--TTCACG--AC------------------------

-----------------------------------------AAAC-G--GATGAG---TATTCAC-------

---CTA-----GGAAAAAAG---TC-AAACAGTT-TC--------------------------ACA-CAC--

AAAA--G-AA-CCT------------TCC--T-TTC-------A-A--CC-C-T-GA-C---------A--G

TGATG-AAT-A--A-A-AA--A-TT-TG-TTGAA-AATATTGT-GGGACGAA----TCT------GGA----

-------------GCTAAAATCTCAG-C----------------CAAAA--CTCAATACACACA---A----

------------------TGA--C----GAA-TGTTTGG----------T--A-AATTTTTC-AGA-CCA--

--AAAGATTC-------AAGGA-AT-----AGGGC---GT-----ACTT------AG-ACCCAGA-TCGAGA

GTGAA-------------------------------------------------------------------

------------------------------------------------------------------------

--------------C--AAAACC--------------------------GG-----------------TTTT

C----------------------CGAAAACAAAA--T--GTCTTGAC-----TTTTCTTCCC----------

--------------TATATC--TCAT-----------TTTTGTGA---------------------------

-------------------------TTT--TTTTAC-GGAC--GTG-CCT-CC-TTCGCA--GGGTGC--AA

AC-----------AACAC-T----TGAAAGT-ACTTGTG-T-------------GAAT--------------

----ATTTCA------------------------------ACGCAAA-TTTT-TAA-TAC-AGA-----AT-

AAAATT--GTAGAAA--GTAAG------------GCGAAATTT--AAAA-G-ATT-TTGC--TAGAG-----

---------GAT-AGCTT-------------------TGGTTTTTCCCA----AAATTT-----------

>Pv02Sk00110/1-530 Pv02Sk00110 undefined product 781594:782123 forward

-TCCCGGAATAA-----TTTTTTCCTG--ACATTCCACGT-AAG-AA--T-------CT---GGACTG-AA-

TTTGGG-AC----------AAAACGCA-AC-AT-TT--TTCACG--TC------------------------

-----------------------------------------AAAC-G--GATGAG---TATTCAC-------

---TTA-----GGAAAAAAG---TC-AAACAGTT-TC--------------------------ACA-CAC--

AAAC--G-AA-CAT------------TCC--T-TTC-------A-A--CT-C-T-GA-C---------A--G

TGATG-AAT-A--A-A-AA--A-TT-TG-TTGAA-AATCTTGT-GGGACGAA----TCA------TTG----

-------------GCTGAAATCTCAG-C----------------CAAAC--CTCAATACACACA---A----

------------------TGA--C----GAA-TGTATGG----------T--A-AAACTTTC-AGA-CCA--

--AAAGACTC-------AAGGA-AT-----AGGGA---GT-----AGTC------AG-ACCAAGA-CCGAGA

GTGAA-------------------------------------------------------------------

------------------------------------------------------------------------

--------------C--AAAACC--------------------------GG-----------------TTTT

C----------------------TGAAAACAAAA--T--GTCCTGAC-----TTTTCTTCCC----------

--------------TATATC--TCCT-----------TTTTGTGA---------------------------

-------------------------TTT--GTTTAT-GGAC--GTG-CCT-AC-TTCGCA--GGGTGC--AA

AC-----------AGCAC-A----TGAACGT-ACCTGTA-T-------------GAAT--------------

----TTGTCA------------------------------ACGCAGT-TTTT-CAA-CAC-AGA-----AT-

AAAATT--GCAGAAA--GTTGG------------GTGAAATTT--CACA-A-ATT-CTGC--TAGAG-----

---------GAT-AGCCT-------------------TGGTCTTTCACA----AAATTT-----------

>Pv02Sk00120/1-532 Pv02Sk00120 undefined product 782125:782656 forward

-TCCCGGAATAA-----TTTTTTTCTG--AAATTCTACGT-AAG-AA--G-------CT---GGACTG-AA-

TTTGGG-AC----------AAAACTCC-AC-AA-AT--TTCACG--TC------------------------

-----------------------------------------AAAC-G--GATGAG---TATTCAC-------

---CTA-----GGAAAAAAG---TC-AAACAGTT-TC--------------------------ACA-CAC--

AAAC--G-AA-CCT------------TCC--T-TTC-------A-A--CC-C-T-GG-C---------A--G

TGATG-AAT-A--A-A-AA--A-TT-TG-TTGAA-AATCTTGT-GGGACGAA----ACT------GGA----

-------------CCTCAAATCTCAG-C----------------CAAAA--CTCAATACACACA---A----

------------------TGA--A----GAA-TGTCTGG----------T--A-AAATTTTC-AGA-CCA--

--AAAGACCA-------AAGGA-AT-----ATGGC---GT-----AGTA------AG-ACCCAGA-CCGAGA

GTGAA-------------------------------------------------------------------

------------------------------------------------------------------------

--------------C--AAAATC--------------------------GG-----------------TTTT

C----------------------CCAAAACAAAA--T--GTCCTCGT-----TTTTCTTCAC----------

--------------TATATC--TCCT-----------TTTTGTGA---------------------------

-------------------------TTT--GTTTAT-GGAC--GTG-CCT-CC-TTCGCT--GGGTGC--AA

AC-----------AGCAC-A----TGAAAGT-AGTTGTG-T-------------GAAT--------------

----TTTTCA------------------------------ACGCAAT-TTTT-CAA-CAT-AGA-----AT-

AAAATT--GCAGAAA--GTAAG------------GCGAAATTT--CACA-A-ATT-TTGC--TAGAG-----

---------GAT-AGCAT-------------------TGGTTTTTCAAA----AAATTTCT---------

>Pv02Sk00130/1-533 Pv02Sk00130 undefined product 789752:790284 forward

-TCCCGGAATAA-----TTTTTTTCTG--AAATTCTACGT-AAG-AA--G-------CT---GGACTG-AA-

TTTGGG-AC----------AAAACTCC-AC-AA-AT--TTCACG--TC------------------------

-----------------------------------------AAAC-G--GATGAG---TATTCAC-------

---CTA-----GGAAAAAAG---TC-AAACAGTT-TC--------------------------ACA-CAC--

AAAC--G-AA-CCT------------TCC--T-TTC-------A-A--CC-C-T-GG-C---------A--G

TGATG-AAT-A--A-A-AA--A-TT-TG-TTGAA-AATCTTGT-GGGACGAA----TCT------GGG----

--------------CTCAAATCTCAG-C----------------CAAAA--CTCAATACACACA---A----

------------------TGA--A----GAA-TGTCTGG----------T--A-AAATTTTC-AGA-CCA--

--AAAGACCA-------AAGGA-AT-----ATGGC---GT-----AGTA------AG-ACCCAGA-CCGAGA

GTGAA-------------------------------------------------------------------

------------------------------------------------------------------------

--------------C--ATAATC--------------------------GG-----------------TTTT

C----------------------CGAAAACAAAA--T--GTCCTGGC-----TTTTCTTCCC----------

--------------TCTATC--TCAT-----------TTTTTTGA---------------------------

-------------------------TTT--GTTTAT-GGAC--GTG-CCT-CC-TTCGCT--GGGTGC--AA

AC-----------AGCAC-A----TGAAAGT-AGTTGTG-T-------------GAAT--------------

----TTTTCA------------------------------ACGCAAT-TTTT-CAA-CTC-AGA-----AT-

AAAATT--GCAGCAA--GTAGG------------GCGAAATTT--CACA-A-ATT-TTTC--TAGAG-----

---------G-------T-------------------TGGTTTTTCACA----AAATTTCAGAAGAATT-

>Pv02Sk00140/1-515 Pv02Sk00140 undefined product 790287:790801 forward

--CCGGGAATAA-----GTTTTTCCTG--AAATTCCACGT-AAG-AA--C-------AT---GGACTT-AA-

TTTGGG-AC----------AAAACGCC-AA-AA-AT--TTCACG--TC------------------------

-----------------------------------------TAAC-G--GATGAG---TATTCAC-------

---CTA-----GAAACAAAG---TC-AAACAGTT-CA--------------------------CAC-ACA--

AACG--A-AC-TTT------------CCT--T-TCA-------A-C--CC-C-T-GG-C---------A--G

TGATG-AAT-T--A-A-AA--A-TT-TG-TTGAA-AATTTTGT-GGGAGGAA----TCT------GGG----

-------------GCTCAAATCTCAG-C----------------CAAAA--GTCAATACACACA---A----

------------------TGA--A----GAA-TGTCTGG----------T--A-AAATTTTC-AGA-CCA--

--AAAGACTC-------AAGTA-AT-----A-GGC---GT-----AGTA------AG-ACCCAGA-CCGAGA

GTGAA-------------------------------------------------------------------

------------------------------------------------------------------------

--------------C--AAAACC--------------------------GG-----------------TTTT

T----------------------CGAAAACAAAA--T--GTCCTGGC-----TTTTCTTCCC----------

--------------TATATC--TCCT-----------TTTTGTGA---------------------------

-------------------------TTT--GTTTAT-GGAC--GTG-CCT-CC-TTCGCA--GGGTGC--AA

AC-----------AGCAC-A----TGAAAGT-ACTTGTG-T-------------G-----------------

--------------------------------------------AAT-TTTT-CAA-CAC-AGA-----AT-

AAAATT--TCAGAAA--GTAGG------------GCGAAATTT--CTCA-A-ATT-TTGC--TAGAG-----

---------GAT-AGCCT-------------------TGGTTTTTCATA----AAATTT-----------

>Pv02Sk00150/1-530 Pv02Sk00150 undefined product 790803:791332 forward

-TCCCAGAATAA-----TTTTTTCCTG--AAATTCCACAT-AAG-TA--T-------CT---GGACTG-AA-

TTTGGG-AT----------AAAACGCC-AC-AA-AT--TTCACG--TC------------------------

-----------------------------------------AAAC-G--GATGAG---TATTCAC-------

---CTA-----GGAAAAAAG---TC-AAACAGTT-TC--------------------------ACA-CAC--

AAAC--G-AA-CCT------------TCC--T-TTC-------A-A--CC-C-T-AG-C---------A--A

TGATG-AAT-A--A-A-AA--G-TT-TG-TTGAA-AATCTTGT-GGGCCGAA----TCT------GGG----

-------------GCTCAAATCTCAG-G----------------GAAAA--CTCAATACACACA---A----

------------------TGA--G----GAA-TGTCTGG----------T--A-AAGTTTTC-GGA-CCA--

--AAAGACCC-------AAGGA-AT-----AGGAA---GT-----TGTA------CG-ACCTAGA-CCGAGA

GTAAA-------------------------------------------------------------------

------------------------------------------------------------------------

--------------C--AAAATC--------------------------CA-----------------TTTT

C----------------------CGAAAACAAAA--T--GTCCTGGC-----TTTTCTTCAC----------

--------------TATATC--TCCT-----------TTTTGTGA---------------------------

-------------------------TTT--GTTTAT-GGAC--GTG-CCT-CC-TTCGCA--GGGTGC--AA

AA-----------AGCAC-A----TGAAAGC-ACTTATG-T-------------GAAT--------------

----TTTTCA------------------------------ACGCAAT-TTTT-CAA-CAC-AGA-----AT-

AAAATT--GCAGAAA--CTAGG------------GAGAAATTT--CACT-A-ATT-TTGC--TAGAG-----

---------GAT-AGCCT-------------------TGGTTTTTCACA----AAATTT-----------

>Pv02Sk00160/1-528 Pv02Sk00160 undefined product 791334:791861 forward

-TCCCGAAATAA-----TTTTTTCCTG--AAATTCCACGT-AAG-AA--T-------TT---GGACTG-AA-

TTTGGG-AC----------AAAACACC-AC-AA-AT--TTCACG--TC------------------------

-----------------------------------------AAAC-G--GATGAG---TACTCAC-------

---CTA-----GGAAAAAAG---TG-AAACAGTT-TC--------------------------ACA-CAC--

AAAC--T-AA-CCT------------TCC--T-TTC-------A-A--CC-C-T-GA-C---------C--G

TGATG-AAT-A--A-A-AA--A-TT-TG-TTAAA-AATCTTGT-GGGACTAA----TGT------GGA----

-------------GCTAAAATCTCAG-C----------------CAAAA--CTCAATACACACA---A----

------------------TGA--C----GAA-TGTCTGG----------T--A-AAATTTTC-AGA-CTA--

--AAAGACAC-------AAGGA-AT-----AGGGC---GT-----AGTT------AG-ACCCAGA-CCGAGA

GTGAA-------------------------------------------------------------------

------------------------------------------------------------------------

--------------C--AAAACC--------------------------GG-----------------TTTT

C----------------------CGAAAA-AAAA--T--GTTCTGGC-----TTTTCTTCCC----------

--------------TATATC--TCCT-----------TTTTGTGA---------------------------

-------------------------TTT--GTTTAT-GGAC--GTG-CCT-CC-TTCGCA--GGGTAA--AA

AC-----------AGCAC-A----TGAAAGT-ACTTGTG-T-------------GAAT--------------

----TTTTCA------------------------------ACGCAAT-TTAT-CAA-CAC-AGA-----AC-

AAAATT--GCAGAAA--GTTGG------------GGGAAATTT--CACA-A-ATT-TTGC--TAGTG-----

---------GAT-AGTCT-------------------TGGATTTTCACA----AATTT------------

>Pv02Sk00170/1-528 Pv02Sk00170 undefined product 791864:792391 forward

--CCCGGAATAA-----TTTTTTCCTG--AAATTCCACGT-AAG-AA--T-------CT---GGACTG-AA-

TTTGGG-AC----------AAAACGCC-AC-AA-AT--TTCACG--TC------------------------

-----------------------------------------AAAT-G--GATGAG---TATTCAC-------

---CTA-----GGAAAAAAG---TC-AAATAGTT-TC--------------------------ACA-CAC--

A--C--G-AA-CAT------------TCC--T-TTT-------A-A--CC-C-T-GA-T---------A--G

TGATG-AAT-A--A-A-AA--A-TT-TA-TTGAA-AATCTTAT-GGGACGAA----TCT------GGG----

-------------GCTCAAATCTCAG-C----------------CAAAA--CTCAATACACACA---A----

------------------TGA--G----GAA-TGTCTGG----------T--A-AAATTTTT-AGA-CCA--

--AAAGATCC-------AAGGA-AT-----AGGGC---AT-----AGTA------AG-ACCCAGA-CCAAGA

GTGAA-------------------------------------------------------------------

------------------------------------------------------------------------

--------------C--AAAACC--------------------------GA-----------------TTTT

C----------------------CGAAAACAAAA--T--GTTCTATA-----TTTTCTTCCC----------

--------------TATATC--TCAT-----------TTTTGTGA---------------------------

-------------------------TTT--GTTTAT-GGAC--GTG-CCT-CC-TTCGCA--GGGTTG-CAA

AC-----------AGCAC-A----TGAAAGT-ACTTGTG-T-------------GATT--------------

----TTTTCA------------------------------ACGCAAT-TTTT-CAA-CAC-ACA-----AT-

AAAATT--GCAGATA--GTGGG------------GCAAAATTT--CACA-A-ATT-TTGC--TAGAG-----

---------GAT-AGCCT-------------------TGGTTTTTCACA----AAATTT-----------

>Pv02Sk00180/1-529 Pv02Sk00180 undefined product 792393:792921 forward

-TCCTGGAATAA-----TTTTTTCCAG--AAATTCCAAGT-AAG-AA--A-------CT---GGACTG-AA-

TTCGGG-AC----------AAAATGCC-AC-AA-AT--TTCACG--TC------------------------

-----------------------------------------AAAC-G--GATGAG---TATTCAC-------

---CTA-----GGAAAAAAG---TC-AAACAGTT-TC--------------------------ACA-CAC--

AAAC--G-AA-CCT------------TCC--T-TTC-------A-A--CC-C-T-GG-C---------A--G

TTATG-AAT-A--A-A-TT--T-TT-TT-TTGAA-AATCTTGT-GGGATGAA----TCT------GGG----

-------------TCTCAAATCTCAG-C----------------CAAAA--CTCAAGACACACA---A----

------------------TGA--G----GAA-TGTCTGG----------T--A-AAATTTTT-AGA-CCA--

--AAAGACCC-------AAGGA-AT-----AGGGC---AT-----AGTA------AA-ACGCAGT-CCGAGA

GTGAA-------------------------------------------------------------------

------------------------------------------------------------------------

--------------C--AAAACT--------------------------GG-----------------TTTT

C----------------------CGAAAACAAAA--T--GTTCTGGC-----TTTTCTTCCC----------

--------------TATATC--TCCT-----------TTTTGTGA---------------------------

-------------------------TTT--GTTTAT-GAAC--GTG-CCT-CC-TTCGCA--GGGTGC--AA

AC-----------AGCAC-A----TTAAAGT-ACTTGTG-T-------------GAAT--------------

-----TTTCA------------------------------ACGCAAT-TTTT-CAA-CAC-AGA-----AT-

AAAATT--GCAGAAA--GTAGG------------GCGAAATTT--CACA-A-ATT-TTGC--TAGAA-----

---------GAT-AGTCT-------------------TGGTTTTTCAAA----AAATTT-----------

>Pv02Sk00190/1-529 Pv02Sk00190 undefined product 792923:793451 forward

-TCTCGGAATAA-----TTTTTTCTTG--AAATTCCACGT-AAA-AA--T-------CT---GGACTG-AA-

-TTGAA-AC----------AAAACGCC-AC-AA-AT--TTCACG--TC------------------------

-----------------------------------------AAAC-G--GATGAG---TATTCAC-------

---CTA-----GGACAAAAG---TG-AAACAGTT-TC--------------------------ACA-CAA--

AAAC--G-AA-CCT------------TCC--T-TTA-------A-A--CC-C-T-AG-C---------A--G

TGATG-AAT-A--A-A-AA--A-TT-TG-TTAAA-AATCTTGT-GGGACGAA----TCT------GGG----

-------------GCTCAAATCTCAT-C----------------CAAAA--TTTAATACACACA---A----

------------------TGA--C----GAA-TGTCTGG----------T--A-AAATTTTC-AGA-CCA--

--AAAGACCC-------AAGGA-AT-----AGGGC---GT-----AGTA------AG-ACCTAGA-CCGAGA

GTGAA-------------------------------------------------------------------

------------------------------------------------------------------------

--------------C--AAAATT--------------------------GG-----------------TTTT

C----------------------CGAAAATAAAA--T--GTCCTGGC-----CTTTCTTCCC----------

--------------TATATC--TCAG-----------TTTTTTGA---------------------------

-------------------------TTT--GTTTAT-GGAC--GTG-CCT-CC-TTCGCA--GGGTGC--AA

AC-----------AGCAC-A----TGAAAGT-ACTTGTG-T-------------GAAT--------------

----TTTTCA------------------------------ACGCAAT-TTTT-CAA-CAC-AGA-----AT-

AAAATT--GCAGAAA--GTAGG------------GCGTAATTT--CACA-A-ATT-TTGA--TAGAG-----

---------GAT-AGCCT-------------------TGGTTCTTCACA----AAATTT-----------

>Pv02Sk00200/1-531 Pv02Sk00200 undefined product 793453:793983 forward

-TCCCGGAATAA-----TTTTTTCCTG--AAATTGCACGT-AAG-AA--T-------CT---GGACTG-AA-

TTTGGG-AC----------AAAACGCC-AC-AA-AT--TTCACG--TC------------------------

-----------------------------------------AAAC-G--GATGAG---TATTCTC-------

---TTA-----AGAAAAAAG---TC-AAACAGTT-TC--------------------------ACA-CAC--

AAAC--G-AA-CCT------------TCC--T-TTC-------A-A--CC-C-T-GG-C---------A--G

TGATG-AAT-A--A-A-AA--A-TT-TG-TTGAA-AATCTTGT-GGGACAAA----TCT------GAT----

-------------GCTCAAATCTCAG-C----------------CAAAA--CTCTATACACACA---A----

------------------TGA--C----GAA-TGTCTGG----------T--A-AAACTTTC-AGA-CCA--

--AAAGACAC-------AAGGA-AT-----AGGGC---GT-----AGTA------AG-ACCTAGA-CCGAGA

GTGAA-------------------------------------------------------------------

------------------------------------------------------------------------

--------------C--AAAACC--------------------------GG-----------------TTTT

C----------------------CGAAAACAGAA--T--GTCCTGGC-----TTTTCTTCCC----------

--------------TATATC--TTCT-----------TTTTGTGA---------------------------

-------------------------TTT--GTTTAT-GGAC--GTG-CCT-TT-TTCGCA--GGGTGC--AA

AT-----------AGCAC-A----TGAAAGT-ACTTGTG-T-------------GAAT--------------

----TTTTCA------------------------------ACGCAAT-TTTT-CAA-CAC-AGA-----AT-

AAAATT--GTAGAAA--ATAGG------------GCGAAATTT--CACA-A-ATT-TTGC--TAGAG-----

---------GAT-AGCTT-------------------TGGTTTTTCACA----AAATTTG----------

>Pv02Sk00210/1-532 Pv02Sk00210 undefined product 793984:794515 forward

-TCCCGGAATAA---TTTTTTTTTCTG--AAATTCCACGT-AAG-AA--T-------TT---GGACTG-AA-

TTTGGG-AC----------AAAACGCC-AC-AA-AT--TTCATG--TC------------------------

-----------------------------------------AAAC-G--GATGAG---TATTCAC-------

---CTA-----GGAAAAAAG---TC-GAACAGTT-TC--------------------------ACA-CAC--

ATAC--G-AA-CCT------------TCC--T-TTC-------A-A--CC-C-T-GC-C---------A--G

TGATG-AAT-A--A-A-AA--A-TT-TG-TTGAA-AATCTTGT-GGGACGAA----TCT------GGG----

-------------GCTCAAATCTCAA-C----------------CAAAA--TTCAATACACACA---A----

------------------TGA--G----GAA-TGTCTGG----------T--A-AAATTTTC-AGA-TCA--

--AAAGACTC-------AAGGA-AT-----AGGGC---GT-----AGTA------AG-ACGCAGA-CCGAGA

GTGAA-------------------------------------------------------------------

------------------------------------------------------------------------

--------------C--AAAACC--------------------------GG-----------------TTTT

C----------------------CGAAAACAAAA--T--GTCCTGGC-----TTTTCTTCCC----------

--------------TATATC--TTCT-----------TTTTGTGA---------------------------

-------------------------TTT--GTTTAT-GGAC--GTG-CCT-TT-TTCGCA--TAGTGC--AA

AC-----------AGCAC-A----TGAAAGT-ACTTGTG-T-------------GAAT--------------

----TTTTCA------------------------------ATGCAAT-TTTT-CAA-CAC-AGA-----AC-

AAAATT--GCAGAAA--GTAGG------------GTGAAATTT--CACA-A-TTT-TTTC--TAGAG-----

---------GAT-AGCCT-------------------TGGTTTTTTACA----AAATTT-----------

>Pv02Sk00220/1-529 Pv02Sk00220 undefined product 794517:795045 forward

-TCCCGGAATA------ATTTTTCCTG--AAATTCCACGT-AAG-AA--T-------CT---GGATTG-AA-

TTTTGG-AC----------AAACAACC-AC-AA-AT--TTCACG--TC------------------------

-----------------------------------------AAAC-G--GATGAG---TATTCAC-------

---AAA-----AGAAAAAAG---TC-AAACAGTT-TC--------------------------ACA-TAC--

AAAC--G-AA-CCT------------TCC--T-TTC-------A-A--CT-C-T-GG-C---------A--G

TGATG-AAG-A--A-T-AA--A-TT-TG-TTGAA-AATCTTGT-GGGACGAA----TCT------GGG----

-------------GCTCAAATCTCTT-C----------------CAAAA--CTCAATACACACA---A----

------------------TGA--C----GAA-TGTCTGG----------T--A-AAATTTTT-AGA-CCA--

--AAAGACCC-------AAGGA-AT-----AGGGC---GT-----AGTA------AG-ACCCAGA-CCGAGA

GTGAA-------------------------------------------------------------------

------------------------------------------------------------------------

--------------C--AAAACC--------------------------GA-----------------TTTT

C----------------------CGATAACAAAA--T--GTCCTGGC-----TTTTCTTCCT----------

--------------TATATC--TCCT-----------TTTTGTGA---------------------------

-------------------------TTT--TTTTAT-GGAC--GTG-TCT-CC-TTCGCA--GAGTGC--AA

AC-----------AGCAC-A----TGAAAGT-ACTTGTG-T-------------GAAT--------------

----TTTTCA------------------------------ACGCAAT-TTTT-CAA-CAC-AAA-----AT-

AAAATT--GCAGAAA--GTAGG------------GCGAAATTT--CACA-A-ATT-TTGC--TAGAG-----

---------GAA-AGCCT-------------------TGGTTTTTCACA----AAATTT-----------

>Pv02Sk00230/1-530 Pv02Sk00230 undefined product 795047:795576 forward

-TCCTGGAATAA-----TTTTTTCCTG--AAATTCCACGT-AAG-TA--T-------CT---GGACTG-AA-

TTTGGG-AC----------AAAACGCC-AC-AA-AT--TTCACG--TC------------------------

-----------------------------------------AAAC-G--CATGAG---TATTCAC-------

---CTA-----CGAAAAAAG---TC-AAACAGTT-TC--------------------------ACA-CAC--

ATAC--A-AA-CCT------------TCC--T-TTC-------A-A--CC-C-T-GG-C---------A--G

TGATG-AAT-A--A-A-AA--A-TT-TG-TAGAA-AATCTTGT-GGGACGAA----TCT------GGG----

-------------GCTCAAATCTCTT-C----------------CAAAA--CTCAATACACACA---A----

------------------TGA--C----GAA-TGTCTGG----------T--A-AAATTTTC-AGA-CCA--

--AAAGACCC-------AAGGA-AT-----AGGAC---GT-----AGTA------AG-ACTCAGA-CCAAGA

GTGAA-------------------------------------------------------------------

------------------------------------------------------------------------

--------------C--AAAACC--------------------------GG-----------------TTTT

T----------------------CGAAAACAAAA--T--GTCCTGAC-----TTTTCTTCCC----------

--------------TATATC--TCCT-----------TTTTGTGA---------------------------

-------------------------TTT--GTTTAT-GGAT--GTG-CCT-CC-TTCGCA--GGTTGC--AA

AC-----------AGCAC-A----TGAAAGT-ACTTGTG-T-------------GAAT--------------

----TTTTCA------------------------------AAGCAAT-ATTT-CAA-AAC-AGA-----AT-

AAAATT--GCATAAA--GTAGG------------GCGAAATTT--CACA-A-ATT-TTGC--TAGAG-----

---------GAT-AGCCT-------------------TGGTTTTTCAAA----AAATTT-----------

>Pv02Sk00240/1-529 Pv02Sk00240 undefined product 795578:796106 forward

-TCCCGGAATAC-----ATTTTTCCTG--GAATTCCACTT-AAT-GA--T-------CT---GGACTG-AA-

-TTTGG-AC----------AAAACGCC-AC-AA-AT--TTCACG--TG------------------------

-----------------------------------------AAAC-G--GATGAG---TTTTCAC-------

---CTA-----GGAAAAAAG---TC-AAACAGTT-TC--------------------------ACA-CAC--

ATAC--A-AA-TCT------------TCC--T-TTC-------A-A--CC-C-T-GG-C---------A--G

TGATG-AAT-A--A-A-AA--T-GT-TG-TAGAA-AATCTTGT-GGGACGAA----TCT------GGG----

-------------GCTCAAATCTCAG-C----------------CAAAA--CTCAATACACACA---A----

------------------TGA--C----GAA-TGTCTGG----------T--A-AAATTTTT-AGA-CCA--

--AAAGACCC-------AAGGA-AT-----AGGGC---GT-----AGTA------AG-ACCCAGA-CCGAGA

GTGAA-------------------------------------------------------------------

------------------------------------------------------------------------

--------------C--AAAACC--------------------------GA-----------------TTTT

C----------------------CGATAACAAAA--T--GTCCTGAC-----TTTTCTTCCC----------

--------------TATATC--TCCT-----------TTTTGTGA---------------------------

-------------------------TTT--TTTTAT-GGAC--GTG-TCT-CC-TTTGCA--GAGTGC--AA

AC-----------AGCAC-A----TGAAAGT-ACTTGTG-T-------------GAAT--------------

----TTTTCA------------------------------ACGCAAT-TTTT-CAA-CAC-AAA-----AT-

AAAATT--GCAGAAA--GTAGG------------GCGAAATTT--CACA-A-ATT-TTGC--TAGAG-----

---------GAA-AACCT-------------------TGGTTTTTCACA----AAATTT-----------

>Pv02Sk00320/1-541 Pv02Sk00320 undefined product 1587204:1587744 forward

CTCCCGGAATAA-----TATTTTCCTG--AAATTCCACGT-AAG-AA--T-------AT---GGACTG-AA-

TTTGGG-AC----------AAAACGCG-AC-AA-AT--TTCACG--TC------------------------

-----------------------------------------AAAC-G--GATGAG---TATTTAC-------

---CTA-----GGAAAAAGA---TC-AAACAGTT-TC--------------------------ACA-CAC--

AAGC--G-GA-CCT------------TCC--T-TTC-------A-A--CC-C-T-GG-C---------T--G

TGATG-AAT-A--A-A-AA--A-TT-TA-TTGAA-ATTCTTGT-GGGACGAA----TCT------GGG----

-------------GCTCAAATCTCAG-C----------------CAAAA--TTCAATACACACA---A----

------------------TGA--C----GAA-TGTCTGG----------T--A-AAATTTTC-AGA-TGA--

--AAAGACCA-------AAGGA-AT-----AGGGC---GT-----AGTA------AG-ACCCAGA-CCAAGA

GTGAA-------------------------------------------------------------------

------------------------------------------------------------------------

--------------C--AAAACC--------------------------GG-----------------TTTT

C----------------------CGAAAACAAAA--T--GTCCTGGC-----TTTTCTTTCC----------

--------------TATATT--TCCT-----------TTTTGTGA---------------------------

-------------------------TTT--GTTTAT-GGAT--GTG-CCT-CC-TTTGCA--GGGTCC--AA

AC-----------AGCAC-A----TGAAAGT-ACTTGTA-T-------------GAAT--------------

----GTTTCA------------------------------ACGCAAT-TTTT-CAA-CAC-AAA-----AT-

AAAATT--GGAGAAA--GTAGG------------GCGAAATTT--CACA-A-ATT-TTGC--TAGAT-----

---------GAT-AGCCT-------------------TGGTTTTTCATA----AAATTTCAGAAAAATT-

>Pv02Sk00340/1-530 Pv02Sk00340 undefined product 2403286:2403815 forward

CTCCCGGAATAG-----TCTTTTCCTG--AAATTCCACCC-AAG-AA--T-------CT---CCACTG-AA-

TTGGG--AC----------AAAACGCG-AG-AA-AT--TTCAGG--TC------------------------

-----------------------------------------ACAC-G--GATGAG---TATTCAC-------

---CCA-----CG-AAAAAAA--TC-AAAC---T-TC--------------------------ACA-CAG--

AAAG--G-AA-CCT------------TCA--T-TTC-------A-G--CC-C-T-CG-C---------A--G

TGACG-AAT-A--A-A-AA--A-TT-TA-TTGCC-AATCTTGT-GGGACGAA----TGG------GGA----

-------------GCTCAAACATCAG-C----------------CAAAA--CCTAATAGACACA---G----

------------------TGA--C----GAA-TGTCTAG----------T----AAAATTTC-AGA-CCA--

--AAATACCC-------AATGA-GT-----AAGGC---GT-----AGTG------AG-TCCCAGA-CCGAGA

GTGAA-------------------------------------------------------------------

------------------------------------------------------------------------

--------------C--AAAACC--------------------------GG-----------------TTTT

C----------------------CGAAAACAAAA--C--GTCCTGGC-----TTTTCTTCCC----------

--------------CGTATC--TTCT-----------TTTTGTGA---------------------------

-------------------------TTC--GTTTAT-GGAC--GTG-TCT-CC-TAGCCT--AGGTGG--AA

AC-----------AACAT-A----CGAAAGT-GCTTGTG-T-------------GAATTT------------

----TTTTCA------------------------------GCACAAT-A-C-G-GA-CCC-AGA-----AT-

GTAATT--GCAGAAA--TTAGG------------CCGGAATTT--TACA-A-GTT-TCGG------------

----------TT-CGTCT-------------------TGG-TTTGCACA----AAATTTCTATAAAATTC

>Pv02Sk00350/1-539 Pv02Sk00350 undefined product 2403816:2404354 forward

-TCGCGGAATAG-----TTTTTTCCTG--AAATTCTACCC-AAG-AA--T-------CT---CCACTG-AA-

TTTGGG-AC----------AAAACGCG-AC-AA-AT--TTCAAG--TC------------------------

-----------------------------------------AAAC-G--AATGAG---TATTCAC-------

---CCA-----CGAAAAAAA---TC-AAATAGTT-TC--------------------------ACA-CAC--

AAAC--G-AA-ACT------------TCC--T-TTC-------A-G--CC-C-T-GG-C---------A--G

TGACG-AAT-A--A-A-A---A-TT-TA-TTGCC-AATCTTGT-GGGACGAA----TCT------GGG----

-------------GCTCAAACCTCAG-C----------------CAAAA--CTCAATAGACACA---G----

------------------TGA--C----GAA-TGTCTGG----------T--A-AAAATTTT-AGA-CCA--

--AAATACCT-------AAGGA-GA-----AAGGC---GT-----AGTG------AG-TCCTAGA-TCGAGA

GTGAA-------------------------------------------------------------------

------------------------------------------------------------------------

--------------C--AAAACC--------------------------GA-----------------TTTT

C----------------------AGAAAACAAAA--C--GTCCTAAA-----TTTTCTTCCC----------

--------------CGTATC--TTCT-----------TTTTGTGA---------------------------

-------------------------TTC--GTTTAT-GGAC--GTG-CCT-CC-TTGCCT--GGGTGC--AA

AC-----------AACAT-A----CGAAAGT-GCTTGTG-T-------------AAATTT------------

----TTTTCA------------------------------GCGAAAT-A-C-G-GA-CTC-AGA-----AT-

GAAATT--GCAGAAA--TTAGG------------CTAGAATTT--CACA-G-CTT-TCGG--TAGAG-----

---------GAT-AGCCT-------------------TGG-TTTGCACA----AAATTTCTGAAAAATTC

>Pv02Sk00360/1-526 Pv02Sk00360 undefined product 2404355:2404880 forward

-TCGCAGAATAT-----TTTTTTCCGG--AAATTCCACCC-AAA-AA--T-------CT---CCACTG-AA-

TTAGGG-GC----------AAAACGTG-AC-AA-AT--TTCAGG--TC------------------------

-----------------------------------------CAAC-G--GATGAG---TATTCAC-------

---CCA-----CGAAAAAAAA--TC-AAAC---T-TC--------------------------ACA-CAT--

AAAC--G-AA-CCT------------TCC--T-TTC-------A-G--CC-C-T-AA-C---------A--G

TGACG-AAT-A--A-A-AA--A-TT-TA-TTGCC-AATCTTGT-GGGAAGAA----TGG------GGG----

-------------GCTCAAACATCAG-C----------------CAAAA--CTCAATAGACACA---G----

------------------TTA--C----GAA-TGTCTAG----------T----AAAATTTC-AGA-CCA--

--AAATACCC-------AATGA-GT-----AAGGC---GT-----AGTG------AG-TCCCAGA-CTAAGA

GTAAA-------------------------------------------------------------------

------------------------------------------------------------------------

--------------C--AAAACC--------------------------GG-----------------TTTT

C----------------------CGAAAACAAAA--C--GTCCTGGC-----TTTTCTTCCC----------

--------------CGTATC--TTCT-----------TTTTGTGA---------------------------

-------------------------TTC--GTTTAT-GGAC--TTG-CCT-CC-TTGCCT--GGGTGC--AA

AC-----------AACAT-A----TGAAAGT-GCTTGTG-T-------------GAAATT------------

----TTTTCA------------------------------ACGCAAT-A-T-G-GA-CCC-ATA-----AT-

GAAAAT--GCAGAAA--TTAGG------------TCGGAATTT--CACA-A-GT------------------

----------TT-CGCCT-------------------TGG-TTTGCACA----AAAGTTCTGATAAATTC

>Pv02Sk00370/1-512 Pv02Sk00370 undefined product 2404880:2405391 forward

CTCCCGGAATAG-----TTTTTTCCTG--AAATTCCACCC-AAG-AA--T-------CT---CCACTG-AA-

TGTGGG-AC----------GAAACGCG-A-------------------------------------------

----------------------------------------------G--GATGAG---TATTCAC-------

---CCA-----CGAAAAAAG---TC-AAACAGTT-TC--------------------------ACA-CAC--

AAAC--G-AA-CCT------------TCC--T-TTC-------A-G--CC-C-T-GG-C---------A--G

TGACG-AAT-A--A-A-AA--A-TT-TA-TTGCC-AATCTTGT-GGCACGAA----TCT------GGG----

-------------GCTCAAACCTCAG-T----------------CAAAA--CTCAATAGACACA---G----

------------------TGA--C----GAA-TTTCTGG----------T--A-AAAATTTC-ATA-CCA--

--AAATACCC-------AAGGA-GT-----AAGGC---GT-----AGTG------AG-TCGCAGA-CCGAGA

GTGAA-------------------------------------------------------------------

------------------------------------------------------------------------

--------------C--AAAATC--------------------------GG-----------------TTTT

C----------------------CTAAAACAAAA--C--GTCATGAC-----TTTTCTTCCC----------

--------------CGTATC--TTCT-----------CTTTGTGA---------------------------

-------------------------TTC--GTTTAT-GGAC--GTG-CCT-CC-TTGCCT--GGGTGC--AA

AC-----------AGCAT-A----CGAAAGT-GCTTATG-T-------------------------------

----------------------------------------GCGCAAT-A-C-G-GA-CCT-AGA-----AT-

GAAATT--GGAGAAA--TTAGG------------CCAGAATTT--CACA-A-GTT-TCGG--TAGAG-----

---------GAT-AGCCT-------------------TGG-TTTGCACA----AAATTTCTGAAAAATTC

>Pv02Sk00390/1-525 Pv02Sk00390 undefined product 4140400:4140924 forward

-------------------------TG--AAATTCCACGT-ATG-AA--T-------CT---TAACTA-AA-

TTAGAA-AC----------AAAATGTG-AC-AA-AT--TTCAGG--TC------------------------

-----------------------------------------AAAC-G--GATGAG---TATTCAC-------

---CTA-----GG-GAAAAA---TC-AAACAGTT-TC--------------------------ACA-CAC--

AAAC--G-AA-CCT------------TCC--T-TTC-------A-C--CC-T-T-GA-C---------A--G

TGTTG-AAT-A--A-A-AA--A-TT-TA-TTGCA-AATCTTGA-GGGACGAA----TTT------GAG----

-------------GGTCAAAACTCAG-C----------------CAAAA--CTTAGCACACACA---A----

------------------TAA--C----AAA-TGTGTGG----------T--A-AATATCTC-ATA-CAA--

--AAAGACCC-------AAGGA-AT-----AAGGC---GT-----AATA------AG-TCCCAGA-CCGAGA

GTGAA-------------------------------------------------------------------

------------------------------------------------------------------------

--------------C--AAAACT--------------------------GG-----------------TTTT

T----------------------TGAAAAAAAAA--C--GTCCTGGA-----TTTTCTTCCC----------

--------------TATACC---TTG-----------TTTTGTGA---------------------------

-------------------------TTT--GTTTAT-GGAC--ATG-CCT-CC-CTAGCT--GGGTGA--AA

AC-----------AACAT-A----TGAAAGT-ACCTGTG-T-------------AAA--TTTTTT----C-G

AAATTTTTCA------------------------------ACGCAAT-A-T-A-AA-CCC-AGA-----AT-

AAAATT--GCAGAGA--ATATG------------GCGAAATTA--CACA-A-ATT-TTTG--TTGAG-----

---------GAT-ACCCT-------------------TGG-TTTTTCAC---AAAATTTAAGAAAT----

>Pv02Sk00400/1-534 Pv02Sk00400 undefined product 4140929:4141462 forward

--TCCAAAATAA-----TTTTTTACTG--AAATTCCACAT-AAG-AA--T-------CT---TGACTG-AA-

TTAGGG-AG----------AAAACGTG-AC-AA-AT--T--AGG--TC------------------------

-----------------------------------------AAAC-A--GATGAG---TATTCTC-------

---CTA-----GGAAAAAAA---TC-AAACAGTT-TC--------------------------ACA-CAC--

AAAC--G-AA-CCT------------TTC--T-TTC-------A-C--CC-TCT-AA-T---------A--A

TGTTG-AAT-A--A-A-AA--A-TT-TA-TTGCA-AATCTTGT-GGGACGAA----TTT------GAG----

-------------GATCAAAACTC------------------------------AGCACACACA---A----

------------------TAA--A----GAA-TGTGTGG----------T--A-AATATTTC-ATA-CGA--

--AAAGACCC-------AATGA-AT-----AAGGC---GT-----AATA------AG-TCTCAGA-CCGAGA

GTAAA-------------------------------------------------------------------

------------------------------------------------------------------------

--------------C--AAAATT--------------------------GG-----------------TTTT

C----------------------CCAAAAAAAA---T--GTCCTAGC-----TTTTCTTCCC----------
[truncated: 3,409,494 more chars]
