# Supplementary material for: The Subtelomeric khipu Satellite Repeat from Phaseolus vulgaris: Lessons Learned from the Genome Analysis of the Andean Genotype G19833
Source: Front Plant Sci. 2013 Oct 16;4:109. doi: 10.3389/fpls.2013.00109 (PMC3797529; doi:10.3389/fpls.2013.00109)
Supplement: Figure S2 — WebLogo representation of the consensus sequence derived from the multiple alignment of the khipu units and sequence of one representative khipu unit from each major clade. [file 47451_Geffroy_DataSheet2.DOCX]

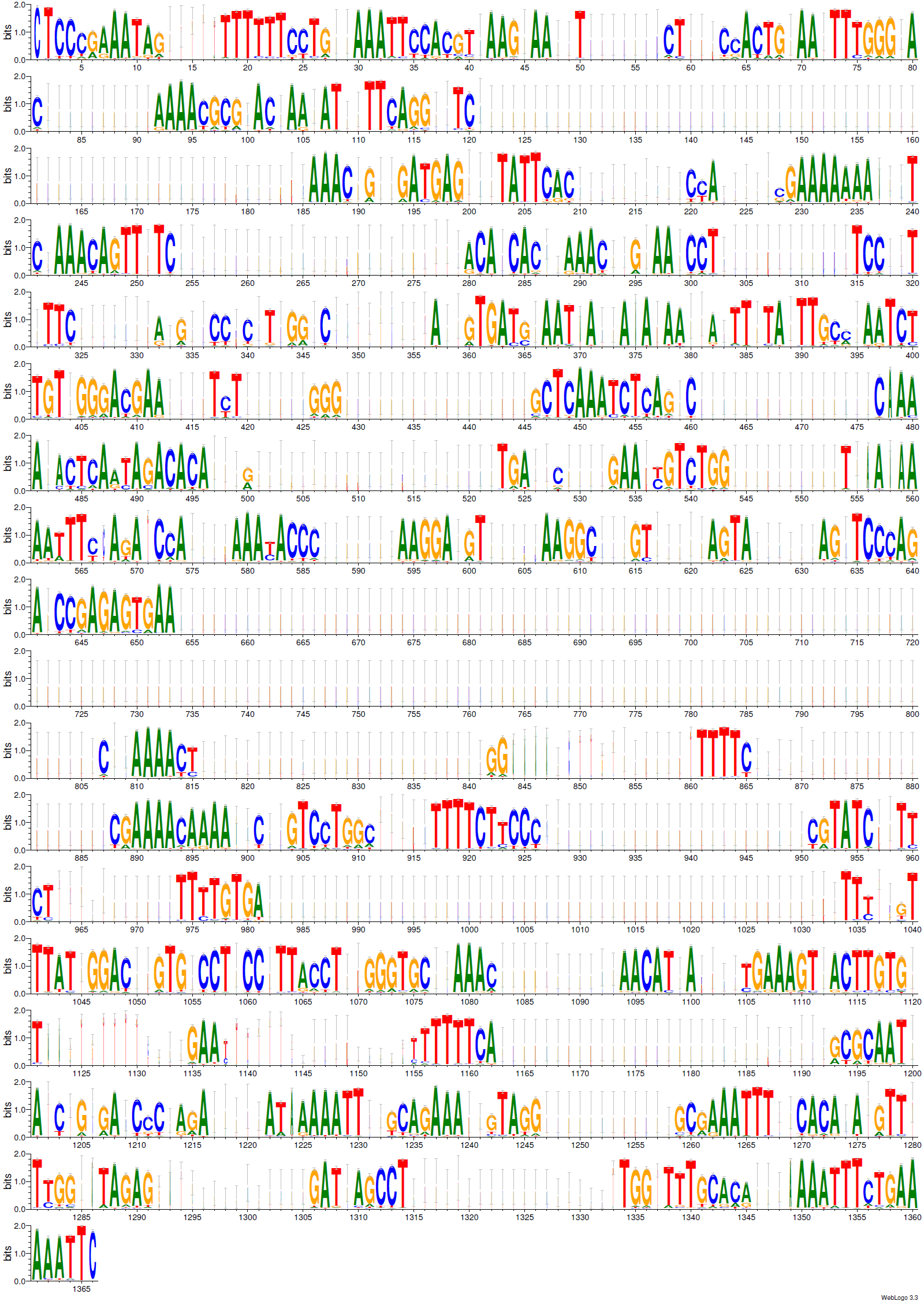


WebLogo representation of the consensus sequence derived from the multiple alignment of the *khipu* units. The sizes of the different letters are proportional to the frequency of the base in the *khipu* units multiple sequence alignment.

Sequence of one representative *khipu* from each major clade presented in Figure 1. Positions and orientation of each *khipu* unit on pseudomolecules are indicated in bracket.

>Pv08Ck00590 (31897394:31897946 reverse) / clade A

ccaaatataatttcttgttgaaatcttatgaaagaattgcaacagaatttggaacaaaac

gcgaaaaatttcagttcaaacgaacgagtattcagttagaaaatgatcaaacagtttcac

actgaaacgaaccttccttgcaaccgtagcaatgatgaatacaaaattcattgcaaatct

tgtggaacgaatttgaggctcatatcttatcctcaactcaatacacacaataaggaattt

ctggtaaaatattcaaatcaaaacacccaagaaataaggtgtaataagtcccagaccgag

aggaaacaaaactagaaaacttggttttccgaaaatagaacgtccttccttttcctcctt

agatctttttctgtattttctttttgaacattcctccttgactagataaaaacgacatag

gaaagtacttgtgtaagtgttttcggaacttttcagcgaaatacgaactcaaaataaaat

tgcagaaatggggcaaaatttcacaaatctggaatgaggagcaccttggagtgctagaag

tttcaggaacatt

>Pv08Lk00820 (59152150:59152688 reverse) / clade B

ctcccgaaatggtttttcctgaaattccgcgtaagaatctccactgagtttgggtcaaaa

cgcgacagatttcaggtcaaatggatgagtattcacccaggaaaagaaatcaaacagttt

cacacacaagcgaacctcatttcagcccgtacactgatgaataaaaaatttattgcgaat

gttgtgggacgattctggggctcaaatctcagccaaaactcagtagacacaatgacgaat

gtctggtcaaaatttcagaccaaaatacccaaggggtaaggcgcagtaagtcccagaccg

agagtgaacaaaaccgattttccgaaaacaaaacgtcctcgcttttcttccccgtatctt

ctttctgtgatttgtttatggtcgtgcctcctcacctgggtgcaaacaacatacgaaagt

agttgtgcgaattttttcaacgcaatacggacgcggaatttttttttcagaaagtagggc

gaaatttcacaagttttgctagaggatagccttggtttgcacaaaatttctgaaaaaat

>Pv03Sk00260 (381833:382371 forward) / clade C

tccgaaatagttttttcctgaaagtccacccaagaacctccactgaattttggacacaac

gcgacaaatttcaggtcaaacggatgagtattcacccacgaaaaaaatcaaacagtttcg

cacacaaacgaacattcctttcagccctggcaataacgaataaaaactttattaacaatc

ttgtgggacgaatctggggctcaaacctcagcgaaaactcaatagacactgtgacggatg

tctggtaaaaatttcagacaaaaatacccaaggagtaaggcgtagtaagtcccagaccga

gagtgaacaaaatcggttttcttaaaaaaaaacgtcctggcctttcttccccgcatcttc

tttttgtgattcctttatggacgtgcttctttgcctgggtgcaaacaacacacgaaagtg

ctcgtgtgaatttttttcggtgcgatacggacccagaatgaaattgcataaattaggcca

aaattttacaagtttcggtagaggataaccttggttttcacaaaatttctgaaaaattc

>Pv11Lk02150 (47608450:47608988 reverse) clade D

ctgaaatagttttttcctaaaattccacgtatgaatctccagtgattttgcgacaaaacg

cgaaaaatttcaggtcaaacggatgagtattcacccacgaaaaaaatcaaacagtttcac

acacaaacgaagcttccattcagccctagtagtgatcaataaaaatatttattgccaatc

ttttgggactaatctggggcgcaaatctcagcaaaaactcaaaagacacagtttcgaacg

tctggtaaaaatttcatatcaaaatacctaaagagtaaggtgtagtaagtcctagaccca

gcggcaacaaaactagtattccgaaaacaagacgtgctggcttttcttccccatatctac

tttttgtgattcgtttatggacgtgtctcctttcctgggggcaagcaacatatgaaagaa

atggcgtgaatttttttgggcaatacgaacacagaataaaaattgaagaatgtagggcga

aatttcacaagttttggattaggaaagccttgggttgcaagaaaatttttgaaaaattc

>Pv05Sk00070 (10368:10891 forward) / clade E

ctcccgaaatagtttcttcctgaaattctacataagaatcaccattgaatttgggacaaa

acgcgacaaatttcaggtcaaacggatgagtattcgcccacgaaaaaaattaaacagttt

cgcacacaaatccctggcagtgatgaattaaaaatttattgtcaatcttgtgggacgaat

ctggggctcaaatctcagccaaaactcaataaatacggtgacaaacgtcaagtaaaaatt

tcagaccaaaatacataaggagtaaggcgtagtaagtcttagaccgagagtgaacaaaac

tggttttctgaaaacaaaacgtccttcttttcttcaccgtatcttctttttgtgatttgt

ttacggtcgtgcctccttacctgggtgcaaacaacatatgaaagtacttgtctgaatttt

ttcagcgaaatacggaccaagaataaaattgcagaaagtagggcaaaatttcacaagttt

cggtagaggatagccttggtttgcacaaaatttctgaaaaattc

>Pv11Lk02800 (48042259:48042797 reverse) / clade F

tcttggaagagttttttcctgaaacttcacgttagaatatccactgaatttgggacaaat

tgcgacaaaattcaggtcaaacggacgagtattcacccacgaaaaaatcaaacagtttca

cgcacaaacgaaccttcccttgaaccctggtagtgatcattaaaaaatgtattgctaatc

aagttggacgaatctggggctcaaatctcagcgaaaactcaatagacacagtttcgaacg

tctggtaaaaatttcagaccaaaacactcaaggagtaaggcgtagtaagtcccagaccca

gactgaacaaaactggttttccaaaaacagaacgccctggcttttcttccgcgtatcttc

tctttgtgatttgtttatggacctgcctccttacctaggtgcaaacaacatatgaaagta

cttctgtgaattttttcagcgcgatacggatcgagaataaaaattgcagaaagtagggcg

aaatttcacaagttttggtagaggacagtcgtggtctgcacaaaatttctaaaaaattc

>Pv01Sk00860 (876570:877107 forward) / clade G

tcccgaaatagttttttcctgaaagtccacataagaatatgcactgaatttgggacaaaa

cgcgacaaatttcaggtcaaacgaatgagtattcaccaacgaaaaaaaacaaaaagtttc

gcacacaaacgaaccttcctttcagctctggcagtggtgaataaaaaatttattgccaat

cttgtgaaaagaatctggtgctaaaatctcagcctaaactcaacagacacagtgacgaac

gtctggtaaaaatttcagaccaaaatacccaaggagtagggcctggtaagtcccaaaccg

agactgaacaaaactggttttccgaaaacaaaacgtcctggcttttcttccccgtatctt

ctttttgtgatttgtttatggaagtccctccttacctaggtgcaaacaacatatgcaagt

actattgtgaattttttcaacgcaatacggacccggaataaaattgcagaaagtagggcg

aaatttcacaagttttggtagaggatagccttggtttgcacaaaatttctgaaaaatt

>Pv04Sk03760 (4460039:4460571 forward) / clade H

ctcccgaaatagttttttcctgaaattccatgtaagaagctccattgaatttgggacaaa

acgcgacaaattttagctcaaacagatgagtattcacccaagaaaaaaatcaaatagttt

cacacacaaacaaaccttcctttcgaccctagcagtgatgaataaaaaatttattgtgaa

tcttgtgggacgaatttggggctcaaatgtcaaccaaaacttagtagacacagtgacgaa

catctggtaaaaatttcagacaaaaatacctaaggagtaaggcgtagtaagtcccagacc

gagagtgaacacaactggttttccgaaacaaaacgtcatggcttttctcctacgtatctt

ctttatgttatttgtttatggacgtcgctccttatctgggtgaaaacaacatatgaaggt

atatgtctgaattttttcaacgcaatacggactcagaataaaacttcagaaagtagggga

atattccacaagttttggtagaggattgccttggtttgctcaaaatttctgaa

Pourcentage of nucleic identity between representative *khipu* from each major clade presented in Figure 1.

|  | A Centromeric *khipu* | B | C | D | E | F | | | G | H |
| --- | --- | --- | --- | --- | --- | --- | --- | --- | --- | --- |
| A  Centromeric *khipu* |  | 73 | 73 | 72 | 71 | 73 | | | 74 | 73 |
| B |  |  | 84 | 82 | 85 | 84 | | | 87 | 85 |
| C |  |  |  | 81 | 84 | 83 | | | 86 | 83 |
| D |  |  |  |  | 81 | 83 | | | 83 | 82 |
| E |  |  |  |  |  | 84 | | | 87 | 85 |
| F |  |  |  |  |  |  | | | 87 | 83 |
| G |  |  |  |  |  |  | | |  | 87 |
| H |  |  |  |  |  | |  |  | |  |
