# Supplementary material for: The Subtelomeric khipu Satellite Repeat from Phaseolus vulgaris: Lessons Learned from the Genome Analysis of the Andean Genotype G19833
Source: Front Plant Sci. 2013 Oct 16;4:109. doi: 10.3389/fpls.2013.00109 (PMC3797529; doi:10.3389/fpls.2013.00109)
Supplement: Figure S3 — Phylogenetic tree of whole-genome khipu sequence. [file 47451_Geffroy_DataSheet3.DOCX]

(((Pv11Lk03400:0.06135,(Pv04Sk01900:0.07734,((Pv04Sk01890:0.06240,(Pv04Sk01870:0.06485,(Pv04Sk02020:0.05661,(Pv04Sk01850:0.04674,Pv04Sk01860:0.05388)0.823:0.00515)0.370:0.00441)0.719:0.00152)0.730:0.00102,(Pv04Sk02010:0.05109,(Pv04Sk01880:0.04635,Pv04Sk02030:0.06589)0.107:0.00167)0.746:0.00014)0.840:0.00305)0.949:0.01057)0.717:0.00166,(((Pv01Sk00700:0.07140,((Pv11Lk05930:0.06010,Pv11Lk05940:0.05801)0.956:0.01641,(((Pv11Lk04640:0.06934,Pv11Lk04610:0.05377)0.080:0.00160,Pv11Lk04630:0.08646)0.701:0.00332,Pv11Lk04620:0.06181)0.898:0.01035)0.749:0.00178)0.282:0.00120,(Pv10Sk04870:0.05424,Pv11Lk03820:0.04404)0.680:0.00264)0.707:0.00101,((((((Pv04Lk04940:0.08650,(Pv04Sk03950:0.06299,Pv11Lk04790:0.06780)0.457:0.00272)0.285:0.00442,Pv05Sk00340:0.05057)0.901:0.00554,(((Pv04Lk04460:0.06206,Pv10Lk02870:0.05681)0.903:0.01615,(((Pv04Lk04920:0.03857,Pv04Lk04930:0.06013)0.963:0.01462,(Pv04Lk04950:0.06300,(Pv04Lk05540:0.06251,Pv04Lk05560:0.05358)0.174:0.00155)0.824:0.00716)0.751:0.00159,(Pv04Lk05550:0.04308,((((((((Pv10Lk02770:0.01657,Pv10Lk02790:0.02730)0.932:0.00929,(Pv10Lk02830:0.03515,Pv10Lk02850:0.02983)0.710:0.00118)0.425:0.00229,(Pv10Lk02800:0.03881,(Pv10Lk02820:0.01847,Pv10Lk02840:0.02835)0.993:0.02241)0.685:0.00735)0.857:0.00042,(Pv10Lk02810:0.03668,(Pv10Lk00750:0.04048,(Pv10Lk00740:0.01950,(Pv10Lk00730:0.02267,Pv10Lk02700:0.02244)0.627:0.00822)0.877:0.01069)0.952:0.00822)0.119:0.00014)0.926:0.00311,(Pv10Lk00270:0.04457,(Pv10Lk02760:0.02863,Pv10Lk02780:0.02343)0.952:0.01106)0.814:0.00308)0.486:0.00014,Pv10Lk02860:0.06480)0.897:0.00462,((((Pv04Sk03070:0.03258,Pv04Sk03080:0.01925)0.599:0.00322,(Pv04Sk03100:0.02411,(Pv04Sk03060:0.01929,Pv04Sk03090:0.03423)0.914:0.00776)0.928:0.00014)0.871:0.00162,((Pv04Sk03030:0.02282,Pv04Sk03050:0.02085)0.884:0.00456,Pv04Sk03010:0.02912)0.855:0.00313)0.000:0.00015,(Pv04Sk03000:0.04392,Pv04Sk03040:0.04080)0.767:0.00026)0.872:0.00155)0.201:0.00014,(((Pv04Lk05100:0.02231,(Pv04Lk05080:0.01444,Pv04Lk05090:0.01918)0.860:0.00465)0.000:0.00015,(Pv04Lk05050:0.02917,Pv04Lk05070:0.05874)0.901:0.00626)0.984:0.00927,((((Pv04Lk05460:0.06041,Pv04Lk05420:0.04770)0.455:0.00565,((Pv04Lk05440:0.03055,Pv04Lk05450:0.02706)0.915:0.00670,(Pv04Lk05430:0.03590,Pv04Lk05470:0.06058)0.834:0.00473)0.881:0.00136)0.278:0.00015,((Pv04Lk05370:0.04088,(Pv04Lk05360:0.05101,(Pv04Lk05350:0.02924,Pv04Lk05380:0.03126)0.748:0.01957)0.819:0.00014)0.877:0.00337,Pv04Lk05480:0.04818)0.749:0.00104)0.893:0.00305,(Pv04Lk05110:0.04402,(((Pv04Lk05590:0.02107,(Pv04Lk05580:0.02363,(Pv04Lk05570:0.04345,Pv04Lk05600:0.02313)0.821:0.00755)0.742:0.00175)0.768:0.00181,Pv04Lk06910:0.02398)0.707:0.00156,Pv04Lk05610:0.03070)0.961:0.00803)0.992:0.00016)0.249:0.00014)0.888:0.00155)0.891:0.00465)0.867:0.00311)0.680:0.00026)0.927:0.00129,((((((((Pv10Lk04620:0.03134,Pv10Lk04650:0.02451)1.000:0.03663,(Pv10Lk04700:0.05412,(Pv10Lk04750:0.04402,(Pv10Lk04730:0.06454,Pv10Lk04780:0.02780)0.871:0.00873)0.934:0.00730)0.736:0.00237)0.813:0.00355,(Pv10Lk01330:0.03933,(Pv10Lk04590:0.05256,Pv10Lk04680:0.06303)0.795:0.00688)0.589:0.00201)0.798:0.00011,((((Pv10Lk01210:0.05367,(Pv10Lk04610:0.02129,Pv10Lk04640:0.02617)0.983:0.01789)0.857:0.00616,(((Pv10Lk01400:0.03474,Pv10Lk01420:0.04367)0.867:0.00592,(((Pv10Lk01350:0.03613,Pv10Lk01360:0.03671)0.938:0.00760,(Pv10Lk01310:0.04684,(Pv10Lk01380:0.03360,((Pv10Lk01300:0.03826,Pv10Lk01410:0.05732)0.775:0.00261,Pv10Lk01370:0.03891)0.941:0.00790)0.548:0.00245)0.693:0.00151)0.696:0.00015,(Pv10Lk01320:0.04403,Pv10Lk01340:0.03363)0.810:0.00367)0.400:0.00159)0.784:0.00160,(((Pv10Lk04550:0.04405,Pv10Lk04570:0.05426)0.927:0.00987,((Pv10Lk04480:0.03359,Pv10Lk04500:0.04289)0.986:0.01975,(Pv10Lk04470:0.04822,(Pv10Lk04450:0.03138,Pv10Lk04460:0.03574)0.833:0.01652)0.900:0.00914)0.593:0.00196)0.878:0.00392,(Pv10Lk04770:0.08062,(Pv10Lk04760:0.06251,(Pv10Lk04720:0.05192,Pv10Lk04740:0.04063)0.989:0.02118)0.698:0.00130)0.941:0.00932)0.626:0.00016)0.402:0.00015)0.387:0.00014,((((Pv10Lk04490:0.04680,Pv10Lk04530:0.04644)0.990:0.02081,((Pv10Lk04600:0.05506,(Pv10Lk04630:0.02891,Pv10Lk04660:0.02824)0.984:0.02003)0.981:0.01935,(Pv10Lk04560:0.04396,Pv10Lk04580:0.05003)0.733:0.01386)0.874:0.00594)0.753:0.00128,(Pv10Lk04540:0.05866,(Pv10Lk01230:0.05044,Pv10Lk01430:0.04137)0.580:0.00518)0.707:0.00039)0.916:0.00096,(Pv10Lk01390:0.04240,Pv10Lk01220:0.06026)0.863:0.00015)0.779:0.00155)0.175:0.00015,(Pv10Lk04710:0.05472,(Pv10Lk04690:0.06127,Pv10Lk01240:0.05645)0.632:0.00404)0.799:0.00016)0.311:0.00016)0.998:0.01147,(((((Pv04Lk04350:0.06952,Pv04Sk03980:0.05897)0.681:0.00579,(Pv04Lk04470:0.07570,((Pv01Lk02210:0.05992,(Pv04Sk02500:0.02063,Pv04Sk02550:0.01520)1.000:0.06525)0.891:0.01300,(Pv05Sk00030:0.06960,(Pv01Lk02250:0.06114,(Pv01Lk02220:0.06916,(Pv01Lk02230:0.04856,Pv01Lk02240:0.05984)0.798:0.00804)0.855:0.00377)0.839:0.00744)0.821:0.00907)0.702:0.00272)0.714:0.00039)0.585:0.00172,((Pv04Lk04490:0.06025,Pv04Lk06310:0.07358)0.928:0.01376,Pv04Lk04480:0.06481)0.776:0.00361)0.712:0.00157,(((((Pv04Lk06250:0.02153,Pv04Lk06240:0.03349)0.858:0.00442,(Pv04Sk03110:0.01427,(Pv04Lk06260:0.02062,(Pv04Lk06280:0.01824,Pv04Lk06270:0.02538)0.896:0.00490)0.743:0.00162)0.925:0.00656)0.977:0.01190,((Pv04Lk04570:0.03989,Pv04Lk04590:0.04591)0.786:0.00391,((Pv03Sk00520:0.04130,Pv03Sk00530:0.01827)0.846:0.00692,Pv04Lk04580:0.05679)0.708:0.00154)0.952:0.00850)0.734:0.00318,(Pv05Sk01510:0.03905,((Pv04Sk03740:0.06678,(Pv04Sk03640:0.07948,Pv04Sk03670:0.05352)0.813:0.00654)0.860:0.00422,((((Pv05Sk01490:0.02942,Pv05Sk01330:0.05017)0.780:0.00281,((Pv05Sk01470:0.01603,Pv05Sk01480:0.01768)1.000:0.02445,((Pv04Lk04300:0.02260,Pv04Lk04390:0.02914)1.000:0.03361,((Pv04Lk04290:0.05756,Pv04Lk04380:0.03819)0.990:0.02518,(((Pv04Lk04310:0.08236,Pv04Lk04400:0.05440)0.945:0.01782,Pv04Lk04260:0.07324)0.814:0.00446,((((Pv04Lk04340:0.05130,(Pv04Lk04320:0.02194,Pv04Lk04330:0.02733)0.971:0.01587)0.997:0.01992,Pv04Lk04230:0.04245)0.568:0.00015,((Pv04Lk04710:0.03108,Pv04Lk04720:0.02602)0.866:0.00422,(((Pv04Lk04740:0.03970,Pv04Lk04750:0.03056)0.397:0.00575,(((Pv04Lk04820:0.01641,Pv04Lk04830:0.02303)0.881:0.00913,Pv04Lk04860:0.02691)0.923:0.00539,((Pv04Lk04800:0.03475,(Pv04Lk04760:0.01920,Pv04Lk04780:0.02602)0.643:0.00856)0.957:0.01105,Pv04Lk04880:0.06347)0.557:0.00016)0.908:0.00183)1.000:0.00015,(Pv04Lk04730:0.03896,(((Pv04Lk04790:0.02989,Pv04Lk04770:0.03505)0.758:0.00259,(Pv04Lk04840:0.03269,(Pv04Lk04810:0.02559,Pv04Lk04850:0.00589)0.730:0.00352)0.980:0.00987)0.853:0.00303,Pv04Lk04870:0.06011)0.925:0.00641)0.738:0.00151)0.915:0.00015)0.974:0.00788)0.730:0.00156,Pv04Lk04220:0.07078)0.775:0.00172)0.975:0.00016)0.907:0.00376)0.488:0.00015)0.842:0.00311)0.069:0.00014,(Pv04Sk03850:0.05625,(Pv04Sk03790:0.05013,Pv05Sk01460:0.05158)0.588:0.00456)0.936:0.00747)0.046:0.00014,(Pv04Sk03820:0.04934,(Pv04Sk03940:0.06303,Pv04Sk03960:0.04162)0.838:0.00597)0.859:0.00330)0.280:0.00015)0.919:0.00015)0.906:0.00374)0.616:0.00016,(((Pv04Sk03750:0.04874,Pv04Sk03760:0.06210)0.932:0.01314,(Pv01Sk01040:0.06472,Pv04Lk04560:0.04822)0.850:0.00893)0.832:0.00391,(((((((Pv11Lk06380:0.03626,((Pv11Lk06260:0.04554,Pv11Lk06280:0.05418)0.785:0.00024,((Pv11Lk06270:0.03076,Pv11Lk06340:0.02889)0.984:0.01897,Pv11Lk06290:0.04104)0.756:0.00130)0.792:0.00160)0.763:0.00152,((Pv11Lk06350:0.03539,(Pv11Lk06300:0.05376,Pv11Lk06310:0.03766)0.813:0.00474)0.911:0.00516,(((((Pv11Lk06230:0.02401,(Pv11Lk06100:0.00936,Pv11Lk06250:0.01284)0.949:0.00648)0.755:0.00152,((Pv11Lk06180:0.00975,(Pv11Lk06120:0.00656,Pv11Lk06210:0.01890)0.198:0.00143)0.998:0.01566,(Pv11Lk06190:0.01743,(Pv11Lk06200:0.01280,Pv11Lk06220:0.01435)0.920:0.00631)0.941:0.00014)0.831:0.00306)0.219:0.00014,(Pv11Lk06140:0.02121,Pv11Lk06240:0.02772)0.726:0.00107)0.680:0.00016,((Pv11Lk06110:0.00944,Pv11Lk06170:0.01111)0.440:0.00156,(Pv11Lk06160:0.01322,(Pv11Lk06150:0.01192,(Pv11Lk06080:0.03395,Pv11Lk06130:0.00962)0.821:0.00353)0.801:0.00316)0.987:0.01049)0.844:0.00015)0.806:0.00156,(Pv11Lk06330:0.03479,(Pv11Lk06090:0.02294,(Pv11Lk06320:0.01755,Pv11Lk06360:0.03676)0.944:0.00961)0.794:0.00080)1.000:0.00014)0.787:0.00155)0.782:0.00155)0.914:0.00315,((Pv11Lk06370:0.03522,(Pv11Lk06400:0.02887,Pv11Lk06420:0.01468)0.826:0.00970)0.714:0.00782,Pv11Lk06410:0.04711)0.669:0.00016)0.826:0.00310,(Pv04Lk06120:0.04400,(Pv04Lk06130:0.02245,(Pv04Lk06200:0.04557,(((Pv04Lk06150:0.02914,Pv04Lk06210:0.01433)0.749:0.00147,(Pv04Lk06160:0.02233,(Pv04Lk06170:0.01444,Pv04Lk06180:0.01756)0.930:0.00795)0.726:0.00153)0.767:0.00155,(((Pv04Lk06580:0.02284,(((((Pv04Lk06590:0.01402,Pv04Lk06620:0.02132)0.959:0.01064,(Pv04Lk06630:0.01829,Pv04Lk06640:0.02940)0.954:0.01161)0.762:0.00229,(Pv04Lk06650:0.00655,Pv04Lk06670:0.01875)0.743:0.00183)0.820:0.00156,Pv04Lk06600:0.04589)1.000:0.00015,(Pv04Lk06660:0.02246,Pv04Lk06700:0.03887)0.486:0.00648)0.766:0.00141)0.884:0.00310,Pv04Lk06140:0.03224)0.716:0.00154,Pv04Lk06190:0.03746)0.126:0.00015)0.465:0.00016)0.437:0.00299)0.936:0.00672)0.959:0.00014)0.797:0.00160,(Pv04Lk06410:0.03070,(((((Pv04Lk06400:0.02230,Pv04Lk06360:0.03388)0.026:0.00014,Pv04Lk06370:0.03223)0.555:0.00153,Pv04Lk06340:0.02898)0.799:0.00158,(Pv04Lk06440:0.02139,Pv04Lk06350:0.03625)0.724:0.00091)0.794:0.00156,((Pv04Lk06420:0.02552,(Pv04Lk06330:0.03264,Pv04Lk06430:0.01429)0.795:0.00457)0.753:0.00176,(Pv04Lk06380:0.03034,Pv04Lk06390:0.02376)0.917:0.00719)0.744:0.00143)0.713:0.00145)0.978:0.00950)0.160:0.00015,(Pv04Lk06070:0.05129,((Pv04Sk03330:0.02988,Pv04Lk06080:0.03150)0.739:0.00076,((Pv04Lk06060:0.03784,Pv04Lk06110:0.02562)0.981:0.01654,(Pv04Sk03320:0.05274,Pv04Sk03340:0.03242)0.591:0.00658)0.787:0.00025)0.767:0.00157)0.778:0.00154)0.974:0.00658,((Pv04Sk01750:0.05015,Pv04Sk01760:0.06618)0.935:0.01157,(Pv11Lk03840:0.06900,(Pv11Lk05720:0.03858,(Pv04Lk04960:0.06128,Pv10Lk01200:0.10843)0.874:0.00984)0.824:0.00338)0.816:0.00016)0.899:0.00525)0.410:0.00014)0.700:0.00013)0.390:0.00015)0.771:0.00213,(Pv04Sk04050:0.05406,(Pv04Sk03930:0.05096,Pv04Sk04010:0.04882)0.985:0.02401)0.918:0.01319)0.756:0.00190)0.000:0.00016,(Pv04Lk04910:0.05972,(Pv01Lk02300:0.04213,Pv06Lk00200:0.06048)0.990:0.02677)0.771:0.00546)0.615:0.00014,((((((Pv04Sk03630:0.07671,(Pv04Sk03610:0.05275,Pv04Sk03810:0.08930)0.839:0.00843)0.796:0.00398,(Pv04Sk03860:0.05350,(Pv04Sk03680:0.07066,(Pv04Sk03800:0.10215,Pv04Sk03970:0.04529)0.724:0.00387)0.251:0.00234)0.716:0.00484)0.057:0.00014,(Pv05Sk01500:0.04475,Pv04Sk03660:0.06770)0.665:0.00164)0.737:0.00140,((Pv04Sk03830:0.06461,(Pv05Sk00360:0.05283,(Pv04Lk04270:0.07204,Pv10Lk01190:0.08404)0.843:0.00878)0.735:0.00376)0.754:0.00176,((Pv04Sk03870:0.03430,Pv04Sk03910:0.04189)0.874:0.00631,Pv05Sk00400:0.06008)0.134:0.00015)0.901:0.00334)0.346:0.00015,((((Pv05Sk01520:0.03753,(Pv05Sk00370:0.04908,Pv04Sk03730:0.08104)0.615:0.00091)0.898:0.00488,((Pv05Sk01300:0.03897,(Pv05Sk01320:0.04550,(Pv05Sk01290:0.05549,Pv05Sk01310:0.03671)0.825:0.00739)0.578:0.00318)0.807:0.00332,((Pv05Sk01340:0.03415,Pv05Sk01360:0.03011)0.929:0.00855,(Pv05Sk01350:0.02918,Pv05Sk01370:0.03603)0.531:0.00151)0.752:0.00175)0.905:0.00338)0.764:0.00149,(Pv04Sk04000:0.07434,(Pv05Sk00540:0.02563,((Pv05Sk00430:0.01749,((Pv05Sk00490:0.01944,Pv05Sk00520:0.02102)0.909:0.00605,(Pv05Sk00460:0.02582,(Pv05Sk00480:0.02262,Pv05Sk00530:0.01430)0.814:0.00303)0.785:0.00179)0.808:0.00156)1.000:0.00014,((Pv05Sk00420:0.02685,(Pv05Sk00470:0.02189,Pv05Sk00510:0.00531)0.541:0.00499)0.744:0.00462,Pv05Sk00450:0.01904)0.812:0.00160)0.561:0.00485)0.924:0.00807)0.765:0.00177)0.700:0.00014,((Pv04Lk04280:0.05727,Pv04Sk03990:0.04319)0.616:0.00682,(Pv05Sk01280:0.04803,(Pv05Sk00550:0.03460,Pv05Sk01450:0.03116)0.871:0.00455)0.876:0.00016)0.798:0.00289)0.625:0.00016)0.392:0.00014,(Pv04Sk03880:0.05172,((Pv04Sk03890:0.05897,(Pv04Sk04020:0.03917,Pv04Sk03920:0.03440)0.955:0.01195)0.776:0.00282,Pv04Sk03900:0.08519)0.654:0.00215)0.967:0.00919)0.916:0.00313)0.278:0.00016,(Pv04Lk04970:0.07074,((Pv04Lk04550:0.03689,(Pv04Lk04510:0.02041,Pv04Lk04520:0.05123)0.454:0.00863)0.781:0.01130,(Pv04Lk04530:0.03011,Pv04Lk04500:0.04580)0.486:0.00541)0.981:0.01670)0.875:0.00592)0.135:0.00016)0.227:0.00014)0.128:0.00014,(((Pv04Lk05930:0.05831,((((Pv04Lk05890:0.00622,Pv04Lk05910:0.02256)0.987:0.01276,(Pv04Lk05870:0.02595,Pv04Lk05880:0.02770)0.910:0.00601)0.748:0.00015,Pv04Lk05850:0.02561)0.443:0.00157,(Pv04Lk05860:0.02240,(Pv04Lk05900:0.02590,Pv04Lk05920:0.02760)0.956:0.00947)0.726:0.00166)0.821:0.00289)0.974:0.00796,(Pv04Lk04640:0.03065,((((Pv01Sk01870:0.06122,((Pv01Sk01920:0.05123,Pv01Sk01910:0.03874)0.776:0.00383,(Pv01Sk01930:0.03669,Pv01Sk01940:0.01872)0.159:0.00457)0.881:0.00396)0.901:0.00016,((Pv01Sk01830:0.03916,((Pv01Sk01840:0.03476,Pv01Sk01850:0.04490)0.873:0.00623,(Pv01Sk01880:0.04510,Pv01Sk01900:0.04666)0.861:0.00618)0.618:0.00165)0.744:0.00140,Pv01Sk01860:0.05636)0.855:0.00014)0.800:0.00154,(Pv10Lk00760:0.03238,(Pv10Lk00720:0.05307,((Pv10Lk00690:0.01703,(Pv10Lk00680:0.02294,(Pv10Lk00700:0.01526,Pv10Lk00710:0.02326)0.912:0.00633)0.848:0.00382)0.978:0.01045,(Pv10Lk00640:0.05493,(Pv10Lk00620:0.02069,(Pv10Lk00610:0.02090,Pv10Lk00630:0.01116)0.888:0.00464)0.683:0.00483)0.847:0.00333)0.935:0.00517)0.364:0.00015)0.915:0.00342)0.781:0.00160,((Pv04Lk04600:0.03115,Pv04Lk04610:0.02980)0.886:0.00755,(Pv04Lk04620:0.03562,Pv04Lk04630:0.02304)0.569:0.00633)0.791:0.00198)0.781:0.00159)0.730:0.00161)0.841:0.00290,(((Pv04Sk01710:0.02875,(((Pv04Sk01280:0.06026,(Pv04Sk01720:0.04215,((((Pv04Sk01310:0.03394,(Pv04Sk01290:0.02711,Pv04Sk01300:0.01743)0.903:0.00808)0.875:0.00546,(Pv04Sk00640:0.03405,(Pv04Sk00130:0.03156,Pv04Sk01340:0.03395)0.958:0.01500)0.898:0.00495)0.380:0.00014,((((Pv04Sk00140:0.02106,Pv04Sk00200:0.01616)0.978:0.01440,(Pv04Sk01550:0.02571,((Pv04Sk00170:0.00916,Pv04Sk00220:0.01862)0.984:0.01511,(Pv04Sk00150:0.01640,Pv04Sk00210:0.00593)0.286:0.00549)0.907:0.00885)0.167:0.00015)0.912:0.00471,((Pv04Sk00190:0.03567,Pv04Sk01330:0.01959)0.939:0.01014,(Pv04Sk00630:0.03937,Pv04Sk00650:0.04026)0.889:0.00718)0.306:0.00164)0.721:0.00152,(Pv04Sk01320:0.01847,Pv04Sk01540:0.02023)0.968:0.01133)0.850:0.00298)0.429:0.00353,(Pv04Sk00100:0.02372,Pv04Sk00110:0.01479)0.765:0.00460)0.912:0.00585)0.745:0.00144)0.758:0.00015,((Pv04Sk01730:0.04257,Pv04Sk01740:0.02082)0.968:0.01099,((((Pv04Sk00590:0.02474,Pv04Sk02590:0.03147)0.905:0.00839,(Pv04Sk02610:0.03405,(Pv04Sk01410:0.02426,(Pv04Sk00600:0.01102,Pv04Sk02600:0.00625)0.997:0.01768)0.868:0.00467)0.705:0.00051)0.910:0.00625,Pv04Sk01560:0.03472)0.833:0.00382,(Pv04Sk00120:0.01579,Pv04Sk01690:0.02762)0.698:0.00461)0.174:0.00015)0.760:0.00016)0.932:0.00014,((((Pv04Sk00950:0.04017,Pv04Sk02630:0.03133)0.999:0.02909,(Pv04Sk00960:0.04241,Pv04Sk02620:0.05204)0.808:0.00682)0.856:0.00377,(Pv04Sk00970:0.05438,(Pv04Sk01270:0.04670,Pv04Sk01490:0.03745)0.887:0.01005)0.543:0.00391)0.890:0.00014,(((Pv04Sk01480:0.04934,(Pv04Sk00910:0.02842,Pv04Sk01380:0.03817)0.942:0.00906)0.816:0.00015,Pv04Sk01670:0.02061)0.796:0.00268,(((Pv04Sk00920:0.02109,Pv04Sk01390:0.00857)0.996:0.02139,(Pv04Sk01470:0.04338,Pv04Sk01680:0.01418)0.869:0.00432)0.521:0.00014,(Pv04Sk01370:0.03006,(Pv04Sk00930:0.03432,(Pv04Sk01360:0.01283,Pv04Sk01400:0.01485)0.957:0.00984)0.863:0.00628)0.990:0.01693)0.395:0.00156)0.660:0.00042)0.372:0.00016)0.902:0.00016)0.610:0.00315,Pv04Sk01500:0.05844)0.720:0.00462,Pv04Sk00180:0.05698)0.959:0.00890)0.785:0.00187)0.876:0.00609,(Pv05Sk00240:0.04889,(Pv05Sk00560:0.05539,Pv05Sk01440:0.06214)0.820:0.00783)0.771:0.00694)0.712:0.00132,((((Pv04Sk01080:0.06865,(Pv04Sk00700:0.03250,(Pv04Sk00680:0.01730,(Pv04Sk01650:0.02738,(Pv04Sk00670:0.01537,Pv04Sk01050:0.04776)0.981:0.01201)0.458:0.00017)0.929:0.00781)0.773:0.00266)0.955:0.00016,((((Pv04Sk01570:0.02152,(Pv04Sk01630:0.01540,(Pv04Sk01060:0.03175,(Pv04Sk00990:0.02371,Pv04Sk01600:0.01932)0.847:0.00561)0.845:0.00591)0.948:0.00919)0.893:0.00485,Pv04Sk01020:0.03337)0.862:0.00377,(Pv04Sk00660:0.03511,(Pv04Sk01070:0.04128,((Pv04Sk00980:0.02696,Pv04Sk01000:0.02372)0.995:0.02121,(Pv04Sk01030:0.04011,(Pv04Sk01610:0.01687,(Pv04Sk01580:0.01020,Pv04Sk01640:0.02426)0.743:0.00128)0.914:0.00735)0.917:0.00725)0.810:0.00355)0.781:0.01029)0.644:0.00358)0.637:0.00016,(Pv04Sk00710:0.06618,(Pv04Lk05020:0.00473,(Pv04Sk00720:0.02259,Pv04Sk01010:0.02759)0.733:0.00151)0.986:0.01156)0.799:0.00263)0.906:0.00014)0.950:0.00305,(((Pv04Sk01100:0.02211,(Pv04Sk00900:0.01957,Pv04Sk01240:0.01016)1.000:0.04662)0.894:0.00833,((Pv04Sk00890:0.01760,Pv04Sk01230:0.00942)0.997:0.02306,(Pv04Sk00690:0.02439,Pv04Sk01090:0.02956)0.969:0.01449)0.972:0.01395)0.760:0.00167,(((Pv04Sk00880:0.05230,Pv04Sk02690:0.03122)0.731:0.00258,(Pv04Sk01130:0.03728,(Pv04Sk00160:0.04295,((Pv04Sk01160:0.03093,Pv04Sk01190:0.02777)0.683:0.00443,(Pv04Sk01140:0.01916,(((Pv04Sk01150:0.03343,Pv04Sk01170:0.04515)0.094:0.00235,Pv04Sk01200:0.02705)0.738:0.00179,Pv04Sk01180:0.03898)0.838:0.00014)0.869:0.00015)0.873:0.00321)0.827:0.00068)1.000:0.00094)0.794:0.00163,(Pv04Sk02640:0.06099,((Pv04Sk02580:0.04376,Pv04Sk02670:0.06569)0.689:0.00208,(Pv04Sk01210:0.03074,(Pv04Sk01220:0.03369,Pv04Sk02680:0.04505)0.233:0.00207)0.765:0.00166)0.745:0.00135)0.804:0.00015)0.676:0.00016)0.738:0.00016)0.785:0.00163,(((Pv04Sk03140:0.07517,(Pv04Sk03130:0.05277,Pv04Sk03150:0.05024)0.778:0.01081)0.720:0.00112,(Pv04Sk03160:0.06839,(Pv04Sk03120:0.06434,Pv04Sk03190:0.04776)0.806:0.00839)0.875:0.00751)1.000:0.00064,Pv04Sk02660:0.04971)1.000:0.00087)0.976:0.00660)0.925:0.00015)0.659:0.00016)0.119:0.00016,((((Pv11Lk03320:0.05616,((Pv11Lk03300:0.04883,(Pv11Lk03280:0.03271,Pv11Lk03290:0.03633)0.464:0.00436)0.292:0.00325,((Pv11Lk03260:0.03277,Pv11Lk03310:0.03648)0.957:0.01281,(Pv11Lk03220:0.03893,Pv11Lk03270:0.03393)0.790:0.00516)0.818:0.00451)0.898:0.00534)0.326:0.00315,((Pv11Lk03230:0.04250,Pv11Lk03240:0.03820)0.957:0.01508,Pv11Lk03340:0.05448)0.711:0.00227)1.000:0.02121,(((((((Pv04Lk05620:0.05123,(Pv04Lk05640:0.06439,Pv04Lk05670:0.06057)0.845:0.00899)0.758:0.00148,(((Pv04Lk05700:0.03825,(Pv04Lk05720:0.03467,Pv04Lk05730:0.02954)0.882:0.00605)0.775:0.00259,((Pv04Lk05690:0.06082,(Pv04Lk05710:0.04801,Pv04Lk05760:0.04199)0.864:0.00450)0.759:0.00242,((Pv04Lk05770:0.03349,(Pv04Lk05740:0.03622,Pv04Lk05750:0.03077)0.681:0.00723)0.735:0.00345,Pv04Lk05780:0.04920)0.717:0.00030)0.746:0.00016)0.964:0.00669,(Pv04Lk05650:0.04725,(Pv04Lk05630:0.06016,Pv04Lk05660:0.06347)0.593:0.00567)0.754:0.00183)0.767:0.00161)0.984:0.00824,((((Pv04Lk05400:0.05407,Pv05Sk00040:0.04937)0.108:0.00371,Pv04Lk05300:0.05433)0.630:0.00014,(((((Pv04Lk05140:0.01945,Pv04Lk05150:0.03772)0.699:0.00629,(Pv04Lk05180:0.02736,(Pv04Lk05210:0.06978,(Pv04Lk05290:0.04752,(Pv04Lk05230:0.04229,Pv04Lk05270:0.02808)0.923:0.01038)0.950:0.01097)0.814:0.00328)0.913:0.00697)0.722:0.00016,Pv04Lk05220:0.05136)0.942:0.00300,(Pv04Lk05200:0.04518,Pv04Lk05330:0.03063)0.986:0.01848)0.865:0.00014,(((((((Pv10Lk03660:0.03620,(Pv10Lk03610:0.06316,Pv10Lk03600:0.03167)0.751:0.00246)0.423:0.00162,Pv10Lk03670:0.05132)0.784:0.00160,(Pv10Lk03590:0.04611,(Pv10Lk03580:0.02913,Pv10Lk03620:0.04280)0.753:0.00336)0.754:0.00168)1.000:0.00016,(Pv01Sk00630:0.06008,((Pv01Sk00650:0.00946,Pv01Sk00670:0.00471)0.769:0.00155,(Pv01Sk00640:0.02226,Pv01Sk00660:0.02404)0.915:0.00015)0.973:0.00968)0.231:0.00446)0.732:0.00152,(((((Pv11Lk00900:0.00014,PvA11Ak00060:0.00014)1.000:0.04552,(Pv04Lk05190:0.06974,Pv04Lk05280:0.04686)0.966:0.01845)0.740:0.00226,(((Pv01Sk01000:0.00587,Pv01Sk01030:0.01151)0.994:0.02272,Pv11Lk03570:0.09559)0.867:0.00675,((((Pv01Sk00880:0.05525,((Pv01Sk00900:0.00683,Pv01Sk00930:0.01637)1.000:0.04429,(Pv01Sk00860:0.02365,(Pv01Sk00920:0.01340,Pv01Sk00950:0.02944)0.980:0.01569)0.834:0.00403)0.803:0.00315)0.608:0.00292,Pv01Sk00980:0.05801)0.510:0.00015,((Pv01Sk00970:0.00777,(Pv01Sk00910:0.00014,Pv01Sk00940:0.01100)0.999:0.02597)0.989:0.02453,(Pv01Sk00990:0.03252,(Pv01Sk01010:0.04139,Pv01Sk01020:0.03918)0.823:0.00501)0.665:0.00014)0.929:0.00612)0.949:0.00624,(Pv01Sk00870:0.02921,Pv01Sk00890:0.04135)0.947:0.00981)0.868:0.00343)0.820:0.00328)0.819:0.00292,(Pv01Sk00750:0.05883,(PvA11Ak00100:0.06131,(PvA11Ak00110:0.06209,(Pv11Lk00950:0.03100,PvA11Ak00070:0.04339)0.932:0.01100)0.070:0.00060)0.777:0.00249)0.804:0.00301)0.025:0.00016,(PvA11Ak00130:0.02902,((Pv01Sk00830:0.03102,Pv01Sk00850:0.02788)0.842:0.00454,(Pv01Sk00840:0.02522,(((Pv01Sk00760:0.02661,Pv01Sk00770:0.02820)0.939:0.01216,(Pv01Sk00780:0.01963,(Pv01Sk00790:0.02685,Pv01Sk00820:0.03398)0.111:0.00332)0.320:0.00355)0.862:0.00459,(Pv01Sk00800:0.00515,Pv01Sk00810:0.00784)0.996:0.01873)0.564:0.00334)0.419:0.00174)0.935:0.00629)0.752:0.00153)1.000:0.00018)1.000:0.00144,(((Pv01Sk00740:0.05587,Pv10Sk05040:0.05945)0.841:0.00808,(Pv01Sk00730:0.04905,(Pv11Lk00890:0.00014,PvA11Ak00050:0.00016)1.000:0.04655)0.421:0.00367)0.909:0.00683,(Pv11Sk00050:0.05078,(Pv04Lk05310:0.08229,Pv01Sk00470:0.05970)0.544:0.00398)0.896:0.00880)0.052:0.00015)0.777:0.00014,(Pv11Sk00060:0.04784,(Pv11Sk00070:0.02976,(Pv11Sk00080:0.03569,((Pv11Sk00090:0.01088,Pv11Sk00140:0.00329)0.968:0.00952,((Pv11Sk00150:0.00312,(Pv11Sk00110:0.00470,Pv11Sk00120:0.00312)0.881:0.00314)0.887:0.00313,(Pv11Sk00130:0.00469,(Pv11Sk00100:0.00468,Pv11Sk00160:0.00015)0.430:0.00156)0.799:0.00015)0.722:0.00174)0.952:0.00776)0.864:0.00318)0.878:0.00471)0.968:0.00950)0.904:0.00443)0.431:0.00014)0.562:0.00016,((Pv11Lk02550:0.00014,PvA11Ek00080:0.00014)1.000:0.05350,(((Pv11Lk02580:0.0,PvA11Ek00110:0.0):0.06034,(Pv11Lk02590:0.00014,PvA11Ek00120:0.00014)1.000:0.06128)0.765:0.00336,(((Pv11Lk02640:0.00014,PvA11Ek00170:0.00014)1.000:0.07433,((Pv11Lk02650:0.0,PvA11Ek00180:0.0):0.04301,(Pv11Lk02600:0.00173,PvA11Ek00130:0.00014)1.000:0.05641)0.761:0.00375)0.730:0.00140,((Pv11Lk02620:0.00014,PvA11Ek00150:0.00014)1.000:0.04853,(Pv11Lk02560:0.00014,PvA11Ek00090:0.00014)0.998:0.03441)0.971:0.01563)0.694:0.00075)0.873:0.00322)0.777:0.00492)0.835:0.00210)0.071:0.00015,(((Pv11Lk03620:0.05480,(Pv11Lk00930:0.00014,PvA11Ak00080:0.00014)1.000:0.04813)0.720:0.00210,(Pv04Lk05340:0.07953,Pv04Lk05390:0.06473)0.329:0.00536)0.417:0.00406,(Pv11Lk03640:0.06059,(Pv11Lk03590:0.02904,Pv11Lk03650:0.03064)1.000:0.02629)0.745:0.00127)0.786:0.00130)0.574:0.00014,(Pv11Lk03600:0.04860,Pv11Lk03660:0.03790)0.957:0.01339)0.634:0.00015,(Pv11Lk04920:0.09016,(((Pv11Lk05670:0.06493,(Pv11Lk05690:0.05354,(Pv11Lk05660:0.05918,Pv11Lk05640:0.06025)0.420:0.00411)0.675:0.00037)0.776:0.00244,(Pv11Lk05650:0.05466,(Pv11Lk05900:0.04263,(Pv11Lk05910:0.02363,Pv11Lk05920:0.02064)0.935:0.00862)0.870:0.00473)0.842:0.00015)0.886:0.00515,(Pv11Lk05610:0.07233,Pv11Lk05620:0.06769)0.297:0.00530)0.780:0.00262)0.769:0.00155)0.467:0.00014,((Pv01Sk01080:0.03229,((Pv01Sk01090:0.03660,Pv01Sk01070:0.03488)0.695:0.00079,(((Pv01Sk01190:0.03709,(Pv01Sk01210:0.02359,Pv01Sk01220:0.02253)0.976:0.01373)0.738:0.00254,((Pv01Sk01270:0.03281,(Pv01Sk01250:0.01966,Pv01Sk01260:0.02226)0.668:0.00964)0.759:0.00607,((Pv01Sk01230:0.04948,Pv01Sk01060:0.04249)0.836:0.00471,(Pv01Sk01200:0.02529,Pv01Sk01100:0.03766)0.719:0.00098)0.834:0.00150)0.985:0.00016)0.777:0.00187,(Pv05Sk00100:0.02860,(((((Pv05Sk00110:0.01918,Pv05Sk00130:0.02243)0.867:0.00303,(Pv05Sk00140:0.02560,Pv05Sk00160:0.02238)0.923:0.00015)0.802:0.00156,(Pv05Sk00170:0.02928,Pv05Sk00180:0.02900)0.730:0.00145)0.802:0.00156,(Pv05Sk00120:0.01820,Pv05Sk00150:0.02966)0.743:0.00092)0.870:0.00312,(Pv05Sk00190:0.03681,Pv05Sk00200:0.03576)0.919:0.00759)0.935:0.00519)0.448:0.00317)0.782:0.00112)0.893:0.00327)0.854:0.00316,((((Pv01Sk00520:0.01423,((Pv01Sk00490:0.02254,Pv01Sk00500:0.03623)0.836:0.00428,(Pv01Sk00480:0.01470,Pv01Sk00510:0.02568)0.354:0.00308)0.735:0.00181)0.968:0.00786,((((((Pv01Sk01700:0.02234,Pv01Sk01710:0.03970)0.342:0.00428,Pv01Sk01810:0.03325)0.623:0.00016,(Pv01Sk01750:0.03335,Pv01Sk01770:0.02440)0.953:0.01284)0.747:0.00211,(Pv01Sk01760:0.03229,Pv01Sk01790:0.05272)0.797:0.00350)0.752:0.00132,(Pv01Sk01720:0.02096,Pv01Sk01730:0.03441)0.991:0.01789)0.870:0.00413,(Pv01Sk01800:0.02849,Pv01Sk01740:0.05171)0.653:0.00040)0.895:0.00378)0.769:0.00016,((((Pv01Sk01620:0.04334,(Pv01Sk01650:0.01609,(Pv01Sk01630:0.01550,Pv01Sk01640:0.02254)0.860:0.00693)0.960:0.01121)0.977:0.01266,Pv01Sk01610:0.02549)0.764:0.00164,(Pv01Sk01580:0.03336,Pv01Sk01600:0.03494)0.942:0.01602)0.976:0.00015,Pv01Sk01590:0.04071)0.810:0.00157)0.942:0.00464,Pv05Sk00090:0.09309)0.129:0.00014)0.864:0.00157)1.000:0.00014)0.828:0.00235,((Pv11Lk04770:0.05442,(Pv11Lk04750:0.05954,Pv11Lk04760:0.05891)0.783:0.00916)0.949:0.01283,(Pv11Lk05600:0.08524,(Pv04Sk03600:0.04195,Pv04Sk03620:0.04159)0.993:0.02932)0.892:0.01419)0.501:0.00240)0.796:0.00200,((Pv11Lk04880:0.08076,((((((((Pv10Lk02370:0.02887,Pv10Lk02390:0.04147)0.992:0.02022,((((Pv10Lk02060:0.04142,(Pv10Lk02080:0.04897,(Pv10Lk02070:0.05156,Pv10Lk02150:0.04154)0.832:0.00741)0.889:0.00639)0.878:0.00730,(Pv10Lk02210:0.05605,Pv10Lk02230:0.05610)0.860:0.00788)0.863:0.00346,(((Pv10Lk02200:0.07744,(Pv10Lk02010:0.05052,Pv10Lk02030:0.05773)0.777:0.00717)0.808:0.00328,(Pv10Lk02330:0.04677,Pv10Lk02340:0.04189)0.987:0.01950)0.966:0.00016,((Pv10Lk02440:0.04874,(Pv10Lk02510:0.06752,(Pv10Lk02550:0.06672,Pv10Lk02540:0.05723)0.088:0.00310)0.803:0.00458)0.380:0.00455,(((Pv10Lk02000:0.07262,Pv10Lk02220:0.06445)0.907:0.01133,Pv10Lk02530:0.05964)0.777:0.00409,((Pv10Lk02400:0.05305,(Pv10Lk02360:0.01359,Pv10Lk02380:0.02535)1.000:0.04117)0.859:0.00728,((Pv10Lk02460:0.04951,Pv10Lk02480:0.03184)0.984:0.02714,(Pv10Lk02490:0.04705,(Pv10Lk02450:0.02433,Pv10Lk02470:0.01784)0.998:0.03420)0.828:0.00810)0.854:0.00721)0.793:0.00277)0.875:0.00160)1.000:0.00015)0.923:0.00015)0.913:0.00155,(((Pv10Lk02270:0.06720,Pv10Lk02500:0.06183)0.041:0.00148,Pv10Lk02410:0.10316)0.311:0.00015,Pv10Lk02430:0.07267)0.605:0.00015)0.758:0.00162)0.781:0.00161,((Pv11Lk00550:0.07933,(Pv10Lk02240:0.05238,Pv10Lk02290:0.06732)0.799:0.00748)0.745:0.00337,(Pv10Lk01990:0.06665,Pv11Lk02010:0.05787)0.774:0.00613)0.762:0.00176)0.792:0.00157,(((((((Pv10Lk00350:0.06155,((Pv10Lk00320:0.05532,Pv10Lk00340:0.05955)0.801:0.01251,(Pv10Lk00290:0.06813,(Pv10Lk00310:0.05176,(Pv10Lk00300:0.06995,Pv10Lk00330:0.06160)0.825:0.00772)0.880:0.00587)0.863:0.00661)0.579:0.00324)0.848:0.00016,(Pv10Lk00240:0.07243,((Pv10Lk00360:0.05983,Pv10Lk00250:0.08189)0.855:0.00014,(Pv10Lk00260:0.06984,Pv10Lk00280:0.07206)0.674:0.00184)0.770:0.00160)0.857:0.00329)0.971:0.00626,((Pv03Sk00170:0.08475,((((Pv03Sk00190:0.00812,Pv03Sk00230:0.00942)1.000:0.04914,(Pv03Sk00210:0.03000,(Pv03Sk00220:0.04665,(Pv03Sk00200:0.01069,Pv03Sk00240:0.02481)1.000:0.02872)0.933:0.01390)0.507:0.01083)0.998:0.02864,(Pv03Sk00150:0.07753,Pv03Sk00180:0.07151)0.816:0.01135)0.585:0.00106,Pv03Sk00160:0.05857)0.699:0.00279)0.996:0.02411,(((((((Pv08Lk00920:0.08307,((Pv08Lk00910:0.03534,((Pv08Lk00840:0.03356,Pv08Lk00850:0.02477)0.965:0.01393,(((Pv08Lk00870:0.02832,Pv08Lk00890:0.03008)0.909:0.01054,Pv08Lk00900:0.02407)0.555:0.00348,(Pv08Lk00880:0.02577,Pv08Lk00860:0.03915)0.858:0.00429)0.786:0.00576)0.936:0.01037)0.971:0.01432,(Pv08Lk00930:0.09712,Pv08Lk00940:0.13674)0.866:0.01715)0.134:0.00628)0.923:0.01161,Pv11Lk05290:0.09007)0.880:0.00773,(Pv10Lk01150:0.08597,(((Pv10Lk01090:0.04393,Pv10Lk01110:0.06110)0.989:0.02755,(Pv10Lk01140:0.06448,Pv10Lk01120:0.09305)0.680:0.00287)0.233:0.00756,(Pv10Lk01070:0.09144,(Pv10Lk01080:0.05606,Pv10Lk01100:0.04151)0.945:0.01610)0.853:0.00883)0.746:0.00016)0.931:0.00545)1.000:0.00015,((((((Pv10Lk00780:0.07540,Pv10Lk00820:0.05427)0.187:0.00654,Pv10Lk00770:0.07996)0.768:0.00354,(Pv10Lk00810:0.08047,(Pv10Lk00790:0.05361,Pv10Lk00800:0.04721)0.896:0.01156)0.963:0.00015)0.182:0.00157,((Pv10Lk00870:0.06645,(Pv10Lk00850:0.02734,Pv10Lk00860:0.04437)0.983:0.01941)0.746:0.00229,(Pv10Lk00830:0.05210,Pv10Lk00840:0.03283)0.857:0.00592)0.889:0.00314)0.990:0.01229,((((Pv11Lk02750:0.00014,PvA11Ek00270:0.00014)1.000:0.07544,(Pv11Lk02760:0.00014,PvA11Ek00280:0.00014)1.000:0.06469)0.988:0.02662,(Pv04Sk02050:0.05695,Pv04Sk02060:0.07315)0.335:0.00476)0.478:0.00375,Pv04Sk02040:0.04311)0.796:0.00015)0.868:0.00322,(Pv04Sk00750:0.06795,(Pv04Sk00740:0.13111,Pv04Sk00760:0.05502)0.549:0.01052)0.897:0.00841)0.805:0.00486)0.788:0.00162,(((Pv11Lk04810:0.05487,(Pv11Lk04830:0.03467,Pv11Lk04860:0.05064)0.998:0.03096)0.852:0.00642,((Pv11Lk04800:0.02696,Pv11Lk04820:0.02646)1.000:0.03723,(Pv11Lk04840:0.04853,Pv11Lk04850:0.08303)0.038:0.01159)0.720:0.00199)0.986:0.01464,((Pv11Lk04180:0.06691,Pv10Lk00410:0.04183)0.726:0.00192,((((Pv05Lk02200:0.05418,(Pv05Lk02210:0.06066,Pv05Lk02220:0.06600)0.860:0.00885)0.994:0.02612,(Pv09Lk00140:0.06949,((Pv10Lk00400:0.08410,Pv10Lk00580:0.09915)0.768:0.00620,(Pv10Lk01020:0.10851,((((((((Pv10Sk04800:0.07196,Pv11Lk05230:0.10946)0.926:0.01827,(Pv01Sk00620:0.09652,(Pv01Sk00610:0.07541,(Pv01Sk00570:0.06544,Pv01Sk00710:0.08173)0.754:0.00481)0.798:0.00437)0.888:0.00713)0.400:0.00015,(Pv05Sk00210:0.11046,(Pv05Lk02190:0.08817,Pv04Sk00770:0.08481)0.305:0.00772)0.848:0.00874)0.799:0.00156,((((Pv10Lk04380:0.09487,(Pv04Lk06000:0.09396,Pv04Lk06040:0.10782)0.042:0.00358)0.975:0.01747,(((Pv02Sk00440:0.07954,Pv02Sk00470:0.10979)0.830:0.00902,((Pv10Sk05450:0.10186,((Pv10Sk05470:0.07997,(Pv02Sk00320:0.03905,((Pv02Sk00170:0.03631,Pv02Sk00180:0.03905)0.866:0.00582,(((((Pv02Sk00120:0.01785,Pv02Sk00130:0.01190)0.987:0.01695,(Pv02Sk00150:0.04529,Pv02Sk00190:0.03829)0.687:0.00147)0.753:0.00164,Pv02Sk00230:0.03466)0.627:0.00015,((Pv02Sk00090:0.02969,(Pv02Sk00110:0.04931,(Pv02Sk00100:0.04518,Pv02Sk00160:0.03547)0.839:0.00720)0.686:0.00129)0.030:0.00155,(Pv02Sk00210:0.03293,Pv02Sk00200:0.02742)0.888:0.00537)0.872:0.00016)0.692:0.00015,(((Pv02Sk00220:0.02320,Pv02Sk00240:0.02594)0.989:0.01663,(Pv02Sk00070:0.04451,Pv02Sk00080:0.02761)0.472:0.00381)0.770:0.00021,(Pv02Sk00140:0.05039,Pv10Sk05480:0.08920)0.956:0.01800)1.000:0.00014)0.754:0.00153)0.854:0.00404)0.786:0.00414)0.890:0.00501,((Pv08Lk00720:0.04713,Pv08Lk00730:0.07451)0.847:0.01633,(Pv01Lk02180:0.05714,((Pv01Lk02150:0.02097,Pv01Lk02160:0.01390)0.993:0.01768,Pv01Lk02190:0.03847)0.392:0.00119)0.994:0.02206)0.519:0.00630)0.756:0.00184)0.752:0.00211,(Pv02Sk00480:0.07493,(((Pv07Lk00400:0.04149,Pv07Lk00420:0.03850)1.000:0.05382,(Pv07Lk00320:0.02814,Pv07Lk00340:0.05311)0.811:0.02651)0.941:0.01756,((Pv07Lk00380:0.06864,(Pv07Lk00430:0.04119,Pv07Lk00460:0.03978)0.909:0.01403)0.956:0.02046,(Pv07Lk00350:0.09414,(Pv07Lk00310:0.02068,Pv07Lk00330:0.03348)0.987:0.02422)0.133:0.00847)0.395:0.00584)0.804:0.00243)0.673:0.00168)0.896:0.00356)0.234:0.00014,Pv02Sk00460:0.07264)0.974:0.01550)0.815:0.00344,(Pv03Lk00890:0.10720,(Pv04Lk06220:0.11265,(Pv04Lk06230:0.08892,Pv04Lk06710:0.11484)0.841:0.01088)0.928:0.01575)0.763:0.00342)0.918:0.00370,(((Pv10Lk01930:0.09040,(Pv10Lk01960:0.05643,(Pv10Lk01940:0.05390,Pv10Lk01950:0.06535)0.653:0.00770)0.810:0.00404)0.738:0.00179,Pv10Lk01970:0.07279)0.772:0.00225,(Pv10Lk01900:0.05552,Pv10Lk01910:0.06981)0.709:0.00191)0.988:0.01261)0.990:0.00014)0.857:0.00200,((((Pv02Sk00390:0.04351,Pv02Sk00400:0.04744)1.000:0.05790,((((Pv10Lk04360:0.10099,(Pv10Lk04350:0.08190,Pv10Lk04370:0.07367)0.288:0.00741)0.915:0.01031,Pv09Lk00130:0.06435)0.790:0.00290,(Pv08Lk01160:0.10049,(Pv10Lk00370:0.10039,(Pv10Lk00380:0.06541,Pv10Lk00390:0.05176)0.939:0.01461)0.893:0.00876)0.050:0.00144)0.856:0.00515,(Pv09Lk00110:0.11791,(Pv09Lk00120:0.14149,(Pv09Ck00010:0.12015,((Pv09Lk00030:0.09668,Pv09Lk00040:0.09202)0.946:0.02251,((((Pv11Lk05550:0.07568,Pv11Lk05560:0.10533)0.924:0.02541,(Pv11Lk05540:0.08698,Pv11Lk05530:0.10783)0.602:0.00474)0.558:0.00618,((Pv01Sk00250:0.12017,((Pv02Lk00960:0.11245,(Pv02Lk00950:0.08967,Pv02Lk00970:0.12103)0.725:0.01576)0.606:0.01159,Pv02Lk00930:0.13314)0.076:0.00174)0.794:0.01723,((Pv09Lk00080:0.08068,(Pv09Lk00090:0.07278,Pv09Lk00100:0.07590)0.846:0.01399)0.943:0.01414,Pv09Lk00060:0.09828)0.704:0.00712)0.995:0.02833)0.990:0.02011,((((Pv11Lk05190:0.05425,Pv11Lk05210:0.05297)0.856:0.00955,(Pv11Lk05220:0.05577,Pv11Lk05200:0.06032)0.388:0.01138)0.999:0.03839,((Pv04Sk01260:0.06169,(Pv04Sk01250:0.07329,Pv04Sk01350:0.07863)0.718:0.00970)1.000:0.04349,(Pv07Sk00190:0.07722,((Pv07Sk00200:0.06475,(Pv07Sk00220:0.07455,Pv07Sk00210:0.02653)0.975:0.03703)0.847:0.01228,(Pv07Sk00150:0.06778,Pv07Sk00160:0.07654)0.581:0.01196)0.784:0.01118)1.000:0.05262)0.748:0.00130)0.834:0.00441,((Pv04Lk04210:0.07623,(Pv04Lk04190:0.09391,Pv04Lk04200:0.06947)0.229:0.00374)0.869:0.00661,(((Pv08Ck00240:0.07587,(((Pv10Ck04790:0.05805,((Pv07Ck00250:0.06754,Pv07Ck00260:0.06533)0.789:0.00037,(Pv02Lk00500:0.03352,(Pv07Ck00240:0.06440,Pv02Lk00490:0.05111)0.120:0.00281)0.751:0.00164)0.762:0.00152)0.908:0.00469,((((Pv03Lk00870:0.04862,Pv07Ck00270:0.07569)0.812:0.00720,Pv07Ck00230:0.05795)0.748:0.00168,Pv07Ck00280:0.11855)0.773:0.00170,((Pv03Lk00750:0.07280,Pv07Ck00290:0.08770)0.916:0.01251,((((Pv03Lk00830:0.04913,Pv03Lk00860:0.05120)0.990:0.02642,(Pv03Lk00780:0.06927,(Pv03Ck00680:0.09805,Pv03Lk00790:0.05378)0.866:0.01133)0.729:0.00187)0.884:0.00626,(Pv03Ck00690:0.06419,(Pv03Ck00670:0.06047,Pv03Lk00710:0.06901)0.475:0.00367)0.807:0.00015)0.777:0.00224,(Pv03Lk00880:0.05465,Pv03Lk00800:0.07296)0.641:0.00529)0.763:0.00269)0.882:0.00733)0.838:0.00015)0.955:0.00600,(((((Pv06Lk00170:0.08093,Pv06Lk00110:0.08154)0.376:0.01526,(((Pv06Lk00060:0.06120,(Pv06Lk00020:0.07659,((Pv06Lk00010:0.06972,Pv06Lk00030:0.05870)0.904:0.01043,Pv06Lk00050:0.04449)0.674:0.00072)0.913:0.00652)0.836:0.00329,((((Pv02Lk00690:0.04464,Pv02Lk00710:0.06470)0.990:0.02216,(Pv02Lk00700:0.06355,Pv02Lk00730:0.07449)0.953:0.01611)0.838:0.00509,((Pv02Lk00820:0.06293,(Pv02Lk00810:0.07986,Pv02Lk00840:0.05841)0.847:0.01270)0.797:0.00376,((Pv02Lk00760:0.08901,(Pv02Lk00740:0.04958,Pv02Lk00750:0.05261)0.874:0.01624)0.834:0.00447,(((Pv02Lk00830:0.05488,Pv02Lk00850:0.04069)0.956:0.01431,Pv02Lk00580:0.06270)0.619:0.00177,(Pv02Lk00800:0.04684,Pv02Lk00860:0.07201)0.947:0.01488)0.258:0.00179)0.870:0.00355)0.861:0.00316)0.734:0.00154,((Pv06Lk00120:0.06942,(Pv02Lk00570:0.06273,(Pv02Lk00630:0.03670,(Pv02Lk00590:0.02791,Pv02Lk00600:0.03532)0.854:0.00738)1.000:0.03738)0.840:0.00638)0.713:0.00193,(Pv02Lk00720:0.06765,Pv06Lk00160:0.05533)0.811:0.00610)0.847:0.00309)0.890:0.00016)0.762:0.00176,(Pv06Lk00180:0.07512,Pv02Lk00790:0.05613)0.532:0.00726)0.765:0.00748)0.891:0.00015,(Pv04Ck04100:0.07909,(Pv04Ck04110:0.03475,Pv04Ck04120:0.07011)0.962:0.01929)0.869:0.00674)0.996:0.00065,(Pv04Ck04180:0.06320,Pv08Ck00120:0.09607)0.932:0.01808)0.980:0.00092,((((Pv08Ck00110:0.06021,(Pv08Ck00280:0.02507,(Pv08Ck00310:0.02867,Pv08Ck00370:0.01465)0.978:0.01728)1.000:0.04436)0.169:0.00509,Pv08Ck00460:0.06290)0.701:0.00244,((((((Pv08Ck00180:0.06796,(Pv08Ck00130:0.04936,Pv08Ck00200:0.07432)0.307:0.00823)0.419:0.00585,((Pv08Ck00230:0.07403,Pv09Ck00020:0.05389)0.890:0.00952,(Pv08Ck00220:0.07417,(Pv08Ck00150:0.09509,Pv08Ck00170:0.05440)0.946:0.01904)0.938:0.01280)0.741:0.00160)0.807:0.00468,(Pv08Ck00330:0.07049,(Pv08Ck00390:0.03783,(Pv08Ck00250:0.03865,Pv08Ck00340:0.04787)0.779:0.00387)0.925:0.01096)0.997:0.02099)0.741:0.00220,(((Pv08Ck00190:0.08271,Pv08Ck00630:0.05171)0.741:0.00840,(Pv08Ck00410:0.02760,Pv08Ck00440:0.04158)1.000:0.03750)0.801:0.00502,(Pv08Ck00140:0.06010,(Pv08Ck00160:0.05886,Pv08Ck00210:0.05593)0.784:0.00792)0.995:0.02401)0.035:0.00016)0.840:0.00163,(Pv08Ck00620:0.06479,(Pv08Ck00600:0.03721,Pv08Ck00610:0.04478)0.936:0.01032)0.920:0.00733)0.711:0.00015,((Pv08Ck00260:0.04088,Pv08Ck00350:0.04470)1.000:0.04446,((Pv06Lk00150:0.08081,((Pv08Ck00300:0.06673,(Pv08Ck00270:0.04664,Pv08Ck00360:0.04914)0.648:0.00668)0.918:0.00882,(Pv08Ck00290:0.05478,(Pv08Ck00320:0.03168,Pv08Ck00380:0.02634)0.908:0.01438)0.993:0.02451)0.530:0.00412)0.507:0.00015,(Pv08Ck00500:0.07254,(Pv06Lk00070:0.06683,Pv08Ck00520:0.07151)0.562:0.00523)0.894:0.00531)0.827:0.00147)0.728:0.00011)0.778:0.00208)0.772:0.00190,((Pv02Lk00680:0.09035,Pv08Ck00590:0.06948)0.918:0.01547,((((Pv04Ck04060:0.06854,Pv04Ck04090:0.05137)1.000:0.05883,Pv08Ck00580:0.06630)0.680:0.00152,(Pv04Ck04140:0.08156,((Pv04Ck04160:0.07554,Pv04Ck04170:0.07757)0.882:0.00887,(Pv04Ck04070:0.04026,Pv04Ck04080:0.05409)0.989:0.01921)0.813:0.00306)0.852:0.00338)0.822:0.00311,(((((Pv04Ck04130:0.04530,(Pv08Ck00540:0.06314,Pv08Ck00560:0.11730)0.018:0.00774)0.922:0.01170,((Pv02Lk00530:0.04559,((Pv02Lk00520:0.02110,Pv02Lk00550:0.02682)0.983:0.01662,(Pv02Lk00540:0.02771,(Pv02Lk00510:0.02455,Pv02Lk00560:0.03780)0.264:0.00270)0.428:0.00194)0.875:0.00646)1.000:0.04477,Pv04Ck04150:0.07654)0.640:0.00282)0.761:0.00015,(Pv08Ck00470:0.06308,Pv08Ck00510:0.07467)0.899:0.01013)0.916:0.00146,(Pv08Ck00530:0.06785,(Pv08Ck00550:0.05005,Pv08Ck00570:0.04568)0.985:0.02396)0.956:0.01161)1.000:0.00011,(Pv08Ck00450:0.08328,(Pv08Ck00430:0.08801,Pv06Lk00140:0.09607)0.806:0.00747)0.816:0.00487)0.748:0.00142)0.233:0.00158)0.744:0.00015)0.119:0.00015)0.023:0.00016)0.322:0.00181)0.778:0.00163,((Pv08Ck00420:0.09169,Pv08Ck00490:0.05967)0.577:0.01317,Pv08Ck00480:0.09554)0.958:0.00014)0.986:0.00015,Pv03Lk00730:0.09462)0.891:0.01552)1.000:0.02806)0.977:0.01303)0.397:0.00621)0.539:0.00877)0.722:0.00331)0.749:0.02208)0.897:0.01907)0.481:0.00454)0.847:0.00591,Pv03Sk00600:0.10123)0.850:0.00479,((Pv10Lk00890:0.10081,(Pv10Lk01040:0.05526,(Pv10Lk00880:0.05422,Pv10Lk01030:0.05507)0.819:0.00873)0.839:0.00568)0.910:0.00834,(Pv10Lk01060:0.08494,(Pv10Lk00650:0.05708,(Pv10Lk00550:0.08886,Pv10Lk00570:0.08949)0.850:0.01124)0.833:0.00553)0.908:0.00847)0.773:0.00205)0.656:0.00014)0.587:0.00015,Pv11Lk06050:0.12194)0.920:0.00644,(Pv03Sk00640:0.06880,(Pv04Sk00730:0.10663,Pv08Lk00830:0.13991)0.576:0.01030)0.915:0.01413)0.196:0.00372,(Pv01Sk01540:0.04374,((Pv01Sk01550:0.05809,(Pv01Sk01660:0.04329,Pv01Sk01690:0.01980)1.000:0.04396)0.931:0.01319,(Pv01Sk01670:0.06463,Pv01Sk01680:0.05748)0.481:0.00584)0.746:0.00202)0.998:0.01810)0.730:0.00249)0.874:0.00710)0.871:0.00626)0.763:0.00381)0.797:0.00430,(Pv11Lk05280:0.07144,(((Pv11Lk03850:0.07063,((Pv11Lk00270:0.06441,(Pv11Lk00210:0.04365,Pv11Lk00260:0.03238)1.000:0.03923)0.089:0.00016,((Pv11Lk00200:0.03652,Pv11Lk00250:0.05122)1.000:0.03809,((Pv11Lk00280:0.05054,(Pv11Lk00190:0.04501,Pv11Lk00240:0.03558)0.963:0.01686)0.990:0.01967,(Pv11Lk00170:0.03780,Pv11Lk00220:0.04168)0.996:0.03813)0.693:0.00160)0.761:0.00273)0.946:0.00517)0.232:0.00016,((Pv11Lk01230:0.00014,PvA11Ck00030:0.00014)1.000:0.08126,((((((Pv11Lk01350:0.00014,(PvA11Ck00130:0.0,PvA11Ck00170:0.0,PvA11Ck00210:0.0,PvA11Ck00250:0.0):0.00014)1.000:0.05282,(Pv11Lk01310:0.00014,PvA11Ck00090:0.00014)1.000:0.07323)0.951:0.01374,(Pv11Lk01430:0.00014,PvA11Ck00340:0.00014)1.000:0.07784)0.721:0.00054,((((((((((Pv11Lk01450:0.00014,PvA11Ck00360:0.00014)1.000:0.04379,((PvA11Ck00120:0.0,PvA11Ck00160:0.0,PvA11Ck00200:0.0,PvA11Ck00240:0.0):0.01992,(Pv11Lk01340:0.00015,PvA11Ck00110:0.03750)0.997:0.02854)1.000:0.04354)0.880:0.00741,((PvA11Ck00190:0.00014,(Pv11Lk01370:0.00014,(PvA11Ck00150:0.0,PvA11Ck00230:0.0,PvA11Ck00270:0.0):0.00014)0.971:0.00787)1.000:0.04762,(Pv11Lk01290:0.00014,PvA11Ck00070:0.00015)1.000:0.06118)0.366:0.00232)0.377:0.00016,(Pv11Lk01270:0.00014,PvA11Ck00050:0.00160)1.000:0.05976)0.782:0.00157,Pv11Lk01320:0.06997)0.753:0.00136,(((Pv11Lk01300:0.0,PvA11Ck00080:0.0):0.04716,(Pv11Lk01280:0.00014,PvA11Ck00060:0.00014)1.000:0.04123)0.970:0.01722,((Pv11Lk01390:0.00014,PvA11Ck00300:0.00014)1.000:0.06174,(Pv11Lk01440:0.00014,(PvA11Ck00280:0.0,PvA11Ck00350:0.0):0.00014)1.000:0.05250)0.973:0.01870)0.804:0.00323)0.107:0.00015,(Pv11Lk01410:0.00014,PvA11Ck00320:0.00014)1.000:0.06909)0.780:0.00162,((PvA11Ck00140:0.0,PvA11Ck00180:0.0,PvA11Ck00220:0.0):0.00014,(Pv11Lk01360:0.00014,PvA11Ck00260:0.00014)0.822:0.00157)1.000:0.05011)0.748:0.00148,(Pv11Lk01420:0.00014,PvA11Ck00330:0.00015)1.000:0.07812)0.794:0.00171,((Pv11Lk01400:0.00014,PvA11Ck00310:0.00014)1.000:0.08005,Pv11Lk01260:0.04431)0.951:0.00015)0.807:0.00164)0.937:0.00015,((Pv11Lk01380:0.05973,(Pv11Lk01240:0.00014,PvA11Ck00040:0.00015)1.000:0.08212)0.840:0.00769,(Pv11Lk01250:0.09324,Pv11Lk01330:0.05420)0.691:0.00481)0.742:0.00118)0.943:0.00801,(Pv11Lk01200:0.00014,PvA11Ck00010:0.00014)1.000:0.07683)0.816:0.00418)0.939:0.00904)0.773:0.00206,(Pv11Lk00180:0.02343,Pv11Lk00230:0.02390)1.000:0.05524)0.785:0.00349)0.747:0.00263)0.734:0.00016,(((((Pv11Lk00840:0.00015,PvA11Ak00010:0.00014)1.000:0.08509,(Pv11Lk04600:0.06813,(Pv11Lk00850:0.00014,PvA11Ak00020:0.00014)1.000:0.07888)0.604:0.00691)0.846:0.00547,((Pv11Lk01010:0.0,PvA11Ak00200:0.0,PvA11Ak00210:0.0):0.07212,((Pv11Lk00860:0.00014,PvA11Ak00030:0.00014)1.000:0.05973,((Pv11Lk01000:0.0,PvA11Ak00180:0.0,PvA11Ak00190:0.0):0.04000,((Pv11Lk00990:0.00014,(PvA11Ak00160:0.0,PvA11Ak00170:0.0):0.00015)0.999:0.02180,(Pv11Lk00980:0.00014,(PvA11Ak00140:0.0,PvA11Ak00150:0.0):0.00015)0.998:0.01817)0.815:0.00340)0.989:0.02413)0.998:0.03841)0.357:0.00599)0.746:0.00380,PvA11Ak00040:0.06272)0.934:0.00911,((Pv05Lk01960:0.08036,((Pv11Lk02670:0.00014,PvA11Ek00200:0.00014)1.000:0.07013,(Pv11Lk02660:0.00014,PvA11Ek00190:0.00015)1.000:0.04917)0.971:0.02160)0.886:0.01038,(Pv10Lk01130:0.09004,Pv11Lk03070:0.05255)0.891:0.01378)0.813:0.00476)0.737:0.00193)0.394:0.00015)0.078:0.00013)0.304:0.00012)0.902:0.00011,((Pv11Lk05250:0.04825,Pv11Lk05260:0.08832)0.978:0.02376,(((((Pv11Lk02500:0.00015,PvA11Ek00030:0.00014)1.000:0.04437,((Pv11Lk02490:0.00014,PvA11Ek00020:0.00014)0.997:0.03144,(Pv11Lk02530:0.00014,PvA11Ek00060:0.00014)1.000:0.04779)0.967:0.02225)0.894:0.01889,((Pv11Lk02480:0.00014,PvA11Ek00010:0.00014)1.000:0.05642,((Pv11Lk02540:0.00016,PvA11Ek00070:0.00014)1.000:0.07215,(Pv11Lk02520:0.00014,PvA11Ek00050:0.00014)1.000:0.05470)0.897:0.01488)0.747:0.00505)0.933:0.01112,(Pv10Lk00900:0.07227,Pv10Lk00930:0.06820)0.343:0.00680)0.702:0.00110,((((Pv01Sk00220:0.02751,((Pv01Sk00140:0.01945,Pv01Sk00190:0.01617)0.880:0.00463,(Pv01Sk00160:0.00473,(Pv01Sk00130:0.01560,(Pv01Sk00150:0.01215,Pv01Sk00200:0.01314)0.921:0.00661)0.925:0.00509)0.928:0.00475)0.864:0.00322)0.747:0.00145,(Pv01Sk00210:0.01735,(Pv01Sk00230:0.04026,Pv01Sk00240:0.03179)0.967:0.01094)0.740:0.00178)0.980:0.00972,((((Pv10Lk00940:0.04271,Pv10Lk01000:0.03920)0.753:0.00126,(Pv10Lk00990:0.03857,Pv10Lk00980:0.04536)0.871:0.00014)0.774:0.00161,(Pv10Lk00950:0.06336,Pv10Lk01010:0.04840)0.666:0.00231)0.721:0.00014,(Pv10Lk00960:0.06320,Pv10Lk00970:0.03452)0.711:0.00100)0.911:0.00482)0.981:0.01025,((Pv01Sk01310:0.07131,Pv01Sk01300:0.05350)0.644:0.01227,((Pv01Sk01290:0.06083,Pv01Sk01320:0.06232)0.828:0.01487,(Pv01Sk01330:0.05254,Pv01Sk01280:0.04906)0.769:0.00628)0.441:0.00623)0.713:0.00172)0.784:0.00168)0.172:0.00333)0.483:0.00015)0.534:0.00014,((Pv01Sk01150:0.06712,Pv01Sk01160:0.04236)0.970:0.01753,(Pv01Sk01170:0.10492,((((((((Pv05Sk00900:0.05652,(Pv05Sk00930:0.02161,Pv05Sk00940:0.03441)0.801:0.01094)0.858:0.00504,(((Pv05Sk00950:0.03753,(Pv05Sk00910:0.02101,Pv05Sk00920:0.01687)0.978:0.01549)0.888:0.00538,(Pv05Sk00970:0.04161,Pv05Sk00960:0.05667)0.845:0.00559)0.805:0.00283,Pv05Sk00890:0.04599)0.322:0.00015)0.913:0.00326,((Pv05Sk00850:0.03462,(Pv05Sk00830:0.03959,(Pv05Sk00840:0.02865,Pv05Sk00870:0.03328)0.715:0.00247)0.839:0.00418)0.746:0.00137,(((Pv05Sk00770:0.01644,Pv05Sk00780:0.02417)0.869:0.00494,(Pv05Sk00800:0.01905,Pv05Sk00860:0.04770)0.868:0.00580)0.775:0.00184,(Pv05Sk00740:0.03237,(Pv05Sk00760:0.06237,(Pv05Sk00750:0.04517,Pv05Sk00790:0.02634)0.962:0.01251)0.371:0.00284)0.659:0.00015)0.779:0.00184)0.775:0.00160)0.456:0.00016,Pv05Sk00980:0.06806)0.992:0.01294,((Pv05Sk00220:0.04939,((Pv05Sk00600:0.05423,(((Pv05Sk00580:0.05228,Pv05Sk00590:0.07532)0.686:0.00197,(Pv05Sk00380:0.04729,Pv05Sk00390:0.04916)0.387:0.00565)0.733:0.00014,((Pv05Sk00620:0.04222,(Pv05Sk00610:0.04047,Pv05Sk00640:0.04301)0.638:0.00273)0.275:0.00157,Pv05Sk00630:0.04796)0.779:0.00165)0.850:0.00308)0.952:0.00634,(Pv05Sk00280:0.04302,Pv05Sk00290:0.01855)0.991:0.02052)0.737:0.00159)0.694:0.00330,((Pv05Sk01540:0.06935,(Pv05Sk01530:0.04319,Pv05Sk01550:0.08244)0.846:0.00807)0.793:0.00299,(Pv05Sk01420:0.04901,(Pv05Sk01410:0.06392,Pv05Sk01430:0.04850)0.382:0.00722)0.714:0.00224)0.943:0.00801)0.139:0.00014)0.728:0.00015,((Pv05Sk01560:0.08780,Pv05Sk01390:0.07680)0.500:0.00015,(Pv05Sk01400:0.07302,Pv05Sk01380:0.05705)0.929:0.01124)0.913:0.00497)0.970:0.00874,(((((Pv11Lk03890:0.04390,(Pv11Lk03880:0.02614,Pv11Lk03900:0.02258)0.973:0.01248)0.839:0.00374,((Pv11Lk03910:0.04389,Pv11Lk03920:0.04707)0.747:0.00421,Pv11Lk03870:0.04089)0.752:0.00143)0.701:0.00144,Pv11Lk03930:0.04602)0.921:0.00478,((Pv11Lk03960:0.06090,Pv11Lk03970:0.05385)0.716:0.00303,(Pv11Lk04190:0.04747,(Pv11Lk04000:0.06287,(Pv11Lk03980:0.04494,Pv11Lk03990:0.03410)0.835:0.01445)0.796:0.00392)0.751:0.00016)0.864:0.00315)0.964:0.00788,(((((Pv11Lk04490:0.01775,Pv11Lk04510:0.02413)0.969:0.01267,((Pv11Lk04520:0.03077,(Pv11Lk04480:0.03279,Pv11Lk04460:0.02968)0.048:0.00279)0.733:0.00187,((Pv11Lk04550:0.01795,Pv11Lk04540:0.04394)0.918:0.00914,(Pv11Lk04470:0.02632,Pv11Lk04500:0.02278)0.746:0.00436)0.863:0.00015)1.000:0.00015)0.920:0.00145,(Pv11Lk04450:0.02887,(Pv11Lk04440:0.02718,Pv11Lk04430:0.03273)0.847:0.00451)0.781:0.00224)0.982:0.00859,((Pv11Lk05310:0.04648,Pv11Lk05300:0.06682)0.736:0.00680,Pv11Lk05360:0.05453)0.478:0.00231)0.826:0.00327,Pv11Lk05330:0.05450)0.967:0.01044)0.751:0.00155)0.811:0.00176,((Pv05Sk01090:0.06849,(Pv07Sk00100:0.04088,Pv07Sk00120:0.03467)0.997:0.03615)0.814:0.00651,(Pv01Sk01140:0.06593,Pv05Sk01100:0.04502)0.864:0.00761)0.291:0.00212)0.948:0.00014)0.105:0.00170)0.965:0.00707)0.774:0.00211)0.765:0.00224)0.774:0.00219,(((((Pv11Lk03940:0.09202,(PvA11Bk00140:0.07455,PvA11Bk00170:0.06624)0.964:0.02504)0.642:0.00321,((Pv11Lk01630:0.0,PvA11Dk00120:0.0):0.07556,Pv11Lk03860:0.06628)0.531:0.00900)0.744:0.00255,(((Pv04Lk05500:0.03839,Pv04Lk05530:0.05119)0.715:0.00149,(Pv04Lk05490:0.01459,Pv04Lk05520:0.01974)0.993:0.01932)0.313:0.00338,Pv04Lk05510:0.03344)0.999:0.02142)0.771:0.00165,(((Pv04Sk03260:0.06158,Pv11Lk04300:0.04700)0.925:0.01053,(Pv11Lk04280:0.07203,(Pv11Lk02440:0.08823,(Pv11Lk02430:0.06038,Pv11Lk02450:0.06411)0.933:0.02022)0.787:0.01090)0.691:0.00148)0.778:0.00263,((Pv04Lk04430:0.07365,((((Pv04Lk06510:0.01955,(Pv04Lk06500:0.03213,(Pv04Lk06530:0.00948,Pv04Lk06540:0.01430)0.930:0.00627)0.535:0.00014)0.762:0.00159,((Pv04Lk06470:0.00947,(Pv04Lk06460:0.03221,(Pv04Lk06490:0.01720,Pv04Lk06520:0.01855)0.989:0.01539)0.458:0.00353)0.702:0.00016,Pv04Lk06480:0.01428)0.799:0.00314)0.946:0.00707,(Pv04Lk06560:0.03349,Pv04Lk06550:0.05180)0.407:0.00322)0.999:0.01884,((Pv04Lk04890:0.03190,Pv04Lk04900:0.04833)0.960:0.01209,(Pv04Lk05970:0.06597,(Pv04Lk05950:0.05690,Pv04Lk05960:0.03970)0.418:0.00413)0.652:0.00365)0.737:0.00149)0.867:0.00339)0.548:0.00344,Pv11Lk04420:0.08591)0.919:0.00612)0.864:0.00015)0.877:0.00156,(((Pv11Lk01190:0.07561,(PvA11Bk00390:0.09073,(PvA11Bk00400:0.06823,(PvA11Bk00420:0.06702,(PvA11Bk00410:0.05740,((Pv11Lk01110:0.00014,PvA11Bk00370:0.00015)1.000:0.06932,(Pv11Lk01120:0.00014,PvA11Bk00380:0.00014)1.000:0.06147)0.113:0.01262)0.850:0.00937)0.774:0.00420)0.722:0.00209)0.877:0.00732)0.962:0.01445,(((Pv11Lk03950:0.08104,((Pv11Lk01610:0.00014,PvA11Dk00100:0.00014)1.000:0.09850,(Pv11Lk02460:0.09153,(Pv11Lk01600:0.00014,PvA11Dk00090:0.00014)1.000:0.08225)0.270:0.00235)0.350:0.00141)0.949:0.00936,Pv11Lk04200:0.04690)0.710:0.00211,(Pv11Lk04270:0.07543,Pv11Lk04220:0.07297)0.717:0.00267)0.791:0.00154)0.736:0.00098,Pv11Lk01640:0.08028)0.993:0.00014)0.932:0.00313)0.249:0.00016,((Pv04Sk00060:0.07079,Pv04Sk03270:0.06865)0.338:0.00746,((((((((Pv04Sk03430:0.04084,Pv04Sk03570:0.07064)0.157:0.00599,((Pv07Sk00030:0.02350,(((Pv07Sk00040:0.02306,Pv07Sk00060:0.02510)0.695:0.00015,Pv07Sk00050:0.02408)0.721:0.00016,(Pv07Sk00070:0.04074,Pv07Sk00080:0.01578)0.012:0.00413)0.938:0.00628)0.958:0.00869,((Pv10Sk05160:0.02715,Pv10Sk05170:0.03601)0.941:0.01157,(Pv10Sk05050:0.02807,(Pv10Sk05090:0.03622,(Pv10Sk05070:0.00836,Pv10Sk05080:0.00573)0.670:0.01176)0.988:0.01834)0.709:0.00696)0.917:0.00564)0.308:0.00139)0.000:0.00016,((Pv04Sk00070:0.05835,(Pv01Lk02080:0.04446,(Pv05Lk01940:0.02100,Pv05Lk01950:0.02766)1.000:0.03866)0.665:0.00263)0.864:0.00342,((((((Pv04Sk01820:0.01334,(Pv04Sk01810:0.03368,Pv04Sk01840:0.01686)0.644:0.00433)0.978:0.01021,((Pv04Sk01800:0.03745,Pv04Sk01830:0.02813)0.716:0.00636,(Pv04Sk01780:0.02502,Pv04Sk01790:0.02771)0.740:0.00079)0.976:0.00015)0.885:0.00343,(Pv04Sk00450:0.06844,(((((Pv04Sk02980:0.02751,((Pv04Sk02930:0.03579,(Pv04Sk02960:0.01721,Pv04Sk02970:0.02802)0.953:0.00964)0.879:0.00489,(Pv04Sk02940:0.01905,Pv04Sk02950:0.03428)0.864:0.00490)0.756:0.00159)0.777:0.00620,Pv04Sk02990:0.03257)0.861:0.00635,((((Pv10Lk01850:0.03258,(Pv10Lk01710:0.01570,Pv10Lk01720:0.01122)0.952:0.00874)0.181:0.00298,(((Pv10Lk01660:0.01271,(Pv10Lk01770:0.02240,((Pv10Lk01750:0.01583,(Pv10Lk01760:0.01905,Pv10Lk01790:0.01588)0.905:0.00014)0.635:0.00312,((Pv10Lk01730:0.01430,Pv10Lk01740:0.00471)0.868:0.00315,Pv10Lk01780:0.01911)0.775:0.00154)0.120:0.00015)0.733:0.00155)0.877:0.00313,((Pv10Lk01690:0.02978,Pv10Lk01810:0.03077)0.746:0.00255,Pv10Lk01820:0.02998)0.684:0.00065)0.764:0.00156,(Pv10Lk01800:0.03411,(Pv10Lk01830:0.02972,Pv10Lk01860:0.04013)0.881:0.00576)0.768:0.00150)0.875:0.00014)0.904:0.00311,(((Pv04Sk03560:0.03466,(Pv10Lk01840:0.03272,Pv10Lk01870:0.01502)0.987:0.01671)0.760:0.00219,Pv10Lk01670:0.03518)0.739:0.00045,(Pv04Sk00020:0.03351,(Pv04Sk00010:0.04401,Pv04Sk00030:0.02108)0.934:0.01136)0.811:0.00699)0.996:0.00015)0.813:0.00160,((Pv08Lk00970:0.01584,(Pv05Sk00230:0.01741,(Pv08Lk00980:0.03228,((Pv08Lk00990:0.01102,(Pv08Lk01100:0.00941,(Pv08Lk01070:0.00626,((Pv08Lk01010:0.00156,(((Pv08Lk01110:0.00165,(Pv08Lk01080:0.00015,Pv08Lk01120:0.00799)0.430:0.00779)0.796:0.00170,Pv08Lk01090:0.00312)0.305:0.00015,(Pv08Lk01130:0.00305,(Pv08Lk01140:0.00149,Pv08Lk01150:0.00162)0.646:0.00312)0.917:0.00013)0.541:0.00014)0.785:0.00157,Pv08Lk01030:0.02121)0.785:0.00157)0.125:0.00015)0.794:0.00157)0.760:0.00157,(((Pv08Lk01050:0.00910,Pv08Lk01000:0.00979)0.832:0.00016,Pv08Lk01060:0.01580)0.832:0.00304,Pv08Lk01040:0.00750)0.744:0.00190)0.846:0.00157)0.266:0.00015)0.006:0.00153)0.997:0.01270,((Pv05Lk01920:0.03287,Pv05Lk01930:0.03557)0.929:0.00953,(Pv10Lk00230:0.04433,((Pv10Lk00160:0.01748,Pv10Lk00170:0.02246)0.860:0.00311,((((Pv10Lk00180:0.01767,Pv10Lk00200:0.02094)0.830:0.00302,Pv10Lk00190:0.02908)0.745:0.00269,(Pv10Lk00140:0.04223,Pv10Lk00150:0.01597)0.686:0.00905)0.634:0.00060,(Pv10Lk00210:0.04724,Pv10Lk00220:0.02803)0.904:0.00973)0.215:0.00015)0.763:0.00156)0.949:0.00637)0.882:0.00324)0.536:0.00015)1.000:0.00015)0.834:0.00166,(Pv04Sk00230:0.03903,(((Pv04Sk00330:0.01396,Pv04Sk00350:0.02730)0.916:0.01010,(Pv04Sk00420:0.02077,Pv04Sk00410:0.03256)0.954:0.00014)0.921:0.00624,(((Pv04Sk00270:0.03203,Pv04Sk00380:0.02509)0.912:0.00705,(Pv04Sk00360:0.03150,Pv04Sk00370:0.04127)0.651:0.00332)0.881:0.00442,((((Pv04Sk00340:0.03387,(Pv04Sk00390:0.00151,Pv04Sk00400:0.00474)0.990:0.01518)0.177:0.00453,Pv04Sk00310:0.02700)0.939:0.00697,(Pv04Sk00320:0.02588,Pv04Sk00260:0.02265)0.455:0.00148)0.730:0.00140,(Pv04Sk00290:0.01425,(Pv04Sk00280:0.02546,(Pv04Sk00250:0.01141,Pv04Sk00240:0.03056)0.416:0.00134)0.987:0.01140)0.920:0.00473)0.807:0.00293)0.871:0.00014)0.727:0.00166)0.977:0.00951)0.791:0.00154,(Pv04Sk03460:0.02652,(Pv04Sk03550:0.02783,Pv10Lk01700:0.04485)0.873:0.00638)0.756:0.00153)0.754:0.00015)0.436:0.00015)0.000:0.00010,Pv04Sk03540:0.05528)0.354:0.00011,(Pv04Sk03450:0.05972,(Pv04Sk03410:0.02914,Pv04Sk03420:0.02832)0.981:0.01793)0.788:0.00290)0.464:0.00015,((((Pv04Sk03210:0.00782,Pv04Sk03230:0.01611)1.000:0.04020,Pv04Sk03440:0.05755)0.750:0.00127,((Pv08Lk00800:0.02294,Pv08Lk00810:0.04923)0.768:0.00477,(Pv08Lk00820:0.01966,(Pv08Lk00790:0.03200,(Pv08Lk00770:0.02269,Pv08Lk00780:0.03634)0.890:0.00721)0.449:0.00200)0.452:0.00222)0.992:0.01679)0.984:0.01047,((((Pv04Sk03350:0.05725,Pv04Sk00440:0.03720)0.778:0.00316,(Pv04Sk00080:0.04166,Pv04Sk00090:0.02095)0.893:0.00795)0.722:0.00016,Pv04Sk00430:0.04317)0.992:0.00106,Pv04Sk03380:0.05809)0.961:0.00057)0.740:0.00131)0.000:0.00016)0.401:0.00014)0.911:0.00016,((Pv04Sk03310:0.03648,(Pv04Sk03220:0.03103,Pv04Sk03240:0.04860)0.944:0.00955)0.819:0.00307,(Pv04Sk03250:0.02388,(Pv04Sk03290:0.03762,Pv04Sk03300:0.02734)0.841:0.00492)0.772:0.00165)0.737:0.00155)0.741:0.00015,((((((Pv04Sk02890:0.03229,((Pv04Sk02920:0.02533,(Pv04Sk02900:0.01934,Pv04Sk02910:0.02292)0.320:0.00600)0.759:0.00197,(Pv10Sk05060:0.02682,(Pv04Sk02870:0.02420,(Pv04Sk02840:0.01977,Pv04Sk02880:0.02413)0.824:0.00500)0.528:0.00410)0.783:0.00208)0.075:0.00155)0.764:0.00158,(((Pv10Lk01640:0.03546,Pv10Lk01630:0.02320)0.459:0.00505,(Pv04Sk00570:0.01783,Pv10Lk01620:0.02847)0.453:0.00409)0.885:0.00519,(((Pv04Sk00560:0.02912,(Pv04Sk00470:0.02407,Pv04Sk00480:0.03167)0.789:0.00319)0.850:0.00331,(Pv04Sk00510:0.03234,(Pv04Sk00530:0.03398,(Pv04Sk00540:0.04461,Pv04Sk00550:0.02587)0.872:0.00479)0.422:0.00160)0.744:0.00160)0.760:0.00161,(Pv04Sk00520:0.02284,(Pv04Sk00500:0.05139,Pv04Sk00490:0.03120)0.607:0.00606)0.735:0.00116)0.781:0.00167)0.775:0.00166)0.229:0.00016,(Pv04Sk00300:0.02940,Pv04Sk00580:0.02768)0.824:0.00281)0.896:0.00167,(Pv05Sk00310:0.03355,(Pv05Sk00330:0.03890,Pv05Sk00320:0.03004)0.847:0.00761)0.935:0.00690)0.389:0.00014,Pv04Sk00460:0.02985)0.824:0.00323,((Pv10Lk01560:0.02266,((Pv10Lk01520:0.04472,(Pv10Lk01530:0.01625,Pv10Lk01540:0.02659)0.996:0.02296)0.726:0.00149,(Pv10Lk01550:0.02765,(Pv10Lk01570:0.04688,Pv10Lk01580:0.02242)0.797:0.00369)0.746:0.00157)0.811:0.00159)0.979:0.00070,(Pv04Sk03360:0.06163,Pv04Sk03520:0.05205)0.772:0.00408)0.981:0.00085)0.779:0.00160)0.789:0.00225,(Pv04Sk03530:0.04442,Pv04Sk03510:0.05048)0.454:0.00460)0.924:0.00676,(Pv10Lk01680:0.08005,(Pv05Sk00350:0.05637,Pv08Lk00960:0.03978)0.828:0.00588)0.354:0.00164)0.978:0.01054,(Pv04Sk00040:0.06773,(Pv04Lk05000:0.05823,((((Pv04Sk02070:0.05444,Pv04Sk02700:0.08686)0.828:0.00621,((Pv04Sk02150:0.07391,(Pv04Sk02110:0.06435,Pv04Sk02140:0.03987)0.180:0.00566)0.776:0.00501,(Pv04Sk02820:0.05382,Pv04Sk02830:0.04181)0.678:0.01068)0.703:0.00068)0.796:0.00015,(Pv04Sk02710:0.06439,(Pv04Sk02080:0.03642,Pv04Sk02090:0.04713)0.976:0.01541)0.706:0.00100)0.818:0.00015,Pv04Lk04980:0.04586)0.875:0.00492)0.892:0.00480)0.834:0.00014)0.939:0.00522)0.003:0.00015)0.908:0.00733,((Pv05Lk02170:0.07914,Pv11Lk04560:0.05368)0.828:0.01258,(Pv01Sk01180:0.05794,Pv04Lk05940:0.04892)0.748:0.00400)0.440:0.00337)0.878:0.00561,(Pv08Sk00030:0.08002,(((Pv01Sk00280:0.02083,(Pv01Sk00290:0.04684,Pv01Sk00300:0.01890)0.888:0.01056)0.944:0.01087,(Pv01Sk00270:0.03343,Pv01Sk00310:0.03507)0.898:0.00753)0.979:0.00984,(((((Pv03Sk00450:0.03777,((Pv03Sk00420:0.00668,Pv03Sk00480:0.02087)0.749:0.00132,(Pv03Sk00430:0.03266,Pv03Sk00440:0.02102)0.581:0.00015)0.970:0.01131)0.863:0.00957,((Pv03Sk00460:0.04818,Pv03Sk00470:0.05334)0.908:0.01301,(Pv03Sk00500:0.03272,Pv03Sk00510:0.04028)0.288:0.00574)0.910:0.00811)0.742:0.00137,((Pv04Lk04670:0.05080,((Pv01Sk01490:0.06503,Pv04Lk07000:0.03657)0.657:0.00619,(((((Pv01Sk02000:0.05612,Pv10Sk05000:0.03151)0.897:0.00784,Pv05Lk02020:0.06717)0.725:0.00118,(((Pv01Sk01460:0.03693,Pv01Sk01970:0.04551)0.773:0.00542,(Pv01Sk01510:0.03885,(Pv01Sk01500:0.03672,Pv01Sk01520:0.03977)0.227:0.00598)0.743:0.00223)0.050:0.00014,((Pv02Sk00340:0.03582,Pv02Sk00360:0.04330)0.956:0.01501,(Pv01Sk02010:0.05874,(Pv01Sk01390:0.03218,Pv04Lk06750:0.02793)0.385:0.00275)0.723:0.00331)1.000:0.00014)0.454:0.00015)1.000:0.00014,(((((Pv10Lk01250:0.03634,Pv10Lk01260:0.05464)0.820:0.00449,(Pv10Lk01270:0.02898,(Pv10Lk01280:0.03951,Pv10Lk01290:0.01699)0.976:0.01336)0.848:0.00015)0.872:0.00159,(Pv08Lk00950:0.03732,Pv02Sk00060:0.02566)0.812:0.00016)0.921:0.00313,(((((((Pv01Sk01430:0.03106,Pv01Sk01480:0.06483)0.862:0.00784,Pv01Lk02260:0.04203)0.742:0.00164,((((Pv08Lk01180:0.03238,(Pv10Sk05310:0.01583,(((((Pv10Sk05110:0.02453,((Pv01Sk00030:0.01911,Pv01Sk00040:0.01589)0.907:0.00471,((Pv01Sk00010:0.02437,Pv01Sk00050:0.02376)0.742:0.00183,((Pv03Lk00950:0.01823,Pv10Sk05130:0.01820)0.989:0.01475,Pv01Sk00020:0.06309)0.748:0.00167)0.762:0.00151)0.732:0.00109)0.674:0.00156,(Pv10Sk05150:0.02630,(Pv10Sk05120:0.02395,(Pv10Sk05100:0.02904,Pv10Sk05140:0.03410)0.860:0.00016)0.668:0.00315)0.851:0.00339)0.642:0.00156,Pv08Sk00070:0.01794)0.735:0.00109,((Pv08Sk00010:0.02098,Pv08Sk00020:0.01435)0.886:0.00461,(Pv03Sk00010:0.01901,Pv11Sk00010:0.01267)0.924:0.00465)0.736:0.00155)0.880:0.00313,(Pv10Sk05410:0.03600,((Pv10Sk05390:0.03707,(Pv10Sk05340:0.01732,Pv10Sk05370:0.02098)0.395:0.00150)0.918:0.00491,((Pv10Sk05400:0.02078,Pv10Sk05320:0.03074)0.730:0.00159,((Pv10Sk05330:0.02096,Pv10Sk05380:0.00901)0.511:0.00555,(Pv10Sk05300:0.01902,(Pv10Sk05350:0.01237,Pv10Sk05360:0.01567)0.521:0.00362)0.999:0.00014)0.857:0.00153)0.796:0.00016)0.652:0.00159)0.893:0.00322)0.765:0.00160)0.878:0.00319)0.919:0.00487,(((((Pv04Lk04650:0.02886,(Pv05Lk02050:0.05582,Pv05Lk02010:0.04265)0.745:0.00314)0.931:0.00016,Pv10Sk05020:0.01915)0.804:0.00156,((Pv01Sk01950:0.03934,Pv02Sk00350:0.03440)0.779:0.00309,(Pv01Sk01960:0.06826,Pv08Lk01170:0.02766)0.387:0.00119)0.877:0.00015)0.765:0.00016,(Pv01Lk02200:0.02594,Pv04Lk06760:0.03279)0.802:0.00277)0.835:0.00156,((((Pv05Lk02080:0.03384,Pv03Sk00320:0.05869)0.655:0.00112,((Pv01Sk02020:0.04468,Pv01Sk02060:0.03661)0.971:0.01593,(Pv01Sk02040:0.04706,Pv01Sk02070:0.03908)0.847:0.00631)0.443:0.00254)0.694:0.00084,(((Pv03Sk00340:0.03757,((Pv03Sk00380:0.02890,(Pv03Sk00390:0.02753,(Pv03Sk00330:0.01793,(Pv03Sk00360:0.01464,Pv03Sk00410:0.00595)0.998:0.01954)0.750:0.00145)0.985:0.01172)0.888:0.00015,(Pv03Sk00370:0.02105,(Pv03Sk00350:0.00468,Pv03Sk00400:0.00632)1.000:0.02773)0.885:0.00438)0.083:0.00152)0.961:0.00662,(Pv03Sk00490:0.04585,((((Pv03Sk00020:0.02733,(Pv03Sk00040:0.01506,Pv03Sk00110:0.02938)0.834:0.00811)0.935:0.00015,Pv03Sk00120:0.02231)0.054:0.00015,((Pv03Sk00070:0.02538,Pv03Sk00090:0.01551)0.857:0.01197,(Pv03Sk00100:0.02564,(Pv03Sk00060:0.02561,Pv03Sk00080:0.02067)0.968:0.01024)1.000:0.00016)0.944:0.00298)0.919:0.00014,(Pv03Sk00030:0.01159,Pv03Sk00050:0.02077)0.402:0.00309)0.994:0.01306)0.833:0.00014)0.500:0.00015,(Pv01Sk01990:0.06575,(Pv01Sk02030:0.04401,(Pv01Sk01980:0.04852,Pv01Sk02050:0.05506)0.300:0.00346)0.391:0.00179)0.842:0.00015)0.002:0.00016)0.869:0.00158,((Pv04Lk06780:0.03524,(Pv10Sk04980:0.03690,Pv10Sk05030:0.02045)0.640:0.00483)0.305:0.00360,(((Pv04Lk06730:0.00944,Pv04Lk06770:0.02112)1.000:0.02583,(((((Pv04Lk06880:0.02270,Pv04Lk06940:0.02419)0.764:0.00129,((Pv04Lk06950:0.02587,(Pv04Lk06800:0.01760,Pv04Lk06830:0.02418)0.988:0.01452)0.727:0.00135,(((((Pv04Lk06870:0.02138,(Pv04Lk06790:0.02456,Pv04Lk06900:0.01229)0.173:0.00210)0.845:0.00604,(Pv04Lk06850:0.01816,Pv04Lk06860:0.01296)0.906:0.00687)0.930:0.00544,(Pv04Lk06810:0.01627,Pv04Lk06820:0.06594)0.801:0.00281)0.811:0.00163,Pv04Lk06920:0.02994)1.000:0.00014,(Pv04Lk06840:0.02423,Pv04Lk06890:0.04406)0.732:0.00468)0.914:0.00158)1.000:0.00014)0.434:0.00337,Pv04Lk06930:0.02590)0.953:0.00689,(Pv01Sk00080:0.04326,(Pv01Sk00070:0.02025,(Pv01Sk00110:0.01960,(Pv01Sk00100:0.02738,(Pv01Sk00060:0.01825,Pv01Sk00090:0.01674)0.726:0.00305)0.979:0.01286)0.854:0.00325)0.906:0.00756)0.780:0.00244)0.766:0.00170,Pv07Sk00020:0.03244)0.781:0.00165)0.377:0.00015,((Pv05Lk02230:0.01206,Pv05Lk02240:0.02563)0.992:0.01364,Pv04Lk06740:0.04410)0.784:0.00015)0.890:0.00164)0.999:0.00016)0.320:0.00016)0.251:0.00014)0.122:0.00015,((((Pv05Lk02030:0.05113,(Pv04Lk04690:0.02711,Pv04Lk04700:0.05154)0.950:0.01046)0.858:0.00472,Pv01Sk00180:0.04904)0.887:0.00014,(Pv04Lk04680:0.05317,Pv05Lk02040:0.04536)0.758:0.00369)0.763:0.00015,((Pv01Lk02270:0.03665,Pv01Lk02290:0.04181)0.944:0.01049,((Pv06Lk00240:0.02105,Pv06Lk00250:0.03282)0.955:0.00927,((Pv02Lk00870:0.02387,Pv02Lk00880:0.02138)0.971:0.01355,(Pv02Lk00900:0.02319,(Pv02Lk00890:0.02595,Pv02Lk00910:0.02585)0.828:0.00474)0.743:0.00439)0.789:0.00224)0.781:0.00154)0.827:0.00016)0.926:0.00155)0.443:0.00015,((Pv05Lk01970:0.02164,(Pv05Lk02000:0.02988,(Pv05Lk01980:0.01111,Pv05Lk01990:0.01776)0.970:0.01041)0.394:0.00150)0.955:0.00710,(Pv02Sk00020:0.02123,(Pv02Sk00010:0.02064,(Pv02Sk00030:0.02093,(Pv02Sk00040:0.00879,Pv02Sk00050:0.03068)0.954:0.00881)0.733:0.00200)0.419:0.00153)0.939:0.00590)0.767:0.00155)0.018:0.00015)0.445:0.00015,(Pv04Lk04660:0.02648,Pv01Sk01440:0.03821)0.830:0.00411)0.000:0.00015,(Pv01Sk01410:0.02859,(Pv01Sk00170:0.03830,Pv01Sk01420:0.03829)0.965:0.01508)0.956:0.00864)0.414:0.00014,((Pv10Sk04990:0.03937,Pv10Sk05010:0.04178)0.908:0.00635,((((Pv10Sk05230:0.05124,(Pv10Sk05250:0.02721,Pv10Sk05270:0.03567)0.836:0.00352)0.883:0.00321,((Pv10Sk05220:0.02472,Pv10Sk05260:0.03290)0.593:0.00593,(Pv10Sk05190:0.03064,Pv10Sk05280:0.03258)0.981:0.01679)0.720:0.00155)0.866:0.00309,((Pv02Sk00420:0.04346,Pv10Sk05240:0.04982)0.501:0.00516,Pv06Lk00230:0.03869)0.732:0.00238)0.779:0.00158,(Pv03Sk00300:0.03045,(((Pv03Sk00260:0.04140,Pv03Sk00290:0.04054)0.751:0.00239,(Pv03Sk00250:0.01729,Pv03Sk00280:0.01806)0.834:0.00306)0.767:0.00015,Pv03Sk00270:0.02189)0.930:0.00664)0.954:0.00778)1.000:0.00068)1.000:0.00086)0.125:0.00011,((Pv01Sk01530:0.06732,(Pv01Sk01380:0.03534,Pv01Sk01450:0.02918)0.703:0.00472)0.876:0.00523,(((Pv11Sk00030:0.02517,(Pv11Sk00020:0.02240,Pv11Sk00040:0.00894)0.992:0.01627)0.867:0.00498,((Pv05Lk02060:0.03419,Pv05Lk02070:0.04496)0.989:0.01516,(Pv01Sk01400:0.02376,Pv10Sk05290:0.03565)0.728:0.00120)0.600:0.00154)0.667:0.00173,Pv01Sk01470:0.04217)0.781:0.00015)0.825:0.00154)0.311:0.00016)0.801:0.00014,Pv02Sk00370:0.03218)0.882:0.00160)0.593:0.00163,(Pv02Sk00410:0.04513,Pv04Sk03280:0.03680)0.737:0.00079)0.809:0.00016)0.844:0.00362)0.418:0.00169,((Pv07Sk00130:0.03891,Pv07Sk00140:0.03465)0.993:0.02678,(Pv10Sk05420:0.07168,Pv08Sk00040:0.04278)0.036:0.00431)0.842:0.00525)0.954:0.00786)0.915:0.00014,(((Pv03Sk00540:0.02731,(Pv03Sk00560:0.01927,Pv03Sk00590:0.01121)0.961:0.00944)0.755:0.00188,Pv03Sk00580:0.03656)0.166:0.00146,Pv03Sk00550:0.04586)0.966:0.00984)0.749:0.00575,((((Pv04Sk02770:0.05359,(Pv04Sk02750:0.04912,Pv04Sk02800:0.07475)0.984:0.02369)0.427:0.00276,Pv04Sk02760:0.05462)0.312:0.00202,Pv04Sk02740:0.05386)0.798:0.00552,(((((Pv04Sk02260:0.05280,Pv04Sk02310:0.05744)0.599:0.00195,Pv04Sk02360:0.05421)0.745:0.00158,(((Pv04Sk02320:0.05950,Pv04Sk02350:0.03190)0.821:0.00396,(Pv04Sk02340:0.03632,(Pv04Sk02250:0.03710,Pv04Sk02270:0.04935)0.427:0.00598)0.774:0.00292)0.761:0.00157,(Pv04Sk02290:0.02372,Pv04Sk02300:0.03755)0.992:0.02005)0.776:0.00170)0.967:0.00622,(Pv04Lk04990:0.06059,Pv04Lk05040:0.06253)0.933:0.01380)0.900:0.00015,(((Pv04Sk02190:0.03778,(Pv04Sk02170:0.03140,Pv04Sk02240:0.03183)0.056:0.00239)0.769:0.00175,(((Pv04Sk02200:0.03324,Pv04Sk02220:0.03087)0.947:0.00937,Pv04Sk02180:0.03245)0.733:0.00157,(Pv04Sk02230:0.04072,Pv04Sk02210:0.02928)0.876:0.00014)0.735:0.00285)0.919:0.00466,((((Pv04Sk01910:0.03319,Pv04Sk01950:0.04927)0.837:0.00492,((Pv04Sk02430:0.04198,(Pv04Sk02420:0.02042,Pv04Sk02470:0.04557)0.950:0.01413)0.958:0.01529,(Pv04Sk01990:0.04466,Pv04Sk02000:0.03729)0.878:0.01045)0.896:0.00577)0.994:0.00016,((Pv04Sk02380:0.03263,Pv04Sk02440:0.03851)0.971:0.01320,((Pv04Sk02390:0.03891,Pv04Sk02450:0.04811)0.981:0.01610,(Pv04Sk02410:0.05565,(Pv04Sk02400:0.04603,Pv04Sk02460:0.03185)0.005:0.00344)0.843:0.00821)0.764:0.00370)0.794:0.00158)0.849:0.00155,(((Pv04Sk01930:0.02800,Pv04Sk01960:0.01965)0.965:0.01145,Pv04Sk01920:0.04938)0.334:0.00283,(Pv04Sk02480:0.01561,Pv04Sk02530:0.03729)0.998:0.02284)0.864:0.00015)0.770:0.00154)0.793:0.00153)0.764:0.00135)0.913:0.00490)0.731:0.00153)0.451:0.00394)0.754:0.00213)0.779:0.00015)0.033:0.00016,((Pv10Lk01180:0.08866,Pv10Lk02280:0.07873)0.411:0.00133,(Pv01Sk00120:0.07382,(Pv08Sk00050:0.06298,Pv10Sk05530:0.06190)0.145:0.00376)0.909:0.00682)0.780:0.00209)0.305:0.00014,((Pv08Sk00060:0.06343,Pv10Lk02300:0.07506)0.743:0.00370,(((Pv04Sk02490:0.01800,Pv04Sk02540:0.02296)1.000:0.04650,((Pv11Lk05980:0.07036,(Pv11Lk05590:0.07354,(Pv11Lk05960:0.06135,Pv11Lk05970:0.05561)0.922:0.01433)0.679:0.00016)0.834:0.00387,(Pv04Lk05120:0.05135,Pv10Sk05520:0.11356)0.896:0.01561)0.745:0.00501)0.776:0.00156,(Pv10Lk02310:0.04830,Pv10Lk02320:0.04355)0.952:0.01309)0.749:0.00160)0.786:0.00157)0.412:0.00016,((((Pv11Lk00720:0.06554,(Pv11Lk00670:0.05753,Pv11Lk00760:0.03234)0.877:0.01647)0.983:0.02116,(Pv11Lk00650:0.09491,(Pv11Lk00660:0.05990,(Pv11Lk00690:0.04230,Pv11Lk00730:0.03715)0.991:0.02584)0.012:0.00846)0.879:0.00709)0.790:0.00436,(Pv10Lk02020:0.07358,Pv11Lk03830:0.06367)0.501:0.00392)0.697:0.00184,((Pv11Lk00710:0.05761,(Pv11Lk00630:0.07346,(Pv11Lk00540:0.08109,Pv11Lk00640:0.05614)0.308:0.01067)0.673:0.00673)0.423:0.00849,Pv11Lk00560:0.08688)0.476:0.00695)0.811:0.00278)0.878:0.00252,(((((((((Pv03Sk00310:0.06646,((Pv10Lk02420:0.09045,Pv10Lk02350:0.07194)0.700:0.00119,(Pv10Lk02050:0.05963,((Pv10Lk02110:0.07977,(Pv10Lk02090:0.05564,(Pv10Lk02160:0.04928,Pv10Lk02180:0.03341)0.952:0.01668)0.582:0.00517)0.821:0.00416,(((Pv10Lk02130:0.04429,Pv10Lk02140:0.03723)0.388:0.00577,((Pv10Lk02100:0.03120,Pv10Lk02120:0.05408)0.877:0.00825,(Pv10Lk02170:0.05523,Pv10Lk02190:0.03894)0.798:0.01412)0.580:0.00061)0.775:0.00425,Pv10Lk02260:0.07121)0.165:0.00411)0.857:0.00548)0.919:0.00768)0.854:0.00346)0.765:0.00167,(((((Pv04Lk06050:0.00667,Pv04Lk06100:0.00895)1.000:0.10044,(Pv01Sk00460:0.05392,Pv11Lk03060:0.07345)0.658:0.00635)0.709:0.00107,((Pv10Lk01880:0.06882,Pv10Lk01890:0.05415)0.978:0.02124,(((Pv11Lk01620:0.00014,PvA11Dk00110:0.00014)1.000:0.06618,(Pv04Lk04420:0.03787,Pv04Lk04440:0.07796)0.988:0.02864)0.841:0.00566,((Pv11Lk05240:0.04681,Pv11Lk05270:0.06723)0.585:0.00582,((Pv11Lk01210:0.00478,PvA11Ck00020:0.00661)1.000:0.07562,Pv10Lk00910:0.09655)0.746:0.00480)0.781:0.00340)0.854:0.00224)0.998:0.00015)0.769:0.00169,(((Pv04Sk00790:0.01043,Pv04Sk00820:0.01716)0.975:0.01094,(((Pv04Sk00810:0.02398,Pv04Sk00850:0.01147)0.754:0.00166,Pv04Sk00800:0.01904)0.248:0.00158,(Pv04Sk00860:0.01577,(Pv04Sk00830:0.01434,Pv04Sk00840:0.01921)0.593:0.00448)0.923:0.00016)0.737:0.00122)1.000:0.03350,Pv01Sk00320:0.08877)0.782:0.00015)0.859:0.00344,((((Pv05Sk01140:0.00312,PvA05Ak00040:0.00014)1.000:0.06591,(Pv05Sk01120:0.00312,PvA05Ak00020:0.00014)1.000:0.04312)0.445:0.00482,((Pv05Sk01130:0.00470,PvA05Ak00030:0.00162)1.000:0.04622,(PvA05Ak00080:0.04565,((Pv08Ck00100:0.00014,PvA05Ak00090:0.00014)1.000:0.04519,((Pv08Ck00090:0.00014,PvA05Ak00100:0.00014)1.000:0.03767,(PvA05Ak00120:0.01109,(Pv08Ck00080:0.00014,PvA05Ak00110:0.00014)0.991:0.01280)0.993:0.02205)0.980:0.01802)0.981:0.01561)0.946:0.01021)0.706:0.00144)0.727:0.00172,((Pv05Sk01150:0.00313,PvA05Ak00050:0.00015)1.000:0.05169,((Pv05Sk01170:0.00312,PvA05Ak00070:0.00015)1.000:0.06740,(Pv05Sk01160:0.00025,PvA05Ak00060:0.00131)1.000:0.05911)0.207:0.00372)0.809:0.00339)0.998:0.01893)0.952:0.00673)0.803:0.00168,(Pv11Lk03370:0.07066,((((Pv11Lk00680:0.03691,Pv11Lk00750:0.04518)0.898:0.01241,Pv11Lk04780:0.05567)0.275:0.00267,(Pv11Lk00700:0.04201,Pv11Lk00740:0.03677)0.999:0.04163)0.715:0.00235,(Pv11Lk00570:0.02800,Pv11Lk00590:0.05538)1.000:0.04105)0.247:0.00015)0.801:0.00170)0.916:0.00016,(((((((((Pv10Sk04880:0.04025,Pv10Sk04890:0.03489)0.823:0.00384,(Pv10Sk04930:0.03749,(Pv10Sk04960:0.04662,(Pv10Sk04920:0.03904,(Pv10Sk04900:0.03452,Pv10Sk04940:0.02623)0.856:0.00468)0.529:0.00403)0.876:0.00584)0.853:0.00015)0.885:0.00313,((Pv10Sk04910:0.04694,Pv10Sk04950:0.02767)0.974:0.01662,Pv01Sk00450:0.07716)0.702:0.00159)0.797:0.00163,((Pv01Sk01350:0.04035,(Pv01Sk01360:0.02293,Pv01Sk01370:0.03134)0.204:0.00277)0.781:0.00178,((((((Pv01Sk00340:0.01930,Pv01Sk00380:0.02117)0.889:0.00451,(Pv01Sk00370:0.01504,Pv01Sk00350:0.03072)0.707:0.00083)0.762:0.00162,Pv01Sk00360:0.01584)0.761:0.00161,(Pv01Sk00430:0.01908,(Pv01Sk00400:0.01730,Pv01Sk00440:0.01568)0.870:0.00355)0.757:0.00155)0.000:0.00016,(Pv01Sk00420:0.00945,Pv11Lk04530:0.02097)0.951:0.00783)0.993:0.00015,(Pv01Sk00330:0.03302,Pv01Sk00390:0.02509)0.906:0.01667)0.955:0.01099)0.753:0.00180)0.979:0.00016,((((Pv11Lk03100:0.05798,Pv11Lk03210:0.04721)0.208:0.00231,Pv10Sk04970:0.04406)0.752:0.00165,((Pv11Lk03350:0.03481,(Pv11Lk03330:0.02789,Pv11Lk03200:0.05692)0.017:0.00578)0.878:0.00569,((Pv11Lk03180:0.03564,(Pv11Lk03170:0.04749,(Pv11Lk03120:0.05034,Pv11Lk03150:0.04200)0.932:0.01122)0.785:0.00335)0.792:0.00157,((Pv11Lk03130:0.04445,Pv11Lk03160:0.05357)0.685:0.00436,((Pv11Lk03190:0.03473,Pv11Lk03250:0.03167)0.910:0.00911,(Pv11Lk03110:0.01995,Pv11Lk03140:0.03983)0.818:0.00465)0.775:0.00245)0.888:0.00016)0.763:0.00016)0.855:0.00154)0.845:0.00014,Pv11Lk03360:0.05079)0.935:0.00611)0.969:0.00488,(Pv11Lk02630:0.00014,PvA11Ek00160:0.00015)1.000:0.08341)0.778:0.00015,(((Pv01Sk00410:0.05446,(Pv04Lk05160:0.06160,(Pv11Lk03580:0.06144,Pv11Lk03630:0.06302)0.791:0.00599)0.833:0.00338)0.851:0.00014,(((Pv05Lk02160:0.05488,((Pv05Lk02130:0.03541,(Pv05Lk02100:0.05396,(Pv05Lk02110:0.06203,Pv05Lk02150:0.03041)0.953:0.01228)0.728:0.00093)0.760:0.00235,(Pv05Lk02120:0.04180,Pv05Lk02140:0.04155)0.954:0.01465)0.879:0.00451)0.706:0.00151,Pv05Lk02090:0.04267)0.872:0.00329,(Pv05Sk00070:0.04017,Pv05Sk00050:0.04909)0.801:0.00377)0.937:0.00483)0.852:0.00157,((Pv05Sk00060:0.04071,Pv05Sk00080:0.06692)0.764:0.00510,Pv04Lk05240:0.04524)0.854:0.00614)0.981:0.00016)0.048:0.00014,Pv01Sk00690:0.08040)0.843:0.00159,((Pv11Lk04870:0.08405,(Pv11Lk04890:0.07159,(Pv11Lk04950:0.06659,Pv11Lk04960:0.05457)0.035:0.00743)0.747:0.00467)0.770:0.00220,((Pv04Lk05410:0.05308,Pv11Lk05630:0.07026)0.843:0.01053,(Pv01Sk00680:0.05609,Pv11Lk03610:0.04897)0.158:0.00304)0.763:0.00326)0.662:0.00015)0.913:0.00318)0.950:0.00317,((Pv11Lk04570:0.07767,(Pv11Lk04380:0.07433,Pv11Lk04400:0.06958)0.896:0.01423)0.056:0.00335,((Pv10Lk03480:0.06235,(Pv10Lk03400:0.05115,Pv10Lk03510:0.03616)0.918:0.02665)0.987:0.02466,((Pv10Lk03440:0.03324,(Pv10Lk03390:0.03949,(Pv10Lk03410:0.02059,Pv10Lk03490:0.01136)0.983:0.01741)0.681:0.00776)1.000:0.03807,Pv11Lk00490:0.06416)0.366:0.00015)0.961:0.00845)0.888:0.00015)0.976:0.01008,Pv10Lk03100:0.06233)0.708:0.00106,(((Pv11Lk01030:0.09052,Pv11Lk01540:0.09140)0.704:0.00405,(Pv10Lk02590:0.06033,(Pv11Lk03380:0.07609,(Pv11Lk03410:0.07093,(Pv11Lk03390:0.04378,Pv11Lk03420:0.06507)0.860:0.00648)0.799:0.00798)0.977:0.01856)0.836:0.00512)0.570:0.00266,(((Pv04Sk01660:0.05648,Pv11Lk03810:0.03811)0.296:0.00618,((Pv11Lk04240:0.04007,(Pv11Lk04250:0.05573,Pv11Lk04260:0.05860)0.715:0.00920)0.877:0.00570,((((Pv10Lk04280:0.06754,(((Pv10Lk04210:0.04422,((Pv10Lk04250:0.03772,(Pv10Lk04100:0.05624,Pv10Lk04170:0.06019)0.906:0.00957)0.725:0.00140,((Pv10Lk04190:0.04713,Pv10Lk04200:0.03266)0.959:0.01348,(Pv10Lk04110:0.05671,(Pv10Lk04120:0.03501,Pv10Lk04140:0.05773)0.748:0.00230)0.935:0.00719)0.831:0.00383)0.525:0.00168)0.805:0.00016,(((Pv10Lk04160:0.05322,Pv10Lk04180:0.03262)0.999:0.03487,(Pv10Lk04130:0.05344,Pv10Lk04150:0.05092)0.534:0.00146)0.894:0.00518,Pv10Lk04240:0.05676)0.129:0.00171)0.103:0.00016,((Pv10Lk04090:0.05756,(Pv10Lk04310:0.04004,(Pv10Lk04230:0.02581,Pv10Lk04270:0.05107)0.981:0.01816)0.206:0.00462)0.145:0.00181,(Pv10Lk04220:0.04293,Pv10Lk04260:0.02801)0.952:0.01181)0.836:0.00014)0.951:0.00958)0.928:0.00926,(((Pv10Lk03230:0.05561,Pv10Lk03360:0.04050)1.000:0.03892,((((Pv10Lk03250:0.05200,(Pv10Lk03160:0.04248,Pv10Lk04300:0.04104)0.906:0.00954)0.712:0.00086,((Pv10Lk03260:0.04189,Pv10Lk03270:0.04534)0.974:0.01740,(((Pv10Lk03130:0.06448,Pv10Lk03330:0.05374)0.685:0.00203,Pv10Lk03340:0.03362)0.402:0.00260,(Pv10Lk03290:0.02288,Pv10Lk03310:0.03901)0.999:0.03202)0.729:0.00150)0.773:0.00155)0.715:0.00139,Pv10Lk03170:0.07310)0.826:0.00928,((Pv10Lk03210:0.05901,(Pv10Lk03180:0.05731,Pv10Lk03190:0.05839)0.934:0.01295)0.836:0.00480,((Pv10Lk03120:0.04548,Pv10Lk03200:0.04854)0.811:0.00510,(Pv10Lk03240:0.06066,(Pv10Lk03150:0.06787,(Pv10Lk03140:0.07238,Pv10Lk03300:0.07436)0.786:0.00676)0.473:0.00016)0.872:0.00813)0.581:0.00014)0.881:0.00316)0.700:0.00014)0.541:0.00014,(Pv10Lk04290:0.05571,(Pv10Lk03320:0.04112,Pv10Lk03350:0.04854)0.947:0.01245)0.715:0.00090)0.797:0.00463)0.876:0.00325,(((Pv05Sk00730:0.04827,Pv10Lk00540:0.04886)0.872:0.00784,((Pv10Lk00530:0.05204,Pv11Lk00510:0.06156)0.934:0.01252,((Pv10Lk00480:0.04804,Pv10Lk00520:0.05095)0.900:0.00904,(Pv10Lk00500:0.05376,Pv10Lk00490:0.05144)0.419:0.00230)0.756:0.00169)0.716:0.00036)0.756:0.00155,((((Pv10Lk03680:0.08110,Pv10Lk00670:0.05132)0.043:0.01002,Pv10Lk00440:0.05110)0.813:0.00530,((((Pv10Lk03720:0.08341,Pv10Lk03760:0.05288)0.863:0.00810,Pv10Lk00450:0.05723)0.772:0.00301,Pv10Lk02610:0.06358)0.810:0.00291,((Pv10Lk00470:0.07475,(Pv10Lk03540:0.04500,(Pv10Lk03430:0.03764,Pv10Lk03460:0.04955)0.915:0.01603)0.974:0.01549)0.835:0.00576,(Pv10Lk03030:0.05500,Pv10Lk03730:0.06163)0.709:0.00143)0.471:0.00016)0.592:0.00154)0.864:0.00015,((((Pv10Lk00460:0.05656,Pv10Lk03070:0.07626)0.716:0.00409,(Pv10Lk03570:0.06912,Pv10Lk03550:0.09233)0.757:0.00575)0.791:0.00260,((Pv10Lk02600:0.06213,Pv05Sk00720:0.06103)0.734:0.00845,(((Pv10Lk03690:0.02570,Pv10Lk03740:0.02743)0.982:0.02486,(Pv10Lk03700:0.03279,Pv10Lk03750:0.05051)0.858:0.01472)0.336:0.00569,(Pv10Lk02580:0.06877,Pv10Lk02570:0.03003)0.435:0.00353)0.869:0.00526)0.833:0.00014)0.720:0.00016,(Pv10Lk03470:0.06572,((((Pv10Lk03520:0.03063,Pv10Lk03530:0.01160)1.000:0.03971,(Pv10Lk03560:0.05555,(Pv10Lk03420:0.04090,(Pv10Lk03450:0.03644,Pv10Lk03500:0.04392)0.275:0.00251)0.889:0.00809)0.343:0.00239)0.964:0.01388,(Pv10Lk03040:0.06936,Pv10Lk00510:0.05816)0.691:0.00244)0.752:0.00246,(Pv10Lk03050:0.05670,Pv10Lk03060:0.03124)0.995:0.02539)0.855:0.00312)0.950:0.00015)0.759:0.00015)0.391:0.00015)0.706:0.00015)0.927:0.00311,(((Pv10Lk00420:0.04646,Pv10Lk00430:0.02511)0.993:0.02368,((Pv11Lk01460:0.07123,(Pv11Lk01470:0.00014,PvA11Dk00010:0.00014)1.000:0.06564)0.030:0.00675,((Pv11Lk01490:0.00015,PvA11Dk00030:0.00014)1.000:0.09065,(Pv11Lk01480:0.00014,PvA11Dk00020:0.00014)1.000:0.06480)0.185:0.00880)0.873:0.00612)0.725:0.00099,((Pv11Lk01900:0.04713,Pv11Lk01910:0.06312)0.998:0.02734,(((((Pv11Lk01700:0.00014,PvA11Dk00180:0.00016)1.000:0.09812,(((Pv11Lk01760:0.00014,PvA11Dk00240:0.00015)1.000:0.05570,((Pv11Lk01740:0.00014,PvA11Dk00220:0.00014)1.000:0.06548,((Pv11Lk01780:0.0,PvA11Dk00260:0.0):0.03509,((Pv11Lk01750:0.0,PvA11Dk00230:0.0):0.06873,(Pv11Lk01770:0.00014,PvA11Dk00250:0.00014)1.000:0.04386)0.479:0.00634)0.788:0.00307)0.793:0.00376)0.731:0.00155,((Pv11Lk01730:0.00014,PvA11Dk00210:0.00014)1.000:0.06211,(Pv11Lk01710:0.00014,PvA11Dk00190:0.00015)1.000:0.06275)0.825:0.00015)0.860:0.00652)0.893:0.00883,((Pv11Lk01660:0.0,PvA11Dk00140:0.0):0.08597,((Pv11Lk01650:0.00014,PvA11Dk00130:0.00318)1.000:0.08316,(Pv11Lk01670:0.00014,PvA11Dk00150:0.00014)1.000:0.07817)0.871:0.01334)0.826:0.00848)0.976:0.01097,((Pv11Lk01500:0.00014,PvA11Dk00040:0.00015)1.000:0.07868,(Pv11Lk01510:0.09384,((Pv11Lk01560:0.00014,PvA11Dk00050:0.00014)1.000:0.04831,((Pv11Lk01580:0.00373,PvA11Dk00070:0.01454)1.000:0.08695,(Pv11Lk01570:0.00014,PvA11Dk00060:0.00016)1.000:0.06641)0.726:0.01226)0.393:0.00376)0.691:0.00581)0.573:0.00145)0.799:0.00169,((((Pv05Sk01850:0.04178,Pv05Sk01660:0.03598)0.431:0.00578,(Pv05Sk01070:0.02999,Pv05Sk01080:0.03864)0.942:0.01049)0.882:0.00014,((Pv05Sk01650:0.03213,(Pv05Sk01870:0.04356,Pv05Sk01900:0.04459)0.602:0.00634)0.636:0.00016,((((Pv05Sk01580:0.04438,(Pv05Sk01590:0.04086,Pv05Sk01600:0.03917)0.271:0.00283)0.839:0.00414,(Pv05Sk01640:0.03090,(Pv05Sk01610:0.05852,Pv05Sk01830:0.04116)0.243:0.00339)0.804:0.00419)0.275:0.00147,((Pv05Sk01840:0.04422,(Pv05Sk01710:0.01849,Pv05Sk01720:0.02272)1.000:0.04385)0.442:0.00014,(Pv05Sk01700:0.06387,(Pv05Sk01690:0.05768,Pv11Lk01790:0.04757)0.766:0.00309)0.821:0.00375)0.787:0.00164)0.745:0.00015,(((Pv05Sk00250:0.02425,Pv05Sk00260:0.01275)0.980:0.01109,(((PvA05Ak00250:0.04013,PvA05Ak00350:0.04036)0.932:0.01103,(PvA05Ak00170:0.02248,(PvA05Ak00230:0.01953,(PvA05Ak00210:0.00156,PvA05Ak00220:0.00469)0.995:0.01948)0.928:0.00970)0.930:0.00637)0.779:0.00160,(((((Pv05Sk01190:0.00014,PvA05Ak00330:0.01000)1.000:0.04692,((PvA05Ak00260:0.03636,(Pv05Sk01210:0.01361,PvA05Ak00290:0.04273)1.000:0.02209)0.844:0.00714,PvA05Ak00300:0.03186)0.803:0.00377)0.910:0.00667,(((Pv05Sk01250:0.00156,PvA05Ak00390:0.00014)1.000:0.03443,(PvA05Ak00270:0.02821,((Pv05Sk01220:0.00519,PvA05Ak00360:0.00150)0.999:0.03029,(Pv05Sk01200:0.01417,PvA05Ak00340:0.01919)0.963:0.01459)0.915:0.00931)0.959:0.01131)0.801:0.00444,PvA05Ak00280:0.02371)0.895:0.00423)0.306:0.00014,(((PvA05Ak00160:0.01869,PvA05Ak00200:0.01549)0.998:0.02577,PvA05Ak00190:0.03894)0.144:0.00338,((Pv05Sk01240:0.00155,PvA05Ak00380:0.00014)1.000:0.04424,(PvA05Ak00180:0.03196,((Pv05Sk01180:0.00014,PvA05Ak00320:0.00014)1.000:0.03721,(PvA05Ak00240:0.02472,PvA05Ak00310:0.03598)0.877:0.00577)0.506:0.00340)0.917:0.00731)0.912:0.00479)0.776:0.00016)0.852:0.00158,(PvA05Ak00400:0.01556,(PvA05Ak00410:0.04789,(PvA05Ak00430:0.03214,(Pv05Sk01260:0.00334,PvA05Ak00420:0.00312)0.812:0.00662)0.967:0.01105)0.813:0.00306)0.871:0.00367)0.000:0.00015)0.954:0.00715)0.424:0.00177,Pv05Sk01570:0.02600)0.666:0.00015)0.313:0.00016)0.810:0.00016)0.870:0.00345,(Pv10Lk02940:0.04930,(((Pv10Lk02920:0.09202,Pv10Lk02960:0.04084)0.171:0.00499,(Pv10Lk03980:0.03654,(Pv10Lk03960:0.02599,Pv10Lk04030:0.04332)0.759:0.01343)0.999:0.02785)0.889:0.00014,(((Pv10Lk00060:0.03250,Pv10Lk00080:0.07049)0.988:0.02362,(((Pv10Lk03800:0.05406,Pv10Lk03820:0.05559)0.842:0.00912,Pv10Lk03810:0.04830)0.423:0.00502,((Pv10Lk03790:0.06721,Pv10Lk03830:0.05528)0.856:0.00751,Pv10Lk03850:0.06129)0.041:0.00016)0.917:0.00586)0.757:0.00277,(((Pv10Lk00070:0.06325,Pv10Lk00030:0.04782)0.316:0.00375,(Pv10Lk02900:0.04236,Pv10Lk03910:0.05111)0.456:0.00689)0.933:0.00014,(((((Pv10Lk02640:0.07006,Pv10Lk02650:0.07109)0.945:0.01532,(((Pv05Sk00010:0.01860,Pv10Lk00020:0.02720)1.000:0.04186,Pv10Lk02930:0.07099)0.899:0.00015,(((Pv10Lk02660:0.06905,(Pv10Lk00110:0.04188,Pv10Lk00130:0.04138)0.908:0.01354)0.777:0.00353,(((Pv10Lk04050:0.05133,Pv10Lk04080:0.02649)0.999:0.02972,(Pv10Lk02970:0.04515,Pv10Lk03010:0.03782)0.797:0.00437)0.851:0.00449,((Pv10Lk03940:0.07084,Pv10Lk04000:0.03979)0.999:0.03003,(Pv10Lk01610:0.04784,(Pv10Lk01460:0.06352,(Pv10Lk01510:0.07735,(Pv10Lk01450:0.03830,Pv10Lk01480:0.06403)0.938:0.01341)0.215:0.00659)0.897:0.01302)0.271:0.00256)1.000:0.00014)0.754:0.00117)0.942:0.00648,Pv10Lk03860:0.09532)0.747:0.00153)0.985:0.00897)0.781:0.00015,(((Pv10Lk02620:0.05295,Pv10Lk02670:0.06405)0.938:0.01432,(Pv10Lk02630:0.06119,(Pv10Lk02680:0.02684,Pv10Lk02750:0.05114)0.874:0.01926)0.476:0.00472)0.734:0.00131,((Pv10Lk00100:0.03311,Pv10Lk00120:0.03500)0.999:0.03786,((Pv10Lk02950:0.06912,Pv10Lk02990:0.05452)0.872:0.01468,(((Pv05Sk00020:0.03630,Pv10Lk04020:0.05399)0.717:0.00060,(Pv10Lk03840:0.03096,Pv10Lk04010:0.03003)0.999:0.03123)0.600:0.00348,(Pv10Lk00050:0.06410,Pv10Lk02980:0.06903)0.893:0.00015)0.855:0.00441)0.813:0.00015)0.756:0.00014)0.074:0.00014)0.191:0.00014,((((Pv10Lk01490:0.05925,Pv10Lk02910:0.07153)0.884:0.00881,Pv10Lk00660:0.03933)0.674:0.00014,(Pv10Lk04040:0.02219,(Pv10Lk03970:0.03227,Pv10Lk03990:0.01615)0.996:0.03030)1.000:0.05400)0.751:0.00161,(Pv10Lk03930:0.08777,(Pv10Lk01500:0.05533,Pv10Lk00040:0.05754)0.541:0.00719)0.755:0.00177)0.757:0.00120)0.175:0.00016,(((Pv10Lk03880:0.07051,Pv10Lk03900:0.08933)0.281:0.01231,(Pv10Lk03870:0.07867,Pv10Lk03890:0.06999)0.846:0.01057)0.893:0.00758,((Pv10Lk00090:0.06836,(Pv10Lk02890:0.06569,Pv10Lk03000:0.05209)0.028:0.00389)0.729:0.00336,(Pv10Lk03920:0.05332,(Pv10Lk01440:0.02783,Pv10Lk01470:0.06179)0.725:0.00727)0.782:0.00315)0.772:0.00228)0.772:0.00016)0.268:0.00016)0.431:0.00015)0.193:0.00014)0.744:0.00170)0.957:0.00752)0.781:0.00200)0.897:0.00015)0.422:0.00015)0.901:0.00014)0.789:0.00187)0.875:0.00180)0.831:0.00014,((((Pv10Sk04850:0.04883,(Pv10Sk04840:0.03322,Pv10Sk04860:0.04481)0.962:0.01637)0.167:0.00374,((Pv11Lk00770:0.04254,(Pv11Lk00810:0.03907,(Pv11Lk00800:0.06620,(Pv11Lk00780:0.01581,Pv11Lk00790:0.01447)0.997:0.01929)0.953:0.00841)0.935:0.00015)0.955:0.00631,((Pv11Lk03800:0.04012,((Pv11Lk03790:0.03094,Pv11Lk03690:0.03086)0.827:0.00294,(((Pv11Lk03750:0.02567,(Pv11Lk03720:0.02943,Pv11Lk03740:0.03613)0.837:0.00461)0.848:0.00310,(Pv11Lk03700:0.02417,Pv11Lk03710:0.03107)0.791:0.00329)0.773:0.00157,((Pv11Lk03670:0.05302,Pv11Lk03680:0.03420)0.910:0.00910,(Pv11Lk03730:0.03112,Pv11Lk03760:0.02935)0.900:0.00831)0.744:0.00132)0.862:0.00315)0.757:0.00127)0.973:0.00836,Pv11Lk00480:0.06198)0.751:0.00144)0.737:0.00217)0.950:0.00719,(((Pv05Sk00710:0.07112,((((((Pv10Sk04830:0.06520,(PvA11Bk00190:0.02509,(Pv11Lk01060:0.00101,PvA11Bk00250:0.06478)0.996:0.03141)0.948:0.01287)0.781:0.00352,(((((Pv11Lk03460:0.02488,Pv11Lk03480:0.05864)0.787:0.00431,Pv05Sk00680:0.04095)0.811:0.00317,(Pv11Lk00290:0.03600,(Pv05Sk00690:0.05419,Pv11Lk03450:0.04262)0.774:0.00469)0.850:0.00016)0.921:0.00145,(Pv11Lk00340:0.05697,Pv11Lk00380:0.05199)0.246:0.00597)1.000:0.00016,((((Pv11Lk05760:0.02351,(Pv05Sk01000:0.02058,Pv04Lk07050:0.05649)0.850:0.00478)0.983:0.01371,((Pv11Lk01070:0.00791,(Pv11Lk01170:0.00014,PvA11Bk00350:0.00014)0.971:0.00955)1.000:0.02526,((Pv11Lk01090:0.00014,PvA11Bk00220:0.00011)1.000:0.07039,(Pv11Lk03500:0.04744,(Pv11Lk03470:0.00330,Pv11Lk03490:0.00634)0.949:0.01969)1.000:0.02682)0.734:0.00163)0.813:0.00265)0.772:0.00105,(PvA11Bk00200:0.02407,PvA11Bk00260:0.01450)0.988:0.01426)0.984:0.00014,(((((Pv05Sk01060:0.03606,Pv05Sk00700:0.05167)0.848:0.00500,(Pv11Lk05380:0.05578,Pv04Sk03590:0.05559)0.741:0.00075)0.874:0.00327,(((Pv04Sk03580:0.06198,Pv05Sk00660:0.04381)0.871:0.00021,(Pv05Sk01050:0.03129,Pv11Lk00330:0.04320)0.869:0.00606)0.908:0.00014,(Pv11Lk00350:0.06354,Pv11Lk00390:0.05478)0.435:0.00881)0.725:0.00142)0.782:0.00161,((Pv11Lk00400:0.04518,Pv11Lk00300:0.05330)0.867:0.00574,((((Pv04Sk03480:0.04197,PvA11Bk00230:0.04913)0.808:0.00382,(Pv11Lk00370:0.06515,((Pv11Lk00310:0.04921,((((Pv11Lk04030:0.01679,Pv11Lk04070:0.03307)0.933:0.01161,(Pv11Lk04050:0.02663,Pv11Lk04060:0.02398)0.908:0.00767)0.960:0.01077,(((Pv11Lk04020:0.02737,Pv11Lk04040:0.03904)0.910:0.00862,Pv11Lk04120:0.04230)0.922:0.00015,(Pv11Lk04090:0.02877,((Pv11Lk04150:0.04297,Pv11Lk04170:0.02650)0.652:0.00063,((Pv11Lk04100:0.02912,Pv11Lk04110:0.02912)0.935:0.00888,(Pv11Lk04140:0.01942,Pv11Lk04160:0.01563)0.984:0.01268)0.762:0.00183)0.775:0.00150)0.796:0.00155)0.752:0.00120)0.835:0.00512,Pv11Lk04010:0.06581)0.458:0.00220)0.755:0.00173,(Pv11Lk00320:0.03781,Pv11Lk00360:0.05593)0.704:0.00122)0.783:0.00171)0.613:0.00171)0.751:0.00169,((((Pv04Lk06970:0.03516,(((Pv11Lk05490:0.03065,(Pv11Lk05480:0.01752,(Pv11Lk05460:0.02387,Pv11Lk05470:0.01762)0.835:0.00307)0.854:0.00306)0.844:0.00326,(Pv11Lk05520:0.02754,Pv11Lk05430:0.04366)0.736:0.00111)0.598:0.00156,(Pv11Lk05390:0.01682,Pv11Lk05450:0.01553)1.000:0.02389)0.747:0.00155)0.871:0.00325,(((Pv11Lk05170:0.02701,Pv11Lk05180:0.02951)0.971:0.01313,(Pv11Lk04990:0.02541,((Pv11Lk05030:0.01585,(((Pv11Lk05090:0.01007,(Pv11Lk05120:0.00591,Pv11Lk05130:0.01026)0.836:0.00522)0.814:0.00593,(Pv11Lk05040:0.00738,Pv11Lk05100:0.01622)0.759:0.00188)0.777:0.00184,((Pv11Lk05060:0.01742,(Pv11Lk05110:0.01755,Pv11Lk05140:0.02082)0.637:0.00302)0.404:0.00016,(Pv11Lk05050:0.02692,(Pv11Lk05150:0.04026,Pv11Lk05160:0.03923)0.825:0.00659)0.739:0.00183)0.452:0.00016)0.882:0.00341)0.717:0.00163,(Pv11Lk04980:0.02554,Pv11Lk05000:0.02493)0.352:0.00148)0.865:0.00334)0.958:0.00016)0.901:0.00014,(Pv11Lk04970:0.02053,Pv11Lk05440:0.04022)0.802:0.00341)0.800:0.00169)0.864:0.00337,(PvA11Bk00120:0.05624,((PvA11Bk00040:0.02914,PvA11Bk00050:0.05479)0.890:0.00676,(((PvA11Bk00060:0.02004,PvA11Bk00080:0.02476)0.997:0.02398,(PvA11Bk00110:0.02908,(PvA11Bk00030:0.02282,PvA11Bk00130:0.04107)0.878:0.00818)0.970:0.01447)0.947:0.00890,((PvA11Bk00020:0.02833,PvA11Bk00070:0.02905)0.817:0.01030,PvA11Bk00010:0.03666)0.707:0.00071)0.315:0.00014)0.747:0.00153)0.884:0.00336)0.933:0.00533,(Pv11Lk04080:0.04401,((((Pv05Sk01030:0.03742,(Pv11Lk06430:0.05041,(Pv05Sk01010:0.02130,Pv05Sk01020:0.03421)0.834:0.00401)0.684:0.00217)0.902:0.00011,(Pv04Lk06990:0.03548,Pv05Sk00670:0.04293)0.877:0.00846)0.717:0.00291,(Pv11Lk06440:0.02521,((((Pv11Lk06450:0.02162,Pv11Lk06460:0.01917)0.845:0.00474,((Pv11Lk06550:0.00627,(Pv11Lk06530:0.00779,(((Pv11Lk06470:0.01582,Pv11Lk06560:0.00483)0.350:0.00313,((Pv11Lk06520:0.00623,((Pv11Lk06480:0.00312,Pv11Lk06490:0.00945)0.889:0.00311,(Pv11Lk06510:0.00787,Pv11Lk06540:0.00472)0.767:0.00154)0.397:0.00156)0.875:0.00015,Pv11Lk06570:0.00626)0.921:0.00151)1.000:0.00015,(Pv11Lk06590:0.00160,(Pv11Lk06500:0.00312,Pv11Lk06580:0.00786)0.960:0.00627)0.287:0.00016)0.837:0.00314)0.914:0.00014)0.979:0.00807,(Pv04Lk06960:0.03501,Pv04Lk06980:0.04675)0.735:0.00067)0.786:0.00162)0.769:0.00162,(Pv04Lk07040:0.04738,Pv04Lk07020:0.03563)0.841:0.00015)0.775:0.00164,(Pv04Lk07010:0.02263,Pv04Lk07030:0.02816)0.925:0.00823)0.840:0.00353)0.944:0.00016)0.743:0.00186,(Pv11Lk04130:0.03115,Pv11Lk05500:0.04872)0.816:0.00438)0.056:0.00016)0.888:0.00371)0.148:0.00016)0.784:0.00015,(Pv11Lk05880:0.04443,(((((Pv11Lk05730:0.01592,Pv11Lk05740:0.02274)0.732:0.00201,Pv11Lk05800:0.02552)0.844:0.00014,((Pv11Lk05790:0.02234,((Pv11Lk05700:0.00793,Pv11Lk05750:0.01276)0.988:0.01426,(Pv11Lk05810:0.01295,Pv11Lk05820:0.02224)0.834:0.00469)0.845:0.00335)0.857:0.00313,Pv11Lk05710:0.02632)0.767:0.00159)0.870:0.00015,Pv11Lk05860:0.02065)0.136:0.00156,((Pv11Lk05830:0.02554,(Pv11Lk05780:0.01468,((Pv11Lk05840:0.01112,Pv11Lk05870:0.01605)0.418:0.00190,Pv11Lk05850:0.02169)0.423:0.00173)0.848:0.00371)0.775:0.00221,Pv11Lk05770:0.02741)0.763:0.00016)0.768:0.00157)0.911:0.00471)0.237:0.00015)0.000:0.00015)0.607:0.00015,(((Pv11Lk00820:0.03783,Pv11Lk00830:0.02966)0.929:0.00788,(Pv04Sk03470:0.06722,(Pv11Lk05420:0.03421,(Pv11Lk05400:0.03779,Pv11Lk05410:0.04570)0.252:0.00606)0.915:0.00726)0.538:0.00191)0.786:0.00182,(((Pv11Lk04740:0.01259,(Pv11Lk04310:0.02111,Pv11Lk04320:0.00574)0.891:0.00570)0.800:0.00014,(Pv11Lk04330:0.03081,Pv11Lk04340:0.01755)0.839:0.00306)0.993:0.00922,(Pv11Lk04730:0.03400,((Pv11Lk04700:0.01115,Pv11Lk04720:0.02351)0.991:0.01832,(Pv11Lk04670:0.01748,(Pv11Lk04680:0.03038,(Pv11Lk04690:0.02317,Pv11Lk04710:0.01387)0.662:0.00182)0.854:0.00336)0.592:0.00015)0.921:0.00720)0.415:0.00370)0.844:0.00014)0.794:0.00154)0.485:0.00013)0.587:0.00014)0.180:0.00012)0.860:0.00218,(PvA11Bk00280:0.05128,((PvA11Bk00290:0.04005,(Pv11Lk01130:0.00014,PvA11Bk00310:0.00014)1.000:0.03257)0.982:0.01831,((Pv11Lk01160:0.00014,PvA11Bk00340:0.00014)1.000:0.05261,PvA11Bk00300:0.05990)0.823:0.00623)0.254:0.00194)0.748:0.00139)0.734:0.00014,Pv05Sk00650:0.03225)0.432:0.00260,(Pv05Sk00990:0.02909,Pv11Lk01180:0.05919)0.921:0.00964)0.895:0.00687,((Pv11Lk02570:0.0,PvA11Ek00100:0.0):0.08658,Pv04Lk05060:0.06377)0.618:0.00118)0.829:0.00544)0.863:0.00550,((Pv11Lk00520:0.06218,Pv11Lk00530:0.04343)0.892:0.00890,Pv11Lk02080:0.09275)0.628:0.00122)0.754:0.00182,(((Pv11Lk01930:0.06529,Pv11Lk01950:0.07044)0.830:0.00786,((Pv11Lk02020:0.05148,Pv11Lk02050:0.05946)0.969:0.01707,(Pv11Lk02400:0.05346,(Pv11Lk02030:0.07946,(Pv11Lk01980:0.07356,Pv11Lk02420:0.05493)0.764:0.01187)0.298:0.00314)0.817:0.00439)0.266:0.00014)0.647:0.00015,(((Pv11Lk01840:0.07216,(Pv11Lk01860:0.06173,Pv11Lk02320:0.06229)0.499:0.00388)0.850:0.00594,(Pv11Lk01810:0.03785,(Pv11Lk01800:0.05183,Pv11Lk01820:0.07911)0.601:0.00517)0.768:0.00281)0.849:0.00015,((Pv11Lk02360:0.08365,((Pv11Lk02090:0.09437,Pv11Lk02370:0.05484)0.344:0.00841,((Pv11Lk02310:0.10678,Pv11Lk02250:0.10241)0.099:0.00015,((Pv11Lk02300:0.07598,(Pv11Lk02280:0.05184,(Pv11Lk02260:0.06167,(Pv11Lk02270:0.04165,Pv11Lk02290:0.05511)0.226:0.00405)0.464:0.00363)0.906:0.00707)0.922:0.00844,(((Pv11Lk02220:0.07056,(Pv11Lk02180:0.04105,(Pv11Lk02160:0.04057,(Pv11Lk02170:0.02072,Pv11Lk02210:0.03438)0.346:0.00309)0.843:0.00338)0.848:0.00673)0.992:0.02300,((Pv11Lk02120:0.05645,Pv11Lk02240:0.04773)0.953:0.01513,(Pv11Lk02140:0.05385,Pv11Lk02190:0.04352)0.889:0.00827)0.928:0.01014)0.974:0.01157,(Pv11Lk02130:0.05502,(Pv11Lk02150:0.06069,(Pv11Lk02200:0.02327,Pv11Lk02230:0.03754)0.890:0.00890)0.993:0.02171)0.965:0.01277)0.736:0.00043)0.803:0.00174)0.953:0.00015)0.281:0.00302)0.862:0.00744,(Pv11Lk01940:0.05605,Pv11Lk02100:0.07875)0.720:0.01357)0.575:0.00129)0.802:0.00171)0.955:0.00343)0.928:0.00016)0.325:0.00015,((((Pv10Sk04820:0.04949,((Pv05Sk01820:0.05331,(Pv05Sk01770:0.04678,Pv05Sk01740:0.03529)0.347:0.00257)0.336:0.00438,(((Pv05Sk01780:0.03663,(Pv05Sk01750:0.06670,Pv05Sk01790:0.06794)0.778:0.00760)0.923:0.00654,(Pv05Sk01810:0.03715,Pv05Sk01760:0.05287)0.798:0.00427)0.777:0.00235,Pv05Sk01800:0.04603)0.780:0.00162)0.777:0.00172)0.698:0.00090,Pv10Sk04810:0.05623)0.973:0.01116,(Pv11Lk00500:0.05136,(Pv11Lk00410:0.05683,(Pv11Lk00420:0.03622,Pv11Lk00430:0.04738)0.793:0.00511)0.683:0.00854)0.363:0.00545)0.243:0.00014,((Pv11Lk01590:0.00012,PvA11Dk00080:0.00014)1.000:0.09176,(Pv11Lk00470:0.07812,(Pv11Lk00440:0.05224,Pv11Lk00450:0.05957)0.913:0.00949)0.081:0.00014)0.787:0.00015)0.814:0.00153)0.639:0.00015)0.713:0.00035)0.810:0.00806)0.749:0.00016,((((Pv11Lk03510:0.09897,(Pv11Lk03550:0.03644,Pv11Lk03560:0.06748)0.765:0.00465)0.746:0.00118,((Pv11Lk03530:0.04769,(Pv10Lk03090:0.09574,((Pv11Lk02730:0.00014,PvA11Ek00240:0.00014)0.998:0.02918,(Pv11Lk02720:0.00014,PvA11Ek00230:0.00014)0.996:0.02530)0.996:0.03295)0.501:0.00285)0.854:0.00584,Pv11Lk03040:0.06640)0.326:0.00271)0.967:0.00016,(Pv11Lk04390:0.04639,(PvA11Ek00260:0.07279,(((Pv11Lk02950:0.0,PvA11Ek00520:0.0):0.04228,(((PvA11Ek00290:0.00786,PvA11Ek00300:0.00949)0.870:0.00310,(PvA11Ek00330:0.01892,PvA11Ek00340:0.02575)0.844:0.00308)0.935:0.00870,(PvA11Ek00320:0.02382,(PvA11Ek00310:0.00160,(Pv11Lk02770:0.00485,PvA11Ek00350:0.01220)0.999:0.02291)0.753:0.00152)0.703:0.00393)0.932:0.02416)0.819:0.00825,(((Pv11Lk03000:0.05307,(((Pv11Lk03010:0.06484,(Pv11Lk02700:0.00014,PvA11Ek00220:0.00014)1.000:0.04955)0.129:0.00045,(Pv11Lk02990:0.00015,PvA11Ek00550:0.00014)1.000:0.04746)0.807:0.00347,(Pv11Lk02980:0.00014,PvA11Ek00540:0.00014)1.000:0.05481)0.768:0.00333)0.971:0.00015,((Pv11Lk02960:0.05295,(((Pv11Lk02940:0.00014,PvA11Ek00510:0.00014)1.000:0.05580,(Pv11Lk02900:0.00014,PvA11Ek00480:0.00014)1.000:0.04204)0.861:0.00718,(Pv11Lk02930:0.00014,PvA11Ek00500:0.00016)0.999:0.04597)0.413:0.00163)0.896:0.00015,((((((Pv11Lk02790:0.00014,PvA11Ek00370:0.00014)1.000:0.05363,(Pv11Lk02820:0.00155,PvA11Ek00390:0.00014)1.000:0.03416)0.968:0.00014,(PvA11Ek00440:0.02578,(Pv11Lk02870:0.00014,PvA11Ek00450:0.00015)0.979:0.01458)0.986:0.02899)0.932:0.00564,((Pv11Lk02860:0.0,PvA11Ek00430:0.0):0.03639,(Pv11Lk02890:0.00014,PvA11Ek00470:0.00015)0.999:0.02411)0.829:0.00434)0.767:0.00200,((Pv11Lk02780:0.00014,PvA11Ek00360:0.00014)1.000:0.05499,((Pv11Lk02850:0.0,PvA11Ek00420:0.0):0.05816,((Pv11Lk02880:0.00014,PvA11Ek00460:0.00014)1.000:0.03572,((Pv11Lk02830:0.00014,PvA11Ek00400:0.00014)0.998:0.02936,((Pv11Lk02800:0.0,PvA11Ek00380:0.0):0.02897,Pv11Lk02810:0.01381)0.966:0.01186)0.890:0.00472)0.764:0.00246)0.663:0.00236)0.770:0.00104)1.000:0.00016,(Pv11Lk02840:0.00014,PvA11Ek00410:0.00014)1.000:0.04781)0.898:0.00319)0.817:0.00154)0.134:0.00016,((Pv11Lk02690:0.00015,PvA11Ek00210:0.00014)1.000:0.04447,(Pv11Lk02970:0.00014,PvA11Ek00530:0.00014)1.000:0.05995)0.866:0.00015)0.881:0.00014)0.858:0.00451)0.911:0.00483)0.812:0.00614)0.829:0.00015,(Pv11Lk03540:0.07068,(Pv11Lk03030:0.08024,Pv11Lk04370:0.07000)0.036:0.00529)0.771:0.00340)0.760:0.00298)0.750:0.00360,Pv11Lk05580:0.07315)0.783:0.00353)0.930:0.00015)0.108:0.00015,(((Pv11Lk00460:0.06482,(Pv04Sk02650:0.05836,Pv11Lk01850:0.05411)0.538:0.00842)0.727:0.00014,(Pv11Lk00580:0.07722,Pv11Lk00600:0.05588)0.933:0.01461)0.044:0.00216,Pv11Lk03520:0.08293)0.758:0.00172)0.423:0.00015);
