# Supplementary material for: The Subtelomeric khipu Satellite Repeat from Phaseolus vulgaris: Lessons Learned from the Genome Analysis of the Andean Genotype G19833
Source: Front Plant Sci. 2013 Oct 16;4:109. doi: 10.3389/fpls.2013.00109 (PMC3797529; doi:10.3389/fpls.2013.00109)
Supplement: Supplementary file 4 [file 47451_Geffroy_Presentation_1.PPTX]

## Slide 1
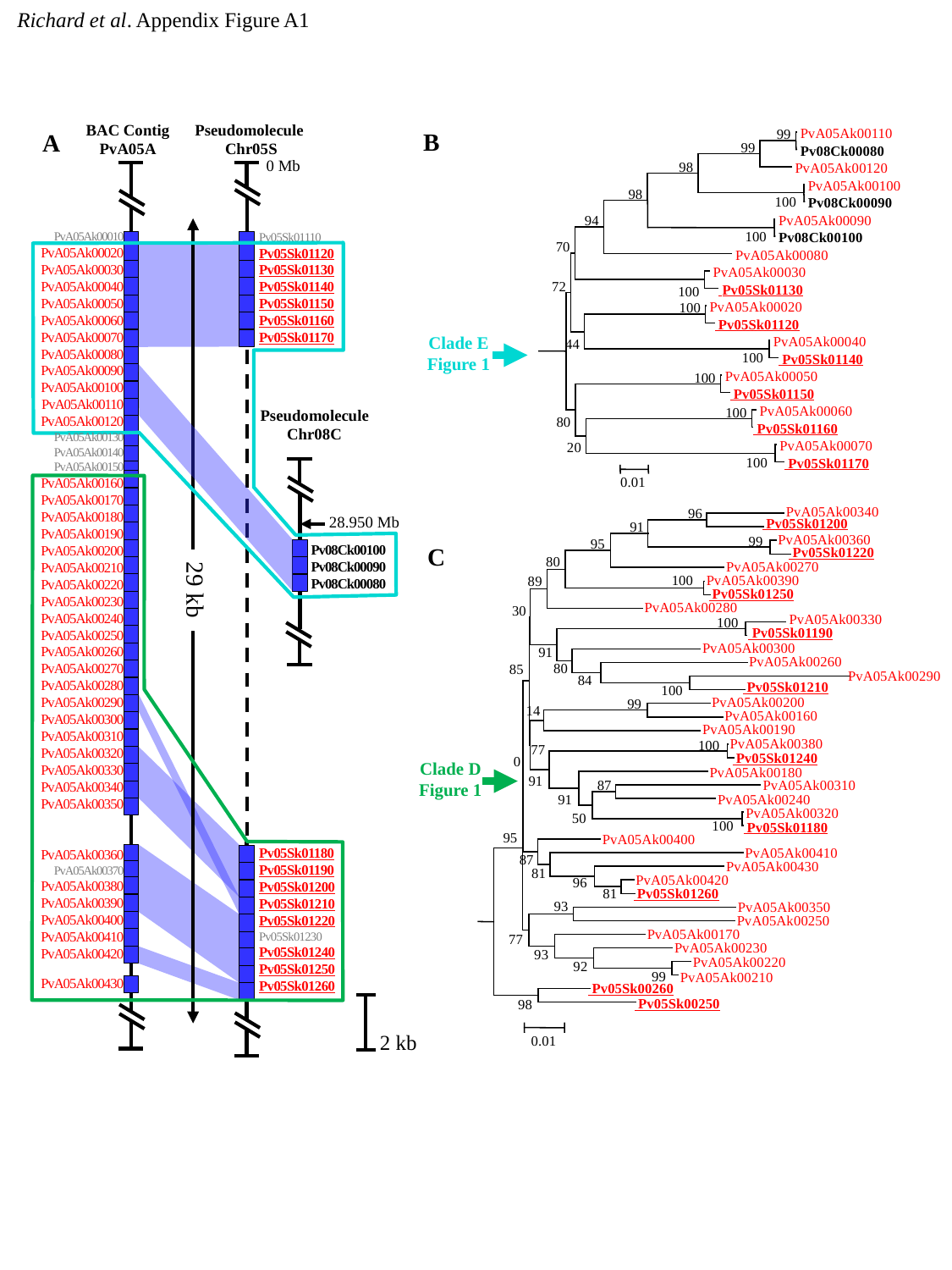

Richard et al. Appendix Figure A1
 PvA05Ak00110
99
99
 Pv08Ck00080
98
 PvA05Ak00120
 PvA05Ak00100
98
100
 Pv08Ck00090
 PvA05Ak00090
94
100
 Pv08Ck00100
70
 PvA05Ak00080
 PvA05Ak00030
72
 Pv05Sk01130
100
 PvA05Ak00020
100
 Pv05Sk01120
 PvA05Ak00040
44
100
 Pv05Sk01140
 PvA05Ak00050
100
 Pv05Sk01150
 PvA05Ak00060
100
80
 Pv05Sk01160
 PvA05Ak00070
20
100
 Pv05Sk01170
0.01
BAC Contig
PvA05A
Pseudomolecule
Chr05S
B
A
0 Mb
PvA05Ak00010
PvA05Ak00020
PvA05Ak00030
PvA05Ak00040
PvA05Ak00050
PvA05Ak00060
PvA05Ak00070
PvA05Ak00080
PvA05Ak00090
PvA05Ak00100
PvA05Ak00110
PvA05Ak00120
PvA05Ak00130
PvA05Ak00140
PvA05Ak00150
PvA05Ak00160
PvA05Ak00170
PvA05Ak00180
PvA05Ak00190
PvA05Ak00200
PvA05Ak00210
PvA05Ak00220
PvA05Ak00230
PvA05Ak00240
PvA05Ak00250
PvA05Ak00260
PvA05Ak00270
PvA05Ak00280
PvA05Ak00290
PvA05Ak00300
PvA05Ak00310
PvA05Ak00320
PvA05Ak00330
PvA05Ak00340
PvA05Ak00350
PvA05Ak00360
PvA05Ak00370
PvA05Ak00380
PvA05Ak00390
PvA05Ak00400
PvA05Ak00410
PvA05Ak00420
PvA05Ak00430
Pv05Sk01110
Pv05Sk01120
Pv05Sk01130
Pv05Sk01140
Pv05Sk01150
Pv05Sk01160
Pv05Sk01170
Pv05Sk01180
Pv05Sk01190
Pv05Sk01200
Pv05Sk01210
Pv05Sk01220
Pv05Sk01230
Pv05Sk01240
Pv05Sk01250
Pv05Sk01260
Clade E
Figure 1
Pseudomolecule Chr08C
 PvA05Ak00340
96
 Pv05Sk01200
91
 PvA05Ak00360
99
95
 Pv05Sk01220
80
 PvA05Ak00270
 PvA05Ak00390
100
89
 Pv05Sk01250
 PvA05Ak00280
30
 PvA05Ak00330
100
 Pv05Sk01190
 PvA05Ak00300
91
 PvA05Ak00260
80
85
 PvA05Ak00290
84
 Pv05Sk01210
100
 PvA05Ak00200
99
14
 PvA05Ak00160
 PvA05Ak00190
 PvA05Ak00380
100
77
 Pv05Sk01240
0
 PvA05Ak00180
91
 PvA05Ak00310
87
 PvA05Ak00240
91
 PvA05Ak00320
50
100
 Pv05Sk01180
95
 PvA05Ak00400
 PvA05Ak00410
87
 PvA05Ak00430
81
 PvA05Ak00420
96
81
 Pv05Sk01260
93
 PvA05Ak00350
 PvA05Ak00250
 PvA05Ak00170
77
 PvA05Ak00230
93
 PvA05Ak00220
92
99
 PvA05Ak00210
 Pv05Sk00260
 Pv05Sk00250
98
0.01
28.950 Mb
Pv08Ck00100
Pv08Ck00090
Pv08Ck00080
C
29 kb
Clade D
Figure 1
2 kb
