# Supplementary material for: The Subtelomeric khipu Satellite Repeat from Phaseolus vulgaris: Lessons Learned from the Genome Analysis of the Andean Genotype G19833
Source: Front Plant Sci. 2013 Oct 16;4:109. doi: 10.3389/fpls.2013.00109 (PMC3797529; doi:10.3389/fpls.2013.00109)
Supplement: Supplementary file 5 [file 47451_Geffroy_Presentation2.PPTX]

## Slide 1
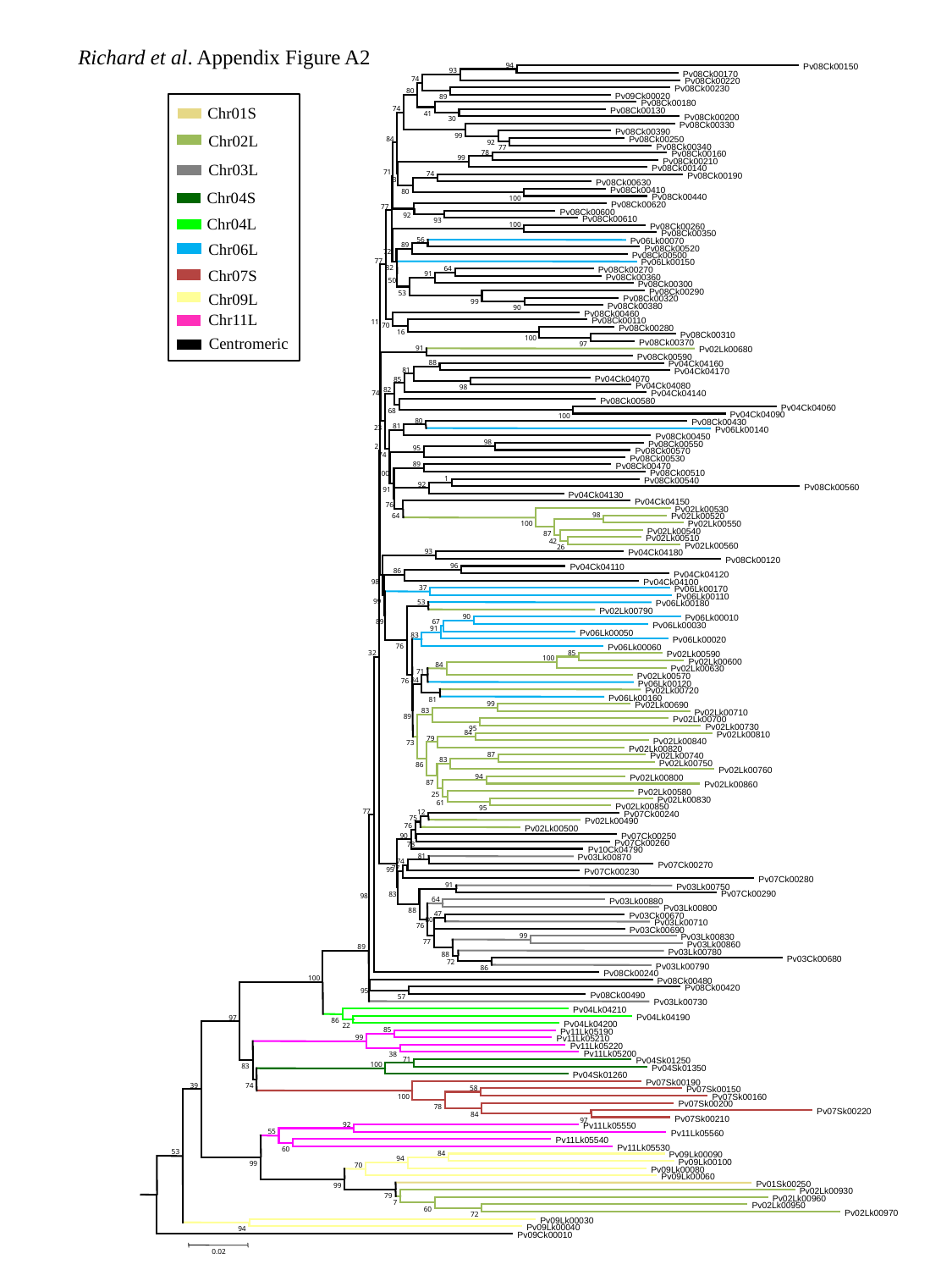

Richard et al. Appendix Figure A2
94
93
74
80
89
74
41
30
99
84
92
77
78
99
71
74
3
80
100
77
92
93
100
56
89
72
77
82
64
91
50
53
99
90
11
70
16
100
97
91
88
81
85
98
82
74
68
100
80
81
23
98
95
74
89
100
1
92
91
76
98
64
100
87
42
26
93
96
86
37
53
90
67
91
83
85
100
84
71
84
81
 Pv08Ck00480
 Pv08Ck00420
 Pv08Ck00490
 Pv03Lk00730
 Pv04Lk04210
 Pv04Lk04190
 Pv04Lk04200
 Pv11Lk05190
 Pv11Lk05210
 Pv11Lk05220
 Pv11Lk05200
 Pv04Sk01250
 Pv04Sk01350
 Pv04Sk01260
 Pv07Sk00190
 Pv07Sk00150
 Pv07Sk00160
 Pv07Sk00200
 Pv07Sk00220
 Pv07Sk00210
 Pv11Lk05550
 Pv11Lk05560
 Pv11Lk05540
 Pv11Lk05530
 Pv09Lk00090
 Pv09Lk00100
 Pv09Lk00080
 Pv09Lk00060
 Pv01Sk00250
 Pv02Lk00930
 Pv02Lk00960
 Pv02Lk00950
 Pv02Lk00970
 Pv09Lk00030
 Pv09Lk00040
 Pv09Ck00010
 Pv08Ck00150
 Pv08Ck00170
 Pv08Ck00220
 Pv08Ck00230
 Pv09Ck00020
 Pv08Ck00180
 Pv08Ck00130
 Pv08Ck00200
 Pv08Ck00330
 Pv08Ck00390
 Pv08Ck00250
 Pv08Ck00340
 Pv08Ck00160
 Pv08Ck00210
 Pv08Ck00140
 Pv08Ck00190
 Pv08Ck00630
 Pv08Ck00410
 Pv08Ck00440
 Pv08Ck00620
 Pv08Ck00600
 Pv08Ck00610
 Pv08Ck00260
 Pv08Ck00350
 Pv06Lk00070
 Pv08Ck00520
 Pv08Ck00500
 Pv06Lk00150
 Pv08Ck00270
 Pv08Ck00360
 Pv08Ck00300
 Pv08Ck00290
 Pv08Ck00320
 Pv08Ck00380
 Pv08Ck00460
 Pv08Ck00110
 Pv08Ck00280
 Pv08Ck00310
 Pv08Ck00370
 Pv02Lk00680
 Pv08Ck00590
 Pv04Ck04160
 Pv04Ck04170
 Pv04Ck04070
 Pv04Ck04080
 Pv04Ck04140
 Pv08Ck00580
 Pv04Ck04060
 Pv04Ck04090
 Pv08Ck00430
 Pv06Lk00140
 Pv08Ck00450
 Pv08Ck00550
 Pv08Ck00570
 Pv08Ck00530
 Pv08Ck00470
 Pv08Ck00510
 Pv08Ck00540
 Pv08Ck00560
 Pv04Ck04130
 Pv04Ck04150
 Pv02Lk00530
 Pv02Lk00520
 Pv02Lk00550
 Pv02Lk00540
 Pv02Lk00510
 Pv02Lk00560
 Pv04Ck04180
 Pv08Ck00120
 Pv04Ck04110
 Pv04Ck04120
 Pv04Ck04100
 Pv06Lk00170
 Pv06Lk00110
 Pv06Lk00180
 Pv02Lk00790
 Pv06Lk00010
 Pv06Lk00030
 Pv06Lk00050
 Pv06Lk00020
 Pv06Lk00060
 Pv02Lk00590
 Pv02Lk00600
 Pv02Lk00630
 Pv02Lk00570
 Pv06Lk00120
 Pv02Lk00720
 Pv06Lk00160
 Pv02Lk00690
 Pv02Lk00710
 Pv02Lk00700
 Pv02Lk00730
 Pv02Lk00810
 Pv02Lk00840
 Pv02Lk00820
 Pv02Lk00740
 Pv02Lk00750
 Pv02Lk00760
 Pv02Lk00800
 Pv02Lk00860
 Pv02Lk00580
 Pv02Lk00830
 Pv02Lk00850
 Pv07Ck00240
 Pv02Lk00490
 Pv02Lk00500
 Pv07Ck00250
 Pv07Ck00260
 Pv10Ck04790
 Pv03Lk00870
 Pv07Ck00270
 Pv07Ck00230
 Pv07Ck00280
 Pv03Lk00750
 Pv07Ck00290
 Pv03Lk00880
 Pv03Lk00800
 Pv03Ck00670
 Pv03Lk00710
 Pv03Ck00690
 Pv03Lk00830
 Pv03Lk00860
 Pv03Lk00780
 Pv03Ck00680
 Pv03Lk00790
 Pv08Ck00240
2
98
99
89
76
32
76
99
83
89
95
84
79
73
87
83
86
94
87
25
61
95
77
12
75
76
90
78
81
74
77
95
91
83
98
64
88
47
80
76
99
77
89
88
72
86
100
95
57
97
86
22
85
99
38
71
100
83
39
74
58
100
78
84
97
92
55
60
53
84
94
99
70
99
79
7
60
72
94
0.02
Chr01S
Chr02L
Chr03L
Chr04S
Chr04L
Chr06L
Chr07S
Chr09L
Chr11L
Centromeric
