# Supplementary figures and images for: The Subtelomeric khipu Satellite Repeat from Phaseolus vulgaris: Lessons Learned from the Genome Analysis of the Andean Genotype G19833
Source: Front Plant Sci. 2013 Oct 16;4:109. doi: 10.3389/fpls.2013.00109 (PMC3797529; doi:10.3389/fpls.2013.00109)

## Slide 1
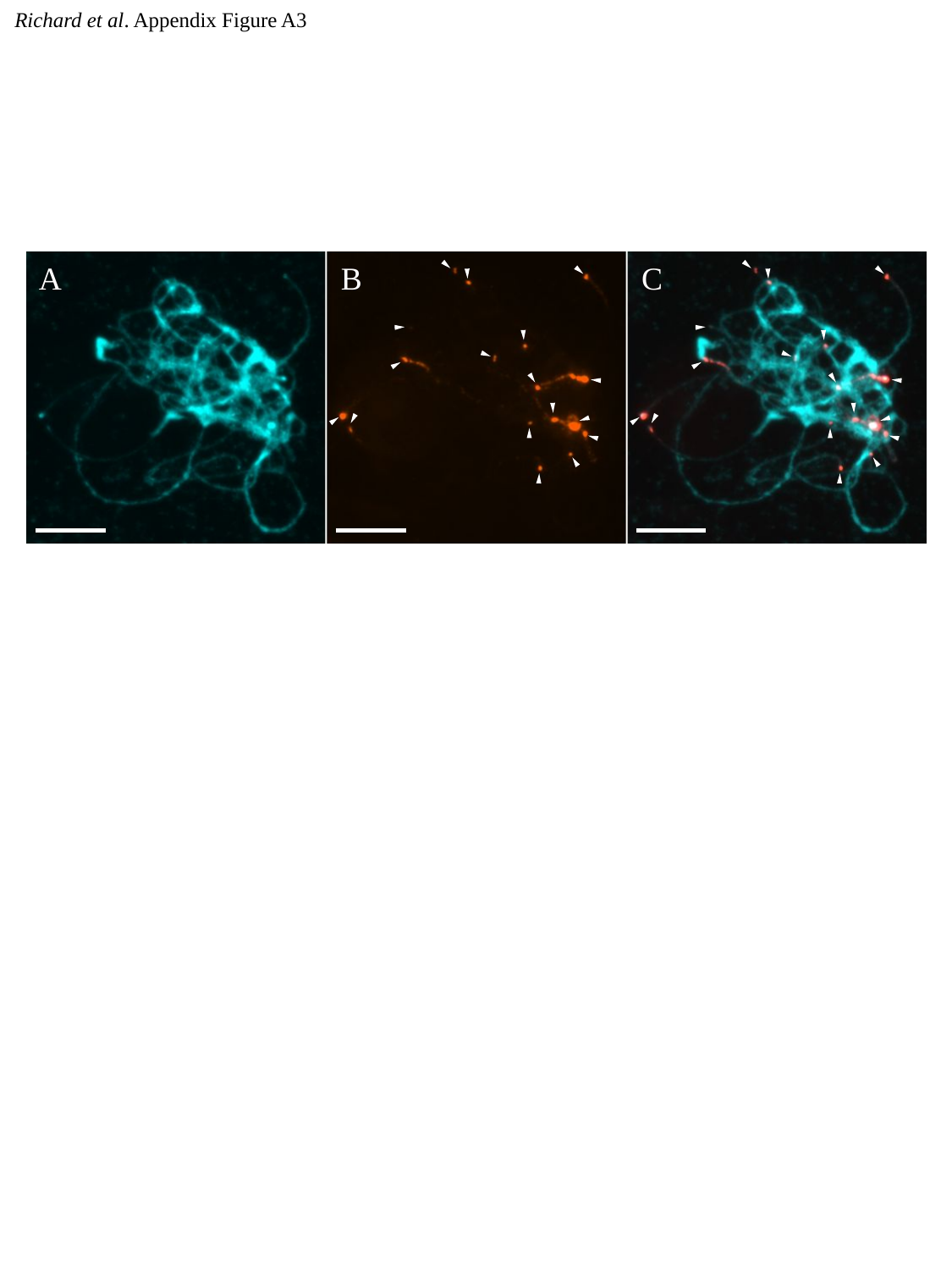

Richard et al. Appendix Figure A3
A
B
C

Supplement: Supplementary file 6 [file 47451_Geffroy_Presentation3.PPTX]
