# Supplementary material for: The Subtelomeric khipu Satellite Repeat from Phaseolus vulgaris: Lessons Learned from the Genome Analysis of the Andean Genotype G19833
Source: Front Plant Sci. 2013 Oct 16;4:109. doi: 10.3389/fpls.2013.00109 (PMC3797529; doi:10.3389/fpls.2013.00109)
Supplement: Supplementary file 7 [file 47451_Geffroy_Presentation4.PPTX]

## Slide 1
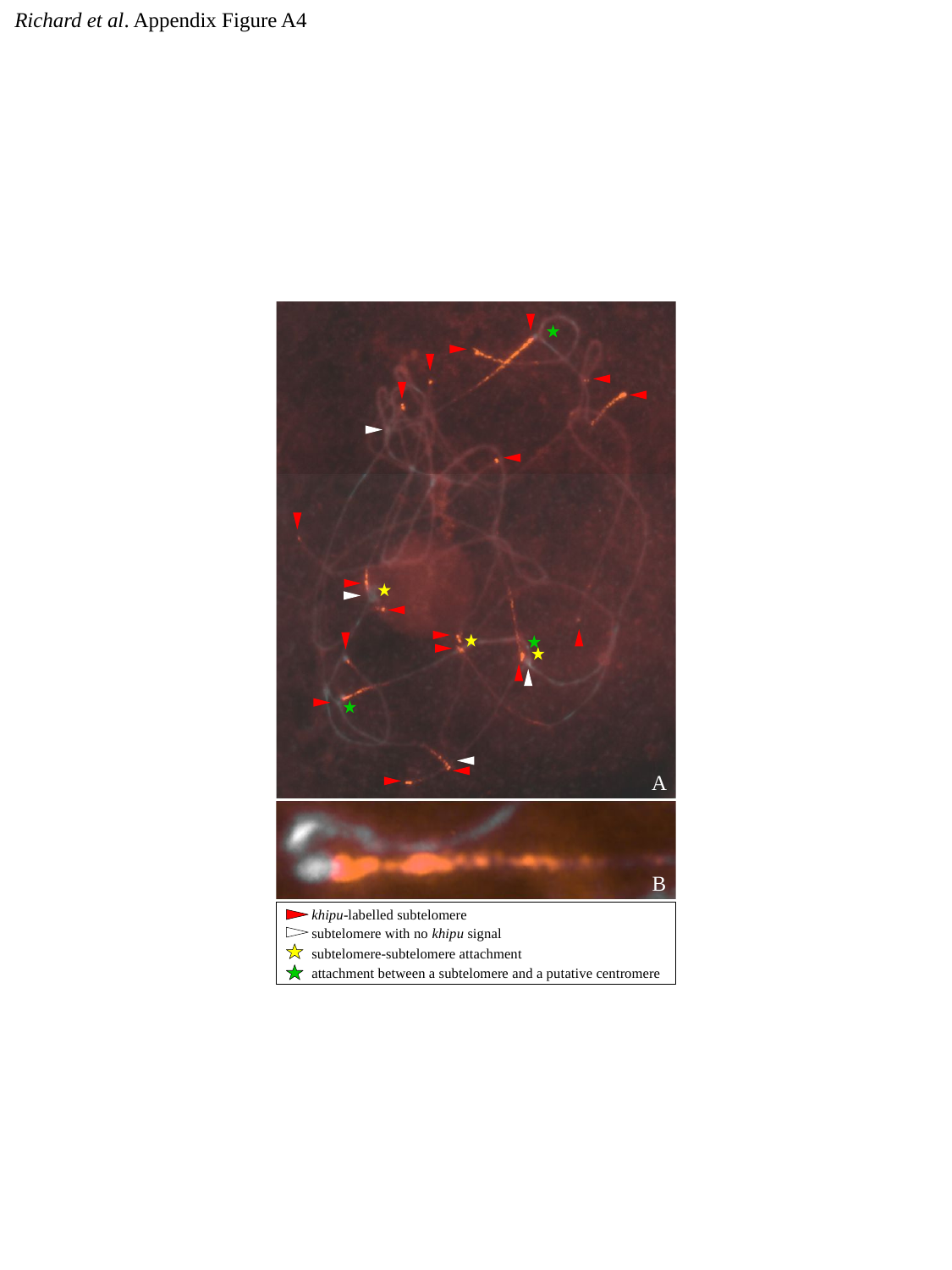

Richard et al. Appendix Figure A4
A
B
khipu-labelled subtelomere
subtelomere with no khipu signal
subtelomere-subtelomere attachment
attachment between a subtelomere and a putative centromere
