# Supplementary material for: The Subtelomeric khipu Satellite Repeat from Phaseolus vulgaris: Lessons Learned from the Genome Analysis of the Andean Genotype G19833
Source: Front Plant Sci. 2013 Oct 16;4:109. doi: 10.3389/fpls.2013.00109 (PMC3797529; doi:10.3389/fpls.2013.00109)
Supplement: Supplementary file 8 [file 47451_Geffroy_Presentation5.PPTX]

## Slide 1
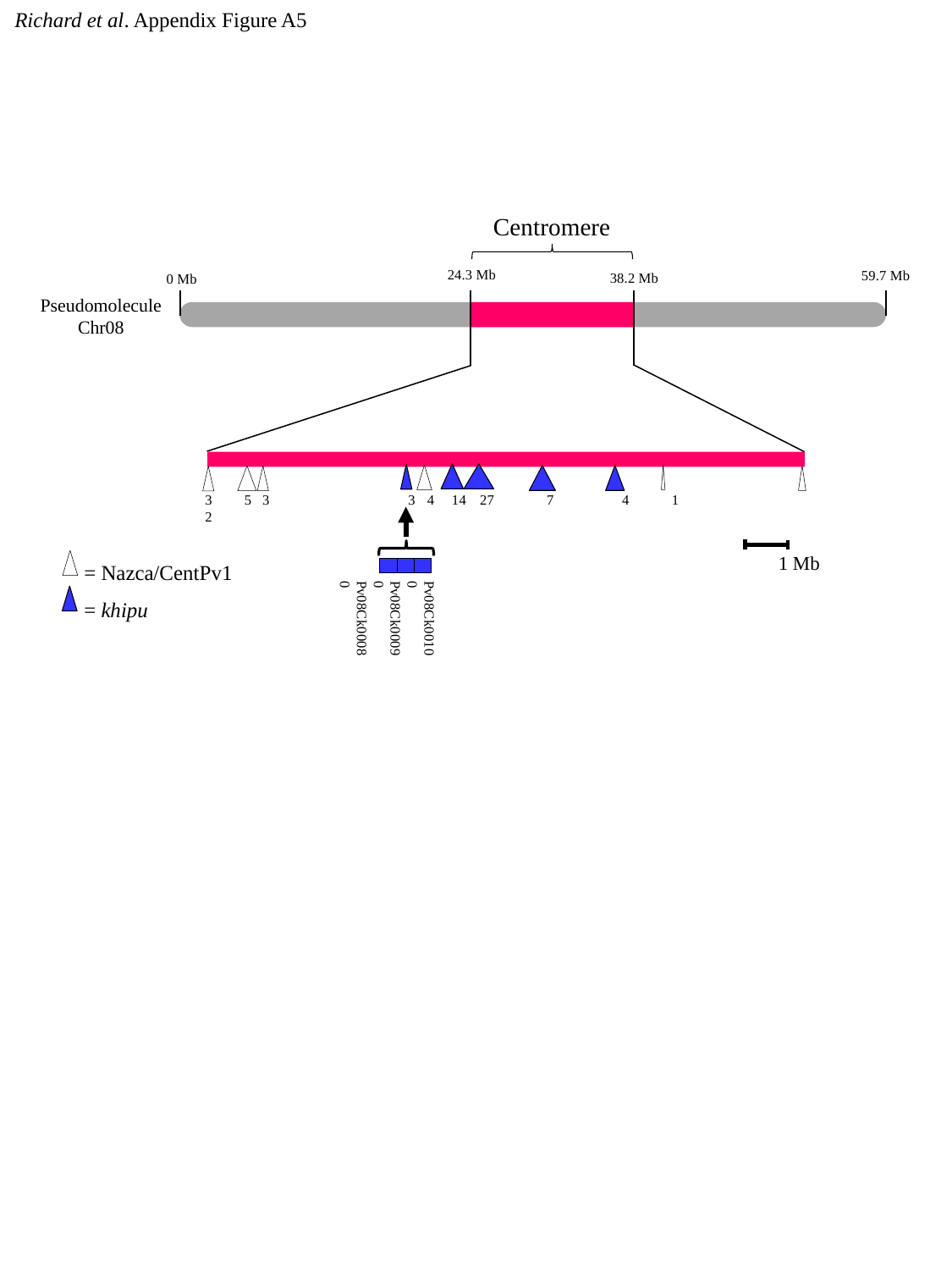

Richard et al. Appendix Figure A5
Centromere
24.3 Mb
59.7 Mb
38.2 Mb
0 Mb
Pseudomolecule Chr08
3 5 3 3 4 14 27 7 4 1 2
= Nazca/CentPv1
= khipu
1 Mb
Pv08Ck00100
Pv08Ck00090
Pv08Ck00080
