# Supplementary material for: The Subtelomeric khipu Satellite Repeat from Phaseolus vulgaris: Lessons Learned from the Genome Analysis of the Andean Genotype G19833
Source: Front Plant Sci. 2013 Oct 16;4:109. doi: 10.3389/fpls.2013.00109 (PMC3797529; doi:10.3389/fpls.2013.00109)
Supplement: Supplementary file 9 [file 47451_Geffroy_Presentation6.PPTX]

## Slide 1
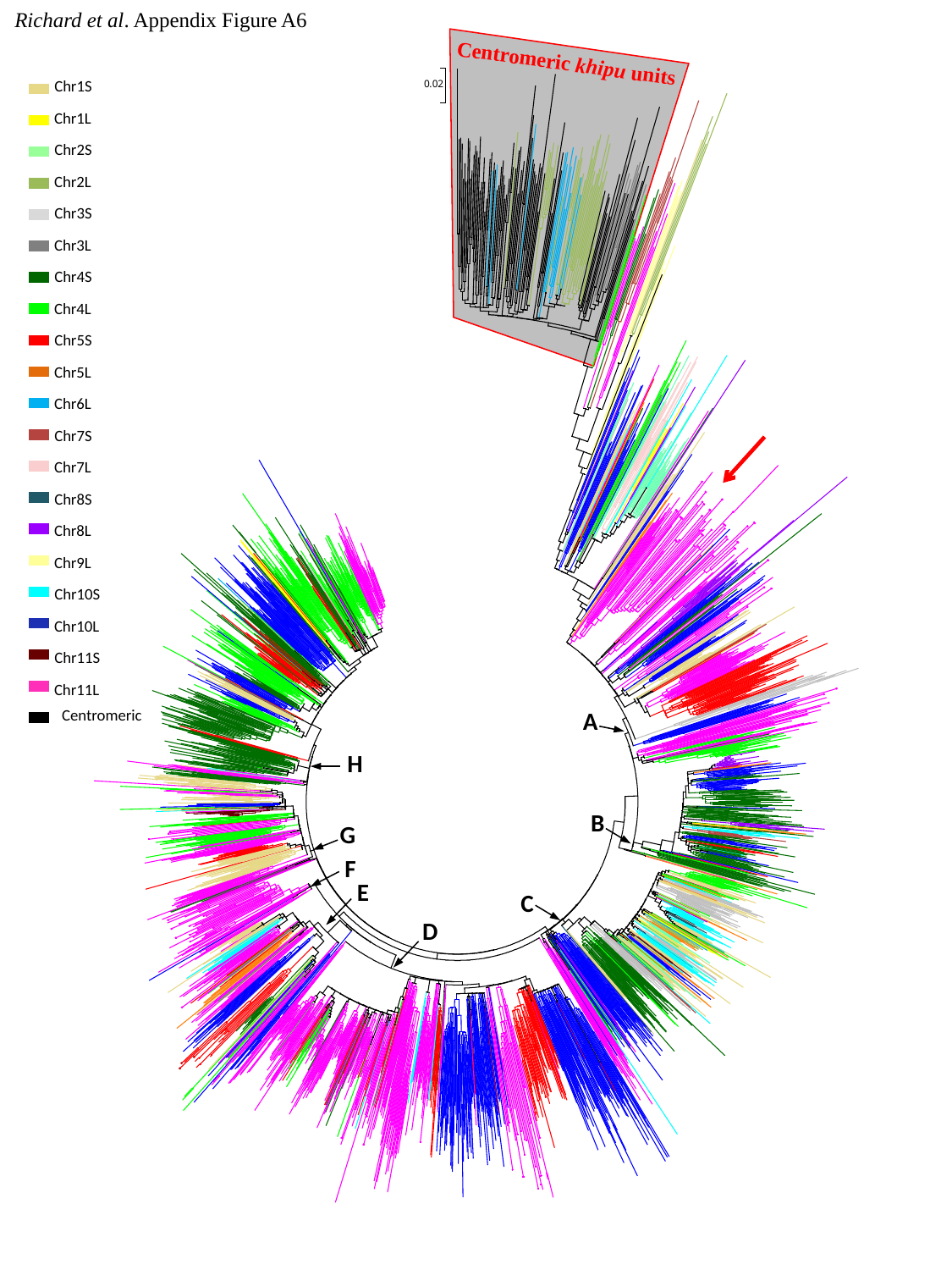

Richard et al. Appendix Figure A6
Centromeric khipu units
Chr1S
Chr1L
Chr2S
Chr2L
Chr3S
Chr3L
Chr4S
Chr4L
Chr5S
Chr5L
Chr6L
Chr7S
Chr7L
Chr8S
Chr8L
Chr9L
Chr10S
Chr10L
Chr11S
Chr11L
Centromeric
A
H
B
G
F
E
C
D
